# Derivation of Xeno-Free and GMP-Grade Human Embryonic Stem Cells – Platforms for Future Clinical Applications

**DOI:** 10.1371/journal.pone.0035325

**Published:** 2012-06-20

**Authors:** Shelly E. Tannenbaum, Tikva Tako Turetsky, Orna Singer, Einat Aizenman, Sophie Kirshberg, Nili Ilouz, Yaniv Gil, Yael Berman-Zaken, Temima Schnitzer Perlman, Nitshia Geva, Ora Levy, Daniel Arbell, Alex Simon, Assaf Ben-Meir, Yoel Shufaro, Neri Laufer, Benjamin E. Reubinoff

**Affiliations:** 1 The Hadassah Human Embryonic Stem Cell Research Center, Goldyne Savad Institute of Gene Therapy, Hadassah Hebrew University Medical Center, Jerusalem, Israel; 2 Department of Obstetrics and Gynecology, Hadassah Hebrew University Medical Center, Jerusalem, Israel; 3 Department of Pediatric Surgery, Hadassah Hebrew University Medical Center, Jerusalem, Israel; University of Southern California, United States of America

## Abstract

Clinically compliant human embryonic stem cells (hESCs) should be developed in adherence to ethical standards, without risk of contamination by adventitious agents. Here we developed for the first time animal-component free and good manufacturing practice (GMP)-compliant hESCs. After vendor and raw material qualification, we derived xeno-free, GMP-grade feeders from umbilical cord tissue, and utilized them within a novel, xeno-free hESC culture system. We derived and characterized three hESC lines in adherence to regulations for embryo procurement, and good tissue, manufacturing and laboratory practices. To minimize freezing and thawing, we continuously expanded the lines from initial outgrowths and samples were cryopreserved as early stocks and banks. Batch release criteria included DNA-fingerprinting and HLA-typing for identity, characterization of pluripotency-associated marker expression, proliferation, karyotyping and differentiation in-vitro and in-vivo. These hESCs may be valuable for regenerative therapy. The ethical, scientific and regulatory methodology presented here may serve for development of additional clinical-grade hESCs.

## Introduction

Human ESC lines [1],[2] holds promise for disease modeling, basic scientific research, drug development, toxicity studies, and may serve as an unlimited renewable source of cells for transplantation therapy.

Most of the reported hESC lines worldwide are not ideal for use in clinical trials. They were developed without adherence to Good Manufacture Practices (GMPs), using animal-derived research-grade reagents, which may infect the cells with animal pathogens. Moreover, many cell lines were derived and cultured on animal feeder cells, which may contaminate the hESCs by nonhuman sialic acid Neu5Gc molecules, which can elicit immune rejection after transplantation [3] and may render them as xenotransplantation products [4],[5]. In order to use hESC derivatives for clinical applications, the hESCs (clinical-grade hESCs) should ideally be developed under stringent ethical guidelines, from traceable and tested donors, preferably in an animal-free, GMP-grade culture system. The hESCs should meet safety criteria including a normal karyotype, definitive identity profile, sterility and the absence of adventitious viruses [6],[5].

Modifications and improvements of specific components of the hESC culture systems, which may enable their use for developing clinical-grade hESCs have been reported. To avoid immunosurgery, which utilizes animal reagents, ICMs were isolated from blastocysts by mechanical [7],[4],[8],[9] or laser-assisted-dissection [10]. Alternatively, whole blastocysts were plated and the pluripotent stem cells were isolated from the outgrowth [11],[10]. The use of mouse embryonic fibroblast feeders was replaced by human fibroblasts [12],[13],[14],[15], feeders derived from hESCs [15], outgrowths of embryoid bodies [16], or by the development of feeder-free defined culture systems [17],[18],[19],[20]. To further avoid xenogenic components in the culture system, fetal calf serum was replaced by human serum [15] or xeno-free serum replacement [21],[22]. Recombinant [22] or synthetic [23] extracellular matrices (ECMs), growth factors [24] and enzymes for passaging were introduced [25],[26]. A single group successfully developed hESC lines under xeno-free conditions. However, although GMP-grade materials were utilized the derivation was not performed within a cleanroom environment [27].

In order to utilize stem cells for cell therapy, they preferably should be developed under strict cleanroom conditions, utilizing GMP-grade reagents and compulsory documentation [5]. So far, only a single group [24] fully complied with these requirements and derived hESC lines under GMP conditions; however, animal products were used in the feeder culture medium as well as the hESC culture system.

Here we report for the first time the derivation of clinical-grade hESC lines developed in an animal-free and GMP-compliant culture system under cleanroom conditions. Donor eligibility was carefully screened, in adherence to regulatory guidelines. All aspects of donor tissue and embryo handling, hESC derivation, culturing, cryopreservation, banking and characterization, were strictly monitored for quality practices, in line with their future use in transplantation therapy. The data, protocols and documentation presented here may serve as a platform for the development of additional clinical-grade hESCs.

## Results

### Qualification of Materials

The study was initiated by the establishment of a quality assurance program to identify materials which would be both xeno-free and GMP-grade. Xeno-free candidate materials were tested in our feeder and hESC culture systems for their effectiveness in replacing research-grade reagents. The vendors of effective xeno-free materials underwent a QA certification process to ensure compliance with GMPs. To further confirm the vendors’ quality statements we arbitrarily sampled some of the materials for sterility, mycoplasma and LAL testing. At the completion of this certification process, all animal-free materials that were accepted were also GMP-grade, except for FGF2 which was available only as a GMP-like product (Table S1).

### Clinical-grade Human Feeders

To avoid the use of feeders from mouse origin, we developed and compared feeders from three primary human tissues including foreskin, aborted fetuses and umbilical cord. All donated tissues were obtained subject to informed consent and Institutional Helsinki Committee approval. Foreskin was obtained during surgical circumcision, tissues from aborted fetuses were collected from first trimester terminations of pregnancy and umbilical cord was harvested during elective cesarean sections. All tissues were obtained under sterile conditions from surgical procedures performed in an operating room. This enabled us to avoid the use of antibiotics in the feeder culture system, as advised by the FDA [28], to prevent potential allergic reaction from trace antibiotic remnants in future transplanted hESC-derived progeny.

Fibroblast feeder lines were derived from the three primary tissues with a culture system containing humanized or recombinant, GMP-grade reagents. After qualifying the derivation process with all ancillary materials [29] by developing feeder lines from the three sources under research conditions, clinical-grade lines were developed in Hadassah’s GMP Facility, in accordance with FDA Aseptic Processing Guidelines [30] and European Agency for the Evaluation of Medicinal product (EMEA [31]) guidelines. Three GMP-grade umbilical cord, two foreskin, and one fetal fibroblast master cell banks (MCBs) were derived. Short tandem repeat (STR) DNA fingerprinting provided a unique identification profile for each line.

**Figure 1:**
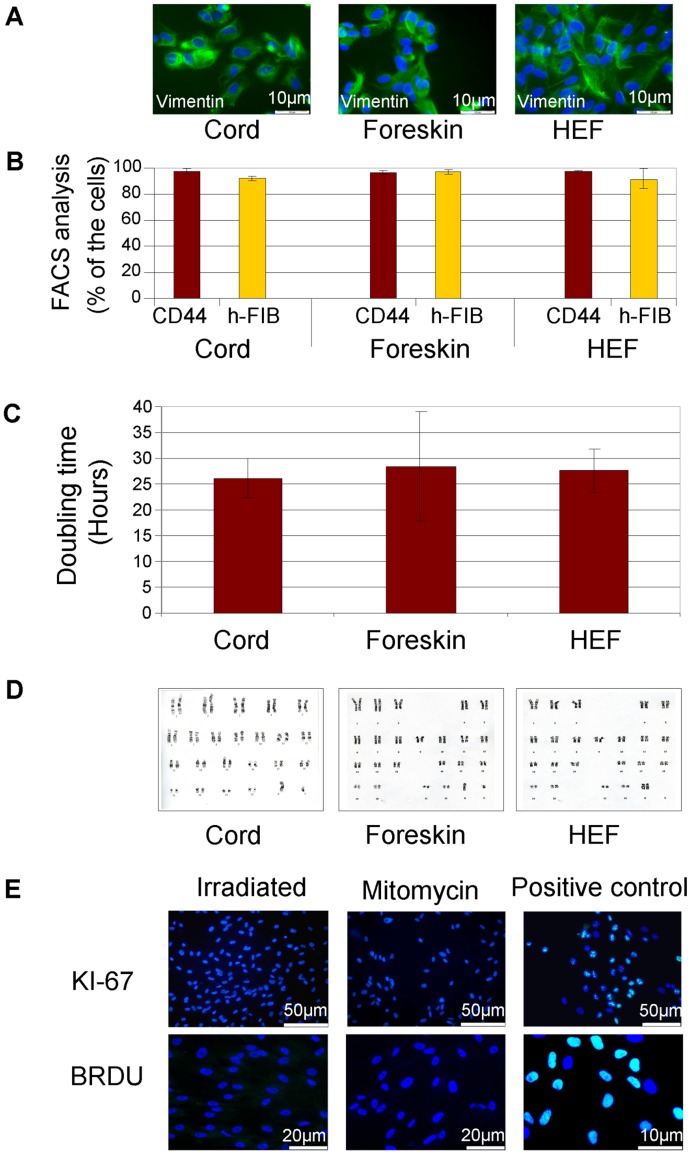
Characterization of human feeders. Human feeders derived from three types of human tissues showed similar properties. They were immunoreactive with anti-vimentin (A; immunostaining, nuclei counterstained with 4', 6-diamidino-2-phenylindole (DAPI)), CD44 and human Fibroblast antigens (B; FACS analysis). They had similar doubling times (C) and normal karyotypes (D). γ-irradiated cord feeders (WCB2) were mitotically inactive as indicated by lack of KI-67 expression and BrdU incorporation (E; immunostaining (green), nuclei counterstained with DAPI (blue)). Mitomycin-C treated and mitotically-active non-treated feeders served as negative and positive controls, respectively. (B & C present analysis of three lines from each feeder type).

We characterized and compared the properties of the feeders derived from the three primary tissues between passages 5–10 in a GMP-compliant QC testing laboratory. Feeders from the three sources showed typical fibroblast morphology and were immunostained for vimentin, which is expressed by fibroblasts (Figure 1A). FACS analysis showed that the majority (>80%) of the cells were immunoreactive with anti-CD44 and anti-human fibroblast antibodies (Figure 1B). The fibroblast average doubling times were 26.1±3.8 hours (umbilical cord), 29.9±9.8 hours (foreskin) and 27.2±3.7 hours (human fetal fibroblasts; n = 3: Figure 1C). All three feeder types exhibited normal karyotypes when a minimum of 30 metaphase spreads were analyzed at passages 7–10 (Figure 1D).

**Figure 2:**
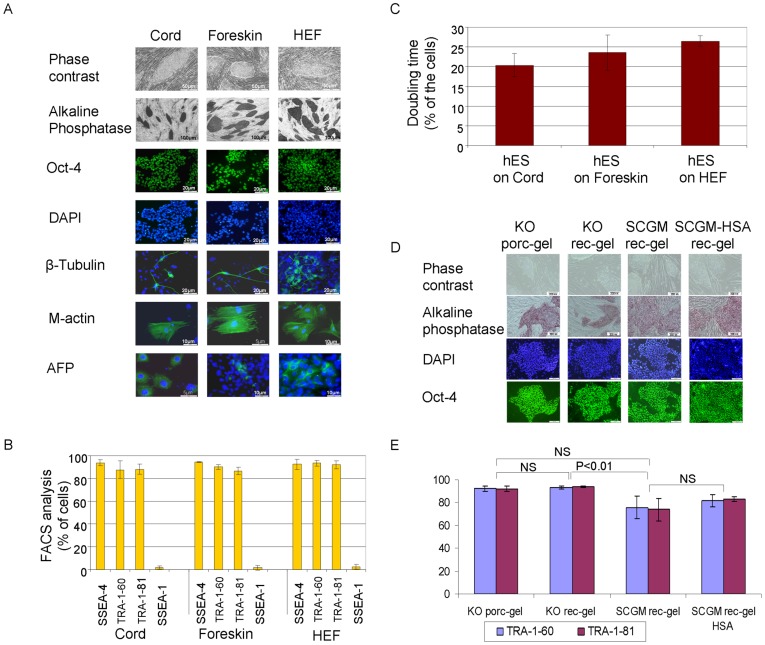
The development of a clinical-grade hESCs culture system. Feeders derived from the three types of human tissues were equally effective in maintaining undifferentiated pluripotent growth of hESCs (HES-1[2]) for a minimum of 5 passages within research-grade KO medium. (A) The hESCs cultured on the three types of feeders formed colonies with typical morphology (phase contrast images), expressed alkaline phosphatase (AP), and were immunoreactive with anti-Oct-4 (nuclei were counterstained with DAPI). They were pluripotent as demonstrated by their potential to differentiate in-vitro into progeny representing the three germ lineages. Immunofluorescence staining showed differentiated cells expressing beta-tubulin III (ectoderm), muscle actin (mesoderm) and alpha-Feto-Protein (AFP, endoderm). (B) FACS analysis showing that the percentage of cells expressing markers of pluripotent human stem cells, and the level of background differentiation (percentage of SSEA-1 expressing cells) was similar (n = 3). (C) The hESC doubling time did not differ significantly after culture on the three types of feeders (n = 3). Cord feeders were further used for the development of the clinical grade culture system, and the phenotype of hESCs was compared after culture within research-grade KO medium system and following replacement and supplementation with xeno-free and GMP-grade reagents. The following culture compositions were compared: hESCs cultured on porcine gelatin (porc-gel) or in recombinant gelatin (rec-gel) in KO medium, SCGM and SCGM supplemented with HSA (SCGM-HSA). (D) With all culture conditions, the hESCs colonies had similar and typical morphological characteristics (phase contrast images), and the stem cells expressed alkaline phosphatase activity (red) and Oct-4 (green; blue-DAPI nuclear counterstaining; immunofluorescence images). (E) The percentage of cells expressing Tra-1-60 and Tra-1-81 was analyzed by FACS and compared between the various culture conditions (n = 4). Scale bar in (D) represents 200 um.

To test and compare the potential of the feeders to support the propagation of pluripotent stem cells, hESCs were cultured on the three types of feeders pretreated with Mitomycin C. The phenotype and differentiation potential of the hESCs was characterized after 1–5 passages, and to ascertain the long-term potential of the feeders to support undifferentiated propagation also again after 6–10 passages. On the three types of feeders, the characteristics of hESCs were similar at the early and late analysis time points. The cultured hESCs retained the morphology of undifferentiated cells and expressed alkaline phosphatase (AP) and Oct-4 (Figure 2A). FACS analysis showed a comparable high percentage (>80%) of cells expressing SSEA-4, TRA-1-60, and TRA-1-81, while a minute percentage expressing SSEA-1 (Figure 2B). The normal karyotype of the hESCs was ascertained by G-banding before and after their passaging on the feeders. The doubling time of hESCs on the three types of feeders did not differ significantly (Figure 2C).

The hESCs could differentiate *in vitro* within embryoid bodies and neural spheres into progeny representing the three embryonic germ layers (Figure 2A). Thus, the potential of the feeders from the three sources to support undifferentiated propagation of hESCs was similar. The full characterization of GMP-grade feeder lines is summarized in Table S2.

We elected to use the cord as the source of feeders since it was not associated with ethical issues such as those related to fetal tissue donation, and was sterile, unlike disinfected foreskin. Samples of the MCB frozen at passage level 3 of the cord feeder line CRD008 were sent to an FDA-approved laboratory for safety testing according to FDA, EMEA, and International Conference on Harmonisation (ICH) [32] guidelines. The cells were found to be free of bacteria, fungi, yeasts, mycoplasmas, and adventitious viruses. Isoenzyme analysis confirmed that the feeders were from a human source. Karyotype analysis was normal (46XY), and endotoxin results (LAL) were within an acceptable range [33]; Table S3).

Four samples of the MCB were expanded and frozen after γ-irradiation at passages 7–8 as four working cell banks (WCBs). The lack of proliferating cells within thawed samples of the WCBs was confirmed by KI-67 immunostaining and analysis of BrdU incorporation (Figure 1E). The WCBs underwent characterization (Table S4) similar to the MCB and limited safety testing (Table S3). The derivation of three cord feeder MCBs and four WCBs enabled us to meet regulatory requirements for three runs of process validation under aseptic cleanroom conditions.

In conclusion, we developed for the first time xeno-free, GMP-compliant and safety-tested clinical-grade human feeders from cord tissue.

### Clinical-grade hESC Culture System

Towards further developing a xeno-free, GMP-grade hESC culture system, while utilizing the irradiated cord feeders, we replaced porcine-skin-derived research-grade gelatin with recombinant gelatin. It did not adversely affect the undifferentiated propagation of the hESCs in research grade KO medium. After 4 passages, qualitative analysis of the morphology of colonies, Oct-4 and alkaline phosphatase expression (Figure 2D) and quantitative analysis of the percentage of cells expressing markers of pluripotency was similar with both gelatins (Figure 2E). In the next step, we identified a commercially available serum-free stem cell growth medium (SCGM; CellGro®/CellGenix™) which is designed for the expansion of hematopoietic and natural killer cells. SCGM is produced under GMP, is animal-free and is intended for clinical ex vivo cell culture use.

We compared the potential of SCGM (supplemented with FGF2) with KO medium to support undifferentiated propagation of hESCs on cord feeders and recombinant gelatin. After 4 passages, the morphology of colonies was similar and the stem cells expressed Oct-4 and AP (Figure 2D). The percentage of cells expressing TRA-1-60 and TRA-1-81 was 75.6±4.9% and 74±4.9%, respectively, with SCGM, significantly (P<0.01) lower compared to KO Medium. Upon supplementation of the SCGM with human serum albumin (HSA), the percentage of undifferentiated cells improved to 81.4±2.7% and 83±1%, respectively. These percentages were slightly lower but not significantly different in comparison to KO medium (Figure 2E). The doubling time of hESCs was similar in SCGM supplemented with FGF2 and HSA compared to the KO-based culture system (23.75±2.5 (n = 6)), compared to 23.30±1.8 (n = 8), respectively).

We next analyzed the feasibility of cryopreservation of hESC clusters by both slow cooling and vitrification methods using animal-free GMP-grade reagents. Slow cooling was performed either within human serum supplemented with 10% DMSO or a commercially available FDA-approved cryo-solution medium (CryoStor®). Ten clusters of hESCs were cryopreserved per vial. With both solutions, undifferentiated hESCs could be retrieved and propagated after thawing from each tested vial (n = 5 vials per solution). The percentage of cryopreserved hESC clusters that survived and gave rise to undifferentiated colonies upon plating was similar with human serum-DMSO and Cryostore solutions (31.4±4.34% and 32.6±4.52%, respectively). For practical reasons CryoStor® solution was further used for cryopreservation of hESCs.

Vitrification was conducted using commercially available solutions, which are FDA-approved for cryopreservation of embryos. Sealed straws (FDA-approved and CE marked) were utilized to avoid potential contamination from liquid nitrogen. Similar to slow cooling, undifferentiated hESCs could be retrieved and propagated from each thawed straw (5 vitrified clusters/straw; n = 6 straws). The percentage of surviving clusters that gave rise to undifferentiated colonies was 47±7.4%. These results showed that both slow cooling and vitrification methods that utilize xeno-free GMP-grade reagents allowed reliable retrieval of cryopreserved hESCs.

Thus we validated that the newly developed culture system could support the undifferentiated propagation and cryopreservation of hESCs and was ready for use under GMP conditions for the derivation of clinical-grade lines.

### Ethics of hESC Development

The study was approved by the Hadassah Medical Center IRB and the Israeli Ministry of Health’s National Ethics Committee for Genetic Research in Humans. Supernumerary IVF embryos not intended to be used for fertility purposes and which were cryopreserved for at least five years were eligible for recruitment to the study. This was in line with Israeli IVF regulations, which permit, subject to the couples’ consent, the discarding or donating to research of embryos frozen for five years. In order to ensure free informed consent, the team of physicians which treated the donors as IVF patients and obtained their consent was not the same team which was involved in the development of the hESC lines. In order to maintain strict confidentiality of the donors, they received a unique identifying code and all documents relating to them and hESCs produced from their embryos were labeled with it. Given that the derived hESCs were intended for clinical use, blood samples from the donors were archived, and a route was established to obtain in the future, if needed, subject to donor permission, additional medical information or blood tests. Approach of the donors for this purpose would only be possible after obtaining approval by the Hadassah Director of the Department of Gynecology. Donors were advised that they would not receive any information about the hESC lines, compensation or benefit for their donation, and would not influence or have any involvement in the use of the hESC lines.

### Recruitment, Screening and Monitoring of Embryo Donors

Potential donor couples who met the ethical eligibility criteria mentioned above received information about the study and their questions were answered. Those interested signed informed consent documents (Attachment 1, File S22) including an authorization to review their medical histories (File S25). The donors were screened according to the FDA’s Donor Eligibility Regulations for Human Tissues and Cells [34], including: (a) a physical examination; (b) review of donors’ medical records to rule out disqualifying medical conditions; (c) an interview/screen for lifestyle risk factors; and (d) serum and swab samples, which were sent for testing using FDA-approved test kits. Results of these tests (Table S5) were sent to the donors. Screening through interview was performed where no acceptable test kit existed, such as for vCJD, sepsis, West Nile, and Vaccinia viruses. All the information related to donor screening and the status and quality of the donated embryos were recorded in the donors’ Case Report Form (CRF). The CRFs and study forms used in the hESC trial are given in Table S6, and Files S2, S3, S4, S5, S6, S7, S8, S9, S10, S11, S12, S13, S14, S15, S16, S17, S18, S19, S20, S21, S22, S23, S24, S25, S26, S27, S28, S29, S30, S31, S32.

The trial was monitored for veracity by a clinical trial associate (CRA). Source data which included verification of the donors’ health histories was included in the CRFs for donors whose embryos resulted in usable hESC lines.

Donors with verified good health histories from preferred countries of origin (low variant Creutzfeldt-Jakob disease and malaria prevalence) and with low reported lifestyle risks were selected to participate in the program. Exclusion criteria included lifestyle and behavioral risks (File S28), or medications and diseases that could have affected the quality of the embryos (Files S30, S31).

### Derivation of Clinical Grade hESC Lines

One hundred embryos were recruited from 12 couples and were thawed in the IVF laboratory. 77 embryos survived thawing of which 34 developed to the blastocyst stage. Details regarding the developmental stage and morphological score of the blastocysts at the time of ICM isolation are summarized in Table S7. To avoid the use of animal-derived products, zona pellucida drilling and separation of the ICMs was performed with the assistance of laser according to our previously described protocol [10]. 23 ICMs were successfully isolated from blastocysts that had well developed ICMs. Eight blastocysts that either had poor ICMs or collapsed during ICM isolation were plated whole on the human feeders. Three blastocysts were damaged during the procedure and were discarded (Table S7).

The isolated ICMs and whole blastocysts were transferred from the IVF laboratory to the GMP-facility following Good Tissue Practices (GTPs), and were plated on the irradiated cord WCB feeders within the clinical-grade hESC culture medium. After 5–8 days, groups of small, tightly packed cells proliferated from the outgrowths of 7 ICMs and a single whole blastocyst. They were mechanically dissected from the outgrowth of differentiated cells and, following replating, gave rise to flat colonies of cells with the morphological appearance of hESCs. The colonies were expanded and early stocks were frozen. Given the difficulty in the expansion of undifferentiated colonies in five of the hESC derivations, we did not attempt to further propagate them beyond this stage. In three derivations, which were more easily propagated as undifferentiated colonies, the early passage (5–9) stocks were frozen by both vitrification and slow cooling methods. These stocks were frozen by both methods for backup reasons given the limited and comparable efficiencies of these methods under clinical-grade conditions. These three stocks (early banks: HAD-C 100, HAD-C 102 and HAD-C 106) underwent STR fingerprinting and HLA class I and II antigen profiling for identity determination, sterility testing and abbreviated characterization including, karyotyping, and limited analysis of the expression of markers of pluripotecy (Table S8).

**Figure 3:**
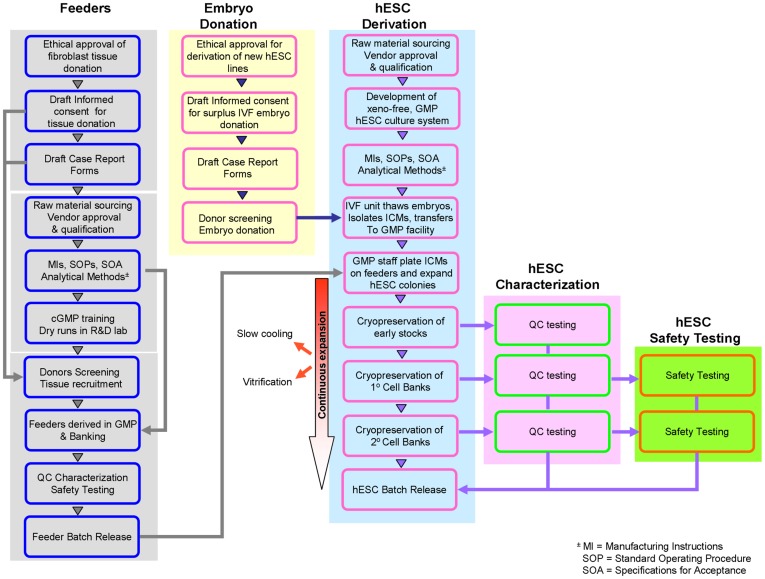
Process flow diagram of the approach taken in deriving xeno-free clinical-grade fibroblast feeders and hESCs. The scheme indicates the ethical, scientific, and regulatory steps taken in deriving our clinical-grade fibroblast feeders and hESCs.

The colonies of these three derivations were continuously expanded from the initial outgrowths to avoid repeated freezing and thawing. Samples were pooled and frozen at passages 11–24 as Primary Cell Banks (1° CB; by both cryopreservation methods; see Figures S1 and S2) and following additional expansion, cryopreserved (passages 16–33) as secondary CBs (2° CB by slow cooling) (Table S8, and Figure S3). Thus we followed the suggested two-tiered cell banking system (as described in [35]) with modifications. The derivation of three independent hESC CBs and their characterization, as described below, enabled us to meet regulatory requirements for three runs of process validation under aseptic cleanroom conditions. A flow diagram showing the steps we took in the derivation of our clinical-grade hESC lines is shown in Figure 3.

### Characterization of Clinical-grade hESCs Lines

Detailed characterization was conducted on the primary (HAD-C100, 102 and 106) and secondary (HAD-C-100) CBs that were frozen by slow cooling. For characterization, thawed samples of the clinical grade hESCs were cultured in the GLP laboratory in the same culture conditions as in the derivation process.

**Figure 4:**
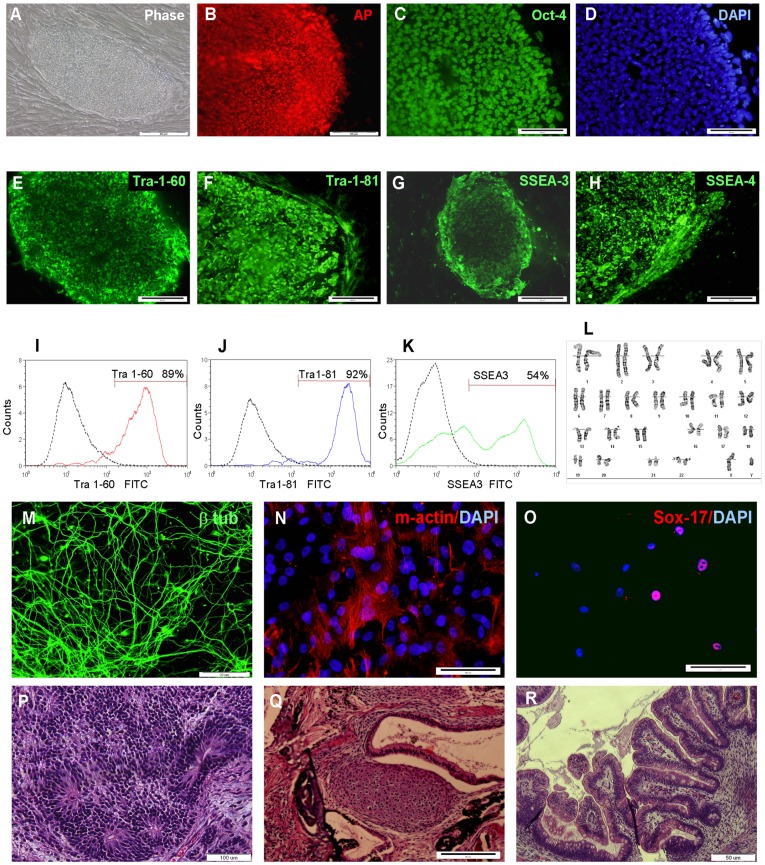
Characterization of HAD-C 100 1° cell bank. The hESCs colonies had the typical morphology of human pluripotent stem cell colonies with clear distinguishable borders from the cord feeder cells (A; phase-contrast image). The cells expressed alkaline phosphatase (B; AP, fluorescence image). Indirect immunofluorescence staining showed that the hESCs were immunoreactive with anti-Oct-4 (C; nuclei counterstained with DAPI, D), TRA-1-60 (E), TRA-1-81 (F), SSEA-3 (G), and SSEA-4 (H). FACS analysis showed that the majority of cells expressed markers of pluripotency TRA-1-60 (I), TRA-1-81 (J) and SSEA-3 (K) (Data from a representative experiment). The hESCs had normal karyotype (46, XY; L) and could differentiate *in vitro* and *in vivo* into cells representing the three embryonic germ layers (M-R). Immunofluorescence staining showing *in vitro* differentiated cells expressing beta-tubulin III (ectoderm, M), muscle actin (mesoderm, N) and sox-17 (endoderm, O). Hematoxylin-eosin stained histological sections of teratoma tumors showing neural rosettes (ectoderm, P), cartilage (mesoderm, Q) and villi structures with columnar glandular epithelium and goblet cells (endoderm, R). Scale bar represent 50 um for (A, B, Q and R), 100 um for (C, D, M, N, O and P) and 200 um for (E–H).

The hESCs colonies showed typical morphology and the karyotype of the three lines was normal. The STR and HLA profiles of the CBs were identical to the profiles of the early stocks, verifying the identities of the CBs (The STR and HLA profiles are not shown to maintain the confidential identity of the donors). The colonies showed alkaline phosphatase activity and were immunoreactive with anti Oct-4, TRA-1-60 TRA-1- 81, SSEA-3 and SSEA-4 antibodies. FACS analysis of markers for pluripotent hESCs of the 1° CBs and 2°CB showed that the majority of cells expressed TRA-1-60, TRA-1-81 and SSEA-3 (Figure 4 and Figures S1, S2, S3 and Table S8).

Background differentiation was evaluated by FACS analysis of the percentage of cells expressing SSEA-1 and was shown to be in the range of 5–36% for all lines (Table S8). KI-67 immunostaining showed that the majority of cells were in an active cell cycle (>87% of the cells stained positive for KI-67, Figure S4, Table S8). Doubling time of thawed hESCs from the 1° CBs was in the range of 35.6–43 hours, and the doubling time of thawed hESCs from the 2°CB of HADC-100 was similar to that of the 1° CBs (Table S8).

The pluripotent potential of the three hESCs lines was demonstrated by their capability to differentiate into progeny representing the three embryonic germ layers in-vitro and in-vivo. Following 3–4 weeks of differentiation as embryoid bodies (EBs), immunofluorescence staining showed cells expressing beta-tubulin III (neuronal marker, ectoderm), muscle actin (mesoderm) and sox-17 (endoderm; Figure 4M-O and Figures S1, S2, S3M–O).

When clusters of hESCs from the three lines were inoculated subcutaneously into SCID mice, teratoma tumors developed and were analyzed histologically after 8–12 weeks. Hematoxylin-eosin stained sections showed neural rosettes (ectoderm), cartilage (mesoderm) and columnar glandular epithelium with goblet cells (endoderm; Figure 4P–R and Figures S1, S2, S3P–R).

Safety testing for adventitious viruses of the hESC lines was negative and is shown in Table S9.

#### Gene expression analysis

**Figure 5:**
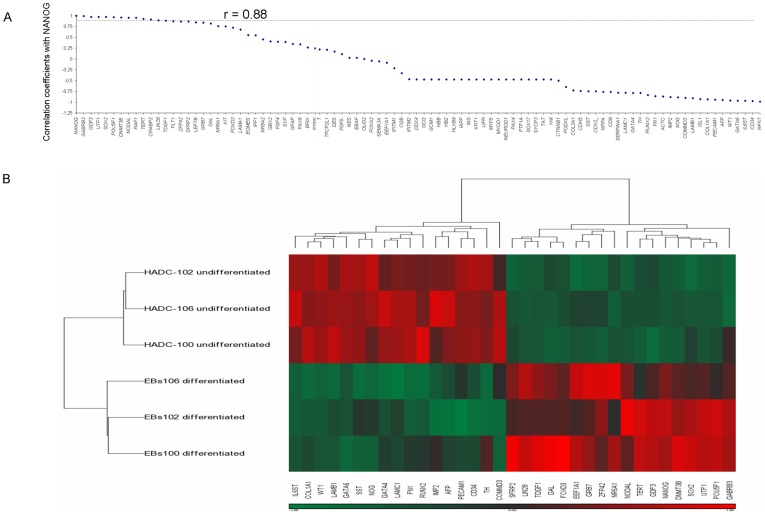
Gene expression analysis of undifferentiated and differentiated progeny of the three hESC lines. Pairwise correlation of NANOG expression with that of all tested genes of the panel of HADC-100, HADC-102 and HADC-106 hESCs is presented in (A). Gene expression is presented by the DeltaCt value of each gene, normalized against the average Ct of three endogenous control genes (Table S9). The Pearson correlation coefficient was calculated from the combined data of DeltaCt values of undifferentiated and differentiated hESCs of the three lines. The results were plotted in descending order of the correlation coefficient values. Clustering analysis of genes that were differentially expressed in the three lines in the undifferentiated and differentiated state is presented in (B). An unpaired t-test for differential gene expression between undifferentiated and differentiated samples yielded 35 genes with a P- value <0.05. Two-way hierarchical clustering with Euclidean distance was performed on the 35 genes and six samples. Green colors in the heat map represent low DeltaCt or high expression. Red colors represent high DeltaCt or low expression.

RNA was prepared from undifferentiated hESCs colonies and EBs from each one of the lines. Gene expression was analyzed by the same Human Stem Cell Panel array that was previously used by the International Stem Cell Initiative (ISCI [36]) to analyze 59 lines. Gene expression of each gene is reported as its DeltaCt value normalized against the average Ct of three selected endogenous control genes (18 S, beta-actin and GAPD-H; Table S10). Using the combined data of undifferentiated and differentiated samples, we tested the pairwise Pearson correlation of expression of the genes to the expression of NANOG (Figure 5A). The expression of genes that are associated with pluripotency including GABRB3, GDF3, SOX2, POU5F1, DNMT3B, TERT, LIN28 and TDGF1 [36] was highly (r>0.88) correlated with the expression of NANOG. Five of these genes (GABRB3, GDF3, POU5F1, DNMT3B and TDGF1) were also found to be highly correlated (r>0.75) with the expression of NANOG in the ISCI study [36]. Genes that are known to be commonly associated with differentiation such as CDX2, GATA6, AFP, CD34, PECAM1, TH and ISL1 showed negative correlation (r<−0.75) with NANOG expression.

We performed two-way hierarchical clustering analysis of the three clinical grade hESC lines with respect to gene expression. The genes that are presented in Figure 5B are those that significantly differed in their expression between undifferentiated and differentiated samples (unpaired t-test; P<0.05). Clustering results show two distinct sample clusters: one includes the undifferentiated cells of the three lines and the other the differentiated cells. The data also shows two clear clusters of genes expressed by undifferentiated and differentiated cells in each one of the samples. The genes NANOG, GABRB3, GDF3, POU5F1, DNMT3B and TDGF1, that were typically upregulated in the pluripotent state in the ISCI study, were also highly expressed by the three clinical-grade undifferentiated cell line samples and were down regulated upon differentiation. Genes that are expressed by differentiated cells (such as AFP, GATA4, GATA6, FN1, COL1A1) [36] were highly expressed in the EBs in all three lines and were down regulated in the undifferentiated hESCs.

## Discussion

In this study we derived for the first time xeno-free and GMP-grade human feeders and ESC lines adhering to regulatory guidelines. We developed a unique animal reagent-free culture system for the derivation of the new hESC lines as well as the regulatory platform for donor selection and testing, which can serve for the establishment of additional clinical-grade lines. The hESC lines developed in this study may be utilized for the development of differentiated cells for transplantation therapy.

The ethical considerations that guided the development of the hESCs strictly followed Israeli law [37], [38] and adopted the recommendations of the Bioethics Advisory Committee of the Israel National Academy of Sciences and Humanities [39]. They were in line with the ethical guidelines of the ISSCR (www.isscr.org), the US’s National Academy of Sciences (www.nasonline.org) and the National Institute of Health (NIH: http://nih.gov). The three lines were approved for inclusion in the NIH Human Embryonic Stem Cell Registry (http://grants.nih.gov/stem_cells/registry/current.htm), and therefore can be utilized in federally funded research in the US. They may be used worldwide subject to specific national regulations. The ethical considerations that guided the derivation of hESC lines in this report, and the draft informed consent for the recruitment of embryo donors, may be valuable to other research groups deriving new clinical-grade hESC lines.

For the establishment of a hESC culture system that will be compliant for future clinical use, we established a material qualification process. We verified that the materials were primarily of GMP-grade, and were xeno-free. Vendors’ adherence to GMP was ascertained by questionnaire and some materials were quality tested. Qualified materials were then introduced into the development process of our protocols in the research environment and tested for their suitability. The vendor quality approval, material quality testing and reagents’ qualification in the process guaranteed that reproducible and safe hESC lines were derived.

The derivation protocols, which incorporated these materials, were initially used to develop, for the first time, xeno-free, GMP-grade human feeders from three sources; umbilical cord, foreskin and fetal tissue. We showed that the feeders from the three sources were similarly potent in supporting the undifferentiated propagation of hESCs, and hence elected to work with umbilical cord due to its many advantages.

Cord may be obtained during elective cesarean sections as a completely sterile tissue compared to foreskin which can only be disinfected before sampling. Cord blood can be easily harvested in sufficient quantities for donor safety testing, as compared to the ethical constraints and technical difficulties in obtaining sufficient blood sampling from infants undergoing circumcisions. Additionally, unlike with aborted fetal tissues, there is no moral debate regarding the use of umbilical cord tissue for research purposes.

At an early stage of the project, we identified a GMP-grade xeno-free media formulation that supported self-renewal of hESCs at an efficiency not significantly different compared to the standard KO research grade medium. At present, additional GMP-grade and animal-free media designed for the propagation of hESCs are available, though their capability to support derivation and long term propagation of genetically stable clinical-grade pluripotent hESC lines has yet to be demonstrated and compared with the culture system reported here. At present, guidelines for screening of donors of embryos that will be used for deriving hESC for therapeutic applications have not been established by regulatory bodies. Hence, we adopted the FDA’s HCT/P [34] regulations for screening of organ, cell and gamete donors as well as the EC’s blood banking and organ donation criteria for screening of donors. Based on these criteria, we developed a detailed series of questionnaires (CRF) of which we here include samples for potential use by future developers of clinical grade lines. It should be noted that it is questionable whether the intensive interview and history-taking process have a significant contribution to safety analysis of donated biologics. Donors are hesitant to reveal private intimate details of past relationships [40],[41]. We therefore complemented the questionnaire with testing the hESC lines and samples of donor serum and reproductive tract for the absence of adventitious viruses. In addition, a reported medical history in an interview may be incomplete because of the donors’ partial understanding of their medical conditions. Therefore, according to FDA guidelines, we reviewed the medical charts of the embryo donors and confirmed their medical histories.

We utilized a laser-based method to assist hatching and to dissect the ICMs to avoid the use of animal derived products. The efficiency of ICM isolation from blastocysts with well defined ICMs was 85% (23 ICMs from 27 blastocysts) and the efficiency of hESC line derivation was 11% (3 lines from 27 blastocysts). This efficiency is lower than the reported derivation efficiency of hESC lines under research-grade conditions (13%–52%) [42],[43] including in our hands while using the laser system (30%) [10]. A low efficiency (4.75%) of hESC derivation under conditions necessary to derive hESC lines of therapeutic grade was also reported by others [44]. It appears that translation to xeno-free culture system and GMP conditions is associated with a reduced derivation success.

We established a modified banking system which included an early stock, 1° and 2° CBs to address potential pitfalls related to the properties of hESCs. The stocks were developed as reservoirs of early passage cells for identity determination, and as backups for the development of additional banks in various circumstances such as the unfortunate loss of the hESCs to differentiation or contamination.

According to the widely-accepted two-tiered cell banking system, a master cell bank (MCB) is initially established, and working cell banks (WCBs) are further developed from thawed vials of MCB. hESCs poorly survive cryopreservation [45] and the stresses that are imposed on hESCs during cryopreservation and thawing, may potentially select for genetically abnormal cells which may survive unfavorable culture conditions [45],[46]. Therefore, to limit the number of freezing and thawing steps, we modified the typical two-tiered banking approach and continuously expanded the hESCs from the initial outgrowths until the freezing of the 2° CBs. During this expansion, hESCs were sampled to generate early stocks and subsequently 1° and 2° CBs. The complete records of all stocks and banks, including their origins, details of their derivation, propagation and cryopreservation locations were documented to maintain the histories of the lines.

Characterization of the lines was initiated by determining the identity of each line at the early stock phase by DNA fingerprinting and HLA analysis. It has been previously shown that mix-ups of lines [47],[48],[49] and cross-cell contamination [50],[51], both leading to false identities, are common occurrences in cell banking. Therefore, the unique genetic signature determined at the stock stage of each hESC line was confirmed by STR and HLA analysis at the 1° and 2° Cell Bank stages. The strict determination of the lines’ identity is important for the essential confirmation of the identity and origin of the differentiated cells that will be developed from them for transplantation.

The specifications for the characterization of the hESC lines were developed on the basis of ICH guidelines (Q5D) [32], recommendations of the International Stem Cell Banking Initiative [52], and regulatory experts [5]. Careful development of a reproducible and validated derivation process, utilization of documented protocols and qualified, verified raw materials enabled the derivation of hESC banks that adhered to the characterization specifications (batch release criteria).

We performed G-banded karyotype analysis to determine the absence of chromosomal abnormalities. The International Stem Cell Banking Initiative [52] recommends analysis of 30 metaphase spreads for the characterization of research-grade lines. Analysis of 30 spreads was also reported for the characterization of hESC lines derived under cleanroom conditions [24]. Fifty spreads were analyzed in this report as it has been shown that the probability of detecting mosaicism patterns increases when the numbers of tested metaphase spreads increase [53]. We thus provide a higher confidence level of the exclusion of mosaicism than previously reported.

Internationally accepted criteria for the characterization of hESC lines were established on the basis of the characterization of 59 hESC lines from multiple laboratories worldwide by the ISCI study [36]. To adhere to these standards, which include gene expression profiling, and to allow the inclusion of the new lines in the ISCI Registry (www.stemcellforum.org), we analyzed gene expression utilizing the same stem cell array that was used by ISCI. The gene expression profile of the three clinical-grade hESC lines was comparable to the profile of the research-grade lines that were characterized in the ISCI study [36]. The genes that were suggested by the ISCI study to be common to undifferentiated hESCs were expressed by the pluripotent hESC of the three lines and were downregulated upon differentiation.

The first clinical trials with hESC-derived progeny have recently commenced, stressing the need for developing clinical-grade lines that can be utilized to translate hESC research into the clinic. In this paper, we present ethical, scientific and regulatory platforms for the development of xeno-free and GMP-grade hESC lines, which may serve the research community for the derivation of additional clinical-grade lines. The animal-free, regulatory-compliant hESC lines derived here will be valuable for developing regenerative therapies.

## Materials and Methods

### Ethics Statement

The study was approved by the Hadassah Medical Center IRB and the Israeli Ministry of Health’s National Ethics Committee for Genetic Research in Humans (13.01.2004). A copy of our informed consent document is included as File S1 in our manuscript. In all cases, written informed consent was obtained by our donors prior to their acceptance into our study, and the clinical investigation was conducted according to the GCPs and Helsinki guidelines.

### Fibroblast Tissue Procurement

Human fibroblasts were obtained from foreskin, umbilical cord, or fetal tissue (10–12 weeks of gestation). Potential donors were approached only after they had decided to undergo each of the surgical procedures (infant surgical circumcision, cesarean section, termination of pregnancy). Tissue donors were screened for eligibility, according to FDA’s Donor Eligibility Regulations 2004 [34]. Their medical histories were examined, blood and reproductive systems (in the case of umbilical cord, and fetal tissues) were sampled for infectious and sexually-transmitted disease testing (Table S5), and the donors were interviewed to complete extensive lifestyle questionnaires to minimize chances of transmission of contaminants to eventual cell recipients. Blood samples were obtained from circumcised infant and his parents, fetal tissue donors, the cord and both parents. They were sent for infectious disease testing and serum was archived at Hadassah. All infectious disease screening was performed by FDA-approved tests at American Medical Laboratories (Herzliya, Israel).

### Derivation and Cryopreservation of Human Fibroblast Feeder Cells

#### Preparation of fibroblasts from umbilical cord

During cesarean sections, after detachment of the placenta, a 10 cm segment of cord was sampled and transferred under sterile conditions into PBS within a prelabeled vessel, which was sealed and transferred to the GMP facility. The cord tissues were minced with 2 sterile surgical blades (#22) or a surgical blade and forceps. A T75 flask (Falcon, Becton Dickinson, Franklin Lakes, NJ, USA) was held vertically and small pieces of tissue were transferred with a pipette to the wall of the flask. Fibroblast Growth Medium (FGM) comprised of DMEM, L-Glutamine 200 mM, (both from Hyclone, Thermo Scientific, Logan, Utah), 20% Human Serum of blood type AB (Male Only, Lonza Walkersville, MD, USA) was then added to the bottom of the flask without disturbing the tissues. Flasks were incubated vertically at 37°C, 5% CO_2_. On the following day, the flasks were gently tilted to moisten the tissue and remained in a vertical position. On the following day, the flasks were placed in a horizontal position for further culture for 17–22 days until the outgrowths of cells formed a confluent monolayer. The cells were split 1∶3 twice a week using TrypLE Select recombinant trypsin (Invitrogen, Grand Island, NY, USA). At passage 3 or 4, when the batches contained 195–243 million cells, master cell banks (MCB) were frozen. The fibroblasts were removed from the flasks with TrypLE Select, resuspended in FGM, centrifuged at 900 RPM for 4 minutes, resuspended and pooled within FGM, and then counted by Trypan Blue exclusion staining to confirm viability in >80% of cells. Samples from the pool were sent to a local accredited testing lab (Hy Labs, Nes Tziona, Israel) for sterility, mycoplasma (culture), and LAL (Limulus Amebocyte Lysate) testing. After centrifugation as above, the cells were frozen (2 million cells per cryovial (NUNC, Roskilde, Denmark)) within 1 ml of 90% Human Serum AB (Lonza) and 10% DMSO (CryoSure, WAK-DMSO-10, WAK-CHEMIE Medical GmbH, Steinbach/Ts. Germany), by a standard slow cooling procedure, and stored in the vapor phase of liquid nitrogen tanks (Custom Biogenic Systems V1500B, Romeo, Michigan USA,).

#### Preparation of fibroblasts from foreskin

Foreskins were obtained from infants up to one year of age from surgical circumcisions. The foreskins were disinfected by Chlorhexidine (dissolved in alcohol) prior to circumcision. The foreskins were transferred in PBS to the GMP-facility as described above. The sides of the tissues were cut with sterile scissors so that the tissues became flat. The top layers of the inner and outer sides were scraped with #22 surgical blades. The tissues were minced; derivation and cryopreservation of fibroblasts MCB proceeded as above.

#### Preparation of fibroblasts from fetal tissue

Tissues from aborted human fetuses were obtained from the operating room and transferred to the GMP-facility as above. The tissues were mechanically minced, and 2 ml of TrypLE Select (Invitrogen) were added for 10 minutes at 37°C. Fibroblast Growth Medium (FGM) was added followed by pipetting and centrifugation at 1000 rpm for 3 minutes. The cells were resuspended and 4–8×10^6^ cells in 10 ml Fibroblast Growth Medium were seeded in a T25 flask (Falcon). The cells were incubated in 37°C at 5% CO_2_, and when confluent, split, expanded and frozen as above.

#### Development of irradiated working cell banks (WCB) of cord feeders

Vials of feeder MCB were thawed and expanded as above up to passages 7–8 when the batches contained 350–475 million cells. The fibroblasts were pooled, and were sampled for sterility, mycoplasma and LAL testing as above. The fibroblasts were then irradiated within 30 ml medium (1–1.6×10^6^/ml) in 50 ml tubes at 3500 rads (Gammacell, 220 Exel, MDS Nordion), repooled, recounted by Trypan Blue exclusion staining and cryopreserved using the methods described for the MCBs.

### Characterization of Feeders

#### Doubling time of feeders

5×10^4^ fibroblasts were seeded per well of 12-well tissue culture plates on day 0. On four subsequent days, the number of fibroblasts in three wells was counted, taking the average of the three. Cell counts were then plotted versus the time of cells harvest using Microsoft Excel software. The software was used to plot the optimized exponential trend line and its equation. The equation was used to calculate the time interval for population doubling.

#### BrdU and KI-67 staining of fibroblasts

25,000–30,000 irradiated human fibroblasts were seeded on 0.1% gelatin-coated coverslips, incubated for 7 days at 37°C in FGM. Mitomycin-treated (Bedford Labs, Bedford, OH, USA) cells were cultured as above as negative controls. For positive controls 30,000 non-irradiated human fibroblasts were seeded and cultured for two days.

For BrdU staining the samples were incubated with BrdU (50 µM final concentration, Sigma, St. Louis, MO, USA) for 1 hour at 37°C and the cells were fixed with 4% PFA. The cells were treated with HCl (1 N and 2 N) and Sodium Borate buffer (0.1 M). The plates were incubated overnight with anti-BrdU (1∶50, Dako, Glostrup, Denmark) and the secondary antibody was goat anti-mouse Ig conjugated FITC (1∶50, Dako). For KI-67 staining cells were fixed with 0.5–1 ml 4% PFA for 20 min at RT and incubated with anti KI-67 (1∶100, Rabbit Polyclonal Antibody KI-67 Antigen, Novo Castra Laboratories, Newcastle-upon-Tyne, UK) for 30–60 minutes at RT. The secondary antibody used was Polyclonal Swine Anti-Rabbit Ig conjugated to FITC (1∶10, Dako).

Slides were visualized after mounting and nuclear counterstaining (Vectashield, Vector Laboratories, Burlingame, Ca, which includes 4′, 6-diamidino-2-phenylindole (DAPI)) with a Nikon E600 fluorescent microscope.

#### Immunostaining of feeders

30,000 fibroblasts were seeded onto laminin-coated coverslips and incubated in FGM for an hour, fixed with 4% PFA for 20 min and permeabilized with Triton X100 (0.2%, Sigma), normal goat serum (5%, Biological Industries, Kibbutz Beit Haemek, Israel), Na Azide (0.1%, Sigma) in PBS for 20 min. The cells were incubated for 20–30 min at RT with anti-Vimentin (1∶100, Dako) followed by incubation with FITC-labeled goat anti-mouse Ig (1∶50, Dako) and mounting as above.

#### FACS analysis of fibroblasts

Fibroblasts were dissociated using TrypLE Select (Invitrogen) followed by gentle trituration to a single-cell suspension, centrifuged and resuspended in FACS buffer (PBS supplemented with 0.1% Na Azide and 1% BSA). The cells were incubated with anti-CD44-FITC (1∶10, IQ Products, Groningen, the Netherlands), anti-human fibroblasts (1∶100, Acris, San Diego, CA, USA) and with the respective isotype control antibodies for 30 min at 4°C. Following washing, primary antibodies were detected by incubating with FITC-labeled goat anti-mouse Ig (1∶100, Dako) for 30 min at 4°C. Following washing the cells were resuspended in FACS buffer supplemented with PI (1∶500, Sigma 1 mg/ml). FACS analysis was performed using the FACSCalibur system (Becton Dickinson).

#### Karyotyping of feeders

Fibroblasts were incubated for 40 minutes with Colcimide (100 ng/ml, Beit Haemek), washed with PBS, dissociated with TrypLE Select (Invitrogen) for 4 minutes at 37°C, centrifuged at 1000 rpm for 5 minutes, resuspended in 1 ml of growth medium into which 5 ml of warm KCl and Sodium citrate hypotonic solution (Sigma) were added dropwise, incubated for 30 min at 37°C, followed by fixation at RT with 3∶1 methanol/acetic acid for 5 min. Centrifugation and fixation of the cells were repeated twice. The karyotype of 30 metaphases was analyzed using the G-banding method. Full analysis was performed on 20 metaphases spreads, chromosome counting on an additional 10 spreads, and 2 cells were photographed.

#### STR analysis for feeders

Genomic DNA was extracted using QIAamp DNA Mini Kit (QIAGEN GmbH, Hilden, Germany)**.** STR (Short Tandem Repeats) profiles of 16 sites (D13S317, D7S820, D16S539, vWA, TH01, TPOX, CSF1PO, D8S1179, D21811, D19S433, D18851, D3S1358, D2S1338, D5S818, FGA and amelogenin (for gender determination)) were obtained using the identifier kit (PE Applied Biosystems Corona, CA, USA).

The STR analysis was performed in the Hadassah Tissue Typing Unit.

#### hESC doubling time when cultured on various feeders

Mitomycin-treated (Bedford Labs) 10^5^ MCB fibroblasts or irradiated WCB fibroblasts were seeded into each of 16 wells of 12-well tissue culture plates coated with gelatin. After 24–96 hours, hESCs were seeded (20,000 hESCs per well) into 12 wells, leaving four wells as controls. At 4 time points within a week, the cells were harvested with Trypsin-EDTA (Invitrogen), the average number of hESCs in three wells was determined by subtracting out the number of fibroblasts counted from the control wells from the total number of cells. Cell counts were then plotted versus the time of cells harvest and the doubling time was calculated using Microsoft Excel software as described above.

### Development of hESC Lines

#### Embryo culture and laser-assisted ICM isolation

Thawing of embryos was performed using Embryo Thaw Media (Irvine Scientific, Santa Ana, CA, USA). After thawing, the embryos were cultured until day 3 of development in Quinn’s Advantage® Cleavage Medium and further to the blastocyst stage in Quinn’s Advantage® Blastocyst Medium, both supplemented with 10% Quinn’s Advantage® Serum Protein Supplement (all from In-Vitro Fertilization Inc., Trumbull, CT, USA). Blastocyst morphology was scored according to Stephenson, Braude and Ilic [54]. For assisted hatching, zona pellucidae was perforated on day 3–4 by laser pulses (200 mW×0.3 ms) from a non-contact Zilos-tkTM infrared laser system (Hamilton Thorne Biosciences, Beverly, MA, USA). Perforation diameter was 30–40 um. Assisted hatching and ICM isolation was performed according to our previously published methods [10].

In cases of collapse of blastocysts aged 5–6 days during assisted hatching or ICM dissection, they were incubated overnight. Another attempt of ICM isolation was then performed. In cases of collapse of blastocysts aged 7 days, or in cases when the ICM was non-detectable, whole blastocysts were plated on feeders for further derivation of hESCs.

#### Research-grade Knockout medium composition

Knockout Dulbecco’s modified Eagle’s medium (DMEM) supplemented with 15% Knock-Out SR, 2 mM L-glutamine, 1% nonessential amino acids, 50 U/ml penicillin, 50 mg/ml streptomycin (all from Invitrogen), supplemented with 4 ng/ml basic fibroblast growth factor (bFGF) (PeproTech Inc., Rocky Hill, NJ, USA).

#### Xeno-free GMP-grade hESCs culture system

For derivation of hESCs, 1.5×10^5^ irradiated thawed WCB feeder cells were plated in center-well tissue culture dishes (Falcon) pre-coated with 0.1% 100 kDa rh-Gelatin (Fibrogen, San Francisco, CA, USA) in Fibroblast Growth Medium. For weekly routine hESC passaging, only 10^5^ irradiated WCB feeder cells were plated. After 24 hours, the medium was replaced by hESC Medium comprised of Cellgro-SCGM (CellGenix, Freiburg, Germany) supplemented with 1% Human Serum Albumin (Irvine Scientific, Santa Ana, CA, USA), 2 mM L-glutamine and 1% nonessential amino acids (both from Hyclone), and with 8 ng/ml bFGF (R&D Systems, Minneapolis, MN, USA). The hESC Medium was refreshed daily. Feeders and hESCs were cultured at 37°C in 5% CO_2_.

Routine passaging of putative hESCs was performed by mechanical dissection (Stem Cell Cutting Tool, Vitrolife, Sweden) of the colonies into small clusters and plating on fresh feeders.

### Cryopreservation of hESCs

#### Vitrification of hESCs

hESC clusters were vitrified using Cryotips (FDA-approved as medical devices, Irvine Scientific) and Irvine Scientific Vitrification Solutions (Vit-Kit Freeze Solutions, Irvine Scientific) according to the manufacturer’s instructions with modification of incubation times adapted from Reubinoff et al., 2001 [45]. 5–6 small colony fragments were transferred to the Equilibration Solution (ES) for 5 minutes, then to the Vitrification Solution (VS) twice for 10 seconds and then to a final drop of VS from which they were aspirated into the straw. The straws were heat sealed and plunged into liquid nitrogen where they were stored. Thawing was performed for 3 seconds in warmed sterile water (37°C) followed by removal of hESC clusters from straws and serial immersion in Irvine Scientific’s Vit Kit Thawing Solutions (Irvine Scientific) according to the manufacturer’s instructions.

#### Slow-cooling of hESCs

8–10 hESC colony fragments were transferred into 2.0 ml cryovials (Nunc) containing 1.0 ml CryoStor 10 (Biolife Solutions, Bothell, WA USA) freezing solution or 90% human serum (Lonza) and 10% DMSO (WAK-Chemie). The vials were slow cooled in a freezing container (Mr. Frosty, Nunc-Nalgene) to −80°C and then were stored in the vapor phase of liquid nitrogen tanks (Custom Biogenic Systems). Thawing was performed by warming at 37°C in a waterbath, transferring into hESC culture medium, centrifuging at 1000 RPM for 3 minutes, and resuspending in hESC culture medium.

### Characterization of hESCs Lines

#### Analysis of HLA and STR fingerprinting

In order to eliminate contamination by feeders, hESCs were cultured for a minimum of three passages in a feeder-free culture system as previously described [55] with slight modifications. STR analysis was performed as described above for feeders. For HLA profiling, the DNA was extracted using the MagNA Pure LC machine (Roche Applies Sciences, Indianapolis, IN, USA). HLA- A, B, DRB1, DQB1 loci at low resolution were typed using the Lipa HLA kit (Murex Immunogenetics, Ghent, Belgium). High resolution typing of the HLA- DRB1 and DQB1 alleles used the PCR- Sequence Specific Primers method (PCR-SSP,Olerup kit, Olerup, Stockholm, Sweden), according to the manufacturer’s instructions. The HLA and STR analyses were done in the Hadassah Tissue Typing Unit.

#### Alkaline phosphatase activity detection and karyotyping

Vector Red Alkaline Phosphatase substrate kit I (Vector Laboratories Inc., Burlingame, CA, US), Alkaline Phosphatase Staining Kit II (Stemgent, Cambridge, MA, USA), or AP Substrate Kit (Sigma) were used for detection of alkaline phosphatase activity within intact colonies on feeders according to the manufacturer’s instructions.

For karyotype analysis, hESC colonies that were expanded by mechanical passaging on feeders, were incubated for 1 hour with 100 ng/ml Colcemid and then dissociated with trypsin (both from Biological Industries, Beit Haemek, Israel). Incubation in hypotonic solution and fixation was as described for the feeders. For the primary reference banks, full analysis was performed on 30 metaphases spreads (20 for stocks and 2° cell bank), chromosome counting on an additional 20 spreads (10 for stocks and 2° cell bank), and 2 cells were photographed for each stock/bank.

#### Immunostaining

hESCs colonies that were cultured on feeder layer for 4–6 days were fixed with 4% paraformaldehyde for 20 min at RT. For immunostaining of intracellular markers, cell membranes were permeabilized with 0.2% Triton X100 (Sigma), 5% normal goat serum (Beit Haemek) in PBS for 20 min. The cells were incubated for 20–30 min at RT with the following primary antibodies: anti**-**human Oct-3/4 antibody (1∶50; Santa Cruz Biotechnology Inc., USA), mouse monoclonal anti-human TRA-1-60 (1∶50), TRA-1-81 (1∶50) and SSEA-4 (1∶50) antibodies (all from Chemicon International, Temecula, CA, USA) followed by [fluorescein isothiocyanate (FITC)]-labeled goat anti-mouse immunoglobulin (1∶50, Dako). Rat monoclonal anti-human SSEA-3 (1∶50, Chemicon), followed by mouse anti-rat IgM conjugated to R-PE, (1∶20; Southern Biotechnology Associates Birmingham, Al, USA) or Alexa Fluor- 488 labeled goat anti-rat IgM (1∶100, Invitrogen). Rabbit Polyclonal Anti KI-67 (1∶100, Novo Castra Laboratories), followed by FITC-conjugated Polyclonal Swine Anti-Rabbit Immunoglobulins (1∶10, Dako) was used. Mounting medium containing DAPI (Vector) was used for nuclei counterstaining and the specimens were visualized with a Nikon E600 fluorescent microscope.

#### FACS analysis

hESC colonies were dissociated with 0.05% EDTA solution followed by gentle trituration to a single-cell suspension. The hESCs were centrifuged and re-suspended in FACS buffer. The cells were incubated with anti-TRA-1-60 (1∶100), anti-TRA-1-81 (1∶100), anti SSEA-4 (1∶200), anti SSEA-1 (1∶300; all from Chemicon) and anti SSEA-3 (1∶100, Millipore) for 30 min at 4°C. As control, hESCs were stained with respective isotype control antibodies (all from eBiosciences). Following washing, primary antibodies were detected by incubating with FITC-labeled goat anti-mouse Ig (1∶100, Dako) and goat anti-rat Ig (1∶100, Invitrogen) for 30 min at 4°C. Following washing, the cells were resuspended in FACS buffer supplemented with PI (1∶500 of 1 mg/ml solution, Sigma). FACS analysis was performed using the FACSCalibur system (Becton Dickinson).

#### hESC doubling time

The doubling time was determined as described above for the characterization of feeders with slight modifications. Irradiated cord feeder cells were plated in 16 center well tissue culture dishes (10^5^ cells/dish). Clusters of hESCs were removed mechanically and equal numbers of clusters (numbers ranged from 15–60) were plated per experiment in each of 12 center well tissue culture dishes (Falcon) leaving four dishes as controls. HESCs together with feeder cells (in triplicates) were harvested using Trypsin-EDTA (Invitrogen) and counted at four time points. The number of cord feeder cells harvested at the same time points from the control dishes was subtracted from the total number of cells. The doubling time was calculated as above.

#### Analysis of pluripotency in vitro

Colonies of undifferentiated hESCs were mechanically removed from the feeders and cultured in suspension as embryoid bodies (EBs) or neurospheres. For the development of EBs, the hESC free-floating clusters were cultured 3 weeks in DMEM supplemented with 15–20% FCS, 2 mM L-glutamine, 1% nonessential amino acids, 50 U/ml penicillin, 50 µg/ml streptomycin, 0.1 mM beta-mercaptoethanol (all from Invitrogen). Neurospheres were developed as previously described [56]. Briefly, the hESC clusters were cultured 3 weeks in DMEM/F12 (1∶1) supplemented with B-27 (1∶50), 2 mM L-Glutamine, 50 U/ml penicillin, 50 µg/ml streptomycin (all from Invitrogen), 20 ng/ml bFGF and 500 ng/ml rm-Noggin (both from Peprotech, London, UK).

After the 3 weeks of suspension culture, the EBs and the neurospheres were partially dissociated by mild trypsin digestion and plated on glass coverslips pretreated with 10 µg/ml poly-d-lysine (Sigma) and 4 µg/ml laminin (Sigma), and cultured for an additional 5–7 days in the respective culture media as above in the absence of noggin and bFGF. They were fixed with 4% paraformaldehyde and then stained with mouse monoclonal anti-beta-tubulin isotype III (1∶2000; Sigma), mouse monoclonal anti-human desmin (1∶50; Dako), or mouse monoclonal anti-human muscle actin (1∶50; Dako), and mouse monoclonal anti-human sox-17 (1∶50; R&D).

Polyclonal goat anti-mouse immunoglobulins conjugated to FITC (1∶50, Dako) or to Cy3 (1∶1000, Jackson Laboratories) were used to detect the primary antibodies. Mounting and visualization was performed as above.

#### Analysis of pluripotency in vivo

Subject to an approval of the Institutional Ethical Committee for Care and Use of Animals, 4–5 week old NOD SCID mice (Harlan, Jerusalem, Israel) were subcutaneously inoculated utilizing a 25 G needle with 100–200 small clusters of approximately 500 cells of undifferentiated hESCs in each cluster, which had been mechanically removed from the feeders. The hESCs were injected with 1×10^6^ irradiated cord fibroblasts cells, in a total volume of 60 ul, together with an equal volume of Matrigel (Basement membrane matrix, Becton Dickenson Biosciences). Maximum teratoma size permitted was 15 mm. 8–12 weeks later, the resulting tumors were removed, fixed in neutral buffered 4% formalin, embedded in paraffin, and examined histologically after hematoxylin and eosin staining.

#### Gene expression

*RNA preparation:* Total RNA was prepared from Trizol (Invitrogen) lysates of hESCs colonies, 5–6 days after passage, and embryoid bodies (EBs as above), after 3–4 weeks of differentiation. DNA was removed with DNA-free (Ambion, Austin, TX, USA). cDNA synthesis was carried out using Moloney murine leukemia virus reverse transcriptase (MMLV RT, Promega, Madison, WI, USA) and random hexamers (Promega), according to the manufacturer’s instructions (Promega).

*Real Time PCR* ***:*** For quantitative gene expression analysis Low density TaqMan array: Human stem Cell Panel, Applied Biosystems Part Number 4385344 was used with Taqman Gene Expression Master Mix (Applied Biosystems, Foster City, CA, USA). ABI Prism 7900 HT Sequence Detection System (Applied Biosystems) was used.

Gene expression was analyzed using the DataAssist Software v2.0 (Applied Biosystems).

### Safety Testing

Feeders and hESCs were outsourced for testing at Bioreliance (Glasgow, UK) and the Hadassah Virology and Hepatology Laboratories.

### Statistical Analysis

Data are presented as mean ±S.E.M. The significance of multiple comparisons was determined using one way analysis of variance (ANOVA) with Tukey-Kramer multiple comparisons test.

## Supporting Information

Figure S1 **Characterization of the primary cell bank of clinical grade HADC102 hESCs.** The hESCs colonies were comprised of small tightly packed cells with a high nuclear to cytoplasmic ratio. Clear distinguishable borders were observed between the colonies and cord feeder cells, (A; phase-contrast image). The cells expressed alkaline phosphatase (B; AP, fluorescence image). Indirect immunofluorescence staining showed that the hESCs were immunoreactive with anti-Oct-4 (C; D, nuclear 4',6-Diamidino-2-phenylindole (DAPI) counter staining), TRA-1-60 (E), TRA-1-81 (F), SSEA-3 (G), and SSEA-4 (H). FACS analysis showed that the majority of cells expressed markers of pluripotency TRA-1-60 (I), TRA-1-81 (J) and SSEA-3 (K) (Data from a representative experiment). The hESCs had normal karyotype (46, XY; L) and could differentiate *in vitro in vivo* into cells representing the three embryonic germ layers (M-R). Immunofluorescence staining showing *in vitro* differentiated cells expressing beta-tubulin III (ectoderm, M), muscle actin (mesoderm, N) and sox-17 (endoderm, O). Hematoxylin-eosin stained histological sections of teratoma tumors showing neural rosettes (ectoderm, P), cartilage (mesoderm, Q) and columnar glandular epithelium with goblet cells (endoderm, R). Scale bar represent 20 um for (Q and R), 50 um for (B, C, D, N and O), 100 um for (A, M and P) and 200 um for (E -H).
(TIF)

Figure S2 **Characterization of the primary cell bank of clinical grade HADC106 hESCs** The hESCs colonies were comprised of small tightly packed cells with a high nuclear to cytoplasmic ratio. Clear distinguishable borders were observed between the colonies and cord feeder cells, (A; phase-contrast image). The cells expressed alkaline phosphatase (B; AP, fluorescence image). Indirect immunofluorescence staining showed that the hESCs were immunoreactive with anti-Oct-4 (C; D, nuclear 4',6-Diamidino-2-phenylindole (DAPI) counter staining), TRA-1-60 (E), TRA-1-81 (F), SSEA-3 (G), and SSEA-4 (H). FACS analysis showed that the majority of cells expressed markers of pluripotency TRA-1-60 (I), TRA-1-81 (J) and SSEA-3 (K) (Data from a representative experiment). The hESCs had normal karyotype (46, XY; L) and could differentiate *in vitro in vivo* into cells representing the three embryonic germ layers (M–R). Immunofluorescence staining showing *in vitro* differentiated cells expressing beta-tubulin III (ectoderm, M), muscle actin (mesoderm, N) and sox-17 (endoderm, O). Hematoxylin-eosin stained histological sections of teratoma tumors showing neural rosettes (ectoderm, P), cartilage (mesoderm, Q) and villi structures with columnar glandular epithelium and goblet cells (endoderm, R). Scale bar represent 50 um for (N and O), 100 um for (A, B, C, D, G, M and P) and 200 um for (E, F, H, Q, R).
(TIF)

Figure S3 **Characterization of the secondary cell bank of clinical grade HADC100 hESCs.** The hESCs colonies were comprised of small tightly packed cells with a high nuclear to cytoplasmic ratio. Clear distinguishable borders were observed between the colonies and cord feeder cells, (A; phase-contrast image). The cells expressed alkaline phosphatase (B; AP, fluorescence image). Indirect immunofluorescence staining showed that the hESCs were immunoreactive with anti-Oct-4 (C; D, nuclear 4',6-Diamidino-2-phenylindole (DAPI) counter staining), TRA-1-60 (E), TRA-1-81 (F), SSEA-3 (G), and SSEA-4 (H). FACS analysis showed that the majority of cells expressed markers of pluripotency TRA-1-60 (I), TRA-1-81 (J) and SSEA-3 (K) (Data from a representative experiment). The hESCs had normal karyotype (46, XY; L) and could differentiate *in vitro* and *in vivo* into cells representing the three embryonic germ layers (M–R). Immunofluorescence staining showing *in vitro* differentiated cells expressing β-tubulin III (ectoderm, M), muscle actin (mesoderm, N) and sox-17 (endoderm, O). Hematoxylin-eosin stained histological sections of teratoma tumors showing neural rosettes (ectoderm, P), cartilage (mesoderm, Q) and columnar glandular epithelium with goblet cells (endoderm, R). Scale bar represent 50 um for (A, B, N, O, P, Q and R), 100 um for (C, D and M) and 200 um for (E–H).
(TIF)

Figure S4 **The majority of hESCs are in an active cell cycle.** Indirect immunofluorescence analysis shows that the majority of hESCs in each of the cell banks are immunoreactive with anti-KI67. (Green: Quantitative analysis of the percentage of KI67+ cells within 200 cells in each of three random fields appears in brackets). Low magnification images and corresponding nuclei DAPI counterstaining are presented in the left two columns, while higher magnification in the right two columns.
(TIF)

Table S1 Feeder and hESC Materials.
(DOC)

Table S2 Feeder Characterization MCB.
(DOC)

Table S3 Feeder Adventitious Virus Testing Results.
(DOC)

Table S4 Feeder Characterization WCB.
(DOC)

Table S5 Donor Testing Results.
(DOC)

Table S6 List of Forms.
(DOC)

Table S7 Blastocyst Table.
(DOC)

Table S8 hESC Characterization.
(DOC)

Table S9 hESC Safety Testing Results.
(DOC)

Table S10 Delta Ct Values.
(XLS)

File S1 Informed Consent.
(DOC)

File S2 Clinical Site Signature Log.
(DOC)

File S3 Clinical Site Phone Log.
(DOC)

File S4 Monitor Sign-In Log.
(DOC)

File S5 Additional Comments Log.
(DOC)

File S6 Telephone Call Report Form.
(DOC)

File S7 Clinical Site Signature Log, Foreign.
(DOC)

File S8 Study Completion/Withdrawal/Exclusion Form.
(DOC)

File S9 Pre-Trial Screening Log.
(DOC)

File S10 Enrollment, Protocol, and Informed Consent Log.
(DOC)

File S11 Source Data.
(DOC)

File S12 Clinical Results Report Form.
(DOC)

File S13 Blood Sample Archiving Form.
(DOC)

File S14 Blood Archiving Form.
(DOC)

File S15 Adverse Events Form.
(DOC)

File S16 Withdrawal from Study Form.
(DOC)

File S17 Request for Contact with the Donor Form.
(DOC)

File S18 Donor Code Log.
(DOC)

File S19 Embryo Log.
(DOC)

File S20 Health and Acceptance Questionnaire.
(DOC)

File S21 Demography.
(DOC)

File S22 Informed Consent.
(DOC)

File S23 IVF Treatment Cycles.
(DOC)

File S24 Laboratory Tests Prior to IVF.
(DOC)

File S25 Medical History Authorization.
(DOC)

File S26 Physical Examination.
(DOC)

File S27 Eligibility Verification Checklist.
(DOC)

File S28 Exclusion Criteria.
(DOC)

File S29 Medication Questionnaire.
(DOC)

File S30 Disease Deferral List.
(DOC)

File S31 Appendix 3, Medication Deferral List.
(DOC)

File S32 Appendix 4, Malaria & vCJD Risk Countries.
(DOC)
