# Supplementary figures and images for: Derivation of Xeno-Free and GMP-Grade Human Embryonic Stem Cells – Platforms for Future Clinical Applications
Source: PLoS One. 2012 Jun 20;7(6):e35325. doi: 10.1371/journal.pone.0035325 (PMC3380026; doi:10.1371/journal.pone.0035325)

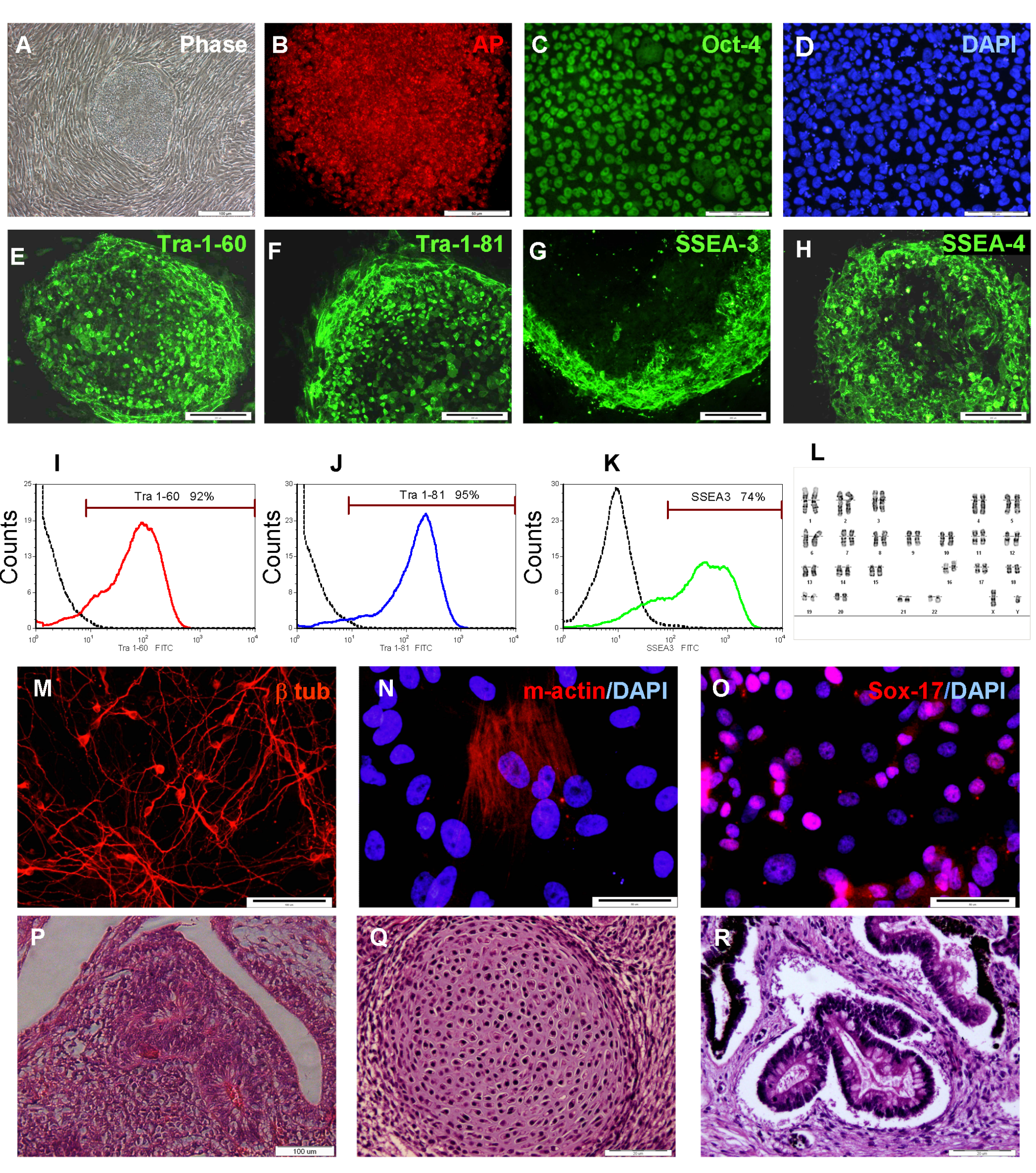

Supplement: Figure S1 — Characterization of the primary cell bank of clinical grade HADC102 hESCs. The hESCs colonies were comprised of small tightly packed cells with a high nuclear to cytoplasmic ratio. Clear distinguishable borders were observed between the colonies and cord feeder cells, (A; phase-contrast image). The cells expressed alkaline phosphatase (B; AP, fluorescence image). Indirect immunofluorescence staining showed that the hESCs were immunoreactive with anti-Oct-4 (C; D, nuclear 4',6-Diamidino-2-phenylindole (DAPI) counter staining), TRA-1-60 (E), TRA-1-81 (F), SSEA-3 (G), and SSEA-4 (H). FACS analysis showed that the majority of cells expressed markers of pluripotency TRA-1-60 (I), TRA-1-81 (J) and SSEA-3 (K) (Data from a representative experiment). The hESCs had normal karyotype (46, XY; L) and could differentiate in vitro in vivo into cells representing the three embryonic germ layers (M-R). Immunofluorescence staining showing in vitro differentiated cells expressing beta-tubulin III (ectoderm, M), muscle actin (mesoderm, N) and sox-17 (endoderm, O). Hematoxylin-eosin stained histological sections of teratoma tumors showing neural rosettes (ectoderm, P), cartilage (mesoderm, Q) and columnar glandular epithelium with goblet cells (endoderm, R). Scale bar represent 20 um for (Q and R), 50 um for (B, C, D, N and O), 100 um for (A, M and P) and 200 um for (E -H). (TIF) [file pone.0035325.s001.tif]

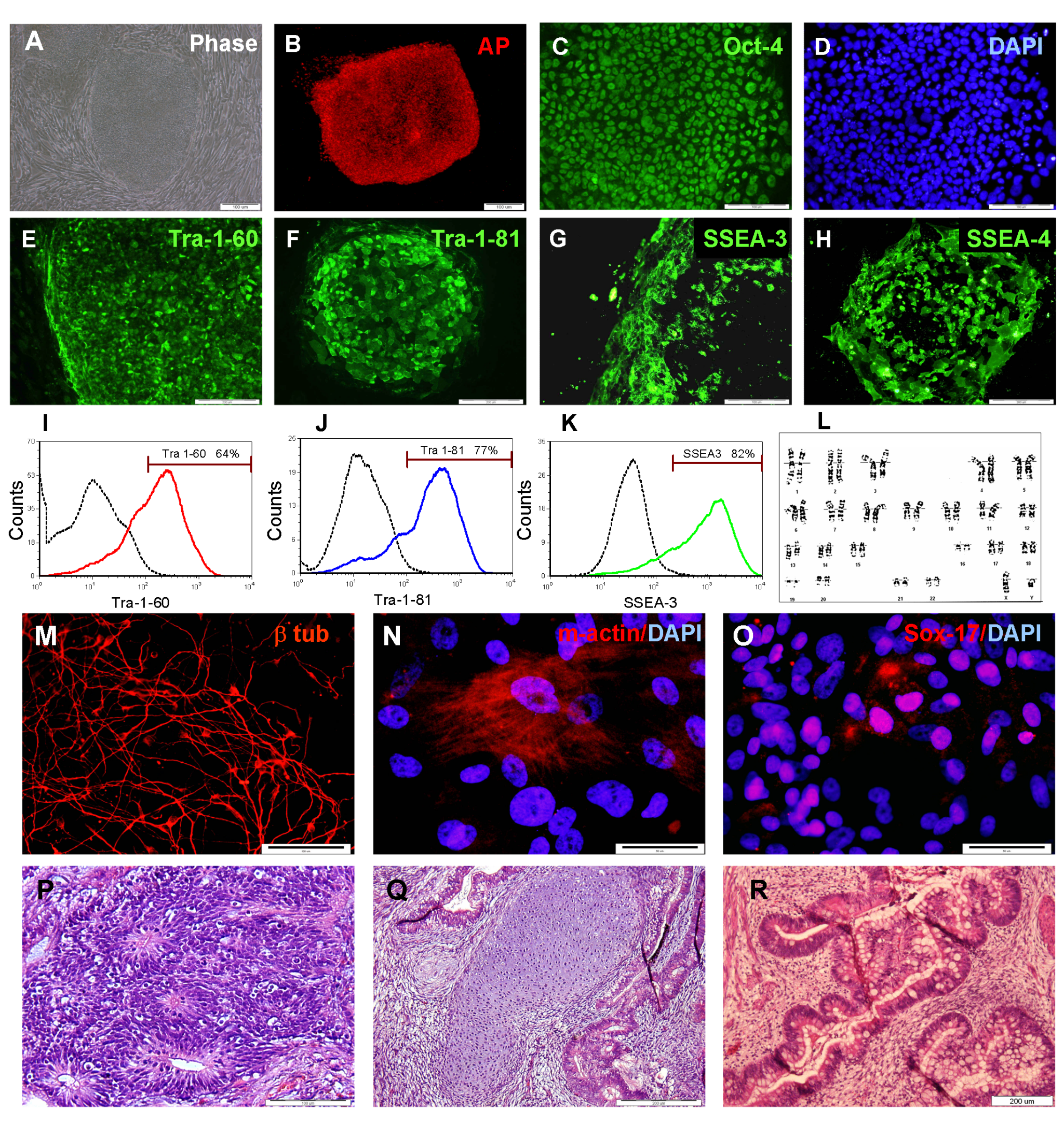

Supplement: Figure S2 — Characterization of the primary cell bank of clinical grade HADC106 hESCs The hESCs colonies were comprised of small tightly packed cells with a high nuclear to cytoplasmic ratio. Clear distinguishable borders were observed between the colonies and cord feeder cells, (A; phase-contrast image). The cells expressed alkaline phosphatase (B; AP, fluorescence image). Indirect immunofluorescence staining showed that the hESCs were immunoreactive with anti-Oct-4 (C; D, nuclear 4',6-Diamidino-2-phenylindole (DAPI) counter staining), TRA-1-60 (E), TRA-1-81 (F), SSEA-3 (G), and SSEA-4 (H). FACS analysis showed that the majority of cells expressed markers of pluripotency TRA-1-60 (I), TRA-1-81 (J) and SSEA-3 (K) (Data from a representative experiment). The hESCs had normal karyotype (46, XY; L) and could differentiate in vitro in vivo into cells representing the three embryonic germ layers (M–R). Immunofluorescence staining showing in vitro differentiated cells expressing beta-tubulin III (ectoderm, M), muscle actin (mesoderm, N) and sox-17 (endoderm, O). Hematoxylin-eosin stained histological sections of teratoma tumors showing neural rosettes (ectoderm, P), cartilage (mesoderm, Q) and villi structures with columnar glandular epithelium and goblet cells (endoderm, R). Scale bar represent 50 um for (N and O), 100 um for (A, B, C, D, G, M and P) and 200 um for (E, F, H, Q, R). (TIF) [file pone.0035325.s002.tif]

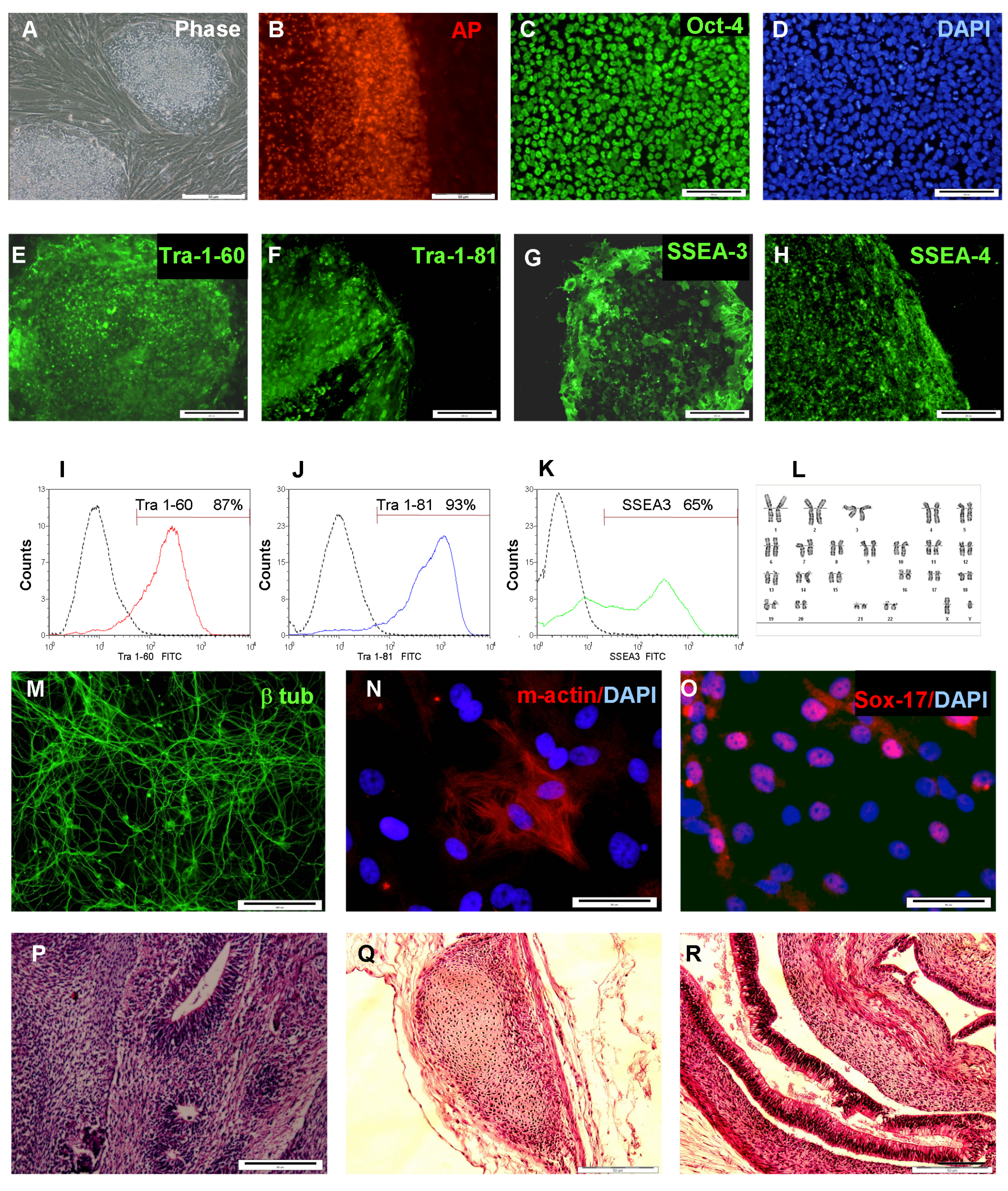

Supplement: Figure S3 — Characterization of the secondary cell bank of clinical grade HADC100 hESCs. The hESCs colonies were comprised of small tightly packed cells with a high nuclear to cytoplasmic ratio. Clear distinguishable borders were observed between the colonies and cord feeder cells, (A; phase-contrast image). The cells expressed alkaline phosphatase (B; AP, fluorescence image). Indirect immunofluorescence staining showed that the hESCs were immunoreactive with anti-Oct-4 (C; D, nuclear 4',6-Diamidino-2-phenylindole (DAPI) counter staining), TRA-1-60 (E), TRA-1-81 (F), SSEA-3 (G), and SSEA-4 (H). FACS analysis showed that the majority of cells expressed markers of pluripotency TRA-1-60 (I), TRA-1-81 (J) and SSEA-3 (K) (Data from a representative experiment). The hESCs had normal karyotype (46, XY; L) and could differentiate in vitro and in vivo into cells representing the three embryonic germ layers (M–R). Immunofluorescence staining showing in vitro differentiated cells expressing β-tubulin III (ectoderm, M), muscle actin (mesoderm, N) and sox-17 (endoderm, O). Hematoxylin-eosin stained histological sections of teratoma tumors showing neural rosettes (ectoderm, P), cartilage (mesoderm, Q) and columnar glandular epithelium with goblet cells (endoderm, R). Scale bar represent 50 um for (A, B, N, O, P, Q and R), 100 um for (C, D and M) and 200 um for (E–H). (TIF) [file pone.0035325.s003.tif]

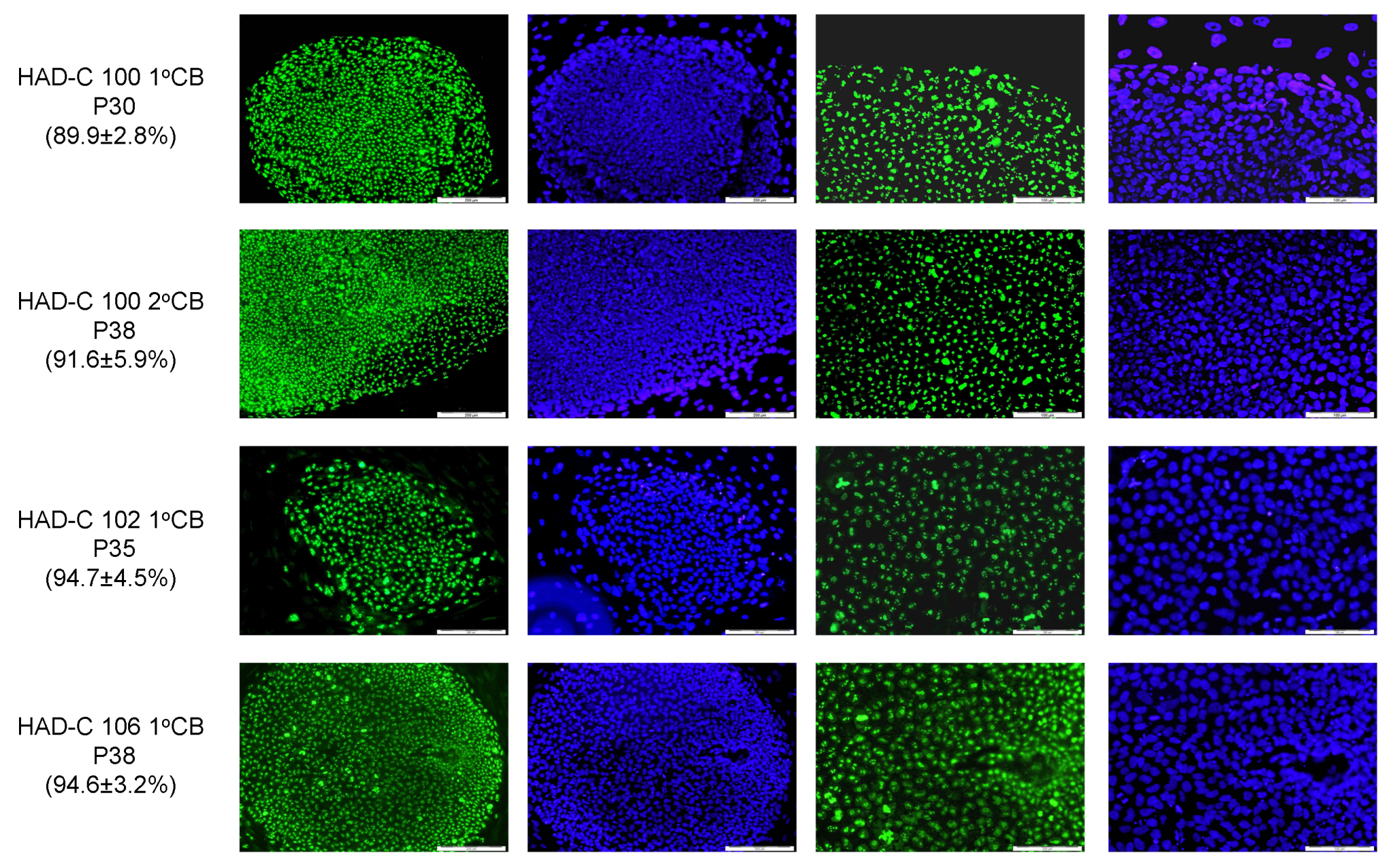

Supplement: Figure S4 — The majority of hESCs are in an active cell cycle. Indirect immunofluorescence analysis shows that the majority of hESCs in each of the cell banks are immunoreactive with anti-KI67. (Green: Quantitative analysis of the percentage of KI67+ cells within 200 cells in each of three random fields appears in brackets). Low magnification images and corresponding nuclei DAPI counterstaining are presented in the left two columns, while higher magnification in the right two columns. (TIF) [file pone.0035325.s004.tif]
