# Supplementary material for: Derivation of Xeno-Free and GMP-Grade Human Embryonic Stem Cells – Platforms for Future Clinical Applications
Source: PLoS One. 2012 Jun 20;7(6):e35325. doi: 10.1371/journal.pone.0035325 (PMC3380026; doi:10.1371/journal.pone.0035325)
Supplement: Table S1 — Feeder and hESC Materials. (DOC) [file pone.0035325.s005.doc]

TABLE S1

MATERIALS USED

**FEEDER DERIVATION**

| **QC Tested** | **Grade** | **Catalogue #** | **Supplier** | **Material** |
| --- | --- | --- | --- | --- |
|  | GMP | SH 30081.01 | Hyclone | DMEM |
|  | GMP | SH 30034.01 | Hyclone | L-Glutamine |
|  | GMP | SH 30221-17 | Hyclone | Sterile Water for Injection |
|  | GMP | SH 30028.02 | Hyclone | PBS without Ca or Mg |
| LAL | GMP | WAK-DMSO-10 | WAK Chemie | Cryosure DMSO-USP |
| LAL, Sterility, Mycoplasma | GMP | 14-498E | Lonza | Human Serum, Male Only AB |
|  | GMP | 12563-011 | Invitrogen | TrypLE Select |
| LAL, Sterility, Mycoplasma | GMP | RhG100-001 | Fibrogen | rh Gelatin 100 kD |

**hESC DERIVATION**

| **QC Tested** | **Grade** | **Catalogue #** | **Supplier** | **Material** |
| --- | --- | --- | --- | --- |
| LAL, Sterility, Mycoplasma | Non-GMP production with GMP-like documentation | 233-FB-025/CF +  20 mM Tris, 1.0 M NaCl, pH 7.0 | R&D Systems | Recombinant-human basic FGF, Carrier Free (146 aa) + Tris |
|  | GMP | ART-1029 | Cooper Surgical | Quinns Advantage® **Blastocyst Medium** |
|  | GMP | ART-1026/27 | Cooper Surgical | Quinn's Advantage® Cleavage Medium |
|  | GMP | ART-  3010/11 | Cooper Surgical | Quinn's Advantage® Serum Protein Supplement |
|  | GMP | ART-4008 | Cooper Surgical | Quinn's Oil for Tissue Culture |
|  | GMP | 90126 | Irvine Scientific | HTF - Hepes |
|  | GMP | 90124 | Irvine Scientific | Embryo Thaw Media |
| LAL, Sterility, Mycoplasma | GMP | 9988 | Irvine Scientific | HSA  Human Serum Albumin (100mg/ml) |
|  | GMP | 99193 | Irvine Scientific | Synthetic Serum Substitute |
|  | GMP | ART-8019 | Cooper Surgical | Quinn's Advantage Thaw Kit |
| LAL, Sterility, Mycoplasma | GMP | 2001 | CellGenix | CellGro® SCGM |
|  | GMP | SH30238.01 | Hyclone | Nonessential Amino Acids |
| LAL, Sterility, Mycoplasma | GMP | IR-90137 | Irvine Scientific | Vit Kit Thaw |
| LAL, Sterility, Mycoplasma | GMP | 640222 | BioLife Solutions | CryoStor CS10 |
| LAL, Sterility, Mycoplasma | GMP | IR-90133-SO | Irvine Scientific | Vit- Kit Freeze Solutions |
