# Supplementary material for: Derivation of Xeno-Free and GMP-Grade Human Embryonic Stem Cells – Platforms for Future Clinical Applications
Source: PLoS One. 2012 Jun 20;7(6):e35325. doi: 10.1371/journal.pone.0035325 (PMC3380026; doi:10.1371/journal.pone.0035325)
Supplement: Table S2 — Feeder Characterization MCB. (DOC) [file pone.0035325.s006.doc]

TABLE S2

FIBROBLAST FEEDER MASTER CELL BANK QC CHARACTERIZATION RESULTS

| HEM 001 MCB | FOR 002 MCB* | CRD 009 MCB | CRD 008 MCB | CRD 007 MCB | Specification | QC TEST |
| --- | --- | --- | --- | --- | --- | --- |
| **PRE-FREEZING** | | | | | | |
| 2 x 10^6^ | 2 x 10^6^ | 2 x 10^6^ | 2 x 10^6^ | 2 x 10^6^ | 2 x 10^6^/ampoule | Cell Count |
| 99% | 99% | 96% | 98% | 97% | NLT 80% viable cells | Viability |
| <0.48 EU/ml | <0.48 EU/ml | <0.48 EU/ml | <1.92 EU/ml | <0.48 EU/ml | < 5.0 EU/ml | LAL |
| Sterile | Sterile | Sterile | Sterile | Sterile | Sterile | Sterility |
| Absent | Absent | Absent | Absent | Absent | Absent | Mycoplasma (Culture) |
| **POST THAWING** | | | | | | |
| √ | √ | √ | √ | √ | Monolayer of adherent cells | Microscopic Exam of Feeders |
| √ | √ | √ | √ | √ | Elongated, fibroblast-like |  |
| √ | √ | √ | √ | √ | High cytoplasm to nuclear ratio |  |
| 1.05 x 10^6^ | 0.95 x 10^6^ | 1.0 x 10^6^ | 1.6 x 10^6^ | 1.23 x 10^6^ | NLT 0.5 x 10^6^ viable cells per vial | Cell Counting |
| 94.3% | N/A | 95.8% | 96.8% | 95.9% |  | Viability (post thawing) |
| 82% | 79% | 76% | 80.8% | 89.8% | NLT 70% cells stain positive when 200 cells are counted | Staining for Vimentin |
| 96% | 95% | 90.2% | 94.5% | 92.2% | NLT 70% cells stain positive when 200 cells are counted | Staining for Anti-human fibroblasts |
| 98% | 96% | 94.7% | 98.7% | 98% | NLT 70% cells stain positive when 200 cells are counted | Staining for CD44 |
| Pass, 46XX | Pass, 46XY | Pass, 46XY | Pass, 46XY | Pass, 46XX | Lot fails if 3 repeat deletions or 2 repeat additions to the chromosomes are noted | Karyotyping of feeders  30 metaphases |
| √ | √ | √ | √ | √ | Definitive human STR profile | DNA Fingerprinting STR |
| 23.5 hours | 40.7 hours | 30.5 hours | 23.4 hours | 24.5 hours | Less than 50 hours | Doubling time of feeders |
| Pass | Pass | Pass | Pass | Pass | Lot fails if 3 repeat deletions or 2 repeat additions to the chromosomes are noted | Karyotyping of hESC prior to seeding on feeders  (hESC NMT P_30_)  30 metaphases |
| **Support of hESC grown on fibroblasts for 1-5 passages** | | | | | | |
| √ | √ | √ | √ | √ | hESC colonies tightly packed cells | Microscopic Exam of hESC on feeders |
| √ | √ | √ | √ | √ | Clear distinguishable border towards feeders |  |
| √ | √ | √ | √ | √ | hESCs small relative to fibroblasts |  |
| √ | √ | √ | √ | √ | High nucleus to cytoplasm ratio of hESCs |  |
| √ | √ | √ | √ | √ | Prominent nucleoli visible in some cells |  |
| SSEA-4 = 89%  TRA 1-60 = 93%  TRA 1-81 = 91% | SSEA-4 = 96%  TRA 1-60 = 76%  TRA 1-81 = 73% | SSEA-4 = 92%  TRA 1-60 = 93%  TRA 1-81 = 93% | SSEA-4 = 76%  TRA 1-60 = 86%  TRA 1-81 = 88% | SSEA-4 = 93%  TRA 1-60 = 92%  TRA 1-81 = 90% | More than 70% of the cells are positive | FACS of hESC for SSEA-4, TRA 1-60, TRA-1-81 |
| 6.5% | 0.7% | 2.4% | 1.9% | 2% | Less than 15% of cells are positive | FACS for SSEA-1 |
| 100% | 100% | 100% | 100% | 100% | More than 80% of colonies are positive | Staining of hESC for AP activity |
| 81% | 72% | 93% | 99.1% | 89.6% | More than 60% of cells are positive when 200 cells are counted | Staining of hESC for Oct-4 expression |
| 14 hours | 23.5 hours | 19.4 hours | 19.4 hours | 17.5 hours | Less than 40 hours | hESC Doubling Time |
| **Support of hESC grown on fibroblasts for 6-10 passages** | | | | | | |
| √ | √ | √ | √ | √ | hESC colonies tightly packed cells | Microscopic Exam of hESC on feeders |
| √ | √ | √ | √ | √ | Clear distinguishable border towards feeders |  |
| √ | √ | √ | √ | √ | hESCs small relative to fibroblasts |  |
| √ | √ | √ | √ | √ | High nucleaus to cytoplasm ratio of hESCs |  |
| √ | √ | √ | √ | √ | Prominent nucleoli visible in some cells |  |
| SSEA-4 = 92%  TRA 1-60 = 93%  TRA 1-81 = 90% | SSEA-4 = 95%  TRA 1-60 = 91%  TRA 1-81 = 86% | SSEA-4 = 91%  TRA 1-60 = 79%  TRA 1-81 = 83% | SSEA-4 = 96%  TRA 1-60 = 93%  TRA 1-81 = 91% | SSEA-4 = 94%  TRA 1-60 = 91%  TRA 1-81 = 90% | More than 70% of the cells are positive | FACS for SSEA-4, TRA 1-60, TRA-1-81 |
| 1.8% | 4.2% | 3.7% | 1.3% | 0.7% | Less than 15% of cells are positive | FACS for SSEA-1 |
| 100% | 100% | 100% | 100% | 100% | More than 80% of colonies are positive | Staining of hESC for AP activity |
| 99.7% | 100% | 99% | 99% | 93% | More than 60% of cells are positive | Staining of hESC for Oct-4 expression |
| 27.5 hours | 23.5 hours | 17.3 hours | 23 hours | 20.9 hours | Less than 40 hours | hESC Doubling Time |
| At least 2 cells stain positive for | | | | | | In vitro differentiation of hESC cultured on fibroblasts |
| √ | √ | √ | √ | √ | α-Feto-Protein (Endoderm) |  |
| √ | √ | √ | √ | √ | β-tubulin III (Ectoderm) |  |
| √ | √ | √ | √ | √ | muscle actin (Mesoderm) |  |
| Pass | Pass | Pass | Pass | Pass | Lot fails if 3 repeat deletions or 2 repeat additions to the chromosomes are noted | Karyotype of hESC grown on feeders after sampling for MCB characterization is complete  30 metaphases |
