# Supplementary material for: Derivation of Xeno-Free and GMP-Grade Human Embryonic Stem Cells – Platforms for Future Clinical Applications
Source: PLoS One. 2012 Jun 20;7(6):e35325. doi: 10.1371/journal.pone.0035325 (PMC3380026; doi:10.1371/journal.pone.0035325)
Supplement: Table S3 — Feeder Adventitious Virus Testing Results. (DOC) [file pone.0035325.s007.doc]

TABLE S3

FIBROBLAST FEEDER MCB AND WCB

ADVENTITIOUS VIRUS TESTING

| Test | Feeder  CRD008 MCB | Feeder  CRD008 WCB3 | Feeder  CRD008 WCB4 |
| --- | --- | --- | --- |
| Cell Growth (Viability) | 73% | 84% | 83% |
| Sterility Testing | Pass | Pass | Pass |
| Bacteriostasis | Pass | Pass | Pass |
| Mycoplasma | Pass | Pass | Pass |
| 28-day in vitro test | Pass | Pass | Pass |
| In-vivo assay for cell substrates | Pass | Pass | Pass |
| RT PCR-HIV 1/2 | Pass |  |  |
| RT PCR-HTLV 1/2 | Pass |  |  |
| RT PCR-HCV | Pass |  |  |
| RT PCR-HBV | Pass |  |  |
| RT PCR-EBV | Pass |  |  |
| RT PCR-CMV | Pass |  |  |
| RT RT-HHV6 | Pass |  |  |
| RT RT-HHV7 | Pass |  |  |
| RT RT-HHV8 | Pass |  |  |
| RT-B19, SV40 | Pass |  |  |
| RT-HAV | Pass |  |  |
| RT-BKV (Polyomavirus) | Pass |  |  |
| RT-JCV (Polyomavirus) | Pass |  |  |
| RT-HPV-(7 different kinds) | Pass |  |  |
| Ultrastructural examination (TEM) | Pass |  |  |
| Isoenzyme analysis | Human | Human | Human |
| In-vitro Tumorigenicity (growth in soft agar) | Pass |  |  |
| Karyotyping (50 metaphases) | Pass |  |  |
| LAL | <5.00 EU/ml | <0.60 EU/ml | <0.30EU/ml |
| West Nile Virus | Pass |  |  |
| DNA Fingerprinting (STR) | Unique Profile | Matches MCB profile | Matches MCB profile |
| RT-Varicella zoster | Pass |  |  |
| FPERT |  | Pass | Pass |

Bioreliance Laboratories, Glasgow UK
