# Supplementary material for: Derivation of Xeno-Free and GMP-Grade Human Embryonic Stem Cells – Platforms for Future Clinical Applications
Source: PLoS One. 2012 Jun 20;7(6):e35325. doi: 10.1371/journal.pone.0035325 (PMC3380026; doi:10.1371/journal.pone.0035325)
Supplement: Table S4 — Feeder Characterization WCB. (DOC) [file pone.0035325.s008.doc]

TABLE S4

FIBROBLAST IRRADIATED FEEDER WORKING CELL BANK QC CHARACTERIZATION RESULTS

| CRD 008 WCB5 | CRD 008 WCB4 | CRD 008 WCB3 | CRD 008 WCB2 | Specification | QC TEST |
| --- | --- | --- | --- | --- | --- |
| **PRE-FREEZING** | | | | | |
| 2 x 10^6^ | 2 x 10^6^ | 2 x 10^6^ | 2 x 10^6^ | 2 x 10^6^/ampoule | Cell Count |
| 98.1% | 95.8% | 98.8% | 98.5% | NLT 80% viable cells | Viability |
| <0.48 EU/ml | <0.48 EU/ml | <0.48 EU/ml | <0.48 EU/ml | < 5.0 EU/ml | LAL |
| Sterile | Sterile | Sterile | Sterile | Sterile | Sterility |
| Absent | Absent | Absent | Absent | Absent | Mycoplasma (Culture) |
| **POST-THAWING** | | | | | |
| √ | √ | √ | √ | Monolayer of adherent cells | Microscopic Exam of Feeders |
| √ | √ | √ | √ | Elongated, fibroblast-like |  |
| √ | √ | √ | √ | High cytoplasm to nuclear ratio |  |
|  | | | | | Proliferative Ability |
| √ | √ | √ | √ | No fibroblasts stain positive when 200 cells are counted | BrdU Incorporation |
| √ | √ | √ | √ | No fibroblasts stain positive when 200 cells are counted | KI-67 Staining |
| 0.75 x 10^6^ | 1.1 x 10^6^ | 1.5 x 10^6^ | 1.4 x 10^6^ | NLT 0.5 x 10^6^ viable cells | Cell Counting |
| 92.5% | 92.4% | 98.4% | 95% |  | Viability |
| 100% | 100% | 100% | 100% | NLT 70% cells stain positive when 200 cells are counted | Staining for Vimentin |
| 88% | 83% | 96% | 92% | NLT 70% cells stain positive when 200 cells are counted | Staining for Anti-human fibroblasts |
| 92% | 93.2% | 96% | 97.4% | NLT 70% cells stain positive when 200 cells are counted | Staining for CD44 |
| Pass | Pass | Pass | Pass | Lot fails if 3 repeat deletions or 2 repeat additions to the chromosomes are noted | Karyotyping of hESC prior to seeding on feeders  (hESC NMT P_45_)  30 metaphases |
| **Support of hESC grown on fibroblasts for 1-5 passages** | | | | | |
| √ | √ | √ | √ | hESC colonies tightly packed cells | Microscopic Exam of hESC Grown on Feeders |
| √ | √ | √ | √ | Clear distinguishable border towards feeders |  |
| √ | √ | √ | √ | hESCs small relative to fibroblasts |  |
| √ | √ | √ | √ | High nucleaus to cytoplasm ratio of hESCs |  |
| √ | √ | √ | √ | Prominent nucleoli visible in some cells |  |
| SSEA-4 = 88%  TRA 1-60 = 89%  TRA 1-81 = 84% | SSEA-4 = 92%  TRA 1-60 = 93%  TRA 1-81 = 93% | SSEA-4 = 73%  TRA 1-60 = 87%  TRA 1-81 = 85% | SSEA-4 = 93%  TRA 1-60 = 95%  TRA 1-81 = 65% | More than 70% of the cells are positive | FACS for SSEA-4, TRA 1-60, TRA-1-81 |
| 1% | 0.7% | 0.3% | 2.4% | Less than 15% of cells are positive | FACS for SSEA-1 |
| 100% | 100% | 100% | 100% | More than 80% of colonies are positive | Staining of hESC for AP activity |
| 100% | 100% | 92% | 100% | More than 60% of cells are positive | Staining of hESC for Oct-4 expression |
| 24 hours | 35 hours | 33 hours | 23 hours | Less than 40 hours | hESC Doubling Time |
| **Support of hESC grown on fibroblasts for 6-10 passages** | | | | | |
| √ | √ | √ | √ | hESC colonies tightly packed cells | Microscopic Exam of hESC Grown on Fibroblasts |
| √ | √ | √ | √ | Clear distinguishable border towards feeders |  |
| √ | √ | √ | √ | hESCs small relative to fibroblasts |  |
| √ | √ | √ | √ | High nucleus to cytoplasm ratio of hESCs |  |
| √ | √ | √ | √ | Prominent nucleoli visible in some cells |  |
| SSEA-4 = 94%  TRA 1-60 = 96%  TRA 1-81 = 94% | SSEA-4 = 83%  TRA 1-60 = 93%  TRA 1-81 = 89% | SSEA-4 = 90%  TRA 1-60 = 91%  TRA 1-81 = 89% | SSEA-4 = 82%  TRA 1-60 = 87%  TRA 1-81 = 89% | More than 70% of the cells are positive | FACS for SSEA-4 TRA 1-60, TRA-1-81 |
| 0.4% | 1.0% | 1.1% | 0.8% | Less than 15% of cells are positive | FACS for SSEA-1 |
| 100% | 100% | 100% | 100% | More than 80% of colonies are positive | Staining of hESC for AP activity |
| 100% | 96% | 100% | 99% | More than 60% of cells are positive | Staining of hESC for Oct-4 expression |
| 16 hours | 19 hours | 16 hours | 24 hours | Less than 40 hours | hESC Doubling Time |
| At least 2 cells stain positive for | | | | | In vitro differentiation of hESC cultured on fibroblasts |
| √ | √ | √ | √ | sox-17 (Endoderm) |  |
| √ | √ | √ | √ | β-tubulin III (Ectoderm) |  |
| √ | √ | √ | √ | muscle actin (Mesoderm) |  |
| Pass | Pass | Pass | Pass | NLT 75% of cells are normal | Karyotype of hESC grown on feeders after sampling for WCB characterization is complete  30 metaphases |
