# Supplementary material for: Derivation of Xeno-Free and GMP-Grade Human Embryonic Stem Cells – Platforms for Future Clinical Applications
Source: PLoS One. 2012 Jun 20;7(6):e35325. doi: 10.1371/journal.pone.0035325 (PMC3380026; doi:10.1371/journal.pone.0035325)
Supplement: Table S5 — Donor Testing Results. (DOC) [file pone.0035325.s009.doc]

TABLE S5

DONOR TESTING RESULTS

| **DONOR**  **NUMBER** | Syphilis TPA | Gonorr. | HIV 1 | HIV 2 | HTLV 1 | HTLV 2 | Chlamyd. IgG | Chlamyd. IgA | HCV | Anti HB_core_ | Rubella  IgG • | Rubella IgM | CMV IGG• | CMV  IgM | HB Sag | EBV IgG | EBV IgM | EBNA IgG | Strep. | Malaria | CBC | Toxoplas.  IgG | Toxoplas.  IgM | Blood Type | Other |
| --- | --- | --- | --- | --- | --- | --- | --- | --- | --- | --- | --- | --- | --- | --- | --- | --- | --- | --- | --- | --- | --- | --- | --- | --- | --- |
| FEEDER LOT CRD008 | | | | | | | | | | | | | | | | | | | | | | | | | |
| Male | - | - | - | - | - | - | - | - | - | - | + | - | + | - | - | + | - | + |  |  | N | - | - | A+ |  |
| Female | - | - | - | - | - | - | - | - | - | - | + | - | + | - | - | - | - | - | - |  | N | - | - | B+ |  |
| Cord | - |  | - | - | - | - | - | - | - | - | + | - | + | - | - | - | - | - |  |  | N | - | - | A+ |  |
| HESC LINE HAD-C 100 | | | | | | | | | | | | | | | | | | | | | | | | | |
| Male | - | - | - | - | - | - | - | - | - | - | + | - | - | - | - | - | - | - |  | - | N |  |  | AB+ |  |
| Female | - | - | - | - | - | - | - | - | - | - | + | - | + | - | - | + | - | + |  | - | N |  |  | A+ |  |
| HESC LINE HAD-C 102 | | | | | | | | | | | | | | | | | | | | | | | | | |
| Male | - | - | - | - | - | - | - | - | - | - | - | - | - | - | - | + | - | + |  | - | N |  |  | O+ |  |
| Female | - | - | - | - | - | - | B | + | - | - | + | - | + | - | - | + | - | + |  | - | N |  |  | AB+ | Checked 2x |
| HESC LINE HAD-C 106 | | | | | | | | | | | | | | | | | | | | | | | | | |
| Male | - | - | - | - | - | - | - | - | - | - | + | - | + | - | - | - | - | - |  | - | N |  |  | A+ |  |
| Female | - | - | - | - | - | - | - | - | - | - | + | - | + | - | - | - | - | - |  | - | N |  |  | A+ |  |

AMERICAN MEDICAL LABORATORIES, HERZLIYA ISRAEL

[[1]](#endnote-2)

1. N = Normal; B = Borderline; + = Positive Response; - = Negative Response; • = Immune Response; Blank = Not tested [↑](#endnote-ref-2)
