# Supplementary material for: Derivation of Xeno-Free and GMP-Grade Human Embryonic Stem Cells – Platforms for Future Clinical Applications
Source: PLoS One. 2012 Jun 20;7(6):e35325. doi: 10.1371/journal.pone.0035325 (PMC3380026; doi:10.1371/journal.pone.0035325)
Supplement: Table S6 — List of Forms. (DOC) [file pone.0035325.s010.doc]

TABLE S6

DERIVATION OF NEW CLINICAL-GRADE hESC LINES STUDY FORMS AND CRFS

| **NAME OF FORM** | **FORM #** | **SUPPORTING FILE #** | **IN STUDY BINDER** | **IN SUBJECT BINDER** |
| --- | --- | --- | --- | --- |
| Clinical Site Signature Log | 1.01 | 2 | √ |  |
| Clinical Site Phone Log | 1.02 | 3 | √ |  |
| Monitor Sign-In Log | 1.03 | 4 | √ |  |
| Additional Comments Log | 1.04 | 5 |  | √ |
| Telephone Call Report Form | 1.05 | 6 |  | √ |
| Clinical Site Signature Log, Foreign | 1.06 | 7 | √ |  |
| Study Completion/Withdrawal/Exclusion Form | 2.01 | 8 |  | √ |
| Pre-Trial Screening Log | 2.02 | 9 | √ |  |
| Enrollment, Protocol, and Informed Consent Log | 2.03 | 10 | √ |  |
| Source Data | 2.04 | 11 |  | √ |
| Clinical Results Report Form | 2.05 | 12 |  | √ |
| Blood Sample Archiving Form | 2.07 | 13 |  | √ |
| Blood Archiving Form | 2.08 | 14 | √ |  |
| Adverse Events Form | 3.01 | 15 |  | √ |
| Withdrawal From Study Form | 3.02 | 16 |  | √ |
| Request for Contact with the Donor Form | 3.03 | 17 |  | √ |
| Donor Code Log | 3.05 | 18 | √ |  |
| Embryo Log | 3.06 | 19 |  | √ |
| Health and Acceptance Questionnaire | CRF | 20 |  | √ |
| Demography | CRF | 21 |  | √ |
| Informed Consent | CRF | 22 |  | √ |
| IVF Treatment Cycles | CRF | 23 |  | √ |
| Laboratory Tests Prior to IVF | CRF | 24 |  | √ |
| Medical History Authorization | CRF | 25 |  | √ |
| Physical Examination | CRF | 26 |  | √ |
| Eligibility Verification Checklist | CRF | 27 |  | √ |
| Exclusion Criteria | CRF | 28 |  | √ |
| Medication Questionnaire | CRF | 29 |  | √ |
| Disease Deferral List | CRF | 30 |  | √ |
| Appendix 3, Medication Deferral List | CRF | 31 | √ |  |
| Appendix 4, Malaria & vCJD Risk Countries | CRF | 32 | √ |  |

CRF = Case Report Form

Subject Binder = On Site at Trial Site

Subject Binder = Individual Donor's CRF
