# Supplementary material for: Derivation of Xeno-Free and GMP-Grade Human Embryonic Stem Cells – Platforms for Future Clinical Applications
Source: PLoS One. 2012 Jun 20;7(6):e35325. doi: 10.1371/journal.pone.0035325 (PMC3380026; doi:10.1371/journal.pone.0035325)
Supplement: Table S7 — Blastocyst Table. (DOC) [file pone.0035325.s011.doc]

TABLE S7

BLASTOCYSTS TABLE FOR hESC LINE DEVELOPMENT

| Notes | hESC Line | Plated As | Day of Blastocyst Development | Blastocyst Grade  (Expansion-TE-ICM) | Blastocyst # |
| --- | --- | --- | --- | --- | --- |
|  | **HAD-C 102** | ICM | 6 | 6AB | 1 |
|  | Initial stock | ICM | 6 | 6AA | 2 |
|  |  | ICM | 6 | 6AA | 3 |
| Some expansion in overall size, zona pellucida beginning to thin |  | Whole | 7 | 2AB | 4 |
| Some expansion in overall size, zona pellucida beginning to thin | Initial stock | Whole | 7 | 2AA | 5 |
|  |  | ICM | 6 | 6BA | 6 |
|  |  | ICM | 7 | 6AB | 7 |
| Degenerative ICM or no ICM visible | Initial stock | Whole | 7 | 6AE | 8 |
|  |  | ICM | 6 | 6AA | 9 |
|  |  | ICM | 7 | 6AA | 10 |
|  |  | ICM | 5 | 5AB | 11 |
|  |  | ICM | 6 | 4AA | 12 |
|  | **HAD-C 106** | ICM | 6 | 4AB | 13 |
|  | Initial stock | ICM | 7 | 6AA | 14 |
|  |  | ICM | 7 | 4AB | 15 |
| Blastocyst collapse during ICM isolation |  | Whole | 7 | 6AC | 16 |
| ICM loss during isolation |  | not plated | 7 | 5AB | 17 |
| ICM loss during isolation |  | not plated | 6 | 5AB | 18 |
| ICM loss during isolation |  | not plated | 6 | 6AB | 19 |
|  |  | ICM | 6 | 4AB | 20 |
|  |  | ICM | 6 | 5AB | 21 |
|  |  | ICM | 6 | 6AA | 22 |
|  |  | ICM | 6 | 6AA | 23 |
|  |  | ICM | 6 | 7AA | 24 |
|  | **HAD-C 100** | ICM | 6 | 5AA | **25** |
|  | Initial stock | ICM | 6 | 4AA | 26 |
| Degenerative ICM or no ICM visible |  | Whole | 6 | 7AE | 27 |
| Degenerative ICM or no ICM visible |  | Whole | 6 | 4AE | 28 |
| Degenerative ICM or no ICM visible |  | Whole | 6 | 6BD | 29 |
| Degenerative ICM or no ICM visible |  | Whole | 6 | 6AE | 30 |
|  |  | ICM | 7 | 6AA | 31 |
|  |  | ICM | 6 | 6AB | 32 |
|  |  | ICM | 6 | 6BA | 33 |
|  |  | ICM | 6 | 4AA | 34 |
