# Supplementary material for: Derivation of Xeno-Free and GMP-Grade Human Embryonic Stem Cells – Platforms for Future Clinical Applications
Source: PLoS One. 2012 Jun 20;7(6):e35325. doi: 10.1371/journal.pone.0035325 (PMC3380026; doi:10.1371/journal.pone.0035325)
Supplement: Table S8 — hESC Characterization. (DOC) [file pone.0035325.s012.doc]

TABLE S8

hESC LINE CHARACTERIZATION

[[1]](#endnote-2)

| QC Test | Specification | HAD-C 100 | | | HAD-C 102 | | | HAD-C 106 | | |
| --- | --- | --- | --- | --- | --- | --- | --- | --- | --- | --- |
|  |  | Stock | 1^o^ Bank | 2^o^ Bank | Stock | 1^o^ Bank | 2^o^ Bank | Stock | 1^o^ Bank | 2^o^ Bank |
| Clump # | NLT 5 clumps/vial or straw | **√** | **√** | **√** | **√** | **√** |  | **√** | **√** |  |
| LAL | < 5.0 EU/ml | <0.48 EU/ml | <0.48 EU/ml | <0.48 EU/ml | <0.48 EU/ml | <0.48 EU/ml | <0.48 EU/ml | <0.48 EU/ml | 0.03 EU/ml | <0.03 EU/ml |
| Sterility | Sterile | pass | pass | pass | pass | pass | pass | pass | pass | pass |
| Mycoplasma | Absent | pass | pass | pass | pass | pass | pass | pass | pass | pass |
| Cluster Survival | At least 1 cluster attaches and develops to a colony 4-7 days after thawing | **√** | **√** | **√** | **√** | **√** |  | **√** | **√** |  |
| Morphology | *hESC Colonies* |  | | | | | | | | |
|  | hESC colonies tightly packed cells | **√** | **√** | **√** | **√** | **√** |  | **√** | **√** |  |
|  | Clear, distinguishable borders towards feeders | **√** | **√** | **√** | **√** | **√** |  | **√** | **√** |  |
|  | hESC small relative to fibroblasts | **√** | **√** | **√** | **√** | **√** |  | **√** | **√** |  |
|  | High nucleus to cytoplasm ratio of hESC | **√** | **√** | **√** | **√** | **√** |  | **√** | **√** |  |
|  | Prominent nucleoli visible in some cells | **√** | **√** | **√** | **√** | **√** |  | **√** | **√** |  |
| HLA Typing(Optional) | Definive HLA profile | **√** (P_14_) | **√** (P_25_) | (Optional) | **√** (P_27_) | **√** (P_29_) |  | (Optional) | **√** (P_32_) |  |
| DNA Fingerprinting—STR Profile | Unique STR profile | **√** | **√** | **√** | **√** | **√** |  | **√** | **√** |  |
| Karyotype of hESCs | Lot fails if 3 repeat deletions or 2 repeat additions to the chromosomes are noted | Pass  (P_12_)  30 metaphases | Pass  (P_17_)  50 metaphases | Pass  (P_30_)  30 metaphases | Pass  (P_21_)  30 metaphases | Pass  (P_26_)  50 metaphases |  | Pass  (P_37_)  30 metaphases | Pass  (P_44_)  50 metaphases |  |
| Staining of hESC for Alkaline Phosphatase activity | NLT 60% of the colonies are positive | 100%  (P_9)_ | 100%  (P_16_) | 100%  (P_32_) | 100%  (P_16_) | 100%  (P_32_) |  | 100%  (P_16_) | 100%  (P_33_) |  |
| At least 3 Antigens will be detected by FACS and 3 by Immunostaining of the Following | | | | | | | | | | |
| Immunostaining of | NLT 60% of colonies or cells stain positive |  | | | | | | | | |
| Nanog |  |  |  |  |  |  |  |  |  |  |
| SSEA3 |  |  | (P_23_) 100% | (P_44_) 100% |  | (P_27_) 100% |  |  | (P_43_) 100% |  |
| SSEA4 |  |  | (P_24_) 100% | (P_32_) 100% |  | (P_28_) 100% |  |  | (P_43_) 100% |  |
| TRA 1-60 |  | (P_17_) 100% | (P_27_) 100% | (P_44_) 100% | (P_15_) 100% | (P_28_) 100% |  | (P_24_) 100% | (P_38_) 100% |  |
| TRA 1-81 |  |  | (P_24_) 100% | (P_32_) 100% |  | (P_28_) 100% |  |  | (P_43_) 100% |  |
| Oct4 |  | (P_16_) 100% | (P_24_) 100% | (P_33_) 100% | (P_17_) 100% | (P_35_) 100% |  | (P_19_) 100% | (P_38_) 100% |  |
| FACS for | NLT 50% of cells are positive |  | | | | | | | | |
| TRA 1-81 |  |  | (P_26_)92% | (P_45_)93% |  | (P_30_)93% |  |  | (P_39_)77% |  |
| TRA 1-60 |  |  | (P_26_)89% | (P_42_)87% |  | (P_30_)87% |  |  | (P_45_)64% |  |
| AP (TRA 2-54) |  |  |  |  |  |  |  |  |  |  |
| CD9 (TG30) |  |  |  |  |  |  |  |  |  |  |
| THY1 (F15-14-1) |  |  |  |  |  |  |  |  |  |  |
| SSEA3 |  |  | (P_28_)54% | (P_45_)63% |  | (P_30_)74% |  |  | (P_42_)82% |  |
| SSEA1 | NMT 50% of the cells are positive |  | (P_30_)36% | (P_44_)13.7 |  | (P_35_)26% |  |  | (P_39_)5% |  |
| Pluripotency  RT-PCR  (Optional)^39^ | Typical hESC profile with markers of pluripotency and differentiation when EBs are tested |  | **√** (P_32 hESC_)  (P_23 EBs_) |  |  | **√** (P_33 hESC_)  (P_38 EBs_) |  |  | **√** (P_35 hESC_)  (P_40 EBs_) |  |
| hESC Doubling Time | ≤ 170 hours |  | 35.6 hours  (P_24_) | 38.7 hours  (P_43_) |  | 35 hours  (P_35_) |  |  | 41.8 hours  (P_38_) |  |
| KI-67 Staining | NLT 60% cells stain positive when 200 cells are counted |  | 87.5%  (P_30_) | 90.5%  (P_38_) |  | 99%  (P_37_) |  |  | 99%  (P_38_) |  |
| In vitro differentiation | At least 2 cells stain for | | | | | | | | | |
|  | sox-17 (endoderm) |  | **√** (P_31_) | **√** (P_43_) |  | **√**(P_41_) |  |  | **√**P_37_) |  |
|  | β-tubulin III **(**ectoderm) |  | **√**P_32_) | **√**P_46_) |  | **√** (P_31_) |  |  | **√**P_41_) |  |
|  | muscle actin (mesoderm) |  | **√**P_29_) | **√** (P_46_) |  | **√** (P_32_) |  |  | **√** (P_41_) |  |
| In Vivo:  Teratoma formation | Successful differentiation into teratomas consisting of the three primary germ layers |  | **√** (P_30_) | **√** (P_38_) |  | **√** (P_37_) |  |  | **√** (P_30_) |  |

Stock

1. * 1^o^ Banks characterized P_1-20_ after thawing; **2^o^ Banks characterized P_21+_ after thawing; NLT = Not Less Than; NMT = Not More Than [↑](#endnote-ref-2)
