# Supplementary material for: Derivation of Xeno-Free and GMP-Grade Human Embryonic Stem Cells – Platforms for Future Clinical Applications
Source: PLoS One. 2012 Jun 20;7(6):e35325. doi: 10.1371/journal.pone.0035325 (PMC3380026; doi:10.1371/journal.pone.0035325)
Supplement: Table S9 — hESC Safety Testing Results. (DOC) [file pone.0035325.s013.doc]

TABLE S9

HESC LINE ADVENTITIOUS VIRUS TEST RESULTS

A.

Liver Unit^

| **HAD-C 106** | **HAD-C 102** | **HAD-C 100** | **Cell Line** |
| --- | --- | --- | --- |
| Not detected | Not detected | Not detected | *HAV-RNA |
| Not detected | Not detected | Not detected | *HBV-DNA |
| Not detected | Not detected | Not detected | *HCV-RNA |

^HADASSAH MEDICAL CENTER TEST LAB RESULTS

*HAV-RNA detected by nested PCR (Liver Unit laboratory protocol)

*HBV-DNA detected by m2000 Realtime PCR commercial assay (ABBOTT Diagnostics)

*HCV-RNA detected by m2000 Realtime PCR commercial assay (ABBOTT Diagnostics)

Virology Test Labs^

| **HAD-C 106** | **HAD-C 102** | **HAD-C 100** | **Cell Line** |
| --- | --- | --- | --- |
|  | | | **Virus Tested** |
| Negative | Negative | Negative | HIV |
| Negative | Negative | Negative | CMV |
| Negative | Negative | Negative | EBV |
| Negative | Negative | Negative | HHV6 |

^HADASSAH MEDICAL CENTER TEST LAB RESULTS

B.

FDA-Approved Test Lab^#^

| **Test** | **1^o^ Reference Bank**  **HAD-C 100** |
| --- | --- |
| 28-day in vitro test | Pass |
| RT PCR-HIV 1/2 | Pass |
| RT PCR-HTLV 1/2 | Pass |
| RT PCR-HCV | Pass |
| RT PCR-HBV | Pass |
| RT PCR-EBV | Pass |
| RT PCR-CMV | Pass |
| RT-HHV6 | Pass |
| RT-HHV7 | Pass |
| RT-HHV8 | Pass |
| RT-B19, SV40 | Pass |
| RT-HAV | Pass |
| Ultrastructural examination (TEM) | Pass |

#Bioreliance Laboratories, Glasgow UK
