# Supplementary material for: Derivation of Xeno-Free and GMP-Grade Human Embryonic Stem Cells – Platforms for Future Clinical Applications
Source: PLoS One. 2012 Jun 20;7(6):e35325. doi: 10.1371/journal.pone.0035325 (PMC3380026; doi:10.1371/journal.pone.0035325)
Supplement: File S1 — Informed Consent. (DOC) [file pone.0035325.s015.doc]

**We, the undersigned:**

| First and last name (husband): First and last name (wife): |
| --- |
| I.D. No (husband): I.D. No (wife): |
| Address: Zip code: |

1. Hereby declare that we agree to participate in a clinical study, as specified in this document.

B. Hereby declare that we do not participate, at the time of signing this document, in any other clinical study, and that we are committed not to participate in any other clinical study during the whole period of this study.

C. Hereby declare that it has been explained to us by:

| Name of explaining investigator/deputy investigator: |
| --- |

- 1. that the senior investigators, **Prof Neri Laufer and Prof Benjamin Reubinoff** obtained from the Medical Institute Director approval to conduct the clinical study involving human subjects, in its implication in the Public Health Regulations (medical experiments involving human subjects) – 1980 (hereafter the Study):
  2. that the senior investigator **Prof. Neri Laufer**, Head of Department of Gynecology, has[[1]](#footnote-2) an affinity[[2]](#footnote-3) to the study initiator, **Prof. Benjamin Reubinoff,** **who is employed in his department.**
  3. that the clinical study is conducted on the subject: **human embryonic stem cells – a potential infinite source of cells for transplantation therapy.**
  4. that we are free to choose not to participate in the clinical study, and that we are free to discontinue at any time our participation in the study, all this without affecting our rights to receive the regular treatment.
  5. that it is guaranteed that our personal identity will be kept confidential by all those dealing with and involved in the study, and will not be made public in any publication, including scientific publications.
  6. that the medical institute has arranged appropriate insurance for the participants in the study.
  7. that if necessary, according to the doctor's decision, and subject to approval by the Institutional Helsinki Committee, we shall continue to receive the study preparation/device/accessory [[3]](#footnote-4) free of charge also after completion of the study, for a period not exceeding three years. During this period we shall continue to be under medical follow-up, if requested.
  8. that we were assured the possibility to get answers to questions raised by us, and the possibility to consult another body (e.g. family doctor, relatives etc), in regards to making the decision to participate in the clinical study and/or to continue our participation..
  9. that in any problem related to the study we may call **Prof.** **Laufer** **at Tel:**

**02-6776424**

10. In case of completing a questionnaire – we are aware that we are allowed not to answer all the questions in the questionnaire or part of them.

11. We are aware that we are not allowed to participate in more than one study at the same time.

12. We are aware that after signing this document we shall receive a copy of this Informed Consent.

13. We are aware that in clinical studies involving women in their fertile years, in case of pregnancy during the clinical study, the woman will consult (with her doctor) concerning the fate of pregnancy, including the possibility of termination of pregnancy.

D. We hereby declare that we received detailed information on the clinical study, and particularly on the following details, which are further specified in this form in the paragraph “detailed information on the study”:

1) The purpose of the study is to produce stem cells from IVF embryos. The stem cells will be produced under the conditions and according to the guidelines which enable possible use of these cells in the future for transplantation in a variety of diseases, such as heart failure, diabetes, Parkinson’s disease, Alzheimer’s disease, spinal cord damage and others. The stem cells which will be produced will be also used for the advancement of basic scientific research and for the development of new drugs.

2) The study would use only donated fertilized eggs and embryos which have been frozen for 5 years or more and which are surplus, and not needed to be returned to the uterus for producing another pregnancy. The donation of frozen embryos will be possible only by free informed consent of both partners, i.e. the woman donating the eggs and the husband donating the sperms. A free informed consent of both partners (wife and husband) will also be required in the event that the sperm was donated to the couple by an anonymous donor. In case the embryo was produced by means of sperm from an anonymous donor donated to an unmarried woman, an informed consent of the donating woman only will be required. The donated embryos will be thawed and cultured until the stage of development of a blastocyst required for producing the stem cells (which occurs 5-7 days after fertilization). The embryos will not continue to exist after the production of the stem cells. In no case will the embryos be used for the fertilization of a woman.

3) The produced stem cells may be kept frozen and used for research and transplantations for years. The use of the stem cells in basic scientific and clinical research will be for the good of the public in accordance with the appropriate ethical rules for research in tissues from human origin, and subject to approval by the appropriate ethics committees and approval by the authorities regulating the production of drugs.

4) In order to advance the research in the field of stem cells and their use in transplantation, the embryonic stem cells produced will be supplied to researchers and doctors throughout the world, subject to their commitment to use these cells in basic research in accordance with the ethical rules appropriate for research with a tissue from human origin, and in transplantation, subject to approval by the appropriate ethics committees and by the authorities regulating the production of drugs. You will not be given any information and you will not be involved or influential in any way in regard to these transplantations.

5) Since the goal is to use the stem cells for transplantation in patients, you will undergo medical examinations similar to those of blood donors, including: an interview to find out about your personal and familial medical history, as well as blood tests. Your blood samples will be kept frozen in case further tests will have to be done with your blood in the future. Further examinations will be performed only after receiving a detailed explanation on the type and significance of those examinations from an appropriate counselor (genetic counselor for genetic tests) and subject to your consent to this. The results of the blood tests will be given to you.

6) It is hereby clarified that the embryo donors will not receive any financial or other compensation for their contribution of embryos to the study. The study is not intended to bring results which would benefit the embryo donors directly from a medical or any other point of view. It is possible that the stem cells derived from the donated embryos, the cells developed from them, or the results of the study performed with the stem cells would have a commercial potential. It is hereby clarified that in such an event the embryo donors will not benefit financially or in any other way.

**Detailed information on the study**

**Production of human embryonic stem cells lines – a potential source of cells for transplantation** therapy

**1) Objectives of the study and introduction:**

Many diseases are caused due to impaired function of cells or organs. In many cases, it is impossible to sufficiently improve the impaired function by drugs or surgery, and the only way to cure the patient is by transplantation of cells or organs. The lack of organs, the difficulty in getting them, and the restrictions related to rejection of the transplant all limit the use of transplantation organs.

A scientific breakthrough which may solve, at least partially, the lack of tissues for transplantation, is the production of human embryonic stem cells. The stem cells are produced from surplus IVF embryos donated by couples who are not interested in continued use of these embryos for the treatment of infertility. The stem cells are produced from IVF embryos on the fifth or sixth day after fertilization. The embryo can no longer exist after the production of the stem cells.

The embryonic stem cells are characterized by their ability to multiply indefinitely, and also by their ability to be transformed into any type of human cell, such as cardiac cells, neurons, blood cells and others. Therefore, these cells may be used in the future as an indefinite source of cells for transplantation in a wide variety of diseases, such as cardiac failure, diabetes, Parkinson’s disease, Alzheimer’s disease, spinal cord damage and others. Moreover, the study of these cells facilitates research on the early development in humans, which may contribute to the development of new drugs. The human embryonic stem cells can improve our understanding of various diseases, such as congenital defects, miscarriages and tumors. In addition, these cells can be used for checking the effectiveness and safety of new drugs.

Human embryonic stem cells have been successfully produced by us, as well as by several other groups of scientists throughout the world. The stem cells available today were produced for research purposes and it is unlikely that they are suitable for treatment of patients.

The purpose of this study is to produce new embryonic stem cells under the conditions and in accordance with the guidelines which will enable to use these cells for transplantation in the future. The new embryonic stem cells to be produced will be used also for the advancement of research and the development of drugs as specified above, in addition to their possible use in transplantation. The use of the stem cells will be strictly monitored for the good of the public and according to the accepted ethical rules.

**2) Methods**

The study protocol and the explanation form in the framework of the application for donation of embryos were prepared according to the guidelines of the Report of the Consulting Committee to the Subject of Bioethics of the National Israeli Academy of Sciences, and according to the ethical guidelines published by the American National Institute of Health (NIH) in 1999, with regards to the production of lines of human embryonic stem cells. The study protocol and the wording of the Informed Consent were approved by the Superior Helsinki Committee for Genetic Studies of the Israeli Ministry of Health.

**The candidates for donation of embryos are couples or women whose egg was fertilized by the sperm of an anonymous donor.**

1) Only the donation of fertilized eggs and surplus embryos which have been frozen for 5 years or more, and are not intended to be returned to the uterus for another pregnancy, will be accepted for the study. The donation of the frozen embryos will only be performed subject to free informed consent of the couple, i.e. the wife donating the eggs and the husband donating the sperm. Free informed consent by the couple (the husband and wife) will also be required in case the sperm was donated to the couple by an anonymous donor. In case the embryo of an unmarried woman was produced by the sperm of an anonymous donor, the informed consent will be requested only from the donating woman.

2) It is hereby clarified that it is your right to request the continued storage of the fertilized eggs up to an inclusive period of 10 years.

3) The embryos donated by you will be used only for the production of embryonic stem cells, in accordance with the protocols proven effective for this purpose. The production of stem cells is successful in part of the embryos and not in all of them.

4) It is hereby emphasized that the embryos donated by you will not be returned to the uterus of another woman.

5) The embryos donated by you will be thawed and cultured up to the stage of development of a blastocyst, which is necessary for the production of stem cells (which appears 5-7 days after fertilization), and not beyond that, and in no case more than 14 days from the day of fertilization. The embryos will not continue to exist after the production of the stem cells.

6) The research in embryos and in the stem cells produced from them will be conducted according to the ethical rules for research on tissues from human origin. The use of cells for transplantation will be conducted only subject to the approval by ethics committees and by the regulating medical authorities for the production of drugs.

7) The derived stem cells, as well as (daughter) cells developed from them, may be kept frozen for years and used for research and treatment.

8) The purpose of this project is to advance the research in the field of human embryonic stem cells and their use in transplantation throughout the world. Embryonic stem cells produced in the framework of this study will be provided to researchers throughout the world, subject to their commitment that the research on stem cells will be conducted according to the rules of ethics appropriate to research in a tissue from human origin. The stem cells will be given for transplantation and clinical research throughout the world, only subject to approval by ethics committees and by authorities regulating the manufacture of drugs. The people receiving the lines of cells throughout the world will not give them to others without a clear approval in writing from the producers of these lines or their representative. The transfer of cells to others will be possible only subject to the conditions specified above in this paragraph.

9) You will not receive any information on the findings of this study or the results of tests conducted with the donated embryos or with the stem cells produced from them.

10) If stem cells will be produced from the embryos donated by you, the stem cells or cells developed from them may be used in research related to transplantation in human beings, as well as in transplantation treatments in other human beings. You will not receive any information and you will not have any involvement or influence on these transplantations.

11) Any sign identifying the relation of the embryos to you will be removed before the production of the embryonic stem cells. In the course of the process of producing the cell lines and the research done with them there will be no sign identifying the cells with you.

12) The lines of cells produced from the embryos donated by you will receive a code name. The information identifying you with the code will be confidential and kept only in a safe at Hadassah Hospital Ein-Karem. It is possible that a new disease which is unknown today will be discovered, which would make it necessary to apply to you for further details related to your medical history, or you may be asked to give blood for another test in order to exclude possible transmission of this disease to the transplanted patients. The probability that you will be approached is low. Such an approach will become possible and done only by approval of the Head of Gynecology at Hadassah Ein Karem. Since it would be possible to find you under the circumstances and subject to the process explained in this paragraph, according to the law of genetic information, the research in the samples donated by you is defined as research with identified samples.

13) Since the target is to use the stem cells produced from the embryos donated by you in the treatment of patients, the U.S. medical authorities require that the donors (husband and wife) will undergo medical examinations similar to those of blood donors, including:

1) An interview to clarify personal and family medical history. The results of blood tests for infectious diseases which were done in the course of IVF treatment will be checked and documented.

Tests for blood type and infectious diseases may include hepatitis A, B & C, rubella, herpes, B19 Parvovirus, HGV, TTV, SEN Family, CMV, as well as venereal diseases including gonorrhea, chlamydia and syphilis. Not necessarily all the above listed diseases will be checked.

2) In accordance with the U.S. medical authorities guidelines, your blood samples will be kept frozen in the event it would be necessary to conduct further tests on your blood in the future. The probability of this happening is low, but it is possible that a new disease which is unknown today will be discovered in the future, such as an inherited or infectious disease, or that progress will be made in understanding the cause for a known disease. In such an event, it may be that in order to exclude with certainty the transmission of the disease by the stem cells and its possible transmission to transplanted patients, checking of the stem cells would not be sufficient, and it would be necessary to conduct further tests on your blood samples which are kept frozen. A further test on your blood samples in the future will be done only after approaching you again, and after you receive a detailed explanation on the type of test and its implication from an appropriate counselor (genetic counselor for genetic test) and subject to your consent to this. As said in paragraph 12, you may be approached only with the approval of the Head of Gynecology at Hadassah Ein Karem.

3) You will receive the results of the blood tests.

**3) The expected duration of time (treatment and follow-up):**

One to two hours in which you will receive an explanation, have a medical interview, and blood will be drawn from you.

**4) The expected benefit to the participant:**

1) It is hereby clarified that you will not receive any financial or other benefit for your donation of embryos to the study.

2) It is hereby clarified that the study is not intended to bring results which would benefit you directly, medically or otherwise.

3) It is possible that the stem cells produced from the embryos donated by you, the cells developed from them or the results of the study performed on the stem cells, would have a commercial potential. It is hereby clarified that in such an event you will not have a financial or any other benefit.

**5) Potential risks:** There are no risks.

**6) Potential discomfort:** The discomfort involved in drawing blood from the vein.

**7) Other relevant information:**

Before you give your consent to the donation of embryos for this study, we wish to emphasize the following points:

1) Your agreement or disagreement to participate in the study and to donate the embryos would not have any implication or influence on the medical treatment you receive.

2) Ethical considerations supporting the production of human stem cells from surplus IVF embryos are:

A) The alternative to donation is to destroy these embryos or keep them frozen forever. It is hereby clarified that the current regulations concerning IVF allow the destruction of frozen embryos after 5 years, unless a different instruction is given by the parents.

B) The withdrawal and growing of cells in a tissue culture from donated embryos does not mean disrespect to the human embryo in general, since diagnosis of embryos prior to their implantation in the uterus may be judged according to the same ethical criterion. Such pre-pregnancy diagnosis involves the selection of embryos and the destruction of other embryos. The pre-pregnancy method for detection of genetic diseases is accepted in the medical practice in Israel and in many other countries. In addition, other forms of research on human embryos are used today, such as studies intended to improve methods of embryo culture in the framework of IVF treatments. These studies involve the growing in test-tubes of fertilized eggs for about 6 days until they reach the blastocyst stage, in order to select, from the total number of embryos, those with the highest potential for implantation, and discard the rest.

**8) Attachment of the company’s explanatory pages to the patient:**

Attached are, in addition, explanatory pages to the patient provided by the company: yes/no? **No.**

E. We hereby declare that we gave our above consent out of our free will and that we understood all the above said. We also received a copy of the Informed Consent Form and the Information Sheet attached to this form (if available).

F. By signing this Consent Form, we give our permission to the initiator of the clinical study, the Institutional Helsinki Committee and the Ministry of Health, free access to our medical records, in order to verify the clinical study methods and the clinical data. This access to our medical information will be done confidentially, in accordance with the laws and procedures of confidentiality.

G. I hereby declare that I know and agree that the information related to my participation in the medical experiment will be submitted to my doctor at the Sick Fund in which I am insured. I am aware that according to the Arrangements Law, 2004, this information will not be used for any purpose, except for medical treatment and follow-up.

| Names of participants in the clinical study | Signatures of participants in the study | Date |
| --- | --- | --- |
| (husband)  (wife) |  |  |

If necessary^2^ :

| Name of independent witness | Signature of witness | date |  |
| --- | --- | --- | --- |
|  |  |  |  |

**Investigator/Deputy Investigator declaration:** The above consent was received by me, after I have explained to the participant in the clinical study all the above said, and I made sure that all my explanations were understood by him/her.

| Name of explaining Investigator / Deputy Investigator: | Signature | Date |
| --- | --- | --- |

^2^ In case the participant in the study or his legal representative is not able to read this Informed Consent Form, an independent witness should be present during the explanation on the nature of the clinical study. After the participant or his legal representative gave his oral consent to participate in the study, the witness will sign the Consent Form and indicate the date of his signature.

1. delete the unnecessary. [↑](#footnote-ref-2)
2. affinity – attachment of employment with salary, contractorship or any other way, or commercial or business attachment, or family or personal attachment, and any other attachment which may raise suspicion of conflict of interest or dependence, except for reimbursement or payment for participation in the committees according to procedure.

   * The informed consent document shown here was modified from Hadassah's typical format for informed consent documents utilized in clinical trials. [↑](#footnote-ref-3)
3. delete the unnecessary. [↑](#footnote-ref-4)
