# Supplementary material for: Derivation of Xeno-Free and GMP-Grade Human Embryonic Stem Cells – Platforms for Future Clinical Applications
Source: PLoS One. 2012 Jun 20;7(6):e35325. doi: 10.1371/journal.pone.0035325 (PMC3380026; doi:10.1371/journal.pone.0035325)
Supplement: File S2 — Clinical Site Signature Log. (DOC) [file pone.0035325.s016.doc]

# FILE S2

# CLINICAL SITE SIGNATURE LOG

# Page ___ of ____

THE DERIVATION OF NEW HUMAN EMBRYONIC STEM CELL LINES FOR CLINICAL USE

STUDY TITLE:

SITE NAME (Check one): Hadassah, Ein Kerem Hadassah, Mt. Scopus

| **Title** | **Printed Name** | **Signature** | **Initials** | **Degree (Dr., Prof., Nurse, Mr. or Ms.)** | **Date** |
| --- | --- | --- | --- | --- | --- |
|  |  |  |  |  |  |
|  |  |  |  |  |  |
|  |  |  |  |  |  |
|  |  |  |  |  |  |
|  |  |  |  |  |  |
|  |  |  |  |  |  |
|  |  |  |  |  |  |
|  |  |  |  |  |  |
|  |  |  |  |  |  |
|  |  |  |  |  |  |
