# Supplementary material for: Derivation of Xeno-Free and GMP-Grade Human Embryonic Stem Cells – Platforms for Future Clinical Applications
Source: PLoS One. 2012 Jun 20;7(6):e35325. doi: 10.1371/journal.pone.0035325 (PMC3380026; doi:10.1371/journal.pone.0035325)
Supplement: File S4 — Monitor Sign-In Log. (DOC) [file pone.0035325.s018.doc]

# File S4

# MONITOR SIGN-IN LOG

# Page ___ of ____

THE DERIVATION OF NEW HUMAN EMBRYONIC STEM CELL LINES FOR CLINICAL USE

STUDY TITLE:

| **DATE** | **TIME OF MONITOR SIGN-IN** | **MONITOR SIGNATURE** | **STUDY/NURSE COORDINATOR SIGNATURE** | **TIME OF MONITOR SIGN-OUT** | **MONITOR SIGNATURE** |
| --- | --- | --- | --- | --- | --- |
|  |  |  |  |  |  |
|  |  |  |  |  |  |
|  |  |  |  |  |  |
|  |  |  |  |  |  |
|  |  |  |  |  |  |
|  |  |  |  |  |  |
|  |  |  |  |  |  |
|  |  |  |  |  |  |
|  |  |  |  |  |  |
|  |  |  |  |  |  |
