# Supplementary material for: Derivation of Xeno-Free and GMP-Grade Human Embryonic Stem Cells – Platforms for Future Clinical Applications
Source: PLoS One. 2012 Jun 20;7(6):e35325. doi: 10.1371/journal.pone.0035325 (PMC3380026; doi:10.1371/journal.pone.0035325)
Supplement: File S5 — Additional Comments Log. (DOC) [file pone.0035325.s019.doc]

# File S5

# ADDITIONAL COMMENTS/PHYSICIAN NOTES

# Page ___ of ____

THE DERIVATION OF NEW HUMAN EMBRYONIC STEM CELL LINES FOR CLINICAL USE

STUDY TITLE:

| **PERTAINS TO FORM #** | **COMMENTS/NOTES** | **SIGNATURE/DATE** |
| --- | --- | --- |
|  |  |  |
|  |  |  |
|  |  |  |

NOTES: ______________________________________________________________________________________________________________________________________________________________________________________________________________________________________________________________________________________________________________________
