# Supplementary material for: Derivation of Xeno-Free and GMP-Grade Human Embryonic Stem Cells – Platforms for Future Clinical Applications
Source: PLoS One. 2012 Jun 20;7(6):e35325. doi: 10.1371/journal.pone.0035325 (PMC3380026; doi:10.1371/journal.pone.0035325)
Supplement: File S6 — Telephone Call Report Form. (DOC) [file pone.0035325.s020.doc]

# File S6

# TELEPHONE CALL REPORT FORM

# Page ___ of ____

THE DERIVATION OF NEW HUMAN EMBRYONIC STEM CELL LINES FOR CLINICAL USE

STUDY TITLE:

**NOTE: COMPLETE THIS FORM WHENEVER DONOR(S) CONTACT THE RESEASRCH/MEDICAL STAFF**

| **DATE** | **PERSON CALLED** | **NOTES/DISCUSSION DETAILS** | **SIGNATURE** |
| --- | --- | --- | --- |
|  |  |  |  |
|  |  |  |  |
|  |  |  |  |
|  |  |  |  |

NOTES: ____________________________________________________________________________________________________________________________________________________________________________________________________________________________________________________________________________________________________________________________________________________________________________________
