# Supplementary material for: Derivation of Xeno-Free and GMP-Grade Human Embryonic Stem Cells – Platforms for Future Clinical Applications
Source: PLoS One. 2012 Jun 20;7(6):e35325. doi: 10.1371/journal.pone.0035325 (PMC3380026; doi:10.1371/journal.pone.0035325)
Supplement: File S7 — Clinical Site Signature Log, Foreign. (DOC) [file pone.0035325.s021.doc]

# File S7

# CLINICAL SITE SIGNATURE LOG – FOREIGN SIGNATURES

# Page ___ of ____

THE DERIVATION OF NEW HUMAN EMBRYONIC STEM CELL LINES FOR CLINICAL USE

STUDY TITLE:

SITE NAME (Check one): Hadassah, Ein Kerem Hadassah, Mt. Scopus

| **Title** | **Printed Name** | **Signature in English** | **Signature in Hebrew** | **Signature in any other Foreign Language** | **Date** |
| --- | --- | --- | --- | --- | --- |
|  |  |  |  |  |  |
|  |  |  |  |  |  |
|  |  |  |  |  |  |
|  |  |  |  |  |  |
|  |  |  |  |  |  |
|  |  |  |  |  |  |
|  |  |  |  |  |  |
|  |  |  |  |  |  |
|  |  |  |  |  |  |
|  |  |  |  |  |  |
|  |  |  |  |  |  |
