# Supplementary material for: Derivation of Xeno-Free and GMP-Grade Human Embryonic Stem Cells – Platforms for Future Clinical Applications
Source: PLoS One. 2012 Jun 20;7(6):e35325. doi: 10.1371/journal.pone.0035325 (PMC3380026; doi:10.1371/journal.pone.0035325)
Supplement: File S8 — Study Completion/Withdrawal/Exclusion Form. (DOC) [file pone.0035325.s022.doc]

# File S8

# STUDY COMPLETION/WITHDRAWAL/EXCLUSION FORM

THE DERIVATION OF NEW HUMAN EMBRYONIC STEM CELL LINES FOR CLINICAL USE

STUDY TITLE:

**Study Completion/Withdrawal/Exclusion**

Date of Study completion, withdrawal, or exclusion:

dd mm yy

**Specify action taken**:

Donor completed the study as planned

Adverse event (complete the Adverse Event Form, form 3.01)

Protocol noncompliance (protocol deviation; describe the deviation on the Additional Comments form, form 1.04)

Donor withdrew from study

Donor completed initial interview with study coordinators, but was not available for follow-up questions (i.e. lost to follow-up)

Donor excluded from study (describe reason _____________________

_________________________________________________________

_________________________________________________________)

Medical Director’s discretion

Other ____________________________________________________

Staff member completing the form: _______________________

Staff position: _____________________

Date completed: ___________________
