# Supplementary material for: Derivation of Xeno-Free and GMP-Grade Human Embryonic Stem Cells – Platforms for Future Clinical Applications
Source: PLoS One. 2012 Jun 20;7(6):e35325. doi: 10.1371/journal.pone.0035325 (PMC3380026; doi:10.1371/journal.pone.0035325)
Supplement: File S9 — Pre-Trial Screening Log. (DOC) [file pone.0035325.s023.doc]

# PRE-TRIAL SCREENING INFORMATION

# Page ___ of ____

THE DERIVATION OF NEW HUMAN EMBRYONIC STEM CELL LINES FOR CLINICAL USE

STUDY TITLE:

SITE NAME (Check one): Hadassah, Ein Kerem Hadassah, Mt. Scopus

| **#** | **Donor Names**  **(Last, First)** | **Teudat Zehuts**  **Of Donors** | **Phone Numbers** | **Hadassah File #** | **Date(s)**  **Of Oocyte**  **Pickup** | **Number/Quality**  **Of Embryos** | | | | | | | | **Contact**  **Date/**  **Time** | **Interested**  **In Donation?** | | **Interview**  **Date/Time** |
| --- | --- | --- | --- | --- | --- | --- | --- | --- | --- | --- | --- | --- | --- | --- | --- | --- | --- |
|  |  |  |  |  |  | **A** | **A/B** | **B** | **B/C** | **C** | **C/D** | **D.** | **2PN** |  | **Y** | **N** |  |
|  | **F** | **F** | Home | (1) | (1) |  |  |  |  |  |  |  |  |  |  |  |  |
|  |  |  | Work |  |  |  |  |  |  |  |  |  |  |  |  |  |  |
|  |  |  | Mobile | (2) | (2) |  |  |  |  |  |  |  |  |  |  |  |  |
|  | **F** | **F** | Home | (1) | (1) |  |  |  |  |  |  |  |  |  |  |  |  |
|  |  |  | Work |  |  |  |  |  |  |  |  |  |  |  |  |  |  |
|  |  |  | Mobile | (2) | (2) |  |  |  |  |  |  |  |  |  |  |  |  |
|  | **F** | **F** | Home | (1) | (1) |  |  |  |  |  |  |  |  |  |  |  |  |
|  |  |  | Work |  |  |  |  |  |  |  |  |  |  |  |  |  |  |
|  |  |  | Mobile | (2) | (2) |  |  |  |  |  |  |  |  |  |  |  |  |
|  | **F** | **F** | Home | (1) | (1) |  |  |  |  |  |  |  |  |  |  |  |  |
|  |  |  | Work |  |  |  |  |  |  |  |  |  |  |  |  |  |  |
|  |  |  | Mobile | (2) | (2) |  |  |  |  |  |  |  |  |  |  |  |  |
|  | **F** | **F** | Home | (1) | (1) |  |  |  |  |  |  |  |  |  |  |  |  |
|  |  |  | Work |  |  |  |  |  |  |  |  |  |  |  |  |  |  |
|  |  |  | Mobile | (2) | (2) |  |  |  |  |  |  |  |  |  |  |  |  |
