# Supplementary material for: Derivation of Xeno-Free and GMP-Grade Human Embryonic Stem Cells – Platforms for Future Clinical Applications
Source: PLoS One. 2012 Jun 20;7(6):e35325. doi: 10.1371/journal.pone.0035325 (PMC3380026; doi:10.1371/journal.pone.0035325)
Supplement: File S10 — Enrollment, Protocol, and Informed Consent Log. (DOC) [file pone.0035325.s024.doc]

# ENROLLMENT, PROTOCOL, AND INFORMED CONSENT LOG

# Page ___ of ____

THE DERIVATION OF NEW HUMAN EMBRYONIC STEM CELL LINES FOR CLINICAL USE

STUDY TITLE:

| **COUPLE**  **#** | **Date Enrolled** | **Donor’s Names**  **(Last, First)** Female/Male | **Donor’s Initials** | **Teudat Zehuts of Donors** | Hadassah **IVF Code #** | Study Code Number | **Donors Read Protocol?**  **Y/N** | **Donors Received Explanation**  **And Opportunity to Ask Questions?**  **Y/N** | **Informed**  **Consent Obtained?**  **See Informed Consent CRF** |
| --- | --- | --- | --- | --- | --- | --- | --- | --- | --- |
| 1 |  | F: | F: | F: |  |  | F: | F: | F: |
|  |  | M: | M: | M: |  |  | M: | M: | M: |
| 2 |  | F: | F: | F: |  |  | F: | F: | F: |
|  |  | M: | M: | M: |  |  | M: | M: | M: |
| 3 |  | F: | F: | F: |  |  | F: | F: | F: |
|  |  | M: | M: | M: |  |  | M: | M: | M: |
| 4 |  | F: | F: | F: |  |  | F: | F: | F: |
|  |  | M: | M: | M: |  |  | M: | M: | M: |
| 5 |  | F: | F: | F: |  |  | F: | F: | F: |
|  |  | M: | M: | M: |  |  | M: | M: | M: |
| 6 |  | F: | F: | F: |  |  | F: | F: | F: |
|  |  | M: | M: | M: |  |  | M: | M: | M: |
