# Supplementary material for: Derivation of Xeno-Free and GMP-Grade Human Embryonic Stem Cells – Platforms for Future Clinical Applications
Source: PLoS One. 2012 Jun 20;7(6):e35325. doi: 10.1371/journal.pone.0035325 (PMC3380026; doi:10.1371/journal.pone.0035325)
Supplement: File S11 — Source Data. (DOC) [file pone.0035325.s025.doc]

# MEDICAL HISTORY SOURCE DATA

NOTE: COMPLETE ONE FORM FOR EACH MALE AND FEMALE COUPLE
