# Supplementary material for: Derivation of Xeno-Free and GMP-Grade Human Embryonic Stem Cells – Platforms for Future Clinical Applications
Source: PLoS One. 2012 Jun 20;7(6):e35325. doi: 10.1371/journal.pone.0035325 (PMC3380026; doi:10.1371/journal.pone.0035325)
Supplement: File S12 — Clinical Results Report Form. (DOC) [file pone.0035325.s026.doc]

# CLINICAL LABORATORY RESULTS FORM

NOTE: COMPLETE ONE LABORATORY TESTS FORM FOR EACH MALE AND FEMALE DONOR

TO: Derivation of New Embryonic Stem Cell Lines Research Group

**The following tests were performed by our Labs**

CHECK ONE: Donor: M F

Sample: Blood Swab

Tests Performed by: _________________________________________ Date: ______________

| **Test to Perform** | **AML Code** | **Performed** | **Results (Circle One)** |
| --- | --- | --- | --- |
| Chlamydia Ab - IgA  IgG | 3405 |  | Positive/ Negative  Positive/ Negative |
| Hbs Ag | 3411 |  | Positive/ Negative |
| HCab | 3406 |  | Positive/ Negative |
| Anti-HB_core_ | 3407 |  | Positive/ Negative |
| HIV 1 + 2 Ab | 3403 |  | Positive/ Negative  Positive/ Negative |
| HTLV 1 + 2 Ab | 3404 |  | Positive/ Negative  Positive/ Negative |
| RPR or VDRL | 3401 | (indicate  which) | Reactive/Non-Reactive  Reactive/Non-Reactive |
| Rubella IgM  IgG | 3408 |  | Positive/ Negative  Positive/ Negative |
| Gonorrhea  (Swab) | 3402 |  | Positive/ Negative |
| CMV IgM  IgG | 3409 |  | Positive/ Negative  Positive/ Negative |
| EBV IgM  IgG  EBNA | 3412 |  | Positive/ Negative  Positive/ Negative  Positive/ Negative |
| Blood Type | 700 |  |  |
| CBC | 100 |  |  |

Tests Recorded by: ___________________________________ Date: ________________________

Attach original test results to this Form.

**Results**
