# Supplementary material for: Derivation of Xeno-Free and GMP-Grade Human Embryonic Stem Cells – Platforms for Future Clinical Applications
Source: PLoS One. 2012 Jun 20;7(6):e35325. doi: 10.1371/journal.pone.0035325 (PMC3380026; doi:10.1371/journal.pone.0035325)
Supplement: File S13 — Blood Sample Archiving Form. (DOC) [file pone.0035325.s027.doc]

# BLOOD SAMPLE ARCHIVING FORM

NOTE: COMPLETE ONE BLOOD SAMPLE ARCHIVING FORM FOR EACH DONOR COUPLE

DATE BLOOD SAMPLES TAKEN: ______________

BY WHOM: ________________________________

LOCATION OF ARCHIVED BLOOD: ________________________

ARCHIVED BLOOD IS STORED IN A LOCKED FACILITY? YES NO

NUMBER OF VIALS TAKEN FOR DONOR MALE: DONOR FEMALE:

ARCHIVED BLOOD IS LABELED WITH DONOR NUMBER AND EITHER “M” OR “F”:

YES NO

TO BE COMPLETED ONLY IF ARCHIVED BLOOD IS NEEDED FOR DONOR TESTING:

ARCHIVED BLOOD IS BEING SENT FOR THE FOLLOWING TESTS (CHECK ALL THAT APPLY):

| **Tests to Perform** | **Donor Male** | **Donor Female** |
| --- | --- | --- |
| Chlamydia Ab - IgA  IgG |  |  |
| Hbs Ag |  |  |
| HCab |  |  |
| Anti-HB_core_ |  |  |
| HIV 1 Ab  2 Ab |  |  |
| HTLV 1 Ab  2 Ab |  |  |
| RPR or  VDRL |  |  |
| Rubella IgM  IgG |  |  |
| Gonorrhea  (Swab) |  |  |
| CMV IgM  IgG |  |  |
| EBV IgM  IgG  EBNA |  |  |
| Blood Type |  |  |
| CBC |  |  |
