# Supplementary material for: Derivation of Xeno-Free and GMP-Grade Human Embryonic Stem Cells – Platforms for Future Clinical Applications
Source: PLoS One. 2012 Jun 20;7(6):e35325. doi: 10.1371/journal.pone.0035325 (PMC3380026; doi:10.1371/journal.pone.0035325)
Supplement: File S14 — Blood Archiving Form. (DOC) [file pone.0035325.s028.doc]

# BLOOD ARCHIVING FORM

# Page ___ of ____

THE DERIVATION OF NEW HUMAN EMBRYONIC STEM CELL LINES FOR CLINICAL USE

STUDY TITLE:

| **ARCHIVING LOG** | | | | |
| --- | --- | --- | --- | --- |
| **Date Archived** | **Donor Number** | **Archived Serum** | | **Location** |
|  |  | **Male** | **Female** |  |
|  |  |  |  |  |
|  |  |  |  |  |
|  |  |  |  |  |
|  |  |  |  |  |
|  |  |  |  |  |
|  |  |  |  |  |
|  |  |  |  |  |
|  |  |  |  |  |
|  |  |  |  |  |
|  |  |  |  |  |
|  |  |  |  |  |
