# Supplementary material for: Derivation of Xeno-Free and GMP-Grade Human Embryonic Stem Cells – Platforms for Future Clinical Applications
Source: PLoS One. 2012 Jun 20;7(6):e35325. doi: 10.1371/journal.pone.0035325 (PMC3380026; doi:10.1371/journal.pone.0035325)
Supplement: File S15 — Adverse Events Form. (DOC) [file pone.0035325.s029.doc]

# ADVERSE EVENTS FORM

THE DERIVATION OF NEW HUMAN EMBRYONIC STEM CELL LINES FOR CLINICAL USE

USED FOR REPORTING ADVERSE CONSEQUENCES TO HUMANS PARTICIPATING IN RESEARCH

**When to use this form**: The Principal Investigator should complete and sign this form and submit it with related attachments (if any) for any event that falls into either Category A or Category B, below:

**Category A - Any *Serious Adverse Event* that Occurs within 48 hours of Participation in the Research.** Serious adverse events are those resulting in death, a life-threatening experience, hospitalization or prolongation of existing hospitalization, a persistent or significant disability or capacity, or a congenital anomaly or birth defect. Every serious adverse event must be reported on this form, even if the event does not appear to be associated with the research protocol. If applicable, also file an FDA Adverse Event Report (found at <http://www.fda.gov/cder/aers/>). In addition, the IRB Office of Hadassah should be notified within 24 hours of discovery of any serious adverse event.

**Category B – Any Event for which *All Three of the Following* are True:**

**(1) Subject or Risks to Subject or Others Adversely Affected** An event or outcome has occurred that has resulted *in harm* to the subject, has affected the subject *detrimentally*, has *worsened* as a result of their participation, or that has resulted in *increased risk to the subject* or to others, whether or not the risk has actually resulted in harm (for example, misplacing a subject’s research records would constitute an increased risk event that should be reported).

**(2) Unexpected Event** The event or outcome was not described as a *risk* of participation in the research, or, though described as a risk, the event or outcome has occurred with *unexpected severity or frequency*.

**(3) Possibly, Probably, or Definitely Related Event** The event or outcome was definitely related to participation in the research or it’s *reasonable to conclude* that the event or outcome was related to participation, or it’s possible the event or outcome was related but not enough information is available at this time to assess the likelihood of this possibility.

**1. DATE OF EVENT:**

**DATE OF EVENT’S DISCOVERY BY RESEARCH PERSONNEL:**

**DATE OF THIS REPORT:**

**2**. **REPORT TYPE**: Initial Follow-Up on Previously Reported Event

**3**. **RESPONSIBLE PROJECT INVESTIGATOR (PI)**:

Last Name: First Name: Title:

Department: Office Address: City: Country: Zip Code:

Phone: FAX: Pelephone:

E-mail:

**4**. **PROJECT TITLE**:  **IRB PROTOCOL NUMBER:**

**5.** **RESEARCH SITE**:

**6**. **RESEARCH PERSONNEL**: Who was present when the incident (or consequent events) was (were) discovered?

**7**. **EVENT TYPE** (See “When to Use this Form”) Category A – Serious Adverse Event

Category B – Other Unanticipated Event Adversely Affecting Subject or Others

**8**. **SUBJECT INFORMATION**

Known pre-existing condition(s), if any: Age: Male Female

**9**. **DESCRIBE THE EVENT (CHECK ALL THAT APPLY):**

Life threatening experience Psychological harm or injury occurred

Required emergency treatment Social harm or injury occurred

Required transport to hospital Economic harm occurred

Required hospitalization Breach of confidentiality occurred

Prolonged current hospitalization Risk of psychological, social, or economic harm or injury increased

Persistent or significant disability/incapacity Risk of confidentiality breach increased

Congenital anomaly/birth defect Related to donation

New disease or problem Other

Death – underlying or progressive disease

Death – research related

**10. PROVIDE A BRIEF NARRATIVE OF THE EVENT**:

**11. DESCRIBE ANY AND ALL STEPS AND ACTIONS TAKEN IN RESPONSE TO THE INCIDENT OR TO RESOLVE THE ISSUE:**

**12. WHAT WAS THE SUBJECT’S PARTICIPATION LEVEL AFTER THE EVENT? (Mark all that apply)**

Subject stopped research participation Subject had already completed research

Subject continued research participation Subject withdrew from further participation

Investigator withdrew subject from further participation Other

**13. PROGNOSIS** Describe the subject’s prognosis

**14. HAS ANY PREVIOUS RESEARCH PRODUCED THIS TYPE OF EVENT OR OUCOME?** YES NO

If yes, describe and reference previous report(s):

**15. MARK ONE IN BOTH A AND B TO CATEGORIZE THIS VENT ACCORDING TO THE PI”S JUDGEMENT:**

15A. Expected Unexpected 15B. Serious Not serious

1. **RELATION TO RESEARCH** In the PI’s judgment, was there a relationship between the event and the research?

Definitely – clearly related to the research

Probably – likely related to the research

Possibly – may be related to the research but information not yet available to assess the likelihood of this

Probably Not – doubtfully related to the research

Definitely Not – clearly not related to the research

**17. RELATION TO STATED RISKS** In the PI’s judgment, are the probability, magnitude, and reversibility of this event consistent with the risk information in the research protocol, IRB application, and informed consent document previously reviewed and approved by the IRB? Yes No

If Yes, attach copies of the protocol, application, and consent document with relevant sections highlighted.

Attached Will Follow

**18. REVISIONS NEEDED?** In the PI’s judgment, should the research protocol, application, or consent form be revised? Yes No

If yes, describe the change below and attach the changed protocol, application, or consent document with relevant sections highlighted.

Check all that apply: Changed protocol application informed consent

Description of changed document:

Changed document attached will follow

**19. NOTIFICATION OF SUBJECTS AND OTHERS** In the PI’s judgment, which of the following subject groups or their legally authorized representatives should be notified? Check all that apply.

New subjects Currently enrolled subjects

Subjects that have completed the research None

**20. RE-CONSENT/ASSENT** In the PI’s judgment, is it necessary to obtain anew the consent or assent of subjects or legally authorized representatives who have already given their consent or assent to participate?

Yes No

**21. AFFECT ON RESEARCH** In the PI’s judgment, should the research

**continue as planned** with no changes to the research protocol or consent process?

**continue with changes** to the research protocol or consent process, as previously noted on this form?

**suspend new subject enrollment** until the event is further assessed?

**be terminated** (stopped completely), with all subjects removed from research?

22. REPORTS FILED To whom has the event been reported? Mark all that apply and attach report(s)

**Report Filed With Date Reported Report(s)**

Research sponsor/coordinating site Attached Will Follow

CRO Attached Will Follow

FDA Attached Will Follow

IRB Attached Will Follow

Other Attached Will Follow

**23. INVESTIGATOR ASSURANCE(S)** I have reviewed the contents of this form, with attachments, and I certify that the information provided is complete and accurate to the best of my knowledge.

______________________________________ ______________________________________

Responsible PI Date Investigator Date

______________________________________

Other Date
