# Supplementary material for: Derivation of Xeno-Free and GMP-Grade Human Embryonic Stem Cells – Platforms for Future Clinical Applications
Source: PLoS One. 2012 Jun 20;7(6):e35325. doi: 10.1371/journal.pone.0035325 (PMC3380026; doi:10.1371/journal.pone.0035325)
Supplement: File S16 — Withdrawal from Study Form. (DOC) [file pone.0035325.s030.doc]

# WITHDRAWAL FROM STUDY FORM

# Title of Research Study:

THE DERIVATION OF NEW HUMAN EMBRYONIC STEM CELL LINES FOR CLINICAL USE

I, ________________________________ previously agreed to participate in the research study named above, under the direction of ________________________________ **Principal Investigator.** I understand that my withdrawal will not prejudice my future care.

Although I am no longer actively participating, I am willing not willing to continue to be followed by the study investigator to allow subsequent clinical data to be collected from my medical records to be used for purposes of this project.

Research Subject’s Printed Name: ______________________________________

__________________________________ _____________

Research Subject’s Signature Date

__________________________________ _____________

Signature of Witness Date
