# Supplementary material for: Derivation of Xeno-Free and GMP-Grade Human Embryonic Stem Cells – Platforms for Future Clinical Applications
Source: PLoS One. 2012 Jun 20;7(6):e35325. doi: 10.1371/journal.pone.0035325 (PMC3380026; doi:10.1371/journal.pone.0035325)
Supplement: File S17 — Request for Contact with the Donor Form. (DOC) [file pone.0035325.s031.doc]

# REQUEST FOR CONTACT WITH PATIENT FORM

# Title of Research Study:

THE DERIVATION OF NEW HUMAN EMBRYONIC STEM CELL LINES FOR CLINICAL USE

We, the undersigned, request to contact donor number nhES for the purposes of:

We understand that, according to our IRB application, it is forbidden to make any contact with the donor after he/she has signed the informed consent form and has donated his/her embryo(s), unless specifically permitted by the Director of Hadassah's Obstetrics and Gynecology Department. Contact is permitted without the Director’s approval exclusively for the transmission of laboratory results to the donor. Contact is permitted if the donor contacts the Research Director or associated staff.

Requestor’s name:

Requestor’s Department:

Requestor’s Role in Study:

Date request made:

**I hereby grant my permission for the above-named individual to contact the donor as listed above for the reason detailed above.**

Director of Hadassah's OB-GYN Dept. Name:

Director of Hadassah's OB-GYN Signature and Stamp:

Date approval given:
