# Supplementary material for: Derivation of Xeno-Free and GMP-Grade Human Embryonic Stem Cells – Platforms for Future Clinical Applications
Source: PLoS One. 2012 Jun 20;7(6):e35325. doi: 10.1371/journal.pone.0035325 (PMC3380026; doi:10.1371/journal.pone.0035325)
Supplement: File S18 — Donor Code Log. (DOC) [file pone.0035325.s032.doc]

# DONOR CODE LOG

THE DERIVATION OF NEW HUMAN EMBRYONIC STEM CELL LINES FOR CLINICAL USE

STUDY TITLE:

| **DATE** | **IVF**  **FILE #’S**  **(List all)** | **CHECK SITE IVF PERFORMED (and location of embryos)** | | **FEMALE**  **DONOR** | | **FEMALE**  **DONOR’S 3 INITIALS**  **(Note: If no second name,**  **utilize “X”)** | | | **MALE**  **DONOR** | | **MALE DONOR’S**  **3 INITIALS**  **(Note: If no second name,**  **utilize “X”)** | | | **CODE**  **NUMBER** | **SIGNATURE** |
| --- | --- | --- | --- | --- | --- | --- | --- | --- | --- | --- | --- | --- | --- | --- | --- |
|  |  | **Ein Kerem** | **Mt. Scopus** | **FULL NAME** | **TEUDAT**  **ZEHUT** |  |  |  | **FULL NAME** | **TEUDAT**  **ZEHUT** |  |  |  |  |  |
|  |  |  |  |  |  |  |  |  |  |  |  |  |  | **nhES001** |  |
|  |  |  |  |  |  |  |  |  |  |  |  |  |  | **nhES002** |  |
|  |  |  |  |  |  |  |  |  |  |  |  |  |  | **nhES003** |  |
|  |  |  |  |  |  |  |  |  |  |  |  |  |  | **nhES004** |  |
|  |  |  |  |  |  |  |  |  |  |  |  |  |  | **nhES005** |  |
|  |  |  |  |  |  |  |  |  |  |  |  |  |  | **nhES006** |  |
|  |  |  |  |  |  |  |  |  |  |  |  |  |  | **nhES007** |  |
|  |  |  |  |  |  |  |  |  |  |  |  |  |  | **nhES008** |  |
|  |  |  |  |  |  |  |  |  |  |  |  |  |  | **nhES009** |  |
|  |  |  |  |  |  |  |  |  |  |  |  |  |  | **nhES010** |  |
