# Supplementary material for: Derivation of Xeno-Free and GMP-Grade Human Embryonic Stem Cells – Platforms for Future Clinical Applications
Source: PLoS One. 2012 Jun 20;7(6):e35325. doi: 10.1371/journal.pone.0035325 (PMC3380026; doi:10.1371/journal.pone.0035325)
Supplement: File S19 — Embryo Log. (DOC) [file pone.0035325.s033.doc]

# EMBRYO LOG

THE DERIVATION OF NEW HUMAN EMBRYONIC STEM CELL LINES FOR CLINICAL USE

STUDY TITLE:

| Donor Number | Date Frozen | No. of Embryos to Donate | # Liq. N_2_ Tank | #Holder | #Cannister | Embryo Quality | | | | | | | | |
| --- | --- | --- | --- | --- | --- | --- | --- | --- | --- | --- | --- | --- | --- | --- |
|  |  |  |  |  |  | #A | #B | #AB | #C | #BC | #D | #CD | #1PN | #2PN |
| nhES001 |  |  |  |  |  |  |  |  |  |  |  |  |  |  |
| nhES002 |  |  |  |  |  |  |  |  |  |  |  |  |  |  |
| nhES003 |  |  |  |  |  |  |  |  |  |  |  |  |  |  |
| nhES004 |  |  |  |  |  |  |  |  |  |  |  |  |  |  |
| nhES005 |  |  |  |  |  |  |  |  |  |  |  |  |  |  |
| nhES006 |  |  |  |  |  |  |  |  |  |  |  |  |  |  |
| nhES007 |  |  |  |  |  |  |  |  |  |  |  |  |  |  |
| nhES008 |  |  |  |  |  |  |  |  |  |  |  |  |  |  |
| nhES009 |  |  |  |  |  |  |  |  |  |  |  |  |  |  |
| nhES010 |  |  |  |  |  |  |  |  |  |  |  |  |  |  |
| nhES011 |  |  |  |  |  |  |  |  |  |  |  |  |  |  |
| nhES012 |  |  |  |  |  |  |  |  |  |  |  |  |  |  |
| nhES013 |  |  |  |  |  |  |  |  |  |  |  |  |  |  |
| nhES014 |  |  |  |  |  |  |  |  |  |  |  |  |  |  |
| nhES015 |  |  |  |  |  |  |  |  |  |  |  |  |  |  |
| nhES016 |  |  |  |  |  |  |  |  |  |  |  |  |  |  |
| nhES017 |  |  |  |  |  |  |  |  |  |  |  |  |  |  |
