# Supplementary material for: Derivation of Xeno-Free and GMP-Grade Human Embryonic Stem Cells – Platforms for Future Clinical Applications
Source: PLoS One. 2012 Jun 20;7(6):e35325. doi: 10.1371/journal.pone.0035325 (PMC3380026; doi:10.1371/journal.pone.0035325)
Supplement: File S20 — Health and Acceptance Questionnaire. (DOC) [file pone.0035325.s034.doc]

# Health and Acceptance Questionnaire - CRF

**Questions for both parties**

a. Within 6 months prior to IVF

| **Question** | **N** | **Y** | **Remarks** |
| --- | --- | --- | --- |
| 1. Within the 36 hours prior to IVF has donor taken aspirin or anything that has aspirin in it for *cold/flu symptoms******? | M  F | M  F |  |
| 1. Within the 3 days prior to IVF has donor had extensive dental work? | M  F | M  F |  |
| 1. Within the month prior to IVF has donor taken any medication (including aspirin*****)? If so, specify.   *Nurse should check list of medications that may delay or defer donation (Appendices 1 and 3^+^).* | M  F | M  F |  |
| 1. Within the month prior to IVF, has donor been vaccinated or immunized? If so, specify.   *Nurse should check list of immunizations that may delay donation (Appendix 3+).* | M  F | M  F |  |
| 1. In the 4 weeks prior to IVF, has donor taken Accutane (Isotretinoin), Proscar (Finasteride) or Propecia (Finasteride)? | M  F | M  F |  |
| 1. In the 4 weeks prior to IVF, has donor had any shots (exclusive of IVF medications)? | M  F | M  F |  |
| 1. In the 8 weeks prior to IVF, has donor given blood (including blood tests), plasma, or platelets? Note: Exclusive of IVF-associated blood tests | M  F | M  F |  |
| 8. Within 3 months of IVF, has donor had epilepsy or seizures? | M  F | M  F |  |
| 9. Within 6 months of IVF, did donor have heart  disease, heart murmur, or heart valve disorder? | M  F | M  F |  |
| **b. Within the year prior to IVF** |  |  |  |
| 10. Have donors been in contact with a jaundiced patient or someone who had viral hepatitis within the year prior to IVF? | M  F | M  F |  |
| 11. Has donor had a tattoo/acupuncture/permanent make up within the 12 months before IVF?  *If yes, can donor guarantee that the needles were sterile?*  *(If not, permanently defer).* | M  F  M  F | M  F  M  F |  |
| 1. Has donor suffered from other serious illness- i.e. Heart disease, bleeding tendency, diabetes, epilepsy, surgery, etc. within 1 year of IVF? | M  F | M  F |  |
| 1. Has donor been bitten by an animal or human within a year prior to IVF? | M  F | M  F |  |
| 1. Is donor anticipating surgery now? If so, for what reason? | M  F | M  F |  |
| 1. For 12 months prior to IVF, has donor’s skin been stuck by something that may have been contaminated with blood or body fluids? | M  F | M  F |  |
| 1. In the 12 months prior to IVF, has donor had an ear or skin piercing? | M  F | M  F |  |
| 1. In the 12 months prior to IVF, has someone else's blood been in contact with donor’s open/broken skin or mucous membranes? | M  F | M  F |  |
| 1. In the 12 months prior to IVF, did donor have surgery, or has donor been under a doctor's care for any other reason other than IVF? | M  F | M  F |  |
| 1. In the 12 months prior to IVF, was donor given rabies shots? | M  F | M  F |  |
| 1. In the 12 months prior to IVF, was donor given hepatitis B immune globulin (HBIG)? | M  F | M  F |  |
| 1. Did donor receive blood, an organ, skin graft, or other tissue transplant from a human donor within 12 months of IVF? | M  F | M  F |  |
| **c. Within the 5 years prior to IVF?** |  |  |  |
| 1. Has donor had Brucellosis/Tuberculosis within the last 10 years (i.e. 5 years prior to IVF, and 5 years after IVF)? | M  F | M  F |  |
| 1. Has suffered from any acute or chronic health problem, genetic, or hereditary diseases?   *If yes, provide details.* | M  F | M  F |  |
| 1. Has donor had hepatitis (jaundice) within the 5 years prior to IVF and 5 years after IVF? | M  F | M  F |  |
| 1. Has donor received blood or blood products in the past? *If yes, date and reason for transfusion?* | M  F | M  F |  |
| 1. Has donor had lymphadenopathy, night sweats, weight loss, fever? If so, when? | M  F | M  F |  |
| 1. If donor suffers from diabetes, what type of insulin is donor taking?   *If donor is taking bovine insulin, permanently defer.* | M  F | M  F |  |
| 1. Did donor have Gilbert’s Disease, bile duct obstruction, alcohol use, gallstones, or trauma to the liver within 5 years prior to IVF? | M  F | M  F |  |
| 1. Have any of donor’s blood relatives had Creutzfeldt-Jakob Disease? | M  F | M  F |  |
| 1. Has donor ever received growth hormone made from human pituitary glands?   *NOTE: If the donor is uncertain about his or her treatment, the following question describing human pituitary-derived growth hormone injections may be asked: "Was the hormone treatment given repeatedly by injection?” This question only needs to be asked once, since human pituitary growth hormone is no longer available.*  *-If the donor is uncertain as to whether he/she received human or recombinant growth hormone, permanently defer.* | M  F | M  F |  |
| 1. Has donor received a dura matter (or brain covering) graft?   *Note: This question may be preceded by the more* *general, "Has donor ever had brain surgery?"*  *The specific question should be asked of the donor only if he/she responds "yes," to the general question*. | M  F | M  F |  |
| 1. Has donor ever received a corneal graft? | M  F | M  F |  |
| 1. Has donor ever been given any substance of human pituitary origin (other than human growth hormone)? | M  F | M  F |  |
| 1. Has donor ever been refused as a blood donor, told not to donate, or had problems donating? | M  F | M  F |  |
| 1. Has donor ever had chest pains, heart disease, stroke or T.I.A.? | M  F | M  F |  |
| 1. Has donor ever had cancer, a blood disease, or a bleeding problem? | M  F | M  F |  |
| 1. Did donor ever take Tegison or Soriatane for psoriasis? | M  F | M  F |  |
| 1. Has donor ever had lung disease? | M  F | M  F |  |
| 1. Has donor ever had yellow jaundice or liver disease? | M  F | M  F |  |
| 1. Has donor ever used a needle, even once, to take any drug (including steroids), exclusive of fertility medication?   *If so, list:* | M  F | M  F |  |
| 1. Were there deaths in the donors’ families due to neurological symptoms that may be attributed to vCJD? | M  F | M  F |  |
| 1. Was anyone in donors’ families diagnosed with Gerstmann-Straussler-Scheinlzer disease or Fatal Familial Insomnia? | M  F | M  F |  |
| 1. Were donors ever exposed to West Nile Virus, SARS, or vaccinia (smallpox) virus? | M  F | M  F |  |
| **d. At the time of IVF?** |  |  |  |
| 1. Did the donor feel well at the time of IVF? | M  F | M  F |  |
| 1. Has donor ever had hemophilia? | M  F | M  F |  |
| 1. Is donor a carrier of Hepatitis B or C, ever tested positive for hepatitis, or had sex with someone who either is a carrier or tested positive? | M  F | M  F |  |
| 1. Has donor had a generalized autoimmune disease, including systemic lupus erythematosus, or multiple sclerosis? | M  F | M  F |  |
| 1. Has donor received, since 1980, a transfusion of blood, platelets, plasma, cryoprecipitate, or granulocytes in the UK (England, Northern Ireland, Scotland, Wales, the Isle of Man, the Channel Islands, Gibralter, or the Falkland Islands)? | M  F | M  F |  |
| 1. Had donor received clotting factor concentrates? | M  F | M  F |  |
| 1. Had donor received cancer treatment with chemotherapy, hormone therapy (injectable, human, or animal-derived) or immunotherapy for leukemia, lymphoma, or Hodgkin’s disease? | M  F | M  F |  |
| 1. Did the donor have a clotting disorder, or take coumadin or heparin? | M  F | M  F |  |
| 1. Did the donor have hemochromatosis? | M  F | M  F |  |
| 1. Did donor have an infection, cold, flu, sore throat, or cough at the time of IVF? | M  F | M  F |  |
| 1. Has donor read and understood all information presented to the donor, and have all donor questions been answered? | M  F | M  F |  |
| 1. Were donors in contact with anyone diagnosed with sepsis at the time of IVF? | M  F | M  F |  |
| 1. Did donors smoke cigarettes or pipes at the time of IVF? | M  F | M  F |  |
| **(Separate Questionnaires)** |  |  |  |
|  |  |  |  |
| **Male Donor Only** |  |  |  |
| 1. Has donor received treatment for gonorrhea, syphilis, chlamydia, or other sexually transmitted disease within the year surrounding IVF? |  |  |  |
| 1. In the 12 months prior to IVF, has donor been held in a jail, prison, psychiatric facility, or any correctional facility (for more than 72 hours)? |  |  |  |
| 1. In the 12 months prior to IVF, did donor have sexual contact, even once, with anyone who has ever used a needle to take drugs (exclusive of fertility medications)? |  |  |  |
| 1. In the 12 months prior to IVF, did donor have sexual contact, even once, with anyone who had taken clotting factor concentrates? |  |  |  |
| 1. In the 12 months prior to IVF, did donor have sexual contact, even once, with anyone who has AIDS, had symptoms of HIV, or who has tested positive for the AIDS virus (HIV)? |  |  |  |
| 1. Did donor have any sexual partner, did donor have, or did any member of donor’s household ever have a transplant or other medical procedure that involved being exposed to organs, tissue, or living cells from an animal?   If the answer is yes, then ask the following question:  *Was it donor,*  *a sexual partner,*  *or some other member of donor’s household who had a transplant or otherwise was exposed to organs, tissue, or cells from an animal?”*  *If the answer is “donor”, defer.*  *If the answer is "sexual partner”, defer.*  *If the answer is “other member of donor’s household” , ask the following question:*  *“Was donor repeatedly exposed to blood, saliva, or other body fluids from these individuals through deep kissing, shared toothbrushes, razors, needles, open wounds, or sores?”*  *If the answer is yes, defer.* |  |  |  |
| 1. Has donor or donor’s partner ever tested positive for HIV infection? |  |  |  |
| 1. Did donor ever receive payment for sexual acts? |  |  |  |
| 1. Did male donor ever have sexual contact with another male? |  |  |  |
| 1. Did donor ever use cocaine or other street drugs through the nose? |  |  |  |
| 1. Did donor have symptoms of HIV at the time of IVF?   Initial symptoms may include:   - Fever - Extreme, unexplained fatigue - Swollen lymph nodes in armpits, neck, or groin - Headache - Dry cough - Night sweats - Rash   Once the virus sufficiently weakens the immune system, the following symptoms may occur over the course of 1 to 3 years:   - Swollen lymph glands all over the body - Fatigue - Fungal infections of the mouth, fingernails, toes - Repeated infections (yeast and trichomonas) - Development of lots of warts - Exacerbations of prior conditions, such as eczema, psoriasis, herpes infection - Shingles - Fever - Night sweats - Weight loss - Chronic diarrhea - Memory loss |  |  |  |
| 1. Has donor ever had sex with someone who visited or lived within the 3 years prior to IVF in an area where Malaria/HIV are prevalent?   *See Country List, page 14 and Appendix 4* |  |  |  |
| 1. Has donor used illicit drugs or steroids intravenously or by sniffing, or had sex with someone who has? |  |  |  |
| 1. Has donor ever been treated under a different name? |  |  |  |
| 1. Did donor have sexual contact with anyone who was born in or lived in any country where HIV is prevalent, since 1977?   *See Countries List, page 14.* |  |  |  |
| 1. Had donor had sexual contact with anyone who was born in or lived in any African country for more than 1 year since 1977 (and up to the date of IVF), such as: Africa, except North and South Africa, South East Asia, the Caribbean Islands, Cameroon, Central African Republic, Chad, Congo, Equatorial Guinea, Gabon, Niger, or Nigeria? |  |  |  |
| 1. Does donor understand that if donor has the AIDS virus, donor can transmit it to someone else, even though donor may feel well and has had a negative AIDS test? |  |  |  |
|  |  |  |  |
| Female Donor Only |
| 1. Has donor received treatment for gonorrhea, syphilis, chlamydia, or other sexually transmitted disease within the year surrounding IVF? |  |  |  |
| 1. Within 6 months prior to IVF, has donor been pregnant or given birth? |  |  |  |
| 1. In the 12 months prior to IVF, has donor been held in a jail, prison, psychiatric facility, or any correctional facility (for more than 72 hours)? |  |  |  |
| 1. In the 12 months prior to IVF, did donor have sexual contact, even once, with anyone who has ever used a needle to take drugs (exclusive of fertility medications)? |  |  |  |
| 1. In the 12 months prior to IVF, did donor have sexual contact, even once, with anyone who had taken clotting factor concentrates? |  |  |  |
| 1. In the 12 months prior to IVF, did donor have sexual contact with a male who had sex, even once, since 1977, with another male? |  |  |  |
| 1. In the 12 months prior to IVF, did you have sexual contact, even once, with anyone who had taken clotting factor concentrates? |  |  |  |
| 1. In the 12 months prior to IVF, did donor have sexual contact, even once, with anyone who has AIDS, had symptoms of HIV, or who has tested positive for the AIDS virus (HIV)? |  |  |  |
| 1. Did donor have any sexual partner, did donor have, or did any member of donor’s household ever have a transplant or other medical procedure that involved being exposed to organs, tissue, or living cells from an animal?   *If the answer is yes, then ask the following question:*  *Was it donor,*  *a sexual partner,*  *or some other member of donor’s household who had a transplant or otherwise was exposed to organs, tissue, or cells from an animal?”*  *If the answer is “donor”, defer.*  *If the answer is "sexual partner” , defer.*  *If the answer is “other member of donor’s household” , ask the following question:*  *“Was donor repeatedly exposed to blood, saliva, or other body fluids from these individuals through deep kissing, shared toothbrushes, razors, needles, open wounds, or sores?”*  *If the answer is yes, defer.* |  |  |  |
| 1. Has donor or donor’s partner ever tested positive for HIV infection? |  |  |  |
| 1. Did donor ever receive payment for sexual acts? |  |  |  |
| 1. Did donor ever use cocaine or other street drugs through the nose? |  |  |  |
| 1. Did donor have symptoms of HIV at the time of IVF?   Initial symptoms may include:   - Fever - Extreme, unexplained fatigue - Swollen lymph nodes in armpits, neck, or groin - Headache - Dry cough - Night sweats - Rash   Once the virus sufficiently weakens the immune system, the following symptoms may occur over the course of 1 to 3 years:   - Swollen lymph glands all over the body - Fatigue - Fungal infections of the mouth, fingernails, toes - Repeated vaginal infections (yeast and trichomonas) - Development of lots of warts - Exacerbations of prior conditions, such as eczema, psoriasis, herpes infection - Shingles - Fever - Night sweats - Weight loss - Chronic diarrhea |  |  |  |
| 1. Has donor ever had sex with someone who visited or lived within the 3 years prior to IVF in an area where Malaria/HIV are prevalent?   *See Country List, page 15 and Appendix 4* |  |  |  |
| 1. Has donor used illicit drugs or steroids intravenously or by sniffing, or had sex with someone who has? |  |  |  |
| 1. Has donor ever been treated under a different name? |  |  |  |
| 1. Did donor have sexual contact with anyone who was born in or lived in any country where HIV is prevalent, since 1977?   *See Countries List, page 15.* |  |  |  |
| 1. Had donor had sexual contact with anyone who was born in or lived in any African country for more than 1 year since 1977 (and up to the date of IVF), such as: Africa, except North and South Africa, South East Asia, the Caribbean Islands, Cameroon, Central African Republic, Chad, Congo, Equatorial Guinea, Gabon, Niger, or Nigeria? |  |  |  |
| 1. Does donor understand that if donor has the AIDS virus, donor can transmit it to someone else, even though donor may feel well and has had a negative AIDS test? |  |  |  |
| **d. Countries lived or visited**✠ |  |  |  |
| 1. Have donors visited within one year prior to IVF or lived within the 3 years prior to IVF in an area where Malaria/HIV are prevalent?   *See Country List, pages 14, 15, and Appendix 4*  *Specifically, Africa –except North and South Africa—South East Asia, the Caribbean islands, Cameroon, Central African Republic, Chad, Congo, Equatorial Guinea, Gabon, Niger, or Nigeria.* | M  F | M  F |  |
| 1. Since 1980 and up to date of IVF, have donors ever lived in, or traveled to Europe■ for 5 years or more, cumulative.   *If the answer is no, no further action is needed.*  *(see page 14)*  *If the answer is yes, ask the following questions:*  *Between 1980 and 1996 did donors spend time that adds up to 3 months or more in the U.K. (England, Northern Ireland, Scotland, Wales, the Isle of Man, the Channel Islands, Gibraltar, or the Falkland Islands)?* | M  F  M  F | M  F  M  F |  |
| 1. Were donors born in or lived in any other country other than Israel?   *If yes, list.* | M  F | M  F |  |
| 1. Since 1980 and up to the date of IVF, did donors spend time that adds up to 10 years or more in France, Ireland, or Portugal? ■ | M  F | M  F |  |
| 1. Between 1980 and 1996, were donors members of the military, a civilian military employee, member of a foreign ministry (or equivalent), or a dependent of a member of the military? ►   *If the answer is no, no further action needs to be taken.*  *If the answer is yes, ask the following question:*  *Did donors spend a total time of 6 months or more associated with a military base in any of the following countries in Europe:*   - 1. *From 1980 through 1990 in Northern Europe, such as Belgium, the Netherlands, the UK, or Germany.*   *b. From 1980 through 1996 in Southern Europe, such as Spain, Portugal, Turkey, Italy, or Greece.*  *c. From 1980 to present, donor stationed for 5 years or more (cumulative) in France.*  *See pages 14 and 15.* | M  F  M  F  M  F  M  F | M  F  M  F  M  F  M  F |  |
| 1. Were donors born in, did donors live in, or did donors travel to any of the following countries since 1977 (and up to the date of IVF): Africa (except North & South Africa), South East Asia, the Caribbean Islands, Cameroon, Chad, Equatorial Guinea, Niger, Nigeria, or Gabon? | M  F | M  F |  |
| 1. Did donors live for a cumulative period of 5 years or more in the UK between 1980 – 1996?   *The UK countries specified for exclusion are: England, Wales, Gibralter, Northern Ireland, Isle of Man, Channel Islands, Scotland, and Falkland Islands.* | M  F | M  F |  |

►*If in the military between 1980 – 1996, should not have been stationed in*

*Europe (as described below):*

Albania

Austria

Belgium

Bosnia-Herzegovina

Bulgaria

Croatia

Czech Republic

Denmark

Federal Republic of Yugoslavia

Finland

France

Germany

Greece

Hungary

Italy

Liechtenstein

Luxembourg

Macedonia

Netherlands

Norway

Poland

Portugal

Republic of Ireland

Romania

Slovak Republic

Slovenia

Spain

Sweden

Switzerland

United Kingdom

■Consider donation exclusion if the donor(s) lived:

AREAS WHERE HIV/MALARIA ARE PREVALENT

| **COUNTRY** | **DATES** | **CRITERIA** |
| --- | --- | --- |
| All European Countries | 1980 – date of IVF | 5 years or more cumulative in **any** European country. |
| United Kingdom (U.K.) | 1980 - 1996 | 3 months or more cumulatively (total) |
| France | 1980 – date of IVF | 5 years or more cumulatively (total) |
| Military bases in Germany, United Kingdom, Belgium, Netherlands | 1980 - 1990 | 6 stationed months or more |
| Military bases in Greece, Turkey, Spain, Portugal, and Italy | 1980 - 1996 | 6 stationed months or more |
