# Supplementary material for: Derivation of Xeno-Free and GMP-Grade Human Embryonic Stem Cells – Platforms for Future Clinical Applications
Source: PLoS One. 2012 Jun 20;7(6):e35325. doi: 10.1371/journal.pone.0035325 (PMC3380026; doi:10.1371/journal.pone.0035325)
Supplement: File S21 — Demography. (DOC) [file pone.0035325.s035.doc]

# Demography - CRF

Visit Date:

dd mm yy

Female: Male:

Date of Birth: **F**: **M**:

dd mm yy dd mm yy

Country of Birth: **F**:_______________________ **M**:_______________________

Did you live outside Israel Between 1980-1996? **F**: Yes No

**M**: Yes No

If yes, where (**F**)___________________ (see attached list)

(**M**)___________________

Lived outside Israel for > 5 years (Cumulative) **F**: Y 🞏 N🞏 If Yes specify:___________

**M**: Y 🞏 N🞏 If Yes specify:___________

Military Service: (**F**)Y 🞏 N🞏 If Yes, specify: Europe 🞏 USA🞏 Israel 🞏 Other _______

(**M**)Y 🞏 N🞏 If Yes, specify: Europe 🞏 USA🞏 Israel 🞏 Other _______

Date of Immigration to Israel: **F** From Where? __________

dd mm yy

**M** From Where? __________

dd mm yy

Race: **F:** Caucasian 🞏 **M:** Caucasian 🞏

Black 🞏 Black 🞏

Oriental 🞏 Oriental 🞏

Other____________ Other_____________

Please specify Please specify

Origin: **F:** Ashkenazi 🞏 **M:** Ashkenazi 🞏

Sephardi 🞏 Sephardi 🞏

Aravi 🞏 Aravi 🞏

Other____________ Other_____________

Please specify Please specify
