# Supplementary material for: Derivation of Xeno-Free and GMP-Grade Human Embryonic Stem Cells – Platforms for Future Clinical Applications
Source: PLoS One. 2012 Jun 20;7(6):e35325. doi: 10.1371/journal.pone.0035325 (PMC3380026; doi:10.1371/journal.pone.0035325)
Supplement: File S22 — Informed Consent. (DOC) [file pone.0035325.s036.doc]

### INFORMED CONSENT - CRF

The Informed Consent must be signed by each one of the donors (female and male) **before** embryos are utilized in the research.

| Informed Consent Was written informed consent obtained from both donors prior to utilizing the embryos in research?  **F: Yes** **No**  **M: Yes** **No** Date consent(s) obtained: **F** Day Month YearM   Day Month Year  (Place the original signed informed consent in the donors’ file. A copy should be given to the donors.)  **IVF Nurse: _____________________________(signature) Date: ____________** |
| --- |
