# Supplementary material for: Derivation of Xeno-Free and GMP-Grade Human Embryonic Stem Cells – Platforms for Future Clinical Applications
Source: PLoS One. 2012 Jun 20;7(6):e35325. doi: 10.1371/journal.pone.0035325 (PMC3380026; doi:10.1371/journal.pone.0035325)
Supplement: File S23 — IVF Treatment Cycles. (DOC) [file pone.0035325.s037.doc]

**IVF Treatment CRF**

1. **INDICATIONS FOR IVF TREATMENT**

|  | INDICATIONS | | CAUSE  (Check all that apply) |
| --- | --- | --- | --- |
| F E  M  A  L  E | 1 | Mechanical |  |
|  | 2 | Endometriosis |  |
|  | 3 | Anovulation |  |
|  | 4 | Age >40 |  |
| M  A  L  E | 1 | OTA Syndrome |  |
|  | 2  3 | Azospermia  A. Non Obstructive  i. Idiopathic  ii. Acquired (Radiation/Chemotherapy)  B. Obstructive  Erectile/Ejaculate Dysfunction |  |
| O  T  H  E  R | 1 | Unexplained |  |
|  | 2 | Habitual Abortions |  |

1. **MEDICATIONS**

(For female)

| MEDICATIONS USED  DURING INFERTILITY TREATMENT FOR CYCLES **BEFORE CYCLE OF EMBRYO DONATION**  (CHECK ALL THAT APPLY) | | | | | | | | | | | | | | |
| --- | --- | --- | --- | --- | --- | --- | --- | --- | --- | --- | --- | --- | --- | --- |
| **ALL INJECTED MEDICATIONS:**  Y N N/A With new sterile syringes and needles  Y N N/A Were self injected, and/or  Y N N/A Were injected by an authorized nurse | | | | | | | | | | | | | | |
| **FSH PREPARATIONS** | | | | | **HCG PREPARATIONS** | | | | | **GnRH**  **AGONISTS/ANTAGONISTS** | | **PROGESTERONE** | | |
| *URINARY* | | *RECOMBINANT* | | | *URINARY* | | *RECOMBINANT* | | | DECAPEPTYL | | GESTONE |  | |
| PERGONAL |  | GONAL-F | |  | CHORIGON |  | OVITRELLE |  | | DEPO 3.75  SHORT- CIRCLE ONE  ACTING 0.05, 0.1, 0.2 | |  |  |  |
| MENOGON |  | PUREGON | |  | PREGNYL |  |  | | | SYNAREL |  | UTROGESTAN | | |
| METRODIN |  |  | | |  | | | | | SUPREFACT |  | CRINONE 8% | | |
| FOSTIMON |  |  |  |  |  |  |  |  |  | CETROTIDE |  | ENDOMETRIN | | |
| MENOPURE |  |  |  |  |  |  |  |  |  | ORGALUTRAN |  |  | | |
| METRODIN HP |  |  |  |  |  |  |  |  |  | BUSERELIN |  |  | | |
| HUMAGON |  |  |  |  |  |  |  |  |  |  | |  | | |
| **ESTROGEN PREPARATIONS** | | | | | **GENERAL/OTHER** | | | | | | | | | |
|  | BEFORE ET | AFTER ET |  | | DEXAMETH/STEROIDS | |  | | DOXYLIN | | | DOPERGIN | |  |
| ESTROFEM |  |  |  | | ASPIRIN - CARTIA | |  | | CLOMIPHENE  CITRATE (IKACLOMIN) | | | PARILAC | |  |
| PROGYNOVA |  |  |  | | FOLIC ACID | |  | | GROWTH HORMONE | | | DOSTINEX | |  |
| VAG. TAB ESTRADIOL |  |  |  | | PARTNER’S LYMPHOCYTES | |  | | GLUCOPHAGE (METFORMIN) | | | ORAL CONTRACEP. | |  |
| OTHER |  |  |  | | I.V. Ig | |  | | CLEXAN | | |  | | |

(For Female)

| MEDICATIONS USED  DURING INFERTILITY TREATMENT FOR CYCLES **OF EMBRYO DONATION**  (CHECK ALL THAT APPLY) | | | | | | | | | | | | | | |
| --- | --- | --- | --- | --- | --- | --- | --- | --- | --- | --- | --- | --- | --- | --- |
| **ALL INJECTED MEDICATIONS:**  Y N N/A With new sterile syringes and needles  Y N N/A Were self injected, and/or  Y N N/A Were injected by an authorized nurse | | | | | | | | | | | | | | |
| **FSH PREPARATIONS** | | | | | **HCG PREPARATIONS** | | | | | **GnRH**  **AGONISTS/ANTAGONISTS** | | **PROGESTERONE** | | |
| *URINARY* | | *RECOMBINANT* | | | *URINARY* | | *RECOMBINANT* | | | DECAPEPTYL | | GESTONE |  | |
| PERGONAL |  | GONAL-F | |  | CHORIGON |  | OVITRELLE |  | | DEPO 3.75  SHORT- CIRCLE ONE  ACTING 0.05, 0.1, 0.2 | |  |  |  |
| MENOGON |  | PUREGON | |  | PREGNYL |  |  | | | SYNAREL |  | UTROGESTAN | | |
| METRODIN |  |  | | |  | | | | | SUPREFACT |  | CRINONE 8% | | |
| FOSTIMON |  |  |  |  |  |  |  |  |  | CETROTIDE |  | ENDOMETRIN | | |
| MENOPURE |  |  |  |  |  |  |  |  |  | ORGALUTRAN |  |  | | |
| METRODIN HP |  |  |  |  |  |  |  |  |  | BUSERELIN |  |  | | |
| HUMAGON |  |  |  |  |  |  |  |  |  |  | |  | | |
| **ESTROGEN PREPARATIONS** | | | | | **GENERAL/OTHER** | | | | | | | | | |
|  | BEFORE ET | AFTER ET |  | | DEXAMETH/STEROIDS | |  | | DOXYLIN | | | DOPERGIN | |  |
| ESTROFEM |  |  |  | | ASPIRIN - CARTIA | |  | | CLOMIPHENE  CITRATE (IKACLOMIN) | | | PARILAC | |  |
| PROGYNOVA |  |  |  | | FOLIC ACID | |  | | GROWTH HORMONE | | | DOSTINEX | |  |
| VAG. TAB ESTRADIOL |  |  |  | | PARTNER’S LYMPHOCYTES | |  | | GLUCOPHAGE (METFORMIN) | | | ORAL CONTRACEP. | |  |
| OTHER |  |  |  | | I.V. Ig | |  | | CLEXAN | | |  | | |

(For male)

| MEDICATIONS USED TO TREAT MALE INFERTILITY  DURING INFERTILITY TREATMENT FOR CYCLES **BEFORE CYCLE OF EMBRYO DONATION**  (CHECK ALL THAT APPLY) | | |
| --- | --- | --- |
| **ALL INJECTED MEDICATIONS:**  Y N N/A With new sterile syringes and needles  Y N N/A Were self injected, and/or  Y N N/A Were injected by an authorized nurse | | |
| **CHECK ALL THE MEDICATIONS THAT APPLY** | | |
|  | | **LIST MEDICATION NAME** |
| FSH PREPARATION |  |  |
| HCG PREPARATION |  |  |
| CLOMIPHENE CITRATE (IKACLOMIN) |  |  |
| TESTOSTERONE PREPARATION |  |  |
| VIAGRA |  |  |
| PHENTOLAMINE (REGETINE) |  |  |
| IMIPRAMINE (TOFRANIL) |  |  |
| YOHEMBIN |  |  |
| DEXAMETHASONE |  |  |
| OTHER |  |  |

| INJECTED MEDICATIONS USED TO TREAT MALE INFERTILITY  DURING INFERTILITY TREATMENT FOR CYCLES **OF EMBRYO DONATION**  (CHECK ALL THAT APPLY) | | |
| --- | --- | --- |
| **ALL INJECTED MEDICATIONS:**  Y N N/A With new sterile syringes and needles  Y N N/A Were self injected, and/or  Y N N/A Were injected by an authorized nurse | | |
| **CHECK ALL THE MEDICATIONS THAT APPLY** | | |
|  | | **LIST MEDICATION NAME** |
| FSH PREPARATION |  |  |
| HCG PREPARATION |  |  |
| CLOMIPHENE CITRATE (IKACLOMIN) |  |  |
| TESTOSTERONE PREPARATION |  |  |
| VIAGRA |  |  |
| PHENTOLAMINE (REGETINE) |  |  |
| IMIPRAMINE (TOFRANIL) |  |  |
| YOHEMBIN |  |  |
| DEXAMETHASONE |  |  |
| OTHER |  |  |

**3. IVF TREATMENTS PRIOR TO AND INCLUDING CYCLES OF EMBRYO DONATION**

**i. IVF CYCLE IN OTHER HOSPITALS**:

- 1. NAME OF UNIT: _________________________________
  2. NUMBER OF CYCLES OF OOCYTE PICKUP: ____________________________
  3. FROM YEAR TO
  4. MEDICATIONS: (Check one)
     1. All injected medications are detailed above
     2. Treatment included injected medications not listed above, as follows: ________________________________________________________________________________________

**ii. IVF CYCLES IN HADASSAH PRIOR TO AND INCLUDING CYCLE/S OF EMBRYO DONATION**

1. NUMBER OF ATTEMPTS (OOCYTE ASPIRATION AND IVF):
2. MEDICATIONS: (Check one)
   - 1. All injected medications are detailed above
     2. Treatment included injected medications not listed above, as follows: _______________________________________________________________________________
3. SUMMARY OF IVF CYCLES IN HADASSAH (CHECK ALL THAT APPLY):

| **CYCLE**  **#** | **DATE OF OOCYTE PICKUP** | | | | **# OF**  **OOCYTES** | **ART** | | | | | **# OF 2PN**. | | **# OF EMBRYOS** | | **RESULTANT**  **PREGNANCY** | | | | |
| --- | --- | --- | --- | --- | --- | --- | --- | --- | --- | --- | --- | --- | --- | --- | --- | --- | --- | --- | --- |
|  | ***dd*** | ***mm*** | | ***yy*** |  | ***IVF*** | ***SUZI*** | ***ICSI*** | ***AH*** | ***HA*** |  | | **# OF EMBRYOS**  **TRANSF.** | **# OF EMBRYOS FROZEN** | ***YES*** | | | | ***NO*** |
|  |  |  |  |  |  |  |  |  |  |  |  |  |  |  | **FRESH** | | **FROZEN** | |  |
|  | | | | | | | | | | | | | | | **# SACS** | **NO**  **SACS** | **# SACS** | **NO**  **SACS** |  |
|  |  | |  |  |  |  |  |  |  |  |  |  |  |  |  |  |  |  |  |
|  |  | |  |  |  |  |  |  |  |  |  |  |  |  |  |  |  |  |  |
|  |  | |  |  |  |  |  |  |  |  |  |  |  |  |  |  |  |  |  |
|  |  | |  |  |  |  |  |  |  |  |  |  |  |  |  |  |  |  |  |
|  |  | |  |  |  |  |  |  |  |  |  |  |  |  |  |  |  |  |  |
|  |  | |  |  |  |  |  |  |  |  |  |  |  |  |  |  |  |  |  |
|  |  | |  |  |  |  |  |  |  |  |  |  |  |  |  |  |  |  |  |
|  |  | |  |  |  |  |  |  |  |  |  |  |  |  |  |  |  |  |  |
|  |  | |  |  |  |  |  |  |  |  |  |  |  |  |  |  |  |  |  |
|  |  | |  |  |  |  |  |  |  |  |  |  |  |  |  |  |  |  |  |
|  |  | |  |  |  |  |  |  |  |  |  |  |  |  |  |  |  |  |  |
|  |  | |  |  |  |  |  |  |  |  |  |  |  |  |  |  |  |  |  |
|  |  | |  |  |  |  |  |  |  |  |  |  |  |  |  |  |  |  |  |
|  |  | |  |  |  |  |  |  |  |  |  |  |  |  |  |  |  |  |  |
|  |  | |  |  |  |  |  |  |  |  |  |  |  |  |  |  |  |  |  |

**4. OBSTETRIC HISTORY (ALL PREGNANCIES):**

| **OBSTETRIC HISTORY - ALL** | | | | | | | | | | | | | | | |
| --- | --- | --- | --- | --- | --- | --- | --- | --- | --- | --- | --- | --- | --- | --- | --- |
|  | PREVIOUS HUSBAND/PARTNER | | | | | | | CURRENT HUSBAND/PARTNER | | | | | | | |
|  | **SPONTANEOUS PREGNANCY** | | | **INFERTILITY TREATMENT** | | | | **SPONTANEOUS PREGNANCY** | | | | | **INFERTILITY TREATMENT** | | |
| Number of Pregnancies |  | | |  | | | |  | | | | |  | | |
| Number of deliveries (>25 weeks) |  | | |  | | | |  | | | | |  | | |
| Number of Abortions | SPONTANEOUS | INDUCED | | SPONTANEOUS | | | INDUCED | SPONTANEOUS | | INDUCED | | | SPONTANEOUS | | INDUCED |
|  |  |  |  |  |  |  |  |  |  |  |  |  |  |  |  |
| Number of Children |  | | |  | | | |  | | | | |  | | |
| Number of Stillbirths |  | | |  | | | |  | | | | |  | | |
| Stillbirth Details | PATHOLOGY RESULTS, IF AVAILABLE | | KARYOTYPE | PATHOLOGY RESULTS, IF AVAILABLE | | KARYOTYPE | | PATHOLOGY RESULTS, IF AVAILABLE | KARYOTYPE | | PATHOLOGY RESULTS, IF AVAILABLE | | | KARYOTYPE | |
| 1 |  | |  |  | |  | |  |  | |  | | |  | |
| 2 |  | |  |  | |  | |  |  | |  | | |  | |
| 3 |  | |  |  | |  | |  |  | |  | | |  | |
| Newborn/Fetal Anomalies Yes No 1      2 | DESCRIBE: | | | | DESCRIBE: | | | DESCRIBE: | | | | DESCRIBE: | | | |
|  | DESCRIBE: | | | | DESCRIBE: | | | DESCRIBE: | | | | DESCRIBE: | | | |
| Genetic Abnormalities Diagnosed in those that were (Pregnancies) Terminated/Aborted 1  Yes No  2 | DESCRIBE: | | | | DESCRIBE: | | | DESCRIBE: | | | | DESCRIBE: | | | |
|  | DESCRIBE: | | | | DESCRIBE: | | | DESCRIBE: | | | | DESCRIBE: | | | |
| Genetic or Hereditary Syndromes/Diseases 1  in Newborns/Children  Yes No  2 | DESCRIBE: | | | | DESCRIBE: | | | DESCRIBE: | | | | DESCRIBE: | | | |
|  | DESCRIBE: | | | | DESCRIBE: | | | DESCRIBE: | | | | DESCRIBE: | | | |
| Health Problems of Children 1  Yes No  2 | DESCRIBE: | | | | DESCRIBE: | | | DESCRIBE: | | | | DESCRIBE: | | | |
|  | DESCRIBE: | | | | DESCRIBE: | | | DESCRIBE: | | | | DESCRIBE: | | | |

| **OBSTETRIC HISTORY OF IVF CYCLES** | | | | | | | | | | | | | | | | | | | | | |
| --- | --- | --- | --- | --- | --- | --- | --- | --- | --- | --- | --- | --- | --- | --- | --- | --- | --- | --- | --- | --- | --- |
|  | | **PREGNANCY** | | | | | | | | | | | **DELIVERY** | | | | | | **DIAGNOSIS/PATHOLOGY**  **/KARYOTYPE**  **DETAILS** | | |
|  |  | **ABNORMAL**  **DEVELOPMENT** | | | | | | | | | | **NORMAL**  **DEVEL.** | **# HEALTHY** | | **# NON-**  **HEALTHY/DIED** | | **#**  **STILLBORN** | |  | | |
| **CYC.**  **#** | **PREG.**  **#** | **# OF SACS** | **# WITH PULSES** | **ABORTION** | | **FETAL AB.** | | | **KARYOTYPE** | | |  | **Male** | **Fem.** | **Male** | **Fem.** | **Male** | **Fem.** | **DETAILS ABOVE?**  **(page 8)** | | **NOT**  **DETERM.** |
|  | | | | **<12W** | **>12W** | **Y** | **N** | **UN*** | **Nor.** | **Ab.** | **UN*** |  |  |  |  |  |  |  | **YES** | **NO** |  |
|  |  |  |  |  |  |  |  |  |  |  |  |  |  |  |  |  |  |  |  |  |  |
|  |  |  |  |  |  |  |  |  |  |  |  |  |  |  |  |  |  |  |  |  |  |
|  |  |  |  |  |  |  |  |  |  |  |  |  |  |  |  |  |  |  |  |  |  |
|  |  |  |  |  |  |  |  |  |  |  |  |  |  |  |  |  |  |  |  |  |  |
|  |  |  |  |  |  |  |  |  |  |  |  |  |  |  |  |  |  |  |  |  |  |
|  |  |  |  |  |  |  |  |  |  |  |  |  |  |  |  |  |  |  |  |  |  |
|  |  |  |  |  |  |  |  |  |  |  |  |  |  |  |  |  |  |  |  |  |  |
|  |  |  |  |  |  |  |  |  |  |  |  |  |  |  |  |  |  |  |  |  |  |
|  |  |  |  |  |  |  |  |  |  |  |  |  |  |  |  |  |  |  |  |  |  |
|  |  |  |  |  |  |  |  |  |  |  |  |  |  |  |  |  |  |  |  |  |  |

***UN=Undetermined**

**5. DETAILS OF FROZEN DONATED EMBRYOS**

**IVF Treatment Cycles 1-2**

|  | **DATE OF OOCYTE PICKUP** | | | | | | | | | | | | | | | | | | | | | | | | | | | | | | | | | | | | | |
| --- | --- | --- | --- | --- | --- | --- | --- | --- | --- | --- | --- | --- | --- | --- | --- | --- | --- | --- | --- | --- | --- | --- | --- | --- | --- | --- | --- | --- | --- | --- | --- | --- | --- | --- | --- | --- | --- | --- |
|  | ATTEMPT # | | | | | | | | | | | | | | | | | | | | ATTEMPT # | | | | | | | | | | | | | | | | | |
|  | **F** Age | | | | Day (of attempt) | | | | | | Month (of attempt) | | | | | | Year (of attempt) | | | | **F** Age | | | | Day (of attempt) | | | | | Month (of attempt) | | | | | Year (of attempt) | | | |
|  |  |  |  |  |  |  |  |  |  |  |  |  |  |  |  |  |  |  |  |  |  |  |  |  |  |  |  |  |  |  |  |  |  |  |  |  |  |  |
| **ART Used** | IVF | | | | | | | SUZI | | | | | | | ICSI | | | | | | IVF | | | | | | SUZI | | | | | | ICSI | | | | | |
|  | AH | | | | | | | TEST. SPERM  EPIDID. SPERM | | | | | | | EJAC.  SPERM | | | | | | AH | | | | | | TEST. SPERM  EPIDID. SPERM | | | | | | EJAC.  SPERM | | | | | |
| **Date of Freezing** |  | | | | | | | | | | | | | | | | | | | |  | | | | | | | | | | | | | | | | | |
| **# Liq. N_2_ tank** |  | | | | | | | | | | | | | | | | | | | |  | | | | | | | | | | | | | | | | | |
| **# Holder**  **(in letters, א'-יג')** |  | | | | | | | | | | | | | | | | | | | |  | | | | | | | | | | | | | | | | | |
| **Cannister # (1-10)** |  | | | | | | | | | | | | | | | | | | | |  | | | | | | | | | | | | | | | | | |
| **Total # Embryos** | Total # Embryos  In Attempt | | | | | | Total # Embryos  Used by Donor | | | | | | Total # Embryos  Available to Donate | | | | | | | | Total # Embryos  In Attempt | | | | | | Total # Embryos  Used by Donor | | | | | | Total # Embryos  Available to Donate | | | | | |
| **# Ampule**  **From Date** | # Ampule  From Date | | | | | | | # Ampule  From Date | | | | | | | # Ampule  From Date | | | | | | # Ampule  From Date | | | | | | # Ampule  From Date | | | | | | # Ampule  From Date | | | | | |
| **Embryo # (i.e. I, II, III)** |  |  |  |  | |  | |  |  |  | |  | |  |  |  | |  |  |  |  |  |  |  | |  |  |  |  | |  |  |  |  | |  |  |  |
| **Division Stage Cell #** |  |  |  |  | |  | |  |  |  | |  | |  |  |  | |  |  |  |  |  |  |  | |  |  |  |  | |  |  |  |  | |  |  |  |
| **Embryo Quality** A |  |  |  |  | |  | |  |  |  | |  | |  |  |  | |  |  |  |  |  |  |  | |  |  |  |  | |  |  |  |  | |  |  |  |
| B |  |  |  |  | |  | |  |  |  | |  | |  |  |  | |  |  |  |  |  |  |  | |  |  |  |  | |  |  |  |  | |  |  |  |
| AB |  |  |  |  | |  | |  |  |  | |  | |  |  |  | |  |  |  |  |  |  |  | |  |  |  |  | |  |  |  |  | |  |  |  |
| C |  |  |  |  | |  | |  |  |  | |  | |  |  |  | |  |  |  |  |  |  |  | |  |  |  |  | |  |  |  |  | |  |  |  |
| BC |  |  |  |  | |  | |  |  |  | |  | |  |  |  | |  |  |  |  |  |  |  | |  |  |  |  | |  |  |  |  | |  |  |  |
| D |  |  |  |  | |  | |  |  |  | |  | |  |  |  | |  |  |  |  |  |  |  | |  |  |  |  | |  |  |  |  | |  |  |  |
| CD |  |  |  |  | |  | |  |  |  | |  | |  |  |  | |  |  |  |  |  |  |  | |  |  |  |  | |  |  |  |  | |  |  |  |
| 1PN |  |  |  |  | |  | |  |  |  | |  | |  |  |  | |  |  |  |  |  |  |  | |  |  |  |  | |  |  |  |  | |  |  |  |
| 2PN |  |  |  |  | |  | |  |  |  | |  | |  |  |  | |  |  |  |  |  |  |  | |  |  |  |  | |  |  |  |  | |  |  |  |
| Ø |  |  |  |  | |  | |  |  |  | |  | |  |  |  | |  |  |  |  |  |  |  | |  |  |  |  | |  |  |  |  | |  |  |  |
| **Stage Frozen** 2PN |  |  |  |  | |  | |  |  |  | |  | |  |  |  | |  |  |  |  |  |  |  | |  |  |  |  | |  |  |  |  | |  |  |  |
| 48Hr |  |  |  |  | |  | |  |  |  | |  | |  |  |  | |  |  |  |  |  |  |  | |  |  |  |  | |  |  |  |  | |  |  |  |
| 72Hr |  |  |  |  | |  | |  |  |  | |  | |  |  |  | |  |  |  |  |  |  |  | |  |  |  |  | |  |  |  |  | |  |  |  |
| Other |  |  |  |  | |  | |  |  |  | |  | |  |  |  | |  |  |  |  |  |  |  | |  |  |  |  | |  |  |  |  | |  |  |  |

**6. GENETIC DISEASES**

i. IS THERE ANY FAMILIAL HISTORY OF KNOWN GENETIC DISEASES IN EITHER THE MALE OR FEMALE DONOR’S HISTORY? YES NO Check One: Male’s Family Female’s Family

IF YES, WHICH DISEASE(S)? **F**____________________________________________

**M**____________________________________________

ii. WAS ANY GENETIC TESTING PERFORMED ON DONORS? YES NO

IF YES, OR IF THE DONORS ARE AWARE THAT THEY ARE CARRIERS OR HAVE GENETIC DISEASES, COMPLETE THE CHART BELOW:

|  | | Canovan’s Disease | Familial Dysautono-mia | ML4 | Y-Deletion | Fanconi’s Anemia | Glycogen Storage Disease 1a | Bloom Syndrome | Von Wilebrand’s | Ataxia-telangiectasia | Fragile-X | Gaucher’s | Tay-Sach’s | SC Anemia | Thalassemia. | Hemophilia. A or B | Usher Syndrome Type 1 | Duchenne’s | Niemann Pick | Cystic Fibrosis | Polycys Kidney disease |
| --- | --- | --- | --- | --- | --- | --- | --- | --- | --- | --- | --- | --- | --- | --- | --- | --- | --- | --- | --- | --- | --- |
| **FEMALE** | **NOT CARRIER** |  |  |  |  |  |  |  |  |  |  |  |  |  |  |  |  |  |  |  |  |
|  | **CARRIER** |  |  |  |  |  |  |  |  |  |  |  |  |  |  |  |  |  |  |  |  |
|  | **HAS DISEASE** |  |  |  |  |  |  |  |  |  |  |  |  |  |  |  |  |  |  |  |  |
| **MALE** | **NOT CARRIER** |  |  |  |  |  |  |  |  |  |  |  |  |  |  |  |  |  |  |  |  |
|  | **CARRIER** |  |  |  |  |  |  |  |  |  |  |  |  |  |  |  |  |  |  |  |  |
|  | **HAS DISEASE** |  |  |  |  |  |  |  |  |  |  |  |  |  |  |  |  |  |  |  |  |

- - 1. WERE THE KARYOTYPES OF THE MALE AND FEMALE TESTED? YES NO
    2. WHAT WERE THE RESULTS OF THE KARYOTYPE TESTING?

| KARYOTYPE | FEMALE | MALE |
| --- | --- | --- |
| NORMAL |  |  |
| ABNORMAL |  |  |

**MEDICAL DIRECTOR’S DISPENSATION:**

1. **DONOR COUPLE HAS A GENETIC DISEASE THAT MAY DISQUALIFY THEM FROM DONATING EMBRYOS TO THE STUDY. DESCRIBE**
2. **EMBRYOS HAVE A PROBLEM THAT MAY DISQUALIFY THEM FROM BEING INCLUDED IN THE STUDY.**

**DESCRIBE**

**ACCEPT REJECT**

**MEDICAL DIRECTOR’S SIGNATURE __________________________________________**

**DATE ________________________**
