# Supplementary material for: Derivation of Xeno-Free and GMP-Grade Human Embryonic Stem Cells – Platforms for Future Clinical Applications
Source: PLoS One. 2012 Jun 20;7(6):e35325. doi: 10.1371/journal.pone.0035325 (PMC3380026; doi:10.1371/journal.pone.0035325)
Supplement: File S24 — Laboratory Tests Prior to IVF. (DOC) [file pone.0035325.s038.doc]

# LABORATORY TESTS PERFORMED PRIOR TO IVF - CRF

NOTE: COMPLETE ONE LABORATORY TESTS FORM FOR EACH MALE AND FEMALE DONOR

Were the following laboratory analyses performed? If so, where, and which health fund?

CHECK ONE: M F

Chlamydia Ab: IgM Yes No Date performed: ----/----/----- Location: ____________

dd mm yy

IgG Yes No Date performed: ----/----/----- Location: ____________

dd mm yy

Hbs Ag Yes No Date performed: ----/----/----- Location: ____________

dd mm yy

HBab (anti HB_core_) Yes No Date performed: ----/----/----- Location: ____________

dd mm yy

HCab Yes No Date performed: ----/----/----- Location: ___________

dd mm yy

HIV 1 + 2 Ab Yes No Date performed: ----/----/----- Location: ___________

dd mm yy

HTLV 1 + 2 Ab Yes No Date performed: ----/----/----- Location: ___________

dd mm yy

Syphilis-G Yes No Date performed: ----/----/----- Location: ___________

dd mm yy

Rubella IgM Yes No Date performed: ----/----/----- Location: ___________

dd mm yy

IgG Yes No Date performed: ----/----/----- Location: ___________

dd mm yy

Gonorrhea Yes No Date performed: ----/----/----- Location: ____________

(Swab) dd mm yy

CMV IgM Yes No Date performed: ----/----/----- Location ____________

dd mm yy

IgG Yes No Date performed: ----/----/----- Location ____________

dd mm yy

EBV IgM Yes No Date performed: ----/----/----- Location ____________

dd mm yy

IgG Yes No Date performed: ----/----/----- Location ____________

dd mm yy

EBNA Yes No Date performed: ----/----/----- Location ____________

dd mm yy

CBC Yes No Date performed: ----/----/----- Location: ___________

dd mm yy

Blood Type Yes No Date performed: ----/----/----- Location: ___________

dd mm yy

2. Are there ANY clinical laboratory abnormalities that would EXCLUDE the

donor from participating in the study? Yes No

If yes, please comment ________________________________________________________

IVF Nurse’s Signature and Date: ______________________________________________________
