# Supplementary material for: Derivation of Xeno-Free and GMP-Grade Human Embryonic Stem Cells – Platforms for Future Clinical Applications
Source: PLoS One. 2012 Jun 20;7(6):e35325. doi: 10.1371/journal.pone.0035325 (PMC3380026; doi:10.1371/journal.pone.0035325)
Supplement: File S25 — Medical History Authorization. (DOC) [file pone.0035325.s039.doc]

THE DERIVATION OF NEW HUMAN EMBRYONIC STEM CELL LINES FOR CLINICAL USE

STUDY TITLE

## MEDICAL HISTORY AUTHORIZATION

We, the donor couple, understand that the necessity for the full disclosure of our medical history is critical to the success of the project and for the health and safety of potential patients who will be treated with our cells in the future.

We therefore grant full permission to the research staff to contact our private physician regarding our medical histories and allow full access to any and all laboratory tests, medical records, and any other medical information that may be necessary to assess our eligibility to the research program.

**Donor Female:**

Private Physician’s Name _____________________________

Private Physician’s Phone Number ______________________

Health Fund ________________________________________

Printed Name _______________________________________

Signature __________________________________________

Date ______________________________________________

**Donor Male:**

Private Physician’s Name _____________________________

Private Physician’s Phone Number ______________________

Health Fund ________________________________________

Printed Name _______________________________________

Signature __________________________________________

Date ______________________________________________

Such access to medical records may include (but is not limited to): direct primary-physician contact, hospital/medical records (Hadassah or other), or donor’s OB/GYN.

Donor, therefore, gives permission for such access by signing above and completing the following information:

Donor Female:

| **Possible Contact** | **Name/Contact Details** | **Available** | **Not Available** |
| --- | --- | --- | --- |
| Primary (Family) Physician | (Given on prior page) |  |  |
| Hadassah Hospital Medical Records |  |  |  |
| OB/GYN |  |  |  |
| Other Hospital  Medical Records |  |  |  |

Donor Male:

| **Possible Contact** | **Name/Contact Details** | **Available** | **Not Available** |
| --- | --- | --- | --- |
| Primary (Family) Physician | (Given on prior page) |  |  |
| Hadassah Hospital Medical Records |  |  |  |
| Other Hospital  Medical Records |  |  |  |

WITNESS: (Signature) ________________________ Date ______________
