# Supplementary material for: Derivation of Xeno-Free and GMP-Grade Human Embryonic Stem Cells – Platforms for Future Clinical Applications
Source: PLoS One. 2012 Jun 20;7(6):e35325. doi: 10.1371/journal.pone.0035325 (PMC3380026; doi:10.1371/journal.pone.0035325)
Supplement: File S26 — Physical Examination. (DOC) [file pone.0035325.s040.doc]

# PHYSICAL EXAMINATION - CRF

**Gender: M/F (Circle One)**

STEP 1: Perform Physical Examination

|  |
| --- |

**VITAL SIGNS**

| ***Height*** | (m) | BP | mmHg |
| --- | --- | --- | --- |
| ***Weight*** | (kg) | ***Pulse*** | bpm |
| ***Frame size*** | Small Medium Large  (Circle) | ***Resps*** | (per min) |

## PHYSICAL EXAMINATION

|  | CHECK AS APPROPRIATE | | |  |
| --- | --- | --- | --- | --- |
|  | NORMAL | ABNORMAL | NOT  DONE | ADDITIONAL DETAILS |
| EAR NOSE AND THROAT |  |  |  |  |
| OPHTHALMOLOGICAL  (FUNDAL EXAM NOT INCLUDED) |  |  |  |  |
| DERMATOLOGICAL |  |  |  |  |
| CARDIOVASCULAR SYSTEM |  |  |  |  |
| RESPIRATORY |  |  |  |  |
| GASTRO-INTESTINAL/  ABDOMINAL |  |  |  |  |
| NEUROLOGICAL |  |  |  |  |
| LYMPH NODES |  |  |  |  |
| BREASTS |  |  |  |  |
| REPRODUCTIVE |  |  |  |  |
| MUSCULO-SKELETAL |  |  |  |  |
| OTHER |  |  |  |  |

If any of the above systems were not examined, please explain why: ______________________________________________________________________________________________________________________________________

**NOTE: If donor refuses a genital examination, list the results of the examination performed during IVF treatment from his/her medical record above and check here:**

**Refused Exam Exam Performed**

**Physician's Signature: Date:**

STEP 2: Examine Medical Records

|  |
| --- |

## MEDICAL RECORD EXAMINATION

INSTRUCTIONS: Examine the medical records of the donors (male and female) and note if the systems are normal/abnormal below:

|  | CHECK AS APPROPRIATE | | |  |
| --- | --- | --- | --- | --- |
|  | NORMAL | ABNORMAL | NOT  NOTED IN MEDICAL HISTORY | ADDITIONAL DETAILS |
| EAR NOSE AND THROAT |  |  |  |  |
| OPHTHALMOLOGICAL |  |  |  |  |
| DERMATOLOGICAL |  |  |  |  |
| CARDIOVASCULAR SYSTEM |  |  |  |  |
| RESPIRATORY |  |  |  |  |
| GASTRO-INTESTINAL/  ABDOMINAL |  |  |  |  |
| NEUROLOGICAL |  |  |  |  |
| LYMPH NODES |  |  |  |  |
| BREASTS |  |  |  |  |
| REPRODUCTIVE |  |  |  |  |
| MUSCULO-SKELETAL |  |  |  |  |
| OTHER |  |  |  |  |

If any of the systems described above have notable abnormalities, forward the results of the Medical Record Examination to the Study Medical Director for final acceptance/exclusion dispensation.

Forward to Study Medical Director: Yes No

### Study Medical Director Dispensation: Accept Reject

**Study Medical Director's Signature: Date:**

**Physician's Signature: Date:**
