# Supplementary material for: Derivation of Xeno-Free and GMP-Grade Human Embryonic Stem Cells – Platforms for Future Clinical Applications
Source: PLoS One. 2012 Jun 20;7(6):e35325. doi: 10.1371/journal.pone.0035325 (PMC3380026; doi:10.1371/journal.pone.0035325)
Supplement: File S27 — Eligibility Verification Checklist. (DOC) [file pone.0035325.s041.doc]

# File S27

# ELIGIBILITY VERIFICATION CHECKLIST CRF

THE DERIVATION OF NEW HUMAN EMBRYONIC STEM CELL LINES FOR CLINICAL USE

STUDY TITLE:

NOTE: TO BE COMPLETED DURING THE SCREENING VISIT FOR EACH POTENTIAL STUDY SUBJECT.

| **ITEM #** | CRITERIA | **MEETS REQUIREMENT?**  **(Check One)** | |
| --- | --- | --- | --- |
|  |  | **YES** | **NO** |
| INCLUSION | | | |
| 1 | Donor couple is married or has signed a legal notarized agreement to parent a child through IVF. |  |  |
| 2 | Donor couple has signed Informed Consents and is willing to donate their excess embryos to the research program. |  |  |
| 3 | Donor couple has embryos in IVF storage for ≥ 5 years and have no desire to utilize the embryos in the future in order to obtain additional children. |  |  |
| 4 | Donor couple has tested negative for infectious diseases, as described in the LABORATORY TESTS PERFORMED PRIOR TO IVF CRF. |  |  |
| 5 | Donor couple has completed an interview with the IVF staff. |  |  |
| 6 | Donor couple has undergone a medical exam by the IVF staff. |  |  |
| 7 | Donor couple has given urine and blood samples for testing for sexually transmitted diseases (gonorrhea, Chlamydia, and syphilis). |  |  |
| 8 | Donor couple has had blood drawn for archiving purposes. |  |  |
| 9 | Donor couple has given a full medical history to the IVF staff and is willing to have their private doctor/health fund contacted to verify their health history. |  |  |
| 10 | Donor couple has no genetic or hereditary diseases that may prevent them for donating to the program. |  |  |
| 11 | The embryos are of sufficient quality that they may be used in the program and may potentially give rise to new human embryonic stem cell lines. |  |  |
| EXCLUSION | | | |
| 12 | Donor couple has no criteria that may exclude them from the study, as described in the Exclusion Criteria CRF. |  |  |

DONORS MEET INCLUSION CRITERIA AND HAVE NO EXCLUSION CRITERIA THAT WOULD PREVENT THEM FROM BEING INCLUDED IN THIS STUDY: YES NO

RESEARCH STAFF SIGNATURE:__________________________________

DATE: ________________________
