# Supplementary material for: Derivation of Xeno-Free and GMP-Grade Human Embryonic Stem Cells – Platforms for Future Clinical Applications
Source: PLoS One. 2012 Jun 20;7(6):e35325. doi: 10.1371/journal.pone.0035325 (PMC3380026; doi:10.1371/journal.pone.0035325)
Supplement: File S28 — Exclusion Criteria. (DOC) [file pone.0035325.s042.doc]

## EXCLUSION CRITERIA - CRF

DIRECTIONS: EXCLUDE DONOR IF ANY OF THE EXCLUSION CRITERIA LISTED BELOW ARE MARKED WITH A “YES” ANSWER AND THE STUDY MEDICAL DIRECTOR, UPON EVALUATION, EXCLUDES THE DONOR FROM THE STUDY:

| **EXCLUSION CRITERIA (CHECK “YES” ANSWERS)** | | | | |
| --- | --- | --- | --- | --- |
| NUMBER | CRITERIA | NO | YES | MEDICAL DIRECTOR APPROVAL AND DISPOSITION |
| **Medications Taken** | | | | |
| 1 | Donor has taken, or donor is now taking any medications listed on the Medication Deferral List (Appendix 1) within a month of IVF. | M  F | M  F |  |
| 2 | Donor currently taking an antibiotic or took an antibiotic that is listed in the Medical Deferral List (Appendix 1). | M  F | M  F |  |
| 3 | Donor currently is taking any other medication for any type of infection. | M  F | M  F |  |
| 4 | Donor received clotting factor concentrates and Medical Director excluded the donor from the study. | M  F | M  F |  |
| 5 | Donor took Tegison (etretinate) or Soriatane (Acitretin) for psoriasis. | M  F | M  F |  |
| 6 | Donor had vaccinations/immunizations clearly marked with Permanent Deferral within a month of IVF. | M  F | M  F |  |
| 7 | Donor had received treatment with any of the following human pituitary gland hormones: TSH, ACTH, Prolactin, LH, FSH, Antidiuretic hormone, or Oxytocin. | M  F | M  F |  |
| 8 | Donor took Accutane (isotretinoin), Proscar (Finasteride) or Propecia (finasteride) within 1 month of IVF. | M  F | M  F |  |
| 9 | Donor had taken aspirin for cold/flu symptoms prior to IVF (does not include aspirin taken for IVF purposes). | M  F | M  F |  |
| 10 | In the 12 months prior to IVF, donor had been given rabies shots or hepatitis B immune globulin (HBIG). | M  F | M  F |  |
| 11 | Donor took injectable steroids. | M  F | M  F |  |
| 12 | Donor or sexual partner had a transplant or other medical procedure that involved being exposed to organs, tissue, or living cells from an animal. | M  F | M  F |  |
| **Diseases and Conditions** | | | | |
| 13 | Donor currently is suffering or has suffered in the past from any diseases currently on the Disease Deferral List (Appendix 2). | M  F | M  F |  |
| NUMBER | CRITERIA | NO | YES | MEDICAL DIRECTOR APPROVAL AND DISPOSITION |
| 14 | Donor is at risk, had, or has a close family member that has/had Cretzfeldt-Jacob Disease or vCJD. | M  F | M  F |  |
| 15 | Donor tested positive for HIV, or partner tested positive for HIV, or had symptoms of HIV. | M  F | M  F |  |
| 16 | Donor has been a carrier of Hepatitis B or C, or tested positive for hepatitis. | M  F | M  F |  |
| 17 | Donor had a generalized autoimmune disease, including systemic lupus erythematosus and/or multiple sclerosis. | M  F | M  F |  |
| 18 | Donor received blood transfusions from any area **exclusive** of the UK within 12 months of IVF. | M  F | M  F |  |
| 19 | Since 1980, donor had received a transfusion of blood, platelets, plasma, cryoprecipitate, or granulocytes in the **U.K.** (England, Northern Ireland, Scotland, Wales, the Isle of Man, the Channel Islands, Gibraltar, or the Falkland Islands). | M  F | M  F |  |
| 20 | Donor was cancer-treated with chemotherapy, hormonal therapy (injectable, human or animal-derived) or immunotherapy NLT 5 years before IVF (even with no cancer recurrence).. Specific cancers include leukemia or lymphoma, including Hodgkin’s Disease. | M  F | M  F |  |
| 21 | Donor had clotting disorder, or taking coumadin or heparin. | M  F | M  F |  |
| 22 | For those donors with diabetes, donors who, since 1980, received an injection of bovine insulin made from cattle from the UK or were treated with Ultralente Iletin or Ultralente Insulin. | M  F | M  F |  |
| 23 | Donor had/has hemochromatosis. | M  F | M  F |  |
| 24 | Donor has hepatitis or jaundice not caused by medications, Gilbert Disease, bile duct obstruction, alcohol use, gallstones, or trauma to the liver within 5 years of IVF. | M  F | M  F |  |
| 25 | Donor had/has syphilis, gonorrhea, or other sexually transmitted disease | M  F | M  F |  |
| 26 | Donor had Brucellosis/Tuberculosis within 10 years of IVF. | M  F | M  F |  |
| 27 | Donor at risk for Chagas Disease or babesiosis. | M  F | M  F |  |
| 28 | Donor had a human or animal bite (if skin broken) within a year of IVF. | M  F | M  F |  |
| NUMBER | CRITERIA | NO | YES | MEDICAL DIRECTOR APPROVAL AND DISPOSITION |
| 29 | Donor has, had, or took medications for Myasthenia Gravis. | M  F | M  F |  |
| 30 | Donor had angina, a heart attack, bypass surgery, or angioplasty within 1 year of IVF. | M  F | M  F |  |
| 31 | Donor had symptoms of malaria. | M  F | M  F |  |
| 32 | Donor did not have symptoms of malaria, but it has been less than 3 years since donor had symptoms of malaria, donor was a permanent resident of, immigrated from, or visited a country for more than 6 months that is endemic for Malaria, or it has been less than 2 years since donor visited areas north of Seoul, South Korea, for 6 months or less, or it has been less than 1 year since donor returned from any other area where malaria is prevalent provided that donor stayed 6 months or less (refer to Malaria Risk Countries List). | M  F | M  F |  |
| 33 | Donor had lymphadenopathy, night sweats, weight loss, and fever within 1 month of IVF. | M  F | M  F |  |
| 34 | Donor suffered from a serious illness- i.e. Heart disease, bleeding tendency, diabetes, surgery, etc…within 1 year of IVF. | M  F | M  F |  |
| 35 | Donor suffered from an acute or chronic health problem or genetic disease that may cause Permanent Deferral (check Diseases list). | M  F | M  F |  |
| 36 | Donor had a pregnancy or birth within 6 weeks prior to IVF. |  |  |  |
| 37 | Donor had epilepsy or seizures within 3 months of IVF. | M  F | M  F |  |
| 38 | Donor had chest pain, stroke or TIA one year prior to IVF. | M  F | M  F |  |
| 39 | Donor had cancer, a blood disease, or a bleeding problem. | M  F | M  F |  |
| 40 | Donor had cold, flu, infection, or cough within 72 hours of IVF. | M  F | M  F |  |
| NUMBER | CRITERIA | NO | YES | MEDICAL DIRECTOR APPROVAL AND DISPOSITION |
| 41 | Donor had lung disease. | M  F | M  F |  |
| 42 | Donor’s close family member had transplant or other medical procedure that involved being exposed to organs, tissue, or living cells from an animal **and** donor had been repeatedly exposed to blood, saliva, or other body fluids from these individuals through deep kissing, shared toothbrushes, razors, needles, open wounds, or sores. | M  F | M  F |  |
| **Behaviors** | | | | |
| 43 | Donor received payment for sexual relationships. | M  F | M  F |  |
| 44 | Donor had sexual contact with men (if donor is male). |  |  |  |
| 45 | Donor was born in or had sexual relations with, or lived for MORE THAN A YEAR in a country where HIV is prevalent since 1977 (Africa, except North and South Africa; South East Asia, the Caribbean Islands, Cameroon, Central African Republic, Chad, Congo, Equatorial Guinea, Gabon, Niger, or Nigeria). | M  F | M  F |  |
| 46 | Donor used illicit drugs or steroids intravenously or by sniffing, or had sexual relations with anyone who had. | M  F | M  F |  |
| 47 | Donor had sexual contact, even once, with anyone who has ever used a needle to take drugs for 12 months prior to IVF. | M  F | M  F |  |
| 48 | Donor has been in close contact with someone who had viral hepatitis or jaundice within 1 year of IVF. | M  F | M  F |  |
| 49 | Donor had used cocaine or other street drugs through the nose. | M  F | M  F |  |
| 50 | Donor had a tattoo/acupuncture/permanent make up/piercing or needle stick within 12 months before IVF and donor cannot guarantee that the needles were sterile. | M  F | M  F |  |
| 51 | In the 12 months prior to IVF, donor’s open/broken skin or mucous membranes were in contact with someone else’s blood. | M  F | M  F |  |
| 52 | Donor’s skin had been stuck by something that may have been contaminated with blood or body fluids within 12 months prior to IVF. | M  F | M  F |  |
| 53 | Donor was held in jail, prison, or a psychiatric hospital for more than 72 hours within the year prior to IVF. | M  F | M  F |  |
| 54 | Donor received blood, an organ, skin graft, or other tissue transplant from a human donor within 12 months of IVF. | M  F | M  F |  |
| 55 | Donor does not feel healthy on day of interview. | M  F | M  F |  |
| 56 | Donor refuses to sign Informed Consent or does not agree to all conditions of the protocol. | M  F | M  F |  |
| **Countries** | | | | |
| NUMBER | CRITERIA | NO | YES | MEDICAL DIRECTOR APPROVAL AND DISPOSITION |
| 57 | Donor lived for a cumulative period of 3 months or more in the UK between 1980-1996 (i.e. England, Wales, Gibralter, Northern Ireland, the Isle of Man, Channel Islands, Scotland, or the Falkland Islands). | M  F | M  F |  |
| 58 | If in the military (or dependant thereof) between 1980 – 1996, donor has been stationed in Europe (i.e. Albania, Austria, Belgium, Bosnia-Herzegovina, Bulgaria, Croatia, Czech Republic, Denmark, Federal Republic of Yugoslavia, Finland, France, Germany, Greece, Hungary, Italy, Liechtenstein, Luxembourg, Macedonia, Netherlands, Norway, Poland, Portugal, Republic of Ireland, Romania, Slovak Republic, Slovenia, Spain, Sweden, Switzerland, Turkey, United Kingdom) for 6 months or more.  and  a. from 1980 – 1990, stationed in Germany, UK, Belgium or the Netherlands.  b. from 1980 – 1996, stationed in Greece, Turkey, Spain, Portugal, or Italy. | M  F  M  F  M  F | M  F  M  F  M  F |  |
| 59 | Donor lived for 5 years or more in any European country from 1980 – present. | M  F | M  F |  |
| 60 | Donor lived for 3 months or more (cumulative) in the UK between 1980 – 1996. | M  F | M  F |  |
| 61 | Donor, if in the military, was stationed for more than 5 years or more (cumulative), between the years of 1980 – present, in France. | M  F | M  F |  |
| 62 | Donor had spent 10 years or more in Ireland, France, or Portugal since 1980. | M  F | M  F |  |
| **EMBRYO QUALITY** | | | | |
| 1 | Embryo was created using a donor oocyte. |  |  |  |
| 2 | Embryo was created using donor sperm. |  |  |  |

**STUDY MEDICAL DIRECTOR’S DISPENSATION: APPROVE**

**DISAPPROVE**

**STUDY MEDICAL DIRECTOR’S SIGNATURE/DATE: _________________________________**
