# Supplementary material for: Derivation of Xeno-Free and GMP-Grade Human Embryonic Stem Cells – Platforms for Future Clinical Applications
Source: PLoS One. 2012 Jun 20;7(6):e35325. doi: 10.1371/journal.pone.0035325 (PMC3380026; doi:10.1371/journal.pone.0035325)
Supplement: File S29 — Medication Questionnaire. (DOC) [file pone.0035325.s043.doc]

# MEDICATION DEFERRAL LIST - CRF

1. **DID DONORS TAKE MEDICATIONS WITHIN A MONTH PRIOR TO EMBRYO DONATION? YES NO IF SO, WHICH DONOR AND WHICH MEDICATIONS?** ___**M**_________________________________________________________________________________________

_____________________________________________________________________________________________

___**F**______________________________________________________________________________________________________________________________________________________________________________________

1. **DID DONORS TAKE MEDICATIONS WITHIN A MONTH PRIOR TO IVF (REFERS TO CYCLE/S IN WHICH EMBRYOS ARE DONATED TO THIS RESEARCH)? YES NO IF SO, WHICH DONOR, DATES OF CYCLES, AND WHICH MEDICATIONS?** ___**M**_________________________________________________________________________________________

_____________________________________________________________________________________________

___**F**______________________________________________________________________________________________________________________________________________________________________________________

1. **ARE ANY OF THE MEDICATIONS LISTED ABOVE FOUND IN THE MEDICATION TEMPORARY DEFERRAL LIST, APPENDIX 3^+^? YES NO IF SO, WHICH DONOR AND WHICH MEDICATIONS?**

___**M**_________________________________________________________________________________________

_____________________________________________________________________________________________

___**F**_____________________________________________________________________________________________________________________________________________________________________________________

(IF ANY MEDICATIONS WERE TAKEN THAT INDICATE TEMPORARY DEFERRAL IS REQUIRED –AS LISTED IN APPENDIX 3^+^, THE MEDICAL DIRECTOR’S DISPENSATION IS REQUIRED).

1. **HAVE DONORS EVER TAKEN THE FOLLOWING MEDICATIONS? CONSIDER PERMANENTLY DEFERRING ALL PATIENTS WHO HAVE EVER TAKEN ANY OF THE FOLLOWING MEDICATIONS.**

**PERMANENT DEFERRAL LIST**

| **MEDICATION** | **ALTERNATE NAMES** | **USED TO TREAT** | **NOT**  **TAKEN** | **TAKEN** |
| --- | --- | --- | --- | --- |
| Acitretin | Soriatane | psoriasis | M  F | M  F |
| Alkeran | melphalan | cancer | M  F | M  F |
| Ambenonium |  | Antimyas-  themic | M  F | M  F |
| Aminoglutethemide |  | prostate cancer | M  F | M  F |
| Aricept | Donepezil HCl | dementia | M  F | M  F |
| Avlosulfan |  | HIV/AIDS | M  F | M  F |
| Azathioprine | Imuran | rheumatoid arthritis; SLE | M  F | M  F |
| Azulfidine | sulfasalazine | Rheumatoid arthritis | M  F | M  F |
| B663 | Clofazimine,  Lamprene | tuberculosis | M  F | M  F |
| Bicitra | Potassium citrate | hypokalemia | M  F | M  F |
| Blenoxane | Bleomycine sulfate | Testicular cancer | M  F | M  F |
| Busulfan |  | Chemo-  therapy | M  F | M  F |
| Calusterone | Methosarb | Anabolic steroid | M  F | M  F |
| Casodex | Bicalutamide | Prostate cancer | M  F | M  F |
| Cedilanid | Deslanoside | Congestive heart failure | M  F | M  F |
| CeeNu | Lomustine,  CCNU | cancer | M  F | M  F |
| Cephulac | Lactulose,  Chronulac | Laxative used in hepatic encephalopa-thy | M  F | M  F |
| Chlorambucil |  | cancer | M  F | M  F |
| Chronulac | Lactulose,  Cephulac | Laxative used in hepatic encephalopa-thy | M  F | M  F |
| Clofaximine |  | Anti-leprosy | M  F | M  F |
| Cotazyme | panocase | Pancrea-lipase | M  F | M  F |
| Crystodigin | Digoxin,  Digitalis, Digitoxin, Lanoxin,  Gitaligin, Gitalis, Purodigin | Congestive heart failure | M  F | M  F |
| Cyclophosphamide | Cytoxan,  Neosar | leukemia | M  F | M  F |
| Cytadren | Aminogluteth-imide | Cancer treatment | M  F | M  F |
| Cytoxan | Cyclophosph-  Amide,  Neosar | leukemia | M  F | M  F |
| Demser | Metyrosine | hypertension | M  F | M  F |
| Desmopressin | Concentraid, DDVP, Stimate | To decrease urine production | M  F | M  F |
| Desoxycorti-costerone |  | Hormone mineralocorti-coid | M  F | M  F |
| Diapid (bovine & porcine sources) | Vasopressin | Improve bladder control | M  F | M  F |
| Diasone | sulfoxone | Hansen’s disease | M  F | M  F |
| Diethylstilbestrol | DES | Prevent miscarriages | M  F | M  F |
| Digitalis, Digitoxin, Digoxin | Lanoxin, Crystodigin, Gitaligin, Gitalis, Purodigin | Congestive heart failure | M  F | M  F |
| Dilatrate-SR | Isosorbide | angina | M  F | M  F |
| Dipentum | Olsalazine sodium | Inflammatory bowel disease, ulcerative colitis | M  F | M  F |
| Doca Acetate | Deoxyxortisone | hypertension | M  F | M  F |
| Dolophine | Methadone | Narcotic analgesis | M  F | M  F |
| Donepezil HCl, | Aricept | dementia | M  F | M  F |
| Dronabinol | Marinol | Nausea, stimulates appetite | M  F | M  F |
| Duotrate | Pentaerythritol tetranitrate | antiangina | M  F | M  F |
| EHDP | Etidronate disodium,  Didronel | Heterotopic ossification | M  F | M  F |
| Emcyt | Taxoteryl | Antineo-plastic | M  F | M  F |
| Etidronate | Didronel | Paget’s disease, bone problems | M  F | M  F |
| Etoposide | Eposin  Etophos, Vepesid, VP-16 | Chemo-therapy | M  F | M  F |
| Etretinate | Tegison | psoriasis | M  F | M  F |
| Fialuridine | FIAU | Hepatitis B | M  F | M  F |
| Florinef | Fludrocortisone | Addison’s disease | M  F | M  F |
| Gitaligin | Digitalis,  Gitalin, Lanoxin, Digoxin, Purodigin | Congestive heart failure | M  F | M  F |
| Growth Hormone (human source) | somatropin | Hypopituit-arism | M  F | M  F |
| Humagro | Homeopathic growth hormone | Increases muscle mass | M  F | M  F |
| Hydrea | Oral hyudroxyurea | Sickle cell disease | M  F | M  F |
| Imuran | azathioprine | rheumatoid arthritis; SLE | M  F | M  F |
| Insulin - bovine |  | diabetes | M  F | M  F |
| Interferons: Alfa-2A, Alfa-2B, Alfa N3, Gamma-1B |  | Anti-tumor | M  F | M  F |
| Kayexalate | Sodium polystyrene sulphonate | Intestinal necrosis in uremic patients | M  F | M  F |
| Koate |  | Antihemophic factor | M  F | M  F |
| Lamprene | clofazimine | Antibacterial  Antimyco-bacterial | M  F | M  F |
| Lanoxin | Digoxin  Digitalis, Digitoxin  Crystodigin; Gitaligin, Gitalin, Purodigin | Congestive heart failure | M  F | M  F |
| Lantoside C |  | Cardiac correction | M  F | M  F |
| Leflunomide | Arava | Rheumatoid arthritis | M  F | M  F |
| Leucovorin | Citrovorum, Wellcovorin, follinic acid | Anemia  (given along with anti-  neoplastics) | M  F | M  F |
| Leukeran | chlorambucil | Anti-neoplastic | M  F | M  F |
| Leuprolide | Lupron | Prostate cancer, endometrio-sis, uterine fibroid  **Ok if used for infertility | M  F | M  F |
| Levophed | Norepinephrine bitartrate | To increase blood pressure | M  F | M  F |
| Lomustine |  | Chemo-therapy | M  F | M  F |
| Lypressin |  | diabetes | M  F | M  F |
| Lysodren | Mitotane | Cushing’s disease  Chemo-therapy | M  F | M  F |
| Marinol | dronabinol | Appetite stimulant for HIV/AIDS;  Nausea and vomiting in chemo-therapy | M  F | M  F |
| Matulane | procarbazine | cancer | M  F | M  F |
| Mecaptopurine | 6-MP | Immuno-suppression for kidney transplan-tation | M  F | M  F |
| Melphalan |  | Antineo-plastic | M  F | M  F |
| Mercaptopurine | Puri-Nethol | cancer | M  F | M  F |
| Mestinon | Pyridostigmine bromide | Myasthenia gravis | M  F | M  F |
| Methosarb | Calusterone | Anabolic steroid | M  F | M  F |
| Methyl CCNU | Semustine | Rectal cancer | M  F | M  F |
| Mitomycin | Mytomycin | Chemo-therapy | M  F | M  F |
| Mitotane | Lysodren | Adrenal cancer | M  F | M  F |
| Mutamycine | aminoglycoside | Anti-neoplastic | M  F | M  F |
| Mycobutin | Rifabutin | Antibacterial,  Antimyco-bacterial | M  F | M  F |
| Myleran | Busulfan | Antineo-plastic | M  F | M  F |
| Mytelase |  | Myasthenia gravis | M  F | M  F |
| Neostigmine | Prostigmin | Myasthenia gravis | M  F | M  F |
| Olsalazine | Dipentum | Ulcerative colitis | M  F | M  F |
| Phentolamine | Regitine | Erectile dysfunction | M  F | M  F |
| Priscoline | tolazoline | Persistent pulmonary hypertension of the newborn | M  F | M  F |
| Procarbazine | Matulane | Hodgkin’s disease | M  F | M  F |
| Profilate |  | Antihemo-philic factor | M  F | M  F |
| Proplex |  | Antihe-morrhagic | M  F | M  F |
| Prostigmin |  | Opthalmic conditions and myasthenia gravis | M  F | M  F |
| Purinethol | mercaptopurine | leukemia | M  F | M  F |
| Purodigin | Digitalis, Digitoxin  Crystodigin, Gitalin, Gitaligis | Cardiac Glycoside | M  F | M  F |
| Pyridostigmine | Mestinon | Myasthenia gravis | M  F | M  F |
| Regitine | phentolamine | Erectile dysfunction | M  F | M  F |
| Regonal |  | Anticholinesterase (for myasthenia gravis) | M  F | M  F |
| Retrovir | AZT, zidovudine | AIDS, HIV | M  F | M  F |
| Rifabutin | Mycobutin | Mycobac-terium avium complex in AIDS patients | M  F | M  F |
| Roferon-A | Peg anti-Interferon | Hepatitis C, leukemia, Kaposis’s sarcoma, CML | M  F | M  F |
| Semustine | Methyl CCNU | Anti-cancer | M  F | M  F |
| Sodium Polystyrene | SPS, Kayexalate | Increases blood potasssium | M  F | M  F |
| Soriatane | Aciretin | psoriasis | M  F | M  F |
| Sub-Quin |  | Antiarrhyth-mic | M  F | M  F |
| Sulfasalazine | azulfidine | Ulcerative colitis, Crohn’s disease, rheumatoid arthritis | M  F | M  F |
| Sulfoxone | Diasone | Inflammatory bowel disease, rheumatoid arthritis | M  F | M  F |
| TACE |  | antineo-  plastic | M  F | M  F |
| Tegison | etretinate | psoriasis | M  F | M  F |
| Teslac | Testolactone | Anabolic steroid | M  F | M  F |
| Thioguanine | Lanvis | Chemo-therapy | M  F | M  F |
| Ultralente Iletin Ultralente Insulin  *made from beef or pork |  | diabetes | M  F | M  F |
| Vasopressin | Diapid, Arginin, AVP, ADH | Antidiuretic hormone | M  F | M  F |
| VePesid | Etoposide, Etopophos., Toposar, VPL-16 | Antineo-plastic | M  F | M  F |
| Wellcovorin | Leucovorin | Cancer treat-ment | M  F | M  F |
| Zidovudine | AZT, Retrovir | AIDS, HIV | M  F | M  F |
| Zonegran | Zonisamide | epilepsy | M  F | M  F |

**CONSIDER PERMANENTLY DEFERRING DONORS WHO HAVE TAKEN ANY OF THE MEDICATIONS LISTED ABOVE.**

1. **MEDICATIONS TAKEN PRIOR TO EMBRYO DONATION THAT INDICATE THAT A TEMPORARY DEFERRAL IS REQUIRED.**
2. **MEDICATIONS TAKEN PRIOR TO EMBRYO DONATION THAT INDICATE THAT A PERMANENT DEFERRAL IS REQUIRED.**

**ACCEPT REJECT**

**STUDY MEDICAL DIRECTOR’S SIGNATURE _____________________________**

**DATE ________________________**
