# Supplementary material for: Derivation of Xeno-Free and GMP-Grade Human Embryonic Stem Cells – Platforms for Future Clinical Applications
Source: PLoS One. 2012 Jun 20;7(6):e35325. doi: 10.1371/journal.pone.0035325 (PMC3380026; doi:10.1371/journal.pone.0035325)
Supplement: File S30 — Disease Deferral List. (DOC) [file pone.0035325.s044.doc]

# DISEASE DEFERRAL LIST - CRF

**DO DONORS SUFFER FROM ANY DISEASES? YES NO IF YES, WHICH DONOR AND WHICH DISEASE?**

**__________________________________________________________________________________________________________________________________________________________________**

**(CHECK DISEASES AGAINST LIST OF MEDICAL CONDITIONS THAT MAY PERMANENTLY DEFER THE DONOR, BELOW)**

**CONSIDER PERMANENTLY DEFERRING ALL PATIENTS WHO ARE SUFFERING FROM THE FOLLOWING (MARKED “YES”, BELOW):**

Circle one: M/F (Note: Complete one CRF for each donor)

| **MEDICAL CONDITION** | **NO** | **YES** | **IF YES, IS DISEASE** | |
| --- | --- | --- | --- | --- |
|  |  |  | **HEREDITARY** | **INFECTIOUS** |
| Addison’s Disease |  |  |  |  |
| Adrenal Disorder |  |  |  |  |
| Adrenal Glands Absent |  |  |  |  |
| Adrenal-Cortical Syndrome |  |  |  |  |
| AIDS |  |  |  |  |
| Alcoholic Cirrhosis |  |  |  |  |
| ALS |  |  |  |  |
| Autoimmune Disease |  |  |  |  |
| Babesiosis |  |  |  |  |
| Bone Disorders (tumerous) |  |  |  |  |
| Cardiomyopathy |  |  |  |  |
| Chagas’ Disease |  |  |  |  |
| Chronic Granulomatosis |  |  |  |  |
| CJD, nvCJD, or vCJD |  |  |  |  |
| Congenital Coag. Factor Deficiencies |  |  |  |  |
| Coccidiomycosis, Disseminated or Extrapulmonary |  |  |  |  |
| Collagen Vascular Disease |  |  |  |  |
| Congestive Heart Failure |  |  |  |  |
| Crest Syndrome |  |  |  |  |
| Crohn’s Disease |  |  |  |  |
| Dermatitis Herpetiformis |  |  |  |  |
| Dermatomyositis |  |  |  |  |
| Diabetes, if treated by bovine insulin; Diabetes Insipidus |  |  |  |  |
| Dura Matter Transplant |  |  |  |  |
| Ehlers Danlos Syndrome |  |  |  |  |
| Emphysema |  |  |  |  |
| Fatal Familial Insomnia |  |  |  |  |
| G6PD Deficiency |  |  |  |  |
| Genital herpes |  |  |  |  |
| Gerstmann-Straussler-Scheinlzer |  |  |  |  |
| Hairy Cell leukemia |  |  |  |  |
| Hemochromatosis |  |  |  |  |
| Hemophilia |  |  |  |  |
| Hemolytic Anemias |  |  |  |  |
| Hepatitis |  |  |  |  |
| Hereditary Spherocytosis |  |  |  |  |
| HIV Test confirmed positive |  |  |  |  |
| Hodgkin’s Disease |  |  |  |  |
| Huntington’s Disease |  |  |  |  |
| Isosporiasis |  |  |  |  |
| Jacob-Creutzfeldt Disease |  |  |  |  |
| Kaposi’s Sarcoma |  |  |  |  |
| Kidney Transplant |  |  |  |  |
| Leishmaniasis |  |  |  |  |
| Leprosy |  |  |  |  |
| Leukemia |  |  |  |  |
| Systemic Lupus (and Erythmatosis) |  |  |  |  |
| Melanoma |  |  |  |  |
| Mickulicz Syndrome |  |  |  |  |
| MS |  |  |  |  |
| Myasthenia Gravis |  |  |  |  |
| Mycosis Fungoides |  |  |  |  |
| Spherocytosis |  |  |  |  |
| Thalassemia Major |  |  |  |  |
| Organ Transplant (Dura Matter) |  |  |  |  |
| Pacemaker |  |  |  |  |
| Paget’s Disease of the Breast |  |  |  |  |
| Parvovirus |  |  |  |  |
| Paroxysmal Nocturnal Hemoglobinuria |  |  |  |  |
| Pemphigoid/Pemphigus Vulgaris |  |  |  |  |
| PCP |  |  |  |  |
| Polyucythemia |  |  |  |  |
| Polymyalgia Rheumatica |  |  |  |  |
| Polymyositis |  |  |  |  |
| Porphyria Cutanea Tarda |  |  |  |  |
| Progressive Multifocal Leukoencaphalopathy |  |  |  |  |
| Progressive Systemic Sclerosis |  |  |  |  |
| Q-Fever |  |  |  |  |
| Regional Enteritis |  |  |  |  |
| Reiter’s Syndrome |  |  |  |  |
| Renal Failure |  |  |  |  |
| Reticuloendotheliosis |  |  |  |  |
| SARs |  |  |  |  |
| Scleroderma |  |  |  |  |
| Syphilis |  |  |  |  |
| Sickle Cell Disease |  |  |  |  |
| Sjogren’s Syndrome |  |  |  |  |
| Transverse Myelitis |  |  |  |  |
| Trypanosomiasis |  |  |  |  |
| Ulcerative Colitis (unless post-colectomy and feeling well) |  |  |  |  |
| Vaccinia (smallpox) |  |  |  |  |
| Von Willebrand’s Disease |  |  |  |  |
| West Nile Virus |  |  |  |  |

**IF ANY OF THE ABOVE DISEASES ARE CHECKED, THE STUDY MEDICAL DIRECTOR MUST DECIDE TO ACCEPT OR REJECT THE DONOR FROM THE STUDY:**

**ACCEPT REJECT**

**STUDY MEDICAL DIRECTOR’S SIGNATURE __________________________**

**DATE _________________**
