# Supplementary material for: Derivation of Xeno-Free and GMP-Grade Human Embryonic Stem Cells – Platforms for Future Clinical Applications
Source: PLoS One. 2012 Jun 20;7(6):e35325. doi: 10.1371/journal.pone.0035325 (PMC3380026; doi:10.1371/journal.pone.0035325)
Supplement: File S31 — Appendix 3, Medication Deferral List. (DOC) [file pone.0035325.s045.doc]

# MEDICATION TEMPORARY AND PERMANENT DEFERRAL LIST

# *Temporary deferral*

# Permanent deferral

| **Drug** | **Acceptable** | **Note** |
| --- | --- | --- |
| 5-FU | No, temporary deferral if for cancer. Yes, if topical for superficial skin cancer such as basal cell carcinoma. | Antineoplastic. Desquamator |
| A.P.C. Tablets | Yes, if taken for allergies. Defer for 72 hours after symptoms are resolved if taken for cold/flu symptoms or for fever.  Defer 72 hrs for plateletpheresis or sole source platelets. | Analgesic, anti-pyretic, anti-inflammatory |
| A/T/S | Defer 24 hrs after course completed and feel well. If IV or IM defer 1 wk.  Yes, if for acne. | Antibiotic |
| Accolate | Yes | Anti-asthmatic |
| Accupril | Yes, for stable hypertension | Ace Inhibitor |
| *Accutane (isotretinoin)* | *Defer for one month after course of therapy completed.* | *Medication for severe acne. Synthetic form of Vitamin A.* |
| ACE Inhibitors | Yes, for hypertension.  Defer if for heart disease. | Antihypertensive, ACE inhibitor |
| Acebutolol | Yes, if for hypertension. | Beta-blocker, anti-hypertensive |
| Acetaminophen | Yes, if taken for pain.  Yes, if taken for allergies. Defer for 72 hours after symptoms are resolved if taken for cold/flu symptoms or for fever. | Analgesic, antipyretic. |
| Acetazolamide | Yes. | Anticonvulsant, diuretic and inhibitor of fluid secretion (glaucoma). |
| Aceticyl | Yes, if taken for allergies. Defer for 72 hours after symptoms are resolved if taken for cold/flu symptoms or for fever.  Defer 72 hrs for plateletpheresis or sole source platelets | Analgesic, anti-pyretic,anti-inflammatory |
| Acetohexamide | Yes. | Oral hypoglycemic agent for diabetes. |
| Acetophenazine | Yes, if donor mentally and legally responsible. | Anti-psychotic |
| Acetosalin | Yes, if taken for allergies. Defer for 72 hours after symptoms are resolved if taken for cold/flu symptoms or for fever.  Defer 72 hrs for plateletpheresis or sole source platelets | Analgesic, anti-pyretic,anti-inflammatory |
| *Acetylcysteine* | *Defer until off drug and underlying condition resolved.* | *Mucolytic agent* |
| Acetylsal (Canton) | Yes, if taken for allergies. Defer for 72 hours after symptoms are resolved if taken for cold/flu symptoms or for fever.  Defer 72 hrs for plateletpheresis or sole source platelets | Analgesic, anti-pyretic,anti-inflammatory |
| Achromycin | Yes, if ophthalmic or topical use, or for acne. Otherwise, defer 24 hrs. After course completed and feel well; if IV or IM defer 1 week. | Antibiotic |
| Achrostatin V | Defer until 24 hrs. after course completed and feel well; if IV or IM defer 1 week. | Antifungal, Antibiotic |
| A-Cill | No, wait 24 hrs. after course completed and feel well, if IV or IM defer 1 wk. | Antibiotic |
| Aciphex (Rabeprazole) | Yes, if no evidence of bleeding. | Prevention of stomach ulcer bleeding |
| **Acitretin (Soriatane)** | **Permanent Deferral (teratogenic)** | **Anti-psoriasis** |
| Acon (Vitamin A) | Yes. | Vitamins |
| Acthar | Evaluate underlying condition. | ACTH |
| Acticin | Yes, if taken for allergies. Defer for 72 hours after symptoms are resolved if taken for cold/flu symptoms. | Antihistamine, decongestant |
| Actidil | Yes, if taken for allergies. Defer for 72 hours after symptoms are resolved if taken for cold/flu symptoms. | Antihistamine |
| Actifed | Yes, if taken for allergies. Defer for 72 hours after symptoms are resolved if taken for cold/flu symptoms. | Antihistamine |
| Actigall | Yes. | Gall stone dissolution agent |
| *Activase* | *Defer until therapy discontinued.* | *Tissue plasminogen activator* |
| Actol | Yes, if taken for allergies. Defer for 72 hours after symptoms are resolved if taken for cold/flu symptoms. | Antitussive, expectorant |
| Acular | Yes | NSAID |
| Acyclovir | Yes, if no active lesions. | Antiviral for Herpes virus |
| Adalat | Yes, if for esophageal achalasia, migraine or hypertension; otherwise, no. | Antianginal, calcium channel blocker |
| Adapin | Yes. | Antidepressant |
| Adderal | Yes. | For attention deficit |
| Adeflor Drops | Yes. | Vitamin/Mineral supplement |
| Adipex-8, Adipex-P | Yes. | Amphetamine |
| Adrenalin | Yes, if not daily dose for maintenance but intermittent use. | Bronchodilator |
| Adult Analgesic Pain Reliever (DeWitt) | Yes, if taken for allergies. Defer for 72 hours after symptoms are resolved if taken for cold/flu symptoms or for fever.  Defer 72 hrs for plateletpheresis or sole source platelets | Analgesic, anti-pyretic,anti-inflammatory |
| Advil | Yes, if not severe arthritis pain. Defer plateletpheresis donors 24 hours | Non-steroidal anti-inflammatory. |
| Aerolate (theophylline) | Yes, even if daily dose for maintenance. | Bronchodilator |
| Afrin | Yes, if taken for allergies. Defer for 72 hours after symptoms are resolved if taken for cold/flu symptoms. | Decongestant |
| Afrinol | Yes, if taken for allergies. Defer for 72 hours after symptoms are resolved if taken for cold/flu symptoms. | Decongestant |
| Afrinol Repetabs | Yes, if taken for allergies. Defer for 72 hours after symptoms are resolved if taken for cold/flu symptoms. | Decongestant |
| Agoral | Yes. | Stool Softener |
| Airet (dyphylline) | Yes, even if daily dose for maintenance. | Bronchodilator |
| Akineton | Yes. | Anticholinergic-Parkinson's Disease |
| Albamycin | Defer for 24 hrs. after course completed, if IV or IM defer 1 week. | Antibiotic |
| Albuterol | Yes, even if daily dose for maintenance. | Bronchodilator |
| Alcopar | Defer 1 wk. After course completed and feel well. | Antihelminthic |
| Aldactazide | Yes. | Antihypertensive, diuretic |
| Aldactone | Yes. | Antihypertensive, diuretic |
| Aldochlor | Yes. | Antihypertensive |
| Aldomet | Yes. | Antihypertensive |
| Aldoril | Yes. | Antihypertensive |
| Alka-Seltzer Antacid and Pain Reliever | Yes, if taken for allergies. Defer for 72 hours after symptoms are resolved if taken for cold/flu symptoms or for fever.  Defer 72 hrs for plateletpheresis or sole source platelets | Analgesic, Antipyretic, antacid, ASA |
| Alka-Seltzer Plus Sinus Allergy or Cold Medicine | Yes, if taken for allergies. Defer for 72 hours after symptoms are resolved if taken for cold/flu symptoms or for fever.  Defer 72 hrs for plateletpheresis or sole source platelets | Analgesic, anti-pyretic, anti-inflammatory |
| **Alkeran** | **No, permanent deferral.** | **Antineoplastic agent** |
| Allegra | Yes, if taken for allergies. Defer for 72 hours after symptoms are resolved if taken for cold/flu symptoms. | Antihistamine |
| Allerest | Yes, if taken for allergies. Defer for 72 hours after symptoms are resolved if taken for cold/flu symptoms. | Antihistamine, decongestant |
| Allopurinol | Yes. | Xanthine oxidase inhibitor |
| Alophen | Yes. | Laxative |
| Alpen, Alpen-N | Defer for 24 hrs. after course completed and feel well, if IV or IM defer 1 week. | Antibiotic |
| Alpha Redisal | Yes. | Vitamin B_12_ |
| Alpha/Beta Adrenergic blockers | Yes. | Antihypertensive |
| *Alphadrol* | *No, if for collagen-vascular disease. Otherwise, wait 72 hrs. after course completed if oral or IM.* | *Oral corticosteroid* |
| Alphalin | Yes. | Vitamin A |
| Alphaprodine | Yes, if not abuser. | Narcotic analgesic |
| *Alphatrex* | *Defer 72 hrs after use completed if IV, IM or PO, otherwise, yes.* | *Corticosteroid Anti-inflammatory* |
| Alprazolam | Yes. | Benzodiazepine, antianxiety agent |
| Alprostadil | Yes | Treatment for erectile dysfunction |
| Alseroxylon | Yes. | Antihypertensive |
| Altace | Yes, if for hypertension. No, if for heart disease. | ACE Inhibitor, antihypertensive |
| *Alteplase* | *Defer until therapy discontinued.* | *Tissue plasminogen activator* |
| Alternagel | Yes, if for esophagitis or ulcer disease (pain-free). No, if renal patient. | Antacid |
| Altex | Yes. | Diuretic |
| Aludrox | Yes, if for esophagitis or ulcer disease (pain-free). No, if renal patient. | Antacid |
| Aluminum Nicotinate | Yes. | Cholesterol lowering agent |
| Alupent | Yes, even if daily dose for maintenance. | Bronchodilator |
| Amantadine | Yes. | Antiviral prophylaxis, anti-Parkinson's |
| Amaryl | Yes. | Oral hypoglycemic |
| **Ambenonium** | **No, permanent deferral.** | **Anticholinesterase (myasthenia gravis)** |
| Ambenyl | Yes, if taken for allergies. Defer for 72 hours after symptoms are resolved if taken for cold/flu symptoms. | Antihistamine |
| Ambien | Yes. | Hypnotic, Sedative |
| Ambihar | Defer 1 wk, after course completed and feel well. | Antihelminthic |
| Ambodryl | Yes, if taken for allergies. Defer for 72 hours after symptoms are resolved if taken for cold/flu symptoms. | Antihistamine |
| Amcill | Defer 24 hrs. after course completed and feel well, if IV or IM defer 1 wk. | Antibiotic |
| Amdinocillin | Defer 1 wk. After course completed and feel well. | Antibiotic |
| Amen | No, if cancer patient; otherwise, yes. | Hormone, progestin |
| Amesec | Yes, even if for daily maintenance use. | Bronchodilator, hypnotic, sedative |
| A-MethaPred | Defer 72 hrs. if oral or IM. Yes, if topical, ophthalmic or intra-articular. | Corticosteroid |
| Amicar | Evaluate underlying condition. | Antifibrinolytic agent |
| Amiloride | Yes. | Diuretic |
| **Aminoglutethemide** | **No, permanent deferral.** | **Antiadrenal agent** |
| Aminophyllin | Yes, even if daily dose for maintenance use. | Bronchodilator |
| Aminophylline | Yes, even if daily dose for maintenance use. | Bronchodilator |
| *Aminosalicylic Acid* | *Defer until course of medication completed and disease inactive* | *Antituberculosis drug* |
| Amitid | Yes. | Tricyclic antidepressant |
| Amitril | Yes. | Tricyclic antidepressant |
| Amitriptyline | Yes. | Tricyclic antidepressant |
| Amnestrogen | Yes. | Hormone replacement |
| Amodiaquine | Defer 1 yr. If in malaria area. Otherwise, yes. | Antimalarial |
| Amodrine | Yes, if taken for allergies. Defer for 72 hours after symptoms are resolved if taken for cold/flu symptoms. | Antihistamine |
| Amoxapine | Yes. | Tricyclic antidepressant |
| Amoxicillin | Defer until 24 hrs. after course completed and feel well. Yes, for acne. | Antibiotic |
| Amoxil | Defer until 24 hrs. after course completed and feel well. Yes, for acne. | Antibiotic |
| Amphetamine | Yes. | Stimulant |
| Amphojel | Yes, if ulcer disease pain-free. No, if renal failure patient. | Antacid |
| Ampicillin | No, wait 24 hrs. after course completed and feel well, if IV or IM defer 1 wk. | Antibiotic |
| Amytal | Yes. | Sedative to relieve anxiety and tension |
| Anacin | Yes, if taken for allergies. Defer for 72 hours after symptoms are resolved if taken for cold/flu symptoms or for fever.  Defer 72 hrs for plateletpheresis or sole source platelets | ASA containing analgesic |
| Anadrol-50 | Yes, unless used for Rx of cancer or aplastic anemia. | Anabolic steroid |
| Anafranil | Yes. | Anti-obsessional drug |
| Analval | Yes, if taken for allergies. Defer for 72 hours after symptoms are resolved if taken for cold/flu symptoms or for fever.  Defer 72 hrs for plateletpheresis or sole source platelets | ASA containing analgesic |
| Ananase | Yes. | Anti-inflammatory, reduces edema, eases pain, speeds healing. |
| Anaprox | Yes, for menstrual cramps and arthritis which is under control. Defer plateletpheresis donors 24 hours. | Non-steroidal anti-inflammatory |
| Anaspaz | Yes, if ulcer disease & pain- free. | Belladonna alkaloid, anticholinergic/antispasmodic |
| Anavar | Yes. | Anabolic steroid |
| Ancobon | Yes, if for infections of skin or nail beds. No, if for systemic (generalized) infection; then defer for 1 week after course completed and condition resolved. | Antifungal |
| Android | Yes. | Anabolic steroid |
| Android | Yes, unless for Rx of cancer or aplastic anemia. | Hormone |
| Anexsia (Smith-Kline Beecham) | Yes, if taken for allergies. Defer for 72 hours after symptoms are resolved if taken for cold/flu symptoms or for fever.  Defer 72 hrs for plateletpheresis or sole source platelets | ASA containing analgesic |
| *Anginar* | *No, defer until off medication and free of symptoms.* | *Antianginal* |
| Anhydron | Yes. | Antihypertensive, diuretic |
| Anilerdine | Yes, if not abuser. | Narcotic analgesic |
| Animal Serum Products | Defer 2 weeks after last injection. |  |
| *Anisindione* | *Defer pending medical evaluation with cessation of drug.* | *Anticoagulant* |
| Anisotropine | Yes, ulcer disease is pain-free. | Anticholinergic, antispasmodic |
| Anodynos (Buffington) | Yes, if taken for allergies. Defer for 72 hours after symptoms are resolved if taken for cold/flu symptoms or for fever.  Defer 72 hrs for plateletpheresis or sole source platelets | ASA containing analgesic |
| Ansaid | Yes, if arthritis inactive. Defer plateletpheresis donors 24 hours. | Non-steroidal anti- inflammatory |
| Anspor | Defer until 24 hrs. after course completed and feel well; if IV or IM defer 1 wk. | Antibiotic |
| Antabuse | Defer 1 wk. After off drug. | Indication-alcoholism |
| Antepar | Defer 1 wk. After course completed and feel well. | Antihelminthic |
| Antiminth | Defer 1 wk. After course completed and feel well. | Antihelminthic |
| Antispas | Yes, if ulcer disease is pain-free. | Anticholinergic antispasmodic |
| Antivert | Yes. | Antihistamine for vertigo |
| Antuitrin S | Yes. | Hormone |
| Anturane | Yes. | Uricosuric agent |
| Anusol | Yes. | Indication hemorrhoids |
| Apap | Yes, if taken for allergies. Defer for 72 hours after symptoms are resolved if taken for cold/flu symptoms or for fever. | Antipyretic, anti-inflammatory |
| A-Poxide | Yes. | Tranquilizer |
| Appetabs | Yes. | Amphetamine, stimulant |
| Apresazide | Yes. | Antihypertensive |
| Apresoline | Yes. | Antihypertensive |
| Aqualine | Yes, even if daily dose for maintenance. | Bronchodilator |
| AquaMephyton | Evaluate underlying condition. | Vitamin K |
| Aquamox | Yes. | Diuretic |
| Aquasol A | Yes. | Vitamin A |
| Aquasol E | Yes. | Vitamin E |
| Aquatag | Yes. | Antihypertensive, diuretic |
| Aquatensen | Yes. | Antihypertensive, diuretic |
| *Aralen* | *Defer 1 yr. If in malaria zone. Otherwise, yes.* | *Antimalarial* |
| Arava | No | Anti-Rheumatoid Arthritis Agent. Defer based on history of Rheumatoid Arthritis. |
| Arfonad | Defer 72 hrs. | Ganglionic blocking agent (potent hypotensive) |
| **Aricept (Donepezil HCl)** | **PD** | **Cholinesterase Inhibitor** |
| Aristocort | Defer 72 hrs. if oral or IM, otherwise yes. | Steroid |
| Aristospan | Defer 72 hrs. if oral or IM, otherwise yes. | Steroid |
| Arlidin | Yes, used in peripheral vascular disease. | Vasorelaxant, vasodilator |
| Artane | Yes. | Anti-Parkinsonism |
| Arthritis Pain Formula (Whitehall) | Yes, if taken for allergies. Defer for 72 hours after symptoms are resolved if taken for cold/flu symptoms or for fever.  Defer 72 hrs for plateletpheresis or sole source platelets | ASA containing analgesic |
| Arthritis Strength BC Powder (Block) | Yes, if taken for allergies. Defer for 72 hours after symptoms are resolved if taken for cold/flu symptoms or for fever.  Defer 72 hrs for plateletpheresis or sole source platelets | ASA containing analgesic |
| Arthritis Strength Bufferin (Bristol-Myers) | Yes, if taken for allergies. Defer for 72 hours after symptoms are resolved if taken for cold/flu symptoms or for fever.  Defer 72 hrs for plateletpheresis or sole source platelets | ASA containing analgesic |
| Arthropan liquid | Yes, if taken for allergies. Defer for 72 hours after symptoms are resolved if taken for cold/flu symptoms or for fever.  Defer 72 hrs for plateletpheresis or sole source platelets | Analgesic, antipyretic, anti-inflammatory, ASA |
| Arthrotic | Yes. | Combination of Voltarin and Cytotec, both approved, follow individual guidelines. |
| ASA Enseals (Lilly) | Yes, if taken for allergies. Defer for 72 hours after symptoms are resolved if taken for cold/flu symptoms or for fever.  Defer 72 hrs for plateletpheresis or sole source platelets | ASA containing analgesic |
| ASA Suppositories (Lilly) | Yes, if taken for allergies. Defer for 72 hours after symptoms are resolved if taken for cold/flu symptoms or for fever.  Defer 72 hrs for plateletpheresis or sole source platelets | ASA containing analgesic |
| Asacol | Yes. | Anti-lipemic |
| Asbron | Yes, even if daily dose for maintenance. | Bronchodilator |
| Ascriptin | Yes, if arthritis is inactive.  Yes, if taken for allergies. Defer for 72 hours after symptoms are resolved if taken for cold/flu symptoms or for fever.  Defer 72 hrs for plateletpheresis or sole source platelets | Analgesic, anti-inflammatory anti-pyretic, ASA. |
| Asendin | Yes. | Anti-depressant, tranquilizer |
| Asmolin | Yes, even if daily dose for maintenance. | Bronchodilator |
| Aspergum (Schering-Plough) | Yes, if taken for allergies. Defer for 72 hours after symptoms are resolved if taken for cold/flu symptoms or for fever.  Defer 72 hrs for plateletpheresis or sole source platelets | ASA containing analgesic |
| Aspermin (Buffington) | Yes, if taken for allergies. Defer for 72 hours after symptoms are resolved if taken for cold/flu symptoms or for fever.  Defer 72 hrs for plateletpheresis or sole source platelets | ASA containing analgesic |
| Aspirin | Yes, if taken for allergies. Defer for 72 hours after symptoms are resolved if taken for cold/flu symptoms or for fever.  Defer 72 hrs for plateletpheresis or sole source platelets | Analgesic, anti-pyretic, anti-inflammatory |
| Aspirjen Jr (Jenkins) | Yes, if taken for allergies. Defer for 72 hours after symptoms are resolved if taken for cold/flu symptoms or for fever.  Defer 72 hrs for plateletpheresis or sole source platelets | ASA containing analgesic |
| Aspirmax (Invamed) | Yes, if taken for allergies. Defer for 72 hours after symptoms are resolved if taken for cold/flu symptoms or for fever.  Defer 72 hrs for plateletpheresis or sole source platelets | ASA containing analgesic |
| Aspir-Max (Perrigo) | Yes, if taken for allergies. Defer for 72 hours after symptoms are resolved if taken for cold/flu symptoms or for fever.  Defer 72 hrs for plateletpheresis or sole source platelets | ASA containing analgesic |
| Aspirtab (Dover) | Yes, if taken for allergies. Defer for 72 hours after symptoms are resolved if taken for cold/flu symptoms or for fever.  Defer 72 hrs for plateletpheresis or sole source platelets | ASA containing analgesic |
| Astemizole | Yes, if taken for allergies. Defer for 72 hours after symptoms are resolved if taken for cold/flu symptoms. | Antihistamine |
| Atabrine | Defer 1 yr. If in malaria zone, otherwise yes. | Antimalarial |
| Atarax | Yes, if taken for allergies. Defer for 72 hours after symptoms are resolved if taken for cold/flu symptoms. | Sedative, antihistamine |
| Atenolol | Yes. | Antihypertensive, beta blocker |
| *Athrombin-K* | *Defer pending medical evaluation with cessation of drug.* | *Anticoagulant* |
| Ativan | Yes. | Tranquilizer |
| Atrocholin | Yes, if ulcer disease is pain free. | Anticholinergic antispasmodic |
| Atromid S | Yes. | Cholesterol lowering agent |
| Atropine | Yes, if ulcer disease is pain free. | Anticholinergic antispasmodic |
| Atropisol | Yes, if ulcer disease is pain free. | Anticholinergic antispasmodic |
| Augmentin | Defer until 24 hrs. after course completed and feel well. | Antibiotic |
| Aureomycin | Defer until 24 hrs. after course completed and feel well; if IV or IM defer 1 wk. Yes, if for acne. | Antibiotic |
| Aurothioglucose | Yes, if disease not active at this time. | Anti-rheumatic, anti-inflammatory |
| Avapro | Yes, for BP | Angiotensin blocker |
| AVC | Yes. | Antibiotic, antiseptic Cream/suppository |
| AVC Dienestrol | As above. | (Non-specific vaginal infection) |
| Aventyl | Yes. | Antidepressant |
| *Avodart (dutasteride)* | *No, defer until 6 months after last dose of medication.* | *Used for treatment of benign prostatic hyperplasia in men with enlarged prostate. Potential fetal anomaly to male fetus.* |
| **Avlosulfan** | **No, permanent deferral.** | **Anti-leprosy agent** |
| Axid | Yes, if ulcer disease inactive. | Anti-ulcer agent |
| Axotal | Yes, if taken for allergies. Defer for 72 hours after symptoms are resolved if taken for cold/flu symptoms or for fever.  Defer 72 hrs for plateletpheresis or sole source platelets | ASA + Butalbital |
| Aygestin | Yes. | Progestin agent |
| Azatadine | Yes, if taken for allergies. Defer for 72 hours after symptoms are resolved if taken for cold/flu symptoms. | Antihistamine |
| **Azathioprine** | **No, permanent deferral.** | **Immunomodulator** |
| Azdone Tablets (Central Pharmaceuticals) | Yes, if taken for allergies. Defer for 72 hours after symptoms are resolved if taken for cold/flu symptoms or for fever.  Defer 72 hrs for plateletpheresis or sole source platelets | ASA containing analgesic |
| Azene | Yes. | Anti-anxiety agent |
| Azithromycin | No, defer 24 hours after course completed. Yes for acne. | Antibiotic |
| Azlin | Defer 1 wk. After course completed and feel well. | Antibiotic |
| Azlocillin | Defer 1 wk. After course completed and feel well. | Antibiotic |
| Azmacort (Vancenase) | Yes, even if daily dose for maintenance. | Anti-inflammatory, steroid |
| Azo-Gantanol | Defer 24 hrs after course completed and feel well, if IV or IM defer 1 wk. | Antibiotic, analgesic |
| Azo-Gantrisin | Defer 24 hrs. after course completed and feel well, if IV or IM defer 1 wk. | Antibiotic |
| Azolid | Yes, if arthritis inactive. | Anti-inflammatory |
| Azo-Mandelamine | Defer 24 hrs. after course completed and feel well, if IV or IM defer 1 wk. | Antibiotic |
| Azo-Standard | Defer 24 hrs. after course completed and feel well, if IV or IM defer 1 wk. | Antibiotic, analgesic |
| Azotrex | Defer 24 hrs. after course completed and feel well, if IV or IM defer 1 wk. | Antibiotic, analgesic |
| **Azulfidine** | **No, Permanent Deferral** | **Immune modulator** |
| **B663** | **No, permanent deferral.** | **Anti-leprosy drug** |
| BA-C Tablets | Yes, if taken for allergies. Defer for 72 hours after symptoms are resolved if taken for cold/flu symptoms or for fever.  Defer 72 hrs for plateletpheresis or sole source platelets | ASA containing analgesic |
| Bacampicillin | Defer 24 hrs. after course completed and feel well, if IV or IM defer 1 wk. | Antibiotic |
| Bacarate | Yes. | Anorexic |
| Bacid | Yes, if symptom free  Defer for 72 hours after symptoms are resolved if taken for cold/flu symptoms. | Antidiarrheal |
| Back-Quell (Otis Clapp) | Yes, if taken for allergies. Defer for 72 hours after symptoms are resolved if taken for cold/flu symptoms or for fever.  Defer 72 hrs for plateletpheresis or sole source platelets | ASA containing analgesic |
| Baclofen | Yes. | Muscle relaxant |
| Bactocill(oxacillin) | Defer 24 hrs. after course completed and feel well, if IV or IM defer 1 wk. | Antibiotic |
| Bactrim | Defer 24 hrs. after course completed and well. Accept for acne use. | Antibiotic |
| Baltron | Yes. | Vitamin/mineral |
| Bamo-400 | Yes. | Sedative/hypnotic |
| Banthine | Yes. | Antispasmodic |
| Barbiturates | Yes, evaluate underlying condition. | Sedative, anti-convulsant |
| Basaljel | No, if renal patient; yes, if ulcer disease pain free. | Antacid |
| Baycol (Cerivastatin) | See Cerivastatin |  |
| Bayer 8-hour Timed Release Aspirin (Glenbrook) | Yes, if taken for allergies. Defer for 72 hours after symptoms are resolved if taken for cold/flu symptoms or for fever.  Defer 72 hrs for plateletpheresis or sole source platelets | ASA containing analgesic |
| Bayer Aspirin (Glenbrook) | Yes, if taken for allergies. Defer for 72 hours after symptoms are resolved if taken for cold/flu symptoms or for fever.  Defer 72 hrs for plateletpheresis or sole source platelets | ASA containing analgesic |
| Bayer Children's Aspirin (Glenbrook) | Yes, if taken for allergies. Defer for 72 hours after symptoms are resolved if taken for cold/flu symptoms or for fever.  Defer 72 hrs for plateletpheresis or sole source platelets | ASA containing analgesic |
| Bayer Plus Aspirin Tablets (Glenbrook) | Yes, if taken for allergies. Defer for 72 hours after symptoms are resolved if taken for cold/flu symptoms or for fever.  Defer 72 hrs for plateletpheresis or sole source platelets | ASA containing analgesic |
| BC Cold Powder Multi-Symptom Formula (Block) | Yes, if taken for allergies. Defer for 72 hours after symptoms are resolved if taken for cold/flu symptoms or for fever.  Defer 72 hrs for plateletpheresis or sole source platelets | ASA containing analgesic |
| BC Cold Powder Non-Drowsy Formula (Block) | Yes, if taken for allergies. Defer for 72 hours after symptoms are resolved if taken for cold/flu symptoms or for fever.  Defer 72 hrs for plateletpheresis or sole source platelets | ASA containing analgesic |
| BC Powder (Block) | Yes, if taken for allergies. Defer for 72 hours after symptoms are resolved if taken for cold/flu symptoms or for fever.  Defer 72 hrs for plateletpheresis or sole source platelets | ASA containing analgesic |
| Beclometasone (Beclomet) | Yes, even if daily maintenance dose. | Anti-inflammatory, steroid |
| Beclovent | Yes, even if daily maintenance dose. | Anti-inflammatory, steroid |
| Beconase | Yes, even if daily dose for maintenance. | Anti-inflammatory, steroid |
| Belap | Yes, if ulcer disease pain free. | Anticholinergic antispasmodic, hypnotic/sedative |
| Belladenal | Yes, if ulcer disease pain free. | Anticholinergic antispasmodic, hypnotic/sedative |
| Belladonna | Yes, if ulcer disease pain free. | Antispasmodic |
| Bellafoline | Yes, if ulcer disease pain free. | Anticholinergic antispasmodic, hypnotic/sedative |
| Bellergal | Yes. | Antispasmodic vasoconstrictor |
| Benacen | Yes. | Uricosuric agent |
| Benadryl | Yes, if taken for allergies. Defer for 72 hours after symptoms are resolved if taken for cold/flu symptoms. | Antihistamine |
| Bendectin | Yes, if symptom free. | Anti-emetic antinauseant |
| Bendopa | Yes. | Anti-Parkinsonism |
| Bendro-Flumethiazide | Yes. | Antihypertensive |
| Benemid | Yes. | Anti-inflammatory |
| Bentyl | Yes, if ulcer disease pain free. | Antispasmodic |
| Bentyl Cough Syrup | Yes, if taken for allergies. Defer for 72 hours after symptoms are resolved if taken for cold/flu symptoms. | Antitussive |
| Benylin | Yes, if taken for allergies. Defer for 72 hours after symptoms are resolved if taken for cold/flu symptoms. | Antitussive |
| Benzamycin | Defer 24 hrs. after course completed and feel well; if IV or IM defer 1 wk. Yes, if for acne. | Antibiotic |
| Benzedrex | Yes. | B_2_ Stimulator |
| Benzedrine | Yes. | Amphetamine |
| Benzonatate | Yes. | Anorexiant |
| Benzquinamide | Yes, if symptom free. | Antiemetic |
| Benzthiazide | Yes. | Antihypertensive |
| Benztropine Mesylate (Cogentin) | Yes, if taken for allergies. Defer for 72 hours after symptoms are resolved if taken for cold/flu symptoms. | Anticholinergic, antihistamine |
| Benzyl Benzoate | Defer 48 hrs. after therapy completed. | Topical pediculicide |
| Bephenium Hydroxynapthoate | Defer 1 wk. After treatment completed and well. | Antihelminthic |
| Beramax | Yes. | Diuretic |
| Beta Adrenergic stimulants | Yes. | Antihypertensive |
| Beta blockers | Yes, if for hypertension, migraine headaches. No, if antianginal for angina or arrhythmias. Yes, if for Mitral Valve Prolapse. | Antiarrhythmic, anti-hypertensive |
| Beta Carotene | Yes. | Mineral supplement. |
| Beta-Chlor | Yes. | Hypnotic/sedative |
| Betalin S | Yes. | Vitamin |
| Betaliniz Crystalline | Yes. | Vitamin |
| Betaloc | Yes, if for hypertension | Antihypertensive beta blocker |
| Betamethasone | Defer 72 hrs after use completed if IV, IM or PO, otherwise, yes. | Corticosteroid Anti-inflammatory |
| Betapace | Yes, if not a Rx for ventricular arrythmia. Needs Medical Clearance from family physician | Beta Blocker |
| Betapar (meprednisone) | Defer 72 hrs. if P.O. or IM; otherwise yes. | Corticosteroid Anti-inflammatory |
| Betapen-VK | Defer 24 hrs. after course completed of drug and feel well; or if IM or IV, defer 1 wk. | Antibiotic |
| Betatrex | Defer 72 hrs after use completed if IV, IM or PO, otherwise, yes. | Corticosteroid Anti-inflammatory |
| Betaxolol | Yes. | Anti-glaucoma agent |
| Bethanecol | Yes. | Cholinomimetic (urinary incontinence) |
| Betopic | Yes. | Anti-glaucoma agent |
| Bewon | Yes. | Vitamin |
| Bezafibrate | Yes. | Cholesterol lowering agent. |
| Biaxin | Yes, after complete and off Rx. 24 hr | Antibiotic |
| Bicillin | Defer 2 weeks. After IM injection. If used for sexually transmitted disease, see disease. | Antibiotic |
| Bicitra | No, permanent deferral. | Electrolyte replacement |
| Bilron | Yes. | Vitamin/mineral/bile salt replacement |
| Bio-Anabolic | Yes. | Vitamin supplement |
| Biocef | Defer 24 hrs. after course completed and feel well. | Antibiotic |
| Biopar Forte | Yes. | Vitamin |
| Biphetamine | Yes. | Anorexiant, stimulant |
| Biporiden | Yes. | Anticholinergic, anti-Parkinsonism |
| Bisacodyl | Yes. | Laxative |
| Biscolax | Yes. | Laxative |
| Bisken | Yes, if for hypertension No, defer if for arrhythmia. | Beta-blocker, anti-hypertensive |
| Bismuth Subsalicylate | Yes if symptom free. | Antidiarrheal |
| Bismuth, Pectin and paregoric | Yes, if symptom free. | Antidiarrheal |
| **Blenoxane** | **No** | **Anti-neoplastic medication** |
| Blocadren | Yes, for hypertension; otherwise, no. | Antihypertensive, beta-blocker |
| Bonine | Yes, if taken for allergies. Defer for 72 hours after symptoms are resolved if taken for cold/flu symptoms. | Antihistamine |
| Bontril | Yes. | Anorexiant |
| Botulinum Toxin | Defer 1 wk. | Biological |
| Breonesin | Yes, if taken for allergies. Defer for 72 hours after symptoms are resolved if taken for cold/flu symptoms. | Expectorant |
| Brethine | Yes, even if daily dose for maintenance. | Bronchodilator |
| Brevicon | Yes. | Oral Contraceptive |
| Brexin | Yes, if taken for allergies. Defer for 72 hours after symptoms are resolved if taken for cold/flu symptoms. | Antihistamine/decongestant |
| Bricanyl | Yes, even if daily dose for maintenance. | Bronchodilator |
| Bristacycline | Defer 24 hrs. after course completed and feel well; if IV or IM defer 1 wk. Yes, if for acne. | Antibiotic |
| Bristamycin | Defer 24 hrs. after course completed and feel well; if IV or IM defer 1 wk. Yes, if for acne. | Antibiotic |
| Bromfed | Yes, if taken for allergies. Defer for 72 hours after symptoms are resolved if taken for cold/flu symptoms. | Decongestant, antihistamine |
| Bromocriptine | Yes. | Anti-Parkinsonism |
| Bromo-diphenhydramine | Yes, if taken for allergies. Defer for 72 hours after symptoms are resolved if taken for cold/flu symptoms. | Antihistamine |
| Bromophen T.D. | Yes, if taken for allergies. Defer for 72 hours after symptoms are resolved if taken for cold/flu symptoms. | Antihistamine |
| Brompheniramine | Yes, if taken for allergies. Defer for 72 hours after symptoms are resolved if taken for cold/flu symptoms. | Antihistamine |
| **Medication** | **Allowed to Take?** | **Type of Medication** |
| Brondecon | Yes, if taken for allergies. Defer for 72 hours after symptoms are resolved if taken for cold/flu symptoms. | Antihistamine |
| Bronkodyl, Bronkodyl S-R, Brokolixie, Bronkotabs | Yes, even if daily dose for maintenance. | Bronchodilator |
| Bucladin-S | Yes, if taken for allergies. Defer for 72 hours after symptoms are resolved if taken for cold/flu symptoms. | Antihistamine |
| Buclizine | Yes, if taken for allergies. Defer for 72 hours after symptoms are resolved if taken for cold/flu symptoms. | Antihistamine |
| Budesonide | Yes, even if daily dose for maintenance. | Anti-inflammatory, steroid (anti-rhinitis) |
| Buffaprin (Buffington) | Yes, if taken for allergies. Defer for 72 hours after symptoms are resolved if taken for cold/flu symptoms or for fever.  Defer 72 hrs for plateletpheresis or sole source platelets | ASA containing analgesic |
| Buffasal (Dover) | Yes, if taken for allergies. Defer for 72 hours after symptoms are resolved if taken for cold/flu symptoms or for fever.  Defer 72 hrs for plateletpheresis or sole source platelets | ASA containing analgesic |
| Bufferin, (All Forms) | Yes, if arthritis inactive.  Yes, if taken for allergies. Defer for 72 hours after symptoms are resolved if taken for cold/flu symptoms or for fever.  Defer 72 hrs for plateletpheresis or sole source platelets | Anti-inflammatory, analgesic, anti-pyretic, ASA. |
| Buffets II (JMI) | Yes, if taken for allergies. Defer for 72 hours after symptoms are resolved if taken for cold/flu symptoms or for fever.  Defer 72 hrs for plateletpheresis or sole source platelets | ASA containing analgesic |
| Buffex (Roberts) | Yes, if taken for allergies. Defer for 72 hours after symptoms are resolved if taken for cold/flu symptoms or for fever.  Defer 72 hrs for plateletpheresis or sole source platelets | ASA containing analgesic |
| Buffinol (Otis Clapp) | Yes, if taken for allergies. Defer for 72 hours after symptoms are resolved if taken for cold/flu symptoms or for fever.  Defer 72 hrs for plateletpheresis or sole source platelets | ASA containing analgesic |
| Bumetanide | Yes, if for hypertension and underlying condition not cause for deferral | Antihypertensive, diuretic |
| Bumex | Yes, if for hypertension and underlying condition not cause for deferral | Antihypertensive, diuretic |
| Bupropion | Yes. | Anti-depressant |
| Buspar | Yes. | Anti-anxiety agent |
| Buspirone | Yes. | Anti-anxiety agent |
| **Busulfan** | **No, permanent deferral.** | **Antineoplastic** |
| Butabarbital | Yes. | Hypnotic/sedative |
| Butal Compound (Novartis) | Yes, if taken for allergies. Defer for 72 hours after symptoms are resolved if taken for cold/flu symptoms or for fever.  Defer 72 hrs for plateletpheresis or sole source platelets | ASA containing analgesic |
| Butalbital | Yes, if taken for allergies. Defer for 72 hours after symptoms are resolved if taken for cold/flu symptoms or for fever.  Defer 72 hrs for plateletpheresis or sole source platelets | ASA containing analgesic |
| Butaperazine | Yes, if legally and mentally responsible. | Antipsychotic |
| Butazolidin | Yes, if arthritis inactive. | Anti-inflammatory. anti-pyretic |
| Butibel | Yes. | Sedative/antispasmodic |
| Butinal (Econolab) | Yes, if taken for allergies. Defer for 72 hours after symptoms are resolved if taken for cold/flu symptoms or for fever.  Defer 72 hrs for plateletpheresis or sole source platelets | ASA containing analgesic |
| Butiserpine, Butiserpazide | Yes. | Antihypertensive with sedative effect |
| Butisol | Yes. | Mild sedative |
| Butizide | Yes. | Antihypertensive |
| Butorphanol | Yes, if not abuser. | Narcotic, analgesic |
| Cabergoline | Yes. | Dopamine receptor agonist |
| Cafergot | Yes, if for migraine headaches. | Vasoconstrictor for migraine headache |
| Caffeine | Yes. | Stimulant |
| Calan | Yes, if for migraine, hypertension. No, if for angina, arrhythmias. | Vasodilator, CA Channel blocker |
| Calcidrine | Yes, if taken for allergies. Defer for 72 hours after symptoms are resolved if taken for cold/flu symptoms. | Antitussive, expectorant |
| *Calciferol* | *No, permanent deferral if renal patient. Yes, otherwise.* | *Vitamin D* |
| Calciparine | Defer 1 month until no longer require medication, evaluate underlying condition. | Heparin anti-coagulant |
| Calcitriol | No, permanent deferral if renal patient. Yes, otherwise. | Vitamin D |
| Calcium Carbonate | Defer permanently if renal patient. Yes, if ulcer disease pain-free. | Mineral, antacid |
| Calcium channel blockers | Yes, if for hypertension. No, if for angina or arrhythmia. | Antianginal, antiarrhythmic, antihypertensive |
| Calcium,  Calcium Gluceptate, Calcium Gluconate, Calcium Lactate | Yes. | Mineral supplement |
| Calderol | No, permanent deferral if renal patient. Yes, otherwise. | Vitamin D |
| Calmol 4 Suppository | Yes. | Topical hemorrhoidal |
| Calurin | Yes, if taken for allergies. Defer for 72 hours after symptoms are resolved if taken for cold/flu symptoms. | Analgesic, anti-pyretic,anti-inflammatory |
| **Calusterone** | **No, permanent deferral.** | **Antineoplastic** |
| Cama Arthritis Pain Reliever (Sandoz) | Yes, if taken for allergies. Defer for 72 hours after symptoms are resolved if taken for cold/flu symptoms or for fever.  Defer 72 hrs for plateletpheresis or sole source platelets | ASA containing analgesic |
| Cama Inlay Tab | Yes, if arthritis inactive.  Yes, if taken for allergies. Defer for 72 hours after symptoms are resolved if taken for cold/flu symptoms or for fever.  Defer 72 hrs for plateletpheresis or sole source platelets | Anti-inflammatory, ASA |
| Camalox | Yes, if ulcer disease pain free. | Antacid |
| Camoquin | Defer for 1 yr. if in malarial zone. Otherwise, yes. | Antimalarial |
| Candeptin | Yes, if for infections of nail beds or skin. | Antifungal-topical |
| Candex | Yes, if for infections of nail beds or skin. | Antifungal-topical |
| Candicidin | Yes, if for infections of nail beds or skin. | Antifungal-topical |
| Cantil | Yes, if ulcer disease pain-free. | Antispasmodic, anticholinergic |
| *Capastat* | *Defer until treatment completed and disease inactive.* | *Antituberculosis* |
| Capathyn, Cap. (Scrip) | Yes, if taken for allergies. Defer for 72 hours after symptoms are resolved if taken for cold/flu symptoms or for fever.  Defer 72 hrs for plateletpheresis or sole source platelets | ASA containing analgesic |
| Capoten | Yes for hypertension. No, defer if for heart disease. | Antihypertensive, ACE inhibitor |
| Capozide | Yes, for hypertension. | Combination antihypertensive |
| *Capreomycin* | *Defer until treatment completed and disease inactive.* | *Antituberculosis* |
| Captopril | Yes for hypertension. No, defer if for heart disease. | Antihypertensive, ACE inhibitor |
| Captron (Eon Labs) | Yes, if taken for allergies. Defer for 72 hours after symptoms are resolved if taken for cold/flu symptoms or for fever.  Defer 72 hrs for plateletpheresis or sole source platelets | ASA containing analgesic |
| Carafate | Yes, if ulcer disease pain-free. | Antiulcer |
| Caramiphen Edisylate | Yes, if taken for allergies. Defer for 72 hours after symptoms are resolved if taken for cold/flu symptoms. | Antitussive |
| Carbamazepine | Yes. | Anticonvulsant, analgesic |
| Carbarsone | Defer 1 wk. After course completed and feel well. | Amebicide |
| Carbenicillin | Defer 24 hrs. after course completed and feel well, if IV or IM defer 1 wk. | Antibiotic |
| Carbenoxolone | Yes, if ulcer disease pain-free. | Anti-ulcer |
| Carbidopa-Levodopa | Yes. | Anti-Parkinson agent |
| Carbinoxamine | Yes, if taken for allergies. Defer for 72 hours after symptoms are resolved if taken for cold/flu symptoms. | Antihistamine |
| Carboxymethyl-cellulose | Yes. | Laxative |
| Cardene | Yes, if for hypertension. No if for angina. | Anti-anginal, anti-hypertensive |
| *Cardilate* | *No, permanent deferral if for angina.* | *Vasodilator* |
| Cardioprin (Smart) | Yes, if taken for allergies. Defer for 72 hours after symptoms are resolved if taken for cold/flu symptoms or for fever.  Defer 72 hrs for plateletpheresis or sole source platelets | ASA containing analgesic |
| *Cardioquin* | *Defer if for heart disease or arrhythmia until off medication and stable.* | *Antiarrhythmic* |
| Cardizem | Defer if for angina. Yes, if for hypertension. | Anti-anginal, anti-hypertensive |
| Cardrase | Yes. | Diuretic, antiglaucoma |
| Cardura | Yes, if for hypertension. | Alpha blocker, potent antihypertensive. |
| Carisoprodol | Yes. Defer 72 hrs for plateletpheresis or sole source platelets | Muscle relaxant, sedative |
| Carnitor | Yes. | Carnitine replacement |
| Caroid and Bile Salts with Phenolphthalein | Yes. | Laxative |
| Carphenazine | Yes, if mentally and legally responsible. | Antipsychotic |
| Cartrol | Yes. | Antihypertensive, Beta blocker |
| Cascara Sagrada | Yes. | Laxative |
| **Casodex** | **No.** | **Used for the treatment of metastatic carcinoma of the prostate.** |
| Castor Oil | Yes. | Laxative |
| Cataflam | Yes, if arthritis inactive. Defer 24 hours if for plateletpheresis. | Non-steroidal anti-inflammatory. |
| Catapres | Yes. | Antihypertensive |
| Caverject (alprostadil; ProstinVR) | Yes | Treatment for erectile dysfunction |
| Ceclor | Defer 24 hrs. after course completed and feel well. | Antibiotic |
| Cecon | Yes. | Vitamin |
| **Cedilanid** | **No, permanent deferral.** | **Digitalis preparation** |
| **CeeNu** | **No, permanent deferral.** | **Antineoplastic** |
| Cefaclor | Defer 24 hrs. after course completed and feel well. | Antibiotic |
| Cefadroxil | Defer 24 hrs. after course completed and feel well. | Antibiotic |
| Cefaperazone | Defer 1 wk. | Antibiotic |
| Cefixime | Defer 24 hrs. after course completed and feel well. | Antibiotic |
| Cefizox, Ceftizoxime | Defer 1 week. | Antibiotic |
| Cefobid | Defer 1 week | Antibiotic |
| Cefotan, Cefotetan | Defer 1 week | Antibiotic |
| Ceftazimide | Defer 1 week. | Antibiotic |
| Ceftriaxone | Defer 1 wk. After course completed and feel well. | Antibiotic |
| Cefuroxime | Defer 1 wk. After course completed and feel well. | Antibiotic |
| Celebrex | Yes | COX-2 inhibitor; no deferral for platelet donation |
| Celestone | Defer 72 hrs. for oral and IM use. Otherwise, yes. | Steroid |
| Celexa | Yes | Antidepressant |
| Cenolate | Yes. | Vitamin |
| Centrax | Yes. | Antianxiety |
| Centrum | Yes. | Vitamins and minerals |
| Cephalexin | Defer 24 hrs. after course completed and feel well. | Antibiotic |
| Cephaloglycin | Defer 24 hrs. after course completed and feel well, if IV or IM defer 1 wk. | Antibiotic |
| Cephradine | Defer 24 hrs. after course completed and feel well. | Antibiotic |
| **Cephulac** | **No, permanent deferral.** | **Laxative used in hepatic encephalopathy.** |
| Ceptaz | Defer 1 week after course completed and feel well. | Antibiotic |
| Cerespan | Yes, evaluate underlying condition. | Muscle relaxant, used for vascular and visceral spasm |
| Cerivastatin (Baycol) | Yes | Anti-lipemic |
| Cevalin | Yes. | Vitamin |
| Cevi-Bid | Yes. | Vitamin |
| Ce-vi-Sol | Yes. | Vitamin |
| Chardonna-2 | Yes, if ulcer disease pain-free. | Anticholinergic/antispasmodic-Hypnotic, sedative |
| Chel-Iron | Yes. | Hematinic |
| Chendiol | Yes. | Bile acid |
| Chenodeoxycholic Acid | Yes. | Bile acid |
| Cheracol | Yes, if taken for allergies. Defer for 72 hours after symptoms are resolved if taken for cold/flu symptoms. | Antitussive, decongestant, antihistamine |
| Childrens Bayer Chewable (Glenbrook) | Yes, if taken for allergies. Defer for 72 hours after symptoms are resolved if taken for cold/flu symptoms or for fever.  Defer 72 hrs for plateletpheresis or sole source platelets | ASA containing analgesic |
| Chloral Betaine | Yes. | Hypnotic/sedative |
| Chloral Hydrate | Yes. | Hypnotic/sedative |
| **Chlorambucil** | **No, permanent deferral.** | **Antineoplastic** |
| Chloramphenicol | Defer 24 hrs. after course completed; defer 1 wk. if IM or IV use. | Antibiotic |
| Chlordiazachel | Yes. | Tranquilizer |
| Chlordiazepoxide | Yes. | Tranquilizer |
| Chloromycetin | Defer 24 hrs. after course completed; defer 1 wk. if IM or IV use. | Antibiotic |
| *Chloroquine* | *Defer 1 yr. If in malaria zone. Otherwise, yes.* | *Antimalarial* |
| *Chloroquine and Primaquine* | *Defer 1 yr. If in malaria zone. Otherwise, yes.* | *Antimalarial* |
| Chlorothiazide | Yes. | Diuretic, antihypertensive |
| ***Chlorotrianisene*** | ***Permanent deferral if cancer. Otherwise, yes.*** | ***Antineoplastic, estrogen*** |
| Chloroxazone | Yes. | Muscle relaxant, hypnotic/sedative |
| Chlorphenesin Carbamate | Yes. | Muscle relaxant, hypnotic/sedative |
| Chlorpheniramine | Yes, if taken for allergies. Defer for 72 hours after symptoms are resolved if taken for cold/flu symptoms. | Antihistamine |
| Chlorphenoxamine | Yes. | Anti-Parkinsonism |
| Chlorphentermine | Yes. | Anorexiant |
| Chlorpromazine | Yes, if legally and mentally responsible. | Antipsychotic |
| Chlorpropamide | Yes. | Oral hypoglycemic |
| Chlorprothixene | Yes, if legally and mentally responsible. | Antipsychotic |
| Chlorthalidone | Yes. | Antihypertensive, diuretic |
| Chlor-Trimeton | Yes, if taken for allergies. Defer for 72 hours after symptoms are resolved if taken for cold/flu symptoms. | Antihistamine |
| Chocks | Yes. | Vitamins |
| Cholan HMB, Cholan-DH | Yes, if ulcer disease pain-free | Anticholinergic, antispasmodic, hypnotic/sedative, bile salt |
| Choledyl | Yes, even if daily dose for maintenance. | Bronchodilator |
| Cholestyramine | Yes. | Cholesterol lowering agent. |
| Cholinate | Yes, if nutritional supplement. No, permanent deferral if tardive dyskinesia or chorea dementia. | Lipotropic agent |
| Choline | Yes, if nutritional supplement. No, permanent deferral if tardive dyskinesia or chorea dementia. | Lipotropic agent |
| Choline Magnesium | Yes, if arthritis inactive. | Anti-inflammatory |
| *Choline Salicylate* | *No, defer if collagen-vascular disease.* |  |
| Choloxin | Yes. | Cholesterol lowering agent. |
| Chromagen Capsules | Yes. | Vitamin, mineral, hematinic |
| **Chronulac** | **No, permanent deferral.** | **Laxative used in hepatic encephalopathy** |
| Cilazapril | Yes, if for hypertension. | Anti-hypertensive |
| Cimetidine | Yes, if ulcer disease pain-free. | Histamine-2 antagonist |
| Cinobac, Cinoxacin | Defer 24 hrs. after course completed and feel well. | Antibiotic |
| *Cin-Quin* | *No, permanent deferral if for heart disease or arrhythmias.* | *Antiarrhythmic* |
| Cipro, Ciprofloxacin hydrochloride | Defer 24 hrs. after course completed and feel well. | Anti-infective |
| Citrolith | Yes. | Phosphate urinary acidifier |
| Claritin | Yes, if taken for allergies. Defer for 72 hours after symptoms are resolved if taken for cold/flu symptoms. | Antihistamine |
| Clemastine | Yes, if taken for allergies. Defer for 72 hours after symptoms are resolved if taken for cold/flu symptoms. | Antihistamine |
| Cleocin | Defer 24 hrs. after course completed or feel well, defer 1 wk. if IM or IV. | Antibiotic |
| Clindamycin | Defer 24 hrs. after course completed or feel well, defer 1 wk. if IM or IV. | Antibiotic |
| Clindamycin Solution | Yes. | Topical antibiotic |
| Clindex | Yes. | Antispasmodic |
| Clinoril | Yes, if arthritis inactive. Defer plateletpheresis donors 24 hours. | Non-steroidal anti-inflammatory |
| Clistin | Yes, if taken for allergies. Defer for 72 hours after symptoms are resolved if taken for cold/flu symptoms. | Antihistamine |
| Clocortolone | Yes. | Topical corticosteroid |
| Cloderm | Yes. | Topical corticosteroid |
| **Clofaximine** | **No, permanent deferral.** | **Anti-leprosy** |
| Clofibrate | Yes. | Cholesterol lowering agent. |
| Clomid | Yes. | Fertility drug |
| Clomiphene | Yes. | Fertility drug |
| Clomipramine HCl | Yes. | Anti-obsessional |
| Clonazepam | Yes. | Anticonvulsant |
| Clonidine | Yes. | Antihypertensive |
| Clonopin | Yes. | Anticonvulsant |
| Clopidrogel (Plavix) | Acceptable for whole blood, red blood cells, FFP, and/or cryoprecipitate; for platelets defer for 5 days after last dose | Anti-platelet |
| Clorazepate | Yes. | Tranquilizer |
| Clorevan | Yes. | Anti-Parkinsonism |
| Clotermine | Yes. | Anorexiant |
| Clotrimazole | Yes, if for infections of skin or nail beds. | Topical antifungal |
| Cloxacillin | Defer 24 hrs. after course completed and feel well. | Antibiotic |
| Cloxapen | Defer 24 hrs. after course completed and feel well. | Antibiotic |
| Clozapine | Yes, if legally and mentally responsible. | Antipsychotic |
| Coactin | Defer 1 wk. After course completed and feel well. | Antibiotic |
| *Cocaine* | *No, defer 12 months for intranasal use; permanent deferral if injected. Use in a medical procedure as a local anesthetic is acceptable.* | *Anesthetic* |
| Codeine | Yes, unless abuser.  Yes, if taken for allergies. Defer for 72 hours after symptoms are resolved if taken for cold/flu symptoms. | Narcotic, analgesic, antitussive |
| Codeine polistirex | Yes, if taken for allergies. Defer for 72 hours after symptoms are resolved if taken for cold/flu symptoms. | Narcotic, antitussive |
| Codone | Yes, if taken for allergies. Defer for 72 hours after symptoms are resolved if taken for cold/flu symptoms. | Antitussive |
| Cogentin | Yes. | Anti-Parkinson |
| Cognex | Yes, if legally and mentally responsible. May cause elevated ALT. | Anti-Dementia, Alzheimer's disease |
| Colace | Yes. | Laxative |
| Colbenemid | Yes, if gout attack not acute. | Uricosuric agent |
| Colchicine | Yes, if gout attack not acute. | Anti-inflammatory-gout |
| Colestid | Yes. | Cholesterol lowering agent. |
| Colestipol | Yes. | Cholesterol lowering agent |
| Cologel | Yes. | Laxative |
| Colonin | Yes, if symptom free. | Antidiarrheal |
| Colrex Compound | Yes, if taken for allergies. Defer for 72 hours after symptoms are resolved if taken for cold/flu symptoms. | Antitussive, decongestant, analgesic |
| Combid | Yes. | Antiemetic, antispasmodic |
| Combipress | Yes. | Antihypertensive |
| Comhist LA | Yes, if taken for allergies. Defer for 72 hours after symptoms are resolved if taken for cold/flu symptoms. | Decongestant, antihistamine |
| Compazine | Yes, if legally and mentally responsible, otherwise OK. | Antiemetic, antipsychotic |
| Compocillin VK | Defer 24 hrs. after course completed and feel well, defer 1 wk. if IV or IM. | Antibiotic |
| Conar-A | Yes, if taken for allergies. Defer for 72 hours after symptoms are resolved if taken for cold/flu symptoms. | Antitussive, decongestant |
| Contac | Yes, if taken for allergies. Defer for 72 hours after symptoms are resolved if taken for cold/flu symptoms. | Decongestant, anticholinergic |
| Cope (Mentholatum) | Yes, if taken for allergies. Defer for 72 hours after symptoms are resolved if taken for cold/flu symptoms or for fever.  Defer 72 hrs for plateletpheresis or sole source platelets | ASA containing analgesic |
| Co-Pyronil | Yes, if taken for allergies. Defer for 72 hours after symptoms are resolved if taken for cold/flu symptoms. | Antihistamine |
| Cordran | Yes. | Topical Corticosteroid |
| Corgard | No, if for angina (permanent deferral) or arrhythmia (defer while on drug); otherwise, OK. | Antianginal, antiarrhythmic |
| Coricidin | Yes, if taken for allergies. Defer for 72 hours after symptoms are resolved if taken for cold/flu symptoms.  Defer 72 hrs for plateletpheresis or sole source platelets | Decongestant |
| Corrective Mixture | Yes, if symptom free for 3 days | Antidiarrheal |
| Cortisol | Defer 72 hrs. if P.O., IM or IV use. Topical and intraarticular OK. | Steroid |
| Cortisone Acetate | Defer 72 hrs. if P.O., IM or IV use. Topical and intra- articular OK. | Steroid |
| Cortisporin | Yes. | Topical antibiotic |
| Corzide | Yes, if for hypertension, no if for angina or arrhythmia. | Combination Corgard and Naturetim |
| **Cotazym** | **No** | **Enzyme replacement** |
| CoTylenol | Yes, if taken for allergies. Defer for 72 hours after symptoms are resolved if taken for cold/flu symptoms. | Decongestant, antihistamine |
| *Coumadin* | *Defer pending medical evaluation with cessation of drug.* | *Anticoagulant* |
| Cozaar | Yes. | Antihypertensive |
| Creon | Yes | Pancreatic enzyme concentrate |
| Creoterp | Yes, if taken for allergies. Defer for 72 hours after symptoms are resolved if taken for cold/flu symptoms. | Expectorant |
| Cromolyn | Yes, even if daily dose for maintenance. | Anti-asthmatic |
| Crysticillin AS | Defer 24 hrs. after course completed and feel well. Defer 1 wk. if IV or IM. | Antibiotic |
| **Crystodigin** | **No, permanent deferral.** | **Digitalis agent** |
| Cuprimine | Yes, if arthritis inactive. No, if collagen vascular disease or other chronic disease. | Anti-inflammatory, Anti-rheumatic |
| *Curretab* | *No, if cancer patient; otherwise yes.* | *Hormone* |
| Cyanocobalamin | Yes. | Vitamin B_12_ |
| Cyantin | Defer 24 hrs. after course completed and feel well; if IV or IM defer 1 wk. | Antibiotic |
| Cyclacillin | Defer 24 hrs. after course completed and feel well; if IV or IM defer 1 wk. | Antibiotic |
| Cyclamycin | Defer 24 hrs. after course completed and feel well; if IV or IM defer 1 wk. | Antibiotic |
| Cyclandelate | Defer if cerebrovascular disease. Yes, if peripheral vascular disease. | Vasodilator |
| Cyclapen | Defer 24 hrs. after course completed and feel well; if IV or IM defer 1 wk. | Antibiotic |
| Cyclazocine | Yes, if not abuser. | Narcotic agonist/antagonist |
| Cyclizine | Yes, if taken for allergies. Defer for 72 hours after symptoms are resolved if taken for cold/flu symptoms. | Antihistamine, antiemetic |
| Cyclobenzaprine | Yes. | Muscle relaxant. |
| Cyclopar | Defer 24 hrs. after course completed and feel well; if IV or IM defer 1 wk. Yes, if used for acne. | Antibiotic |
| **Cyclophosphamide** | **No, permanent deferral.** | **Antineoplastic** |
| Cycloserine | Yes, if treatment complete and disease inactive. | Antituberculous |
| Cyclospasmol | Defer if cerebrovascular disease. Yes, if peripheral vascular disease. | Vasodilator |
| Cyclothiazide | Yes. | Antihypertensive, diuretic |
| Cycrimine | Yes. | Anti-Parkinsonism |
| Cycrin | Yes | Progesterone derivative |
| Cylert | Yes. | CNS stimulant |
| Cyproheptadine | Yes, if taken for allergies. Defer for 72 hours after symptoms are resolved if taken for cold/flu symptoms. | Antihistamine |
| Cystospaz | Yes, if ulcer disease pain-free | Anticholinergic, antispasmodic |
| **Cytadren** | **No, permanent deferral.** | **Anti-adrenal agent** |
| Cytellin | Yes. | Anti-hyperlipidemic |
| Cytomel | Yes. | Synthetic thyroid hormone |
| Cytotec | Yes, if 4 hours since last dose. | Anti-ulcer medication |
| **Cytoxan** | **No, permanent deferral.** | **Antineoplastic** |
| D.H.E.45 | Yes. | Vasoconstrictor (migraine headaches) |
| Dalmane | Yes. | Tranquilizer |
| Damason-P (Mason) | Yes, if taken for allergies. Defer for 72 hours after symptoms are resolved if taken for cold/flu symptoms or for fever.  Defer 72 hrs for plateletpheresis or sole source platelets | ASA containing analgesic |
| Danazol | Yes. | Gonadotropin antagonist |
| Danocrine | Yes. | Gonadotropin antagonist |
| Danthron | Yes. | Laxative |
| Dantrium | Yes. | Spasmolytic |
| Dantrolene | Yes. | Spasmolytic |
| Daprisal | Yes, if taken for allergies. Defer for 72 hours after symptoms are resolved if taken for cold/flu symptoms or for fever.  Defer 72 hrs for plateletpheresis or sole source platelets | Analgesics (ASA+amphetamine) |
| *Dapsone* | *No, permanent deferral if for Leprosy. Yes, if for other entities.* | *Anti-leprosy agent* |
| Daranide | Yes. | Anti-glaucoma |
| *Daraprim* | *Defer 1 yr. If in malarial zone. Otherwise, yes.* | *Antimalarial* |
| Darbid | Yes, if ulcer disease pain-free. | Anticholinergic, antispasmodic |
| Daricon, Daricon-PB | Yes, if ulcer disease pain-free. | Anticholinergic, antispasmodic |
| Darvocet-N | Yes, if taken for allergies. Defer for 72 hours after symptoms are resolved if taken for cold/flu symptoms. | Analgesic, Antipyretic |
| Darvon | Yes, if not an abuser  Yes, if taken for allergies. Defer for 72 hours after symptoms are resolved if taken for cold/flu symptoms. | Analgesic |
| Darvon Compound, Darvon-N (Lilly) | Yes, if taken for allergies. Defer for 72 hours after symptoms are resolved if taken for cold/flu symptoms or for fever.  Defer 72 hrs for plateletpheresis or sole source platelets | ASA containing analgesic |
| Dasikon (Smith-Kline) | Yes, if taken for allergies. Defer for 72 hours after symptoms are resolved if taken for cold/flu symptoms or for fever.  Defer 72 hrs for plateletpheresis or sole source platelets | ASA containing analgesic |
| Dasin (Beecham) | Yes, if taken for allergies. Defer for 72 hours after symptoms are resolved if taken for cold/flu symptoms or for fever.  Defer 72 hrs for plateletpheresis or sole source platelets | ASA containing analgesic |
| Dasprin | Yes, if taken for allergies. Defer for 72 hours after symptoms are resolved if taken for cold/flu symptoms or for fever.  Defer 72 hrs for plateletpheresis or sole source platelets | ASA containing analgesic |
| Datril | Yes, if taken for allergies. Defer for 72 hours after symptoms are resolved if taken for cold/flu symptoms. | Analgesic, Antipyretic |
| Daxolin | Yes, if legally and mentally responsible. | Antipsychotic |
| Dayalets | Yes. | Vitamins/minerals |
| Daypro | Yes, if arthritis inactive. Defer plateletpheresis donors 24 hours. | Non-steroidal anti-inflammatory |
| *DDAVP* | *No, if for von Willebrand’s Disease. Yes, if for other uses.* | *Antidiuretic (also increases Factor VIII in Hemophilia A and von Willebrand's Disease)* |
| Deaner | Yes, if movement does not interfere with phlebotomy and if legally and mentally responsible | Acetylcholine precursor (hyperkinesis, tardive dyskinesia) |
| Deanol | Yes, if movement does not interfere with phlebotomy and if legally and mentally responsible | Acetylcholine precursor |
| Deapril-ST | Defer 24 hrs. | Ergot alkaloid (elderly-selected mood elevator) |
| Decadron | Defer 72 hrs. if P.O. or IM. Yes, if topical or intra-articular. | Steroid |
| *Deca-Durabolin* | *No, if renal disease patient. Yes, otherwise.* | *Anabolic steroid* |
| Decapryn | Yes. | Bile salt |
| Decholin | Yes. | Bile salt |
| Declomycin | No, defer 24 hrs. If for acne, yes. | Antibiotic |
| Declostatin | Defer 24 hrs. after course completed and feel well. | Antibiotic, antifungal |
| Decolyn (Richlyn) | Yes, if taken for allergies. Defer for 72 hours after symptoms are resolved if taken for cold/flu symptoms or for fever.  Defer 72 hrs for plateletpheresis or sole source platelets | ASA containing analgesic |
| Deconamine | Yes, if taken for allergies. Defer for 72 hours after symptoms are resolved if taken for cold/flu symptoms. | Antihistamine |
| Decotussin | Yes, if taken for allergies. Defer for 72 hours after symptoms are resolved if taken for cold/flu symptoms or for fever.  Defer 72 hrs for plateletpheresis or sole source platelets | ASA containing analgesic |
| Dehist | Yes, if taken for allergies. Defer for 72 hours after symptoms are resolved if taken for cold/flu symptoms. | Antihistamine |
| Dehydrocholic Acid | Yes. | Bile salt |
| Dehydroemetine | Defer 1 wk. After course completed and feel well. | Amebiasis |
| Deladumone | Yes. | Hormone |
| Delatestryl | Yes. | Androgen hormone |
| Delautin | Yes. | Hormone |
| Delaxin | Yes. | Hypnotic/sedative |
| Delcid | Yes, if ulcer disease pain-free. | Antacid |
| Delta-Cortef | Defer 72 hrs. if oral or IM. Yes, if topical or intra-articular. | Steroid |
| Deltalin | Yes, unless renal patient. | Vitamin |
| Deltasone | Defer 72 hrs. if oral or IM Yes, if topical or intra-articular. | Steroid |
| Demadex (torsemide) | Yes. | Diuretic |
| Demazin | Yes, if taken for allergies. Defer for 72 hours after symptoms are resolved if taken for cold/flu symptoms. | Decongestant, antihistamine |
| Demeclocycline | Defer 24 hrs. after course completed and feel well. Yes, if for acne. | Antibiotic |
| Demerol | Yes, if not abuser. | Narcotic analgesic |
| Democlomycin | Defer 24 hrs. after course completed and feel well. Yes, if for acne. | Antibiotic |
| **Demser** | **No, permanent deferral.** | **Rx of pheochromocytoma** |
| Demulen | Accept. | Oral contraceptive |
| Dentavite | Yes. | Vitamin/mineral |
| Depakene | Yes. | Anticonvulsant |
| Depakote | Yes. | Anticonvulsant |
| Depancol | Yes. | Pancreatic enzyme |
| Depen | Yes, if arthritis inactive. No, if collagen vascular disease or other chronic disease. | Anti-inflammatory |
| Depo-Medrol | Defer 72 hrs. if oral or IM, otherwise, yes. | Steroid |
| *Depo-Provera* | *No, permanent deferral if cancer, otherwise, yes.* | *Hormone* |
| Deprol | Yes, maintenance only. | Antidepressant, tranquilizer |
| DES (Diethylstilbestrol) | See Diethylstilbestrol |  |
| Deserpidine | Yes. | Antihypertensive |
| Desipramine | Yes. | Anti-depressant |
| **Desmopressin** | **No, permanent deferral.** | **Antidiuretic hormone** |
| **Desoxycorti-costerone** | **No, permanent deferral.** | **Hormone mineralocorticoid** |
| Desoxyn | Yes. | Anorexiant |
| Desyrel | Yes. | Antidepressant. |
| Dexamethasone | Defer 72 hrs. if oral or IM, otherwise yes. | Steroid |
| Dexamyl | Yes. | Amphetamine, barbiturate |
| Dexbrompheniramine | Yes, if taken for allergies. Defer for 72 hours after symptoms are resolved if taken for cold/flu symptoms. | Antihistamine |
| Dexchlorpheniramine | Yes, if taken for allergies. Defer for 72 hours after symptoms are resolved if taken for cold/flu symptoms. | Antihistamine |
| Dexedrine | Yes. | Amphetamine |
| Dextroamphetamine | Yes. | Anorexiant |
| Dextromethorphan | Yes, if taken for allergies. Defer for 72 hours after symptoms are resolved if taken for cold/flu symptoms. | Antitussive |
| Dextrothyroxine | Yes. | Cholesterol lowering agent |
| DiaBeta | Yes. | Oral hypoglycemic |
| Diabinese | Yes. | Oral hypoglycemic |
| Diafen | Yes, if taken for allergies. Defer for 72 hours after symptoms are resolved if taken for cold/flu symptoms. | Antihistamine |
| Dialose | Yes. | Laxative |
| Dialume | Yes, if ulcer disease pain-free. | Antacid |
| Diamox | Yes. | Enzyme inhibitor controlling fluid excretion (Glaucoma) |
| Dianabol | Yes. | Anabolic steroid |
| **Diapid** | **No, permanent deferral.** | **Antidiuretic hormone** |
| **Diasone** | **No, permanent deferral.** | **Antileprosy agent** |
| Diazepam | Yes. | Tranquilizer |
| Diazide | Yes. | Diuretic |
| Diazoxide | Yes, if blood glucose stable. | Hyperglycemic |
| *Dibenzyline* | *Accept if for urinary problems. No, permanent deferral if for pheochromocytoma.* | *Vasodilator, antihypertensive(may cause very low pressure or tachycardia with standing)* |
| *Dicarbosil* | *No, permanent deferral if renal patient. Yes, if ulcer disease pain-free.* | *Mineral, antacid* |
| Dichlorphenamide | Yes. | Antiglaucoma |
| Diclofenac | Yes, if arthritis inactive. Defer 24 hrs. if plateletpheresis donor. | Non-steroidal anti-inflammatory |
| Dicloxacillin | Defer 24 hrs. after course completed and feel well. | Antibiotic |
| Dicodid | Yes, if taken for allergies. Defer for 72 hours after symptoms are resolved if taken for cold/flu symptoms. | Antitussive |
| *Dicoumarin, Dicoumarol, Dicumarol* | *Defer pending medical evaluation with cessation of drug.* | *Anticoagulant* |
| Dicyclomine | Yes, if ulcer disease pain-free. | Anticholinergic/Antispasmodic |
| Didrex | Yes. | Anorexiant |
| Didronel | Yes, if for osteoporosis. | Inhibits bone metabolism (Paget's Disease) |
| Diethylcarbamazine | Defer 1 wk. and feel well. | Antihelminthic |
| Diethylpropion | Yes. | Anorexiant |
| **Diethylstilbestrol (DES)** | **No, permanent deferral.** | **Estrogen** |
| Differin Gel | Yes. | Topical treatment for acne. |
| Diflucan | Yes, if for infection of skin or nail beds. No, if for generalized (systemic) infection | Antifungal; may cause elevated ALT. |
| Diflunisal | Yes, if arthritis inactive  Yes, if taken for allergies. Defer for 72 hours after symptoms are resolved if taken for cold/flu symptoms.  Defer plateletpheresis donors 24 hours. | Analgesic, non-steroidal anti-inflammatory. |
| **Digitalis, Digitoxin, Digoxin** | **No, permanent deferral.** | **Cardiac Glycoside** |
| Dihycon | Yes. | Anticonvulsant |
| Dihydroergotamine | Yes. | Vasoconstrictor |
| *Dihydrotachysterol* | *No, permanent deferral if renal disease. Yes, otherwise.* | *Vitamin D* |
| Dihydroxyaluminum-Aminoacetate, Sodium Carbonate | Yes, if ulcer disease pain-free. | Antacid |
| Diiodohydroxyquin | Defer 1 wk. and feel well. | Amebicide |
| Dilacor x R | Yes, if for hypertension. | Beta Blocker |
| Dilantin | Yes. | Anticonvulsant |
| **Dilatrate-SR** | **No, permanent deferral.** | **Antianginal** |
| Dilaudid | Yes, if not an abuser | Narcotic analgesic |
| Dilor | Yes, even if daily dose for maintenance. | Bronchodilator |
| Dilotab (Zee Medical) | Yes, if taken for allergies. Defer for 72 hours after symptoms are resolved if taken for cold/flu symptoms or for fever.  Defer 72 hrs for plateletpheresis or sole source platelets | ASA containing analgesic |
| Diloxanide Furoate | Defer 1 wk. and feel well. | Amebicide |
| *Diltiazem* | *No, permanent deferral if for angina. Yes, if for hypertension.* | *Anti-anginal, anti-hypertensive* |
| Dimacol | Yes, if taken for allergies. Defer for 72 hours after symptoms are resolved if taken for cold/flu symptoms. | Antihistamine, decongestant, Antitussive |
| Dimenhydrinate | Yes, if taken for allergies. Defer for 72 hours after symptoms are resolved if taken for cold/flu symptoms. | Antinauseant, antihistamine |
| Dimetane | Yes, if taken for allergies. Defer for 72 hours after symptoms are resolved if taken for cold/flu symptoms. | Antihistamine |
| Dimetapp | Yes, if taken for allergies. Defer for 72 hours after symptoms are resolved if taken for cold/flu symptoms. | Antihistamine, decongestant |
| Dimethindene | Yes, if taken for allergies. Defer for 72 hours after symptoms are resolved if taken for cold/flu symptoms. | Antihistamine |
| Dimethyl Sulfoxide | Defer 1 week after course completed. | Anti-inflammatory, anti-spasmodic |
| Dioctyl Sodium Sulfossuccinate, Dioctyl Calcium Sulfosuccinate | Yes. | Laxative |
| Diovan (Valsartan) | Yes. | Angiotensin blocker (receptor) |
| **Dipentum** | **No.** | **Anti-inflammatory for ulcerative colitis** |
| Diphemanil | Yes, if ulcer disease pain-free. | Anticholinergic/Antispasmodic |
| Diphenhydramine | Yes, if taken for allergies. Defer for 72 hours after symptoms are resolved if taken for cold/flu symptoms. | Antihistamine |
| Diphenidol | Yes, if symptom free. | Antiemetic |
| Diphenoxylate | Yes, if symptom free.  Defer for 72 hours after symptoms are resolved if taken for cold/flu symptoms or for fever. | Antidiarrheal |
| Di-Phenyl | Yes. | Anticonvulsant |
| Diphenylpyraline | Yes, if taken for allergies. Defer for 72 hours after symptoms are resolved if taken for cold/flu symptoms. | Antihistamine |
| Diphylets | Yes. | Amphetamine |
| Diprolene | Defer 72 hrs after use completed if IV, IM or PO, otherwise, yes. | Corticosteroid Anti-inflammatory |
| *Dipyridamole* | *No, permanent deferral if for angina or heart disease. Yes if used as anti-platelet and underlying condition is not cause for deferral. No, if used as vasodilator.* | *Anti-platelet. Anti-anginal* |
| Disalcid | Yes, if arthritis inactive. | Anti-inflammatory |
| Disipal | Yes. | Muscle relaxant. Anti-Parkinsonism |
| Disophrol | Yes, if taken for allergies. Defer for 72 hours after symptoms are resolved if taken for cold/flu symptoms. | Antihistamine, decongestant |
| *Disopyramide* | *Defer until off drug and condition stable.* | *Anti-arrhythmic* |
| Disulfiram | Defer 1 week, evaluate underlying condition. | Enzyme inhibitor used for alcoholism |
| Ditropan | Yes. | Antispasmodic |
| Diucardin | Yes. | Antihypertensive, diuretic |
| Diulo | Yes. | Antihypertensive, diuretic |
| Diupres | Yes. | Antihypertensive-patient susceptible to orthostatic hypotension. |
| Diuril | Yes. | Antihypertensive, diuretic |
| Diutensen | Yes. | Antihypertensive, diuretic; patient susceptible to orthostatic hypotension. |
| DMSO | Yes. | Anti-inflammatory |
| Doans Pills | Yes, if taken for allergies. Defer for 72 hours after symptoms are resolved if taken for cold/flu symptoms. | Analgesic |
| **Doca Acetate** | **No, permanent deferral.** | **Hormone mineralocorticoid** |
| Docusate Calcium, Docusate Potassium, Docusate Sodium | Yes. | Laxative |
| Dolcin (Dolcin Corp) | Yes, if taken for allergies. Defer for 72 hours after symptoms are resolved if taken for cold/flu symptoms or for fever.  Defer 72 hrs for plateletpheresis or sole source platelets | ASA containing analgesic |
| Dolene | Yes, if taken for allergies. Defer for 72 hours after symptoms are resolved if taken for cold/flu symptoms. | Analgesic |
| Dolobid | Yes, if taken for allergies. Defer for 72 hours after symptoms are resolved if taken for cold/flu symptoms.  Defer plateletpheresis donors 72 hours. | Analgesic, anti-inflammatory |
| **Dolophine** | **No, permanent deferral if drug abuser.** | **Analgesic** |
| *Domestrol* | *No, permanent deferral if cancer; yes, otherwise.* | *Hormone* |
| **Donepezil HCl, (Aricept)** | **No, permanent deferral** | **Cholinesterase inhibitor** |
| Donnatal | Yes, if ulcer disease pain-free. | Antispasmodic, anti-cholinergic |
| Donnazyme | Yes, if ulcer disease pain-free. | Anticholinergic, antispasmodic |
| Donnegel | Yes, if symptom free. | Antidiarrheal |
| Donphen | Yes, if ulcer disease pain-free. | Anticholinergic, antispasmodic |
| Dopar | Yes. | Anti-Parkinsonism |
| Doral | Yes. | Tranquilizer |
| Dorbane | Yes. | Laxative |
| Dorbantyl | Yes. | Laxative |
| Dorcol Cough Syrup | Yes, if taken for allergies. Defer for 72 hours after symptoms are resolved if taken for cold/flu symptoms. | Antitussive |
| Doriden | Yes, if not abuser. | Hypnotic, sedative |
| Doryx | Defer 24 hrs. after course completed and feel well; IV or IM defer 1 wk. Yes, if for acne | Antibiotic |
| Dostinex | Yes | Dopamine receptor agonist |
| Doxazosin | Yes, if B/P is stable. | Alpha blocker, potent anti-hypertensive |
| Doxepin | Yes. | Tricyclic antidepressant |
| Doxicomine | Yes, if not abuser.  Yes, if taken for allergies. Defer for 72 hours after symptoms are resolved if taken for cold/flu symptoms. | Analgesic (opioid) |
| Doxidan | Yes. | Laxative |
| Doxpicodine | Yes, if not abuser.  Yes, if taken for allergies. Defer for 72 hours after symptoms are resolved if taken for cold/flu symptoms. | Analgesic (opioid) |
| Doxychel | Defer 24 hrs. after course completed and feel well; IV or IM defer 1 wk. Yes, if for acne | Antibiotic |
| Doxycycline | Defer 24 hrs. after course completed and feel well; IV or IM defer 1 wk. Yes, if for acne | Antibiotic |
| Doxylamine | Yes, if taken for allergies. Defer for 72 hours after symptoms are resolved if taken for cold/flu symptoms. | Antihistamine |
| Dralserp | Yes, watch for orthostatic pressure changes. | Antihypertensive |
| Dralzine | Yes, if for hypertension. No, if for congestive heart failure. | Vasodilator Antihypertensive |
| Dramamine | Yes, evaluate., not accept if currently symptomatic. | Antinauseant, antiemetic |
| Drisdol | Yes, unless renal patient. | Vitamin D |
| Dristan | Yes, if taken for allergies. Defer for 72 hours after symptoms are resolved if taken for cold/flu symptoms.  Defer 72 hrs for plateletpheresis or sole source platelets | Decongestant, antihistamine |
| Drixoral | Yes, if taken for allergies. Defer for 72 hours after symptoms are resolved if taken for cold/flu symptoms. | Antihistamine |
| **Dronabinol** | **No, permanent deferral.** | **Treats anorexia present in AIDS** |
| Dulcolax | Yes. | Laxative |
| Duolax | Yes. | Laxative |
| Duo-Medihaler | Yes, even if daily dose for maintenance. | Bronchodilator |
| **Duotrate** | **No, permanent deferral.** | **Anti-anginal** |
| Duphaston | Yes. | Hormone replacement |
| *Durabolin* | *No, permanent deferral if renal disease. Otherwise, yes.* | *Anabolic steroid* |
| Duracillin | Defer 1 month, unless used to treat STD's, then 12 months. | Long acting penicillin |
| Duract | Yes. | Nonsteroidal anti-inflammatory |
| Duradrin | Yes, if taken for allergies. Defer for 72 hours after symptoms are resolved if taken for cold/flu symptoms. | Analgesic |
| Duradyne (Forest) | Yes, if taken for allergies. Defer for 72 hours after symptoms are resolved if taken for cold/flu symptoms or for fever.  Defer 72 hrs for plateletpheresis or sole source platelets | ASA containing analgesic |
| Dura-Meth | Defer 72 hrs. if P.O. or IM. Yes, if topical or intra-articular. | Corticosteroid |
| *Duraquin* | *Defer until off drug and condition stable.* | *Anti-arrhythmic* |
| Duravent | Yes, if taken for allergies. Defer for 72 hours after symptoms are resolved if taken for cold/flu symptoms. | Decongestant, expectorant |
| Duricef | Defer 24 hrs. after course completed and feel well. | Antibiotic |
| *Dutasteride (AVODART)* | *No, defer until 6 months after last dose of medication.* | *Used for treatment of benign prostatic hyperplasia in men with enlarged prostate. Potential fetal anomaly to male fetus.* |
| Duvoid | Yes. | Cholinomimetic |
| Dyazide | Yes. | Antihypertensive, diuretic |
| Dycill | Defer 24 hrs. after course completed and feel well. Defer 1 wk. if IM or IV. | Antibiotic |
| Dydrosterone | Yes. | Hormone |
| Dyhydrogesterone | Yes. | Hormone |
| Dymelor | Yes. | Oral hypoglycemic |
| Dynacirc | Yes. | Calcium antagonist (israpidine) |
| Dynapen | Defer 24 hrs. after course completed and feel well; if IM or IV defer 1 wk. | Antibiotic |
| Dyphylline | Yes, even if daily dose for maintenance. | Bronchodilator |
| Dyrenium | Yes. | Diuretic |
| Dyspas | Yes. | Anti-spasmodic |
| E.E.S. | Defer 24 hrs. after course completed and feel well. Yes, if for acne. | Antibiotic |
| Easprin | Yes, if taken for allergies. Defer for 72 hours after symptoms are resolved if taken for cold/flu symptoms or for fever. Defer 72 hrs for plateletpheresis or sole source platelets | Analgesic-enteric coated ASA |
| Ecotrin | Yes, if taken for allergies. Defer for 72 hours after symptoms are resolved if taken for cold/flu symptoms or for fever. Defer 72 hrs for plateletpheresis or sole source platelets | Analgesic, ASA. |
| Edecrin | Yes. | Antihypertensive, diuretic |
| Edrisal | Yes, if taken for allergies. Defer for 72 hours after symptoms are resolved if taken for cold/flu symptoms.  Defer 72 hrs for plateletpheresis or sole source platelets | Analgesic |
| E-Ferol | Yes. | Vitamin |
| Effersyllium | Yes. | Laxative |
| Effexor | Yes. | Anti-Depressant |
| Efudex | Yes, if topical. No, permanent deferral if IV or IM. | Desquamator. Antineoplastic |
| **EHDP** | **No, permanent deferral.** | **Inhibitor of bone metabolism. (Paget's Disease)** |
| E-Ionate P.A. | Yes. | Hormone |
| EKKO | Yes. | Anticonvulsant |
| Elavil | Yes. | Tricyclic antidepressant |
| Eldepryl | Yes. | Anti-Parkinsonism |
| Elixicon | Yes, even if daily dose for maintenance. | Bronchodilator |
| Elixophyllin | Yes, even if daily dose for maintenance. | Bronchodilator |
| Emagrin (Otis Clapp) | Yes, if taken for allergies. Defer for 72 hours after symptoms are resolved if taken for cold/flu symptoms or for fever.  Defer 72 hrs for plateletpheresis or sole source platelets | ASA containing analgesic |
| **Emcyt** | **No** | **Anti-neoplastic medication** |
| Emete-con | Yes, if asymptomatic. | Antiemetic |
| Emetine | Defer 1 wk. after course completed and feel well. | Amebicide |
| Emetrol | Yes. | Antiemetic |
| Emgel | Defer 24 hrs. after course completed and feel well; if IV or IM defer 1 wk. Yes, if for acne. | Antibiotic |
| Empirin | Yes, if taken for allergies. Defer for 72 hours after symptoms are resolved if taken for cold/flu symptoms or for fever.  Defer 72 hrs for plateletpheresis or sole source platelets | Analgesic, anti-pyretic, anti-inflammatory, ASA. |
| Empirin with Codeine | Yes, if taken for allergies . Defer for 72 hours after symptoms are resolved if taken for cold/flu symptoms or for fever.  Defer 72 hrs for plateletpheresis or sole source platelets | ASA containing analgesic |
| Empracet | Yes, if taken for allergies. Defer for 72 hours after symptoms are resolved if taken for cold/flu symptoms. | Analgesic, antipyretic |
| E-mycin | Defer 24 hrs. after course completed and feel well. | Antibiotic |
| Enalapril Maleate | Yes, if for hypertension. No, if for heart disease. | Antihypertensive, ACE inhibitor |
| Enalapril Maleate-Hydrochlorothiazide | Yes, if for hypertension. No, if for heart disease. | Antihypertensive, ACE inhibitor |
| Enarex | Yes, if ulcer disease pain-free. | Anticholinergic/antispasmodic |
| En-Cebrin F | Yes, if taken for allergies. Defer for 72 hours after symptoms are resolved if taken for cold/flu symptoms. | Antihistamine, decongestant |
| Endal | Yes, if taken for allergies. Defer for 72 hours after symptoms are resolved if taken for cold/flu symptoms. | Antihistamine, decongestant |
| Endep | Yes. | Tricyclic antidepressant |
| Endodan (Endo) | Yes, if taken for allergies. Defer for 72 hours after symptoms are resolved if taken for cold/flu symptoms or for fever.  Defer 72 hrs for plateletpheresis or sole source platelets | ASA containing analgesic |
| Enduron | Yes, if for hypertension. | Diuretic, antihypertensive |
| Enduronyl | Yes, if for hypertension. | Diuretic, antihypertensive |
| Engran-HP | Yes. | Vitamin/minerals |
| Enovid | Yes. | Oral Contraceptive |
| Enterfilm (Time Cap Labs) | Yes, if taken for allergies. Defer for 72 hours after symptoms are resolved if taken for cold/flu symptoms or for fever.  Defer 72 hrs for plateletpheresis or sole source platelets | ASA containing analgesic |
| Entex, Entex-LA | Yes, if taken for allergies. Defer for 72 hours after symptoms are resolved if taken for cold/flu symptoms. | Decongestant |
| Entozyme | Yes. | Bile salt and enzyme replacement |
| Ephedrine | Yes, even if daily dose for maintenance. | Bronchodilator |
| Epinephrine | Yes, if not daily dose for maintenance but intermittent use. | Bronchodilator |
| Epragen (Lilly) | Yes, if taken for allergies. Defer for 72 hours after symptoms are resolved if taken for cold/flu symptoms or for fever.  Defer 72 hrs for plateletpheresis or sole source platelets | ASA containing analgesic |
| Eprolin | Yes. | Vitamin E |
| Epromate (Major) | Yes, if taken for allergies. Defer for 72 hours after symptoms are resolved if taken for cold/flu symptoms or for fever.  Defer 72 hrs for plateletpheresis or sole source platelets | ASA containing analgesic |
| Equagesic | Yes, if taken for allergies. Defer for 72 hours after symptoms are resolved if taken for cold/flu symptoms or for fever.  Defer 72 hrs for plateletpheresis or sole source platelets | Analgesic, ASA. |
| Equanil | Yes. | Tranquilizer |
| *Equanitrate* | *No, until off medication and symptom free.* | *Anti-anginal* |
| Equagesic Tablets (Wyeth-Ayerst) | Yes, if taken for allergies. Defer for 72 hours after symptoms are resolved if taken for cold/flu symptoms or for fever.  Defer 72 hrs for plateletpheresis or sole source platelets | ASA containing analgesic |
| Equazine (Quantum) | Yes, if taken for allergies. Defer for 72 hours after symptoms are resolved if taken for cold/flu symptoms or for fever.  Defer 72 hrs for plateletpheresis or sole source platelets | ASA containing analgesic |
| *Ergamisol* | *Defer 1 wk. if for worms; otherwise, permanent deferral.* | *Antihelminthic, immunomodulator, anti-rheumatoid* |
| Ergomar | Yes. | Vasoconstrictor (migraine headache) |
| Ergonovine | Yes. | Vasoconstrictor |
| Ergostat | Yes. | Vasoconstrictor |
| Ergot Alkaloids | Yes. | Vasoconstrictor |
| Ergotamine | Yes. | Vasoconstrictor |
| Ergotrate | Yes. | Vasoconstrictor |
| ERYC | Defer 24 hrs. after course completed and feel well; if IV or IM defer 1 wk. Yes, if for acne. | Antibiotic |
| EryDerm | Defer 24 hrs. after course completed and feel well, if IV or IM defer 1 wk. Yes, if for acne. | Antibiotic |
| Erypar | Defer 24 hrs. after course completed and feel well, if IV or IM defer 1 wk. Yes, if for acne | Antibiotic |
| Ery-Tab | Defer 24 hrs. after course completed and feel well; if IV or IM defer 1 wk. Yes, if for acne. | Antibiotic |
| *Erythrityl Tetranitrate* | *No, until off medication and symptom free.* | *Antianginal* |
| Erythrocin | Defer 24 hrs. after course completed and feel well. Yes, if for acne. | Antibiotic |
| Erythromycin | Defer 24 hrs. after course completed and feel well; if IV or IM defer 1 wk. Yes, if for acne. | Antibiotic |
| Escot | Yes, if ulcer disease pain-free. | Antacid |
| Esgic | Yes, if taken for allergies. Defer for 72 hours after symptoms are resolved if taken for cold/flu symptoms. | Analgesic, sedative |
| Esidrix | Yes. | Antihypertensive, diuretic |
| Esimil | Defer 72 hours. | Ganglionic blocking agent and diuretic, potent antihypertensive |
| Eskabarb | Yes. | Anticonvulsant, sedative, tranquilizer |
| Eskalith | Yes, if for maintenance. | Lithium |
| Eskatrol | Yes. | Amphetamine |
| Estinyl | Yes. | Hormone |
| Estrace | Yes. | Hormone |
| Estradiol | Yes. | Hormone |
| Estratab | Yes. | Hormone |
| Estrogens | Yes. | Hormone |
| Estrone | Yes. | Hormone |
| Estronol | Yes. | Hormone |
| Ethacrynic Acid | Yes. | Antihypertensive, diuretic |
| *Ethambutol* | *Defer until off drug and disease inactive.* | *Antituberculous* |
| Ethamide | Yes. | Antiglaucoma |
| Ethchlorvynol | Yes. | Sedative/hypnotic |
| Ethinamate | Yes. | Sedative/hypnotic |
| Ethinyl Estradiol | Yes. | Hormone |
| *Ethionamide* | *Defer until off drug and disease inactive.* | *Antituberculous* |
| *Ethmozine* | *No, until off drug and symptom free.* | *Anti-arrhythmic.* |
| Ethopropazine | Yes. | Anti-Parkinsonism |
| Ethosuximide | Yes. | Anticonvulsant |
| Ethoxzolamide | Yes. | Anti-glaucoma |
| Ethril | Defer 24 hrs. after course completed and feel well; if IV or IM defer 1 wk. Yes, if for acne. | Antibiotic |
| Ethylestrenol | Yes. | Anabolic androgen |
| **Etidronate** | **No, permanent deferral.** | **Inhibits bone metabolism (Paget's Disease)** |
| **Etoposide** | **Permanent deferral.** | **Chemotherapeutic agent.** |
| Etrafon | Yes, if mentally and legally responsible. | Antidepressant. Antipsychotic |
| **Etretinate** | **No, permanent deferral.** | **Antipsoriasis agent (teratogenic)** |
| Eulexin | Yes, for acne only. No if for cancer. | Antiandrogen |
| Euthroid | Yes, if controlled. | Thyroid replacement |
| Eutonyl | Defer 72 hours after course completed. | Ganglionic blocking agent and potent diuretic |
| Eutron | Defer 72 hours after course completed. | Ganglionic blocking agent and potent diuretic |
| Evex | Yes. | Hormone |
| Excedrin | Yes, if taken for allergies. Defer for 72 hours after symptoms are resolved if taken for cold/flu symptoms or for fever.  Defer 72 hrs for plateletpheresis or sole source platelets | Analgesic, ASA |
| Exgest LA | Yes, if taken for allergies. Defer for 72 hours after symptoms are resolved if taken for cold/flu symptoms. | Decongestant |
| Exna | Yes. | Diuretic, antihypertensive |
| Famotidine | Yes, if ulcer disease inactive. | Anti-ulcer medication |
| Famvir | Yes. | Antiviral Compound |
| *Fansidar* | *Defer 1 yr. If in malaria zone, otherwise, yes.* | *Antimalarial* |
| Fast Relief Pain Formula (Perrigo) | Yes, if taken for allergies. Defer for 72 hours after symptoms are resolved if taken for cold/flu symptoms or for fever.  Defer 72 hrs for plateletpheresis or sole source platelets | ASA containing analgesic |
| Fastin | Yes. | Anorexiant |
| Fedahist | Yes, if taken for allergies. Defer for 72 hours after symptoms are resolved if taken for cold/flu symptoms. | Antihistamine |
| Feldene | Yes, defer plateletpheresis donors for 3 days. | Non-steroidal anti-inflammatory |
| Fem/hrt | Yes | Progestin-estrogen combination |
| Feminone | Yes. | Hormone |
| Femogen | Yes. | Hormone |
| Fenfluramine | Yes. | Anorexiant |
| Fenoprofen | Yes, if arthritis inactive. Defer plateletpheresis donors 24 hours. | Non-steroidal anti-inflammatory |
| Feosol | Yes. | Hematinic |
| Feostat | Yes. | Hematinic |
| Ferancee | Yes. | Hematinic |
| Fergon | Yes. | Hematinic |
| Fer-In-Sol | Yes. | Hematinic |
| Fermalox | Yes. | Hematinic plus antacid |
| Fero-Folic-500 | Yes. | Iron/vitamins |
| Fero-Grad-500 | Yes. | Iron/vitamins |
| Fero-Gradumet | Yes. | Hematinic |
| Ferrocholinate | Yes. | Hematinic |
| Ferrolip | Yes. | Hematinic |
| Ferro-Sequels | Yes. | Hematinic |
| Ferrous Fumarate, Ferrous Gluconate, Ferrous Sulfate | Yes. | Hematinic |
| Festal | Yes. | Bile salt, pancreatic enzymes |
| Festalan | Yes. | Bile salt, pancreatic enzymes |
| **Fialuridine** | **No, permanent deferral.** | **Treatment for chronic Hepatitis B** |
| Filibon | Yes. | Vitamins/Minerals |
| *Finasteride (Proscar)* | *Defer for 1 month after stop taking the drug* | *Used for benign prostatic hypertrophy* |
| Fiogesic | Yes, if taken for allergies. Defer for 72 hours after symptoms are resolved if taken for cold/flu symptoms. | Decongestant, analgesic, antihistamine. |
| Fiorgen (Zenith Goldline) | Yes, if taken for allergies. Defer for 72 hours after symptoms are resolved if taken for cold/flu symptoms or for fever.  Defer 72 hrs for plateletpheresis or sole source platelets | ASA containing analgesic |
| Fioricil | Yes, if taken for allergies. Defer for 72 hours after symptoms are resolved if taken for cold/flu symptoms. | Analgesic |
| Fiorinal | Yes, if taken for allergies. Defer for 72 hours after symptoms are resolved if taken for cold/flu symptoms or for fever.  Defer 72 hrs for plateletpheresis or sole source platelets | ASA containing analgesic |
| Fiorinal with Codine Capsules (Sandoz) | Yes, if taken for allergies. Defer for 72 hours after symptoms are resolved if taken for cold/flu symptoms or for fever.  Defer 72 hrs for plateletpheresis or sole source platelets | ASA containing analgesic |
| Flagyl | Yes, if for vaginitis. Otherwise, no, defer until 1 wk. after course completed and feel well. | Antimicrobial |
| Flavoxate | Yes, if ulcer disease pain-free and/or urinary tract infection resolved. | Anticholinergic/antispasmodic |
| *Flecainide acetate* | *No, defer until off medication and stable.* | *Antiarrhythmic* |
| Flexeril | Yes. | Antispasmodic |
| Flomax | Yes | Alpha Antagonist, used for treating prostatic hypertrophy |
| Flonase | Yes | Nasal Spray for allergies |
| **Florinef** | **No, permanent deferral.** | **Mineralocorticoid** |
| Flovent (fluticasone propionate) | Yes, even if daily dose for maintenance. | Anti-inflammatory, steroid (anti-asthmatic) |
| Flubiprofen | Yes, if arthritis inactive. Defer plateletpheresis donors 24 hours. | Non-steroidal anti-inflammatory |
| Flucinonide | Yes. | Topical steroid |
| Fluconazole | Yes, if for infection of skin or nail beds. No, if for generalized (systemic) infection | Antifungal; may cause elevated ALT. |
| Flucytosine | Accept if for infection of skin or nail beds. Defer 1 wk. after course completed and condition resolved for systemic (generalized) infection. | Anti-fungal |
| **Fludrocortisone** | **No, permanent deferral.** | **Mineralocorticoid (Addison's Disease)** |
| Flumadine | Yes | Anti-influenza medication |
| Flunisolide | Yes. | Topical steroid |
| Fluorouracil | No, if for cancer; Yes, if topical. | Antineoplastic, desquamator |
| Fluoxetine | Yes. | Antidepressant |
| Fluoxymesterone | No, if for cancer; Yes, otherwise. | Hormone, anabolic steroid. Antineoplastic |
| Fluphenazine | Yes, if mentally and legally responsible. | Antipsychotic |
| Fluprednisolone | Defer 72 hrs. if P.O. or IM. Yes, if topical or intra-articular. | Corticosteroid |
| Flurazepam | Yes. | Tranquilizer |
| Fluroplex | No, if for cancer; Yes, if topical. | Antineoplastic, desquamator |
| Fluticasone propionate | Yes, even if daily dose for maintenance. | Anti-inflammatory, steroid (anti-asthmatic) |
| Folacin | Yes. | Vitamin |
| Folate | Yes. | Vitamin |
| Folic Acid | Yes. | Vitamin |
| Folinate Calcium | Yes. | Vitamin |
| Follestrol | Yes. | Hormone |
| Folvite | Yes. | Vitamin |
| Forhistal | Yes, if taken for allergies. Defer for 72 hours after symptoms are resolved if taken for cold/flu symptoms. | Antihistamine |
| Formatrix | Yes. | Hormones/vitamins |
| Fortaz | Defer 1 week. | Antibiotic |
| Fortespan | Yes. | Vitamins/minerals |
| Fosamax | Yes. | Aminobisphosponate (for osteoporosis) |
| Fosenapril | Yes, if for hypertension. No, if for heart disease. | ACE Inhibitor Antihypertensive |
| Fulvicin | Yes, if for fungal infections of nail beds. No, if for systemic (generalized) infection. | Antifungal |
| Furacin | Yes. | Topical Antibiotic |
| Furadantin | Defer 24 hrs. after course completed and feel well, if IV or IM defer 1 wk. | Antibiotic |
| Furamide | Defer 1 wk. after course completed and feel well. | Amebicide |
| Furazolidone | Defer 1 wk. after course completed and feel well. | Amebicide |
| Furosemide | Yes, if for hypertension and underlying condition not cause for deferral. | Antihypertensive, diuretic |
| Furoxone | Defer 1 wk. after course completed and feel well. | Amebicide |
| *Gamimmune* | *Defer 12 mos. if taken as hepatitis prophylaxis for known exposure.* | *Immune globulin, fractionated* |
| Gamma Benzene Hexachloride | Defer 48 hrs. after course completed. | Scabicide |
| *Gamma Globulin* | *Defer 12 mos. if taken as hepatitis prophylaxis for known exposure.* | *Immune globulin, fractionated* |
| Gammar see gamma globulin |  |  |
| Ganglionic blocking agent | Defer 72 hours. | Ganglionic blocking agent |
| Gantanol | Defer 24 hrs. after course completed and feel well; defer 1 wk. if IM or IV. | Antibiotic |
| Gantrisin | Defer 24 hrs. after course completed and feel well. | Antibiotic |
| Garamycin | Defer 24 hrs. after course completed and feel well; defer 1 wk. if IM or IV. | Antibiotic |
| Gaviscon | Yes, if ulcer disease pain-free. | Antacid |
| Gelprin (Alra) | Yes, if taken for allergies. Defer for 72 hours after symptoms are resolved if taken for cold/flu symptoms or for fever.  Defer 72 hrs for plateletpheresis or sole source platelets | ASA containing analgesic |
| Geltabs | Yes, if not renal failure patient. | Vitamin D |
| Gelusil | Yes, if ulcer disease pain-free | Antacid |
| Gemfibrozil | Yes. | Antihyperlipidemic |
| Gemnisyn | Yes, if taken for allergies. Defer for 72 hours after symptoms are resolved if taken for cold/flu symptoms.  Defer 72 hrs for plateletpheresis or sole source platelets | Analgesic |
| Genaced (Zenith) | Yes, if taken for allergies. Defer for 72 hours after symptoms are resolved if taken for cold/flu symptoms or for fever.  Defer 72 hrs for plateletpheresis or sole source platelets | ASA containing analgesic |
| Genacote (Zenith) | Yes, if taken for allergies. Defer for 72 hours after symptoms are resolved if taken for cold/flu symptoms or for fever.  Defer 72 hrs for plateletpheresis or sole source platelets | ASA containing analgesic |
| Genprin (Zenith Goldline) | Yes, if taken for allergies. Defer for 72 hours after symptoms are resolved if taken for cold/flu symptoms or for fever.  Defer 72 hrs for plateletpheresis or sole source platelets | ASA containing analgesic |
| Gensan (Zenith Goldline) | Yes, if taken for allergies. Defer for 72 hours after symptoms are resolved if taken for cold/flu symptoms or for fever.  Defer 72 hrs for plateletpheresis or sole source platelets | ASA containing analgesic |
| Gentamicin | Defer 24 hrs. after course completed and feel well; defer 1 wk. if IM or IV. | Antibiotic |
| Genuine Bayer Aspirin | Yes, if taken for allergies. Defer for 72 hours after symptoms are resolved if taken for cold/flu symptoms or for fever.  Defer 72 hrs for plateletpheresis or sole source platelets | ASA containing analgesic |
| Geocillin | Defer 24 hrs. after course completed and feel well. | Antibiotic |
| Geopen | Defer 24 hrs. after course completed and feel well; if IM or IV defer 1 wk. | Antibiotic |
| Gerilets | Yes. | Vitamins/minerals |
| Gevral | Yes. | Vitamins/minerals |
| **Gitaligin** | **No, permanent deferral.** | **Cardiac Glycoside** |
| **Gitalin** | **No, permanent deferral.** | **Cardiac Glycoside** |
| Glipizide | Yes. | Oral hypoglycemic |
| Glucophage | Yes. | Oral hypoglycemic |
| Glucotrol | Yes. | Oral hypoglycemic |
| Glutethimide | Yes. | Hypnotic/sedative |
| Glyburide | Yes. | Oral hypoglycemic |
| Glyceryl Guaiacolate | Yes, if taken for allergies. Defer for 72 hours after symptoms are resolved if taken for cold/flu symptoms. | Expectorant |
| Glycopyrrolate | Yes, if ulcer disease pain-free. | Anticholinergic, antispasmodic |
| Glyestrin | Yes. | Hormone |
| Glynase PresTab | Yes. | Oral hypoglycemic |
| Glysennid | Yes. | Laxative |
| Glyset | Yes. | Oral hypoglycemic |
| G-mycin | Defer 24 hrs. after course completed and feel well. | Antibiotic |
| Gold Sodium-thiomalate | Yes, if arthritis inactive. | Antirheumatic |
| Gonadotropin, Human | Yes. | Infertility |
| Goody’s Extra Strength (Goody’s) | Yes, if taken for allergies. Defer for 72 hours after symptoms are resolved if taken for cold/flu symptoms or for fever.  Defer 72 hrs for plateletpheresis or sole source platelets | ASA containing analgesic |
| Goody’s Headache Powder (Goody’s) | Yes, if taken for allergies. Defer for 72 hours after symptoms are resolved if taken for cold/flu symptoms or for fever.  Defer 72 hrs for plateletpheresis or sole source platelets | ASA containing analgesic |
| Gramicidin | Yes. | Topical antibiotic and steroid |
| Grifulvin, Grisactin, Giseofulvin, Gris-PEG | Yes, if for fungal infections of skin or nail beds. No, if for systemic (generalized) infection. | Antifungal |
| Growth hormone | See medical criteria section. |  |
| Guaifenesin | Yes, if taken for allergies. Defer for 72 hours after symptoms are resolved if taken for cold/flu symptoms. | Expectorant |
| Guanethidine | Defer 72 hours. | Ganglionic blocking agent |
| Guarabenz | Yes. | Antihypertensive |
| Gynergen | Yes. | Vasoconstrictor |
| Gynorest | Yes. | Hormone |
| Halcion | Yes. | Antianxiety agent |
| Haldol | Yes, if donor legally and mentally responsible. | Antipsychotic |
| Haldrone | Defer 72 hrs. if P.O. or IM. Yes, if topical or intra-articular. | Corticosteroid |
| Haley's M-O | Yes. | Laxative |
| Halfprin 81, EC Tab (Kramer) | Yes, if taken for allergies. Defer for 72 hours after symptoms are resolved if taken for cold/flu symptoms or for fever.  Defer 72 hrs for plateletpheresis or sole source platelets | ASA containing analgesic |
| Haloperidol | Yes, if donor legally and mentally responsible. | Antipsychotic |
| Halotestin | No, if for cancer; otherwise, yes. | Androgen |
| Halprin (Kramer) | Yes, if taken for allergies. Defer for 72 hours after symptoms are resolved if taken for cold/flu symptoms or for fever.  Defer 72 hrs for plateletpheresis or sole source platelets | Aspirin containing analgesic |
| Harmonyl | Yes. | Antihypertensive |
| HBIG | Defer 12 months. | Biological |
| HCG | Yes. | Hormone |
| *Hedulin* | *Defer pending medical evaluation with cessation of drug.* | *Anticoagulant* |
| *Heparin* | *Defer pending medical evaluation with cessation of drug.* | *Anticoagulant* |
| *Hepathrom* | *Defer pending medical evaluation with cessation of drug.* | *Anticoagulant* |
| Hepatitis B Immune Globulin | Defer 12 months. | Biological |
| *Heprinar* | *Defer pending medical evaluation with cessation of drug.* | *Anticoagulant* |
| Hetacillin | Defer 24 hrs after course completed and feel well; if IV or IM defer 1 wk. | Antibiotic |
| Hetrazan | Defer 1 wk. after course completed and feel well. | Antihelminthic |
| Hexa-Betalin | Yes. | Vitamin |
| Hexadrol | Defer 72 hrs. if P.O. or IM Yes, if intra-articular or topical. | Corticosteroid |
| Hexalet | Defer 24 hrs. after course completed and feel well. | Urinary antibacterial |
| Hexavibex | Yes. | Vitamin |
| Hexavitamins | Yes. | Vitamins/minerals |
| Hexestrol | Yes. | Hormone |
| Hiprex | Defer 24 hrs. after course completed and feel well. | Urinary antibacterial |
| Hismanal | Yes, if taken for allergies. Defer for 72 hours after symptoms are resolved if taken for cold/flu symptoms. | Antihistamine |
| Hispril | Yes, if taken for allergies. Defer for 72 hours after symptoms are resolved if taken for cold/flu symptoms. | Antihistamine |
| Hista-Derfule | Yes, if taken for allergies. Defer for 72 hours after symptoms are resolved if taken for cold/flu symptoms. | Antihistamine/analgesic |
| Histadyl E.C. | Yes, if taken for allergies. Defer for 72 hours after symptoms are resolved if taken for cold/flu symptoms.  Defer 72 hrs for plateletpheresis or sole source platelets | Antitussive, expectorant, decongestant |
| Histalet | Yes, if taken for allergies. Defer for 72 hours after symptoms are resolved if taken for cold/flu symptoms. | Antihistamine, decongestant |
| Histaspan | Yes, if taken for allergies. Defer for 72 hours after symptoms are resolved if taken for cold/flu symptoms. | Antihistamine |
| HMG | Yes. | Gonadotropin |
| Homatropine | Yes, if ulcer disease pain-free. | Anticholinergic/antispasmodic |
| Hormonin | Yes. | Hormones |
| **Humagro** | **PD** | **Lamb pituitary and herb compound. Sold in health food stores.** |
| Humatin | Defer 1 wk. after course completed and feel well. | Amebicide |
| Humatrope (Somatropin) | Yes. | Growth hormone (recombinant) |
| Humulin | Yes, if dosage controlled and stable. | Anti-hyperglycemic |
| Hybephen | Yes, if ulcer disease pain-free. | Anticholinergic/antispasmodic |
| Hycodan | Yes, if taken for allergies. Defer for 72 hours after symptoms are resolved if taken for cold/flu symptoms. | Antitussive, anticholinergic |
| Hycomine | Yes, if taken for allergies. Defer for 72 hours after symptoms are resolved if taken for cold/flu symptoms. | Antitussive, decongestant |
| Hycotuss | Yes, if taken for allergies. Defer for 72 hours after symptoms are resolved if taken for cold/flu symptoms. | Antitussive, expectorant |
| Hydeltra-T.B.A. | Defer 72 hrs. if P.O. or IM. Yes, if topical or intra-articular. | Corticosteroid |
| Hydergine | Yes. | Vasodilator, improves cognition |
| Hydralazine, Hydralin | Yes, if for hypertension. No, if for congestive heart failure. | Antihypertensive. Vasodilator |
| **Hydrea** | **No** | **Chemotherapy medication** |
| Hydro-cholorothiazide | Yes. | Antihypertensive, diuretic |
| Hydrocil | Yes. | Laxative |
| Hydrocodone | Yes, if taken for allergies. Defer for 72 hours after symptoms are resolved if taken for cold/flu symptoms. | Antitussive |
| Hydrocortisone, Hydrocortone | Defer 72 hrs. if P.O. or IM. Yes, if topical or intra-articular. | Corticosteroid |
| HydroDIURIL | Yes. | Antihypertensive, diuretic |
| Hydroflumethiazide | Yes. | Antihypertensive, diuretic |
| Hydromorphone | Yes, if not abuser. | Narcotic analgesic |
| Hydromox | Yes. | Antihypertensive, diuretic |
| Hydropres | Yes. | Antihypertensive, diuretic |
| Hydroxocobalamin | Yes. | Vitamin B_12_ |
| *Hydroxychloroquine* | *No, defer 1 yr. if for malaria and in malarial zone; if not in malaria zone or given for other indication then yes.* | *Antimalarial. Anti-arthritic, anti-inflammatory* |
| Hydroxyprogesterone | No, permanent deferral if cancer; otherwise, yes | Hormone |
| Hydroxyzine | Yes. Yes if taken for allergies. Defer for 72 hours after symptoms are resolved if taken for cold/flu symptoms. | Antihistamine, anti-anxiety |
| Hygroton | Yes. | Antihypertensive, diuretic |
| Hyoscyamine | Yes, if ulcer disease pain-free. | Anticholinergic/antispasmodic |
| Hypertussis | Yes. | Pertussis immune globulin |
| Hyrye | Yes. | Vitamin |
| Hytakerol | Yes, unless renal failure patient. | Vitamin D |
| Hytrin | Yes. | Antihypertensive |
| Hyzarr | Yes. | Antihypertensive |
| Iberet | Yes. | Vitamins/hematinic |
| Ibuprofen | Yes, if arthritis inactive. No, if for fever. Defer plateletpheresis donor 24 hrs. | Non-steroidal. Anti-inflammatory analgesic |
| Ilosone, Ilotycin | Defer 24 hrs. after course completed and feel well. | Antibiotic |
| Ilozyme | Yes. | Enzyme replacement |
| Imavate | Yes. | Tricyclic antidepressant |
| Imdur | Yes | Vasodilator |
| Imferon | Yes, evaluate underlying condition. | Iron replacement, migraine headaches. |
| Imipramine | Yes. | Tricyclic antidepressant |
| Imitrex | Accept if migraine symptoms are resolved. Defer until next day after receiving injection. | Blocks production of Serotonin |
| Immune globulin see gamma globulin |  |  |
| Immune Globulin, ISG | Defer 12 mos. if taken as hepatitis prophylaxis for known exposure. | Immune globulin, fractionated |
| Imodium | Yes, if symptom free. | Anti-diarrheal |
| **Imuran** | **No, permanent deferral.** | **Immunomodulator** |
| Indapamide | Yes, if for hypertension. No, if for congestive heart failure. | Antihypertensive, diuretic |
| Inderal | Yes, if for hypertension, migraine headaches. No, if antianginal for angina or arrhythmias. Yes, if for Mitral Valve Prolapse. | Antiarrhythmic, anti-hypertensive |
| Inderide | Yes, if for hypertension. No, if angina, arrhythmias. | Antihypertensive, diuretic |
| Indocin | Yes, if arthritis inactive. Defer plateletpheresis donors 24 hours. | Non-steroidal anti-inflammatory |
| Indomethacin | Yes, if arthritis inactive. Defer plateletpheresis donors 24 hours. | Non-steroidal anti-inflammatory |
| INH | Yes, if for prophylaxis. Otherwise, defer until course of medication completed and disease inactive. | Antituberculous |
| Initia Drop with Fluoride | Yes. | Vitamins/minerals |
| *Isordil* | *Medical Director evaluation required* | *Anti-angina agent* |
| **Insulin** | **Yes, if dosage controled and stable.**  **No, if bovine (beef) insulin injected since 1980. Then, indefinite deferral for vCJD risk.** | **Anti-hyperglycemic** |
| Intal | Yes, even if daily dose for maintenance. | Anti-asthmatic |
| **Interferons: Alfa-2A, Alfa-2B, Alfa-N3, Gamma-1B** | **No, permanent deferral for Cancer, Hepatitis and Chronic Granulomatosis. Venereal warts; defer 12 months after treatment and condition resolved. All othersevaluate underlying condition. Defer for 12 months if treated with Interferon Alfa-N3. (Human derived Interferon)** | **Anti-leukemic** |
| Inversine | Defer 72 hrs. | Ganglionic blocking agent |
| Iodoquinol | Defer 1 wk. after course completed and feel well. | Amebicide |
| Ionamin | Yes. | Anorexiant |
| Ipol | Accept immediately if symptom-free. | Vaccine: Poliovirus Inactivated (injectible) |
| Ircon | Yes. | Hematinic |
| Iron-Polysaccharide Complex | Yes. | Hematinic |
| Ismelin | Defer 72 hours. | Ganglionic blocking agent |
| ISMO | Defer until off drug and free of symptoms. | Antianginal |
| Isocarboxazid | Defer 72 hours. | Ganglionic blocking agent anti-depressant (MAO inhibitor) |
| Isoclor | Yes, if taken for allergies. Defer for 72 hours after symptoms are resolved if taken for cold/flu symptoms. | Antihistamine, decongestant |
| Isollyl | Yes, if taken for allergies. Defer for 72 hours after symptoms are resolved if taken for cold/flu symptoms or for fever.  Defer 72 hrs for plateletpheresis or sole source platelets | Analgesic, ASA |
| Isoniazid | Yes, if for prophylaxis. Otherwise, defer until course of medication completed and disease inactive. | Antituberculous |
| Isoproterenol | Yes, even if daily dose for maintenance. | Bronchodilator |
| Isoptin | Yes, if for hypertension. Otherwise, no. | Antihypertensive, antianginal, antiarrhythmic |
| Isopto-Carpine | Yes. | Antiglaucoma |
| *Isordil* | *Must be evaluated by Medical Director.* | *Anti-anginal* |
| *Isosorbide Dinitrate* | *Must be evaluated by Medical Director.* | *Antianginal* |
| Isotretinoin (Accutane) | Defer 1 month after last dose. | Vitamin A derivative |
| Isoxsuprine | Yes. (check for orthostatic change) | Beta adrenergic stimulants |
| Israpidine | Yes. | Calcium antagonist (Dynacirc) |
| Isuprel | Yes, even if daily dose for maintenance. | Bronchodilator |
| Itraconazole | Yes if for superficial fungal infection. No, if for systemic (generalized) infection. | Anti-fungal |
| *IVIG* | *Evaluate indication for use for deferral.* | *IV Immune globulin* |
| Janimine | Yes. | Tricyclic antidepressant |
| Kadalex | Yes. | Electrolyte replacement |
| Kafocin | Defer 24 hrs. after course completed and feel well; if IV or IM defer 1 wk. | Antibiotic |
| Kanamycin, Kantrex | Defer 24 hrs. after course completed and feel well; if IV or IM defer 1 week. If for TB defer until off drug and disease inactive. | Antibiotic |
| Kaochlor | Yes. | Electrolyte replacement |
| Kaolin | Yes, if symptom free. | Antidiarrheal |
| Kaon | Yes. | Electrolyte replacement |
| Kaopectate | Yes, if symptom free. | Antidiarrheal |
| Kappadione | Evaluate underlying condition. | Vitamin K |
| Karaya Gum | Yes. | Laxative |
| Kasof | Yes. | Laxative |
| Kato | Yes. | Electrolyte replacement |
| Kay Ciel | Yes. | Electrolyte replacement |
| **Kayexalate** | **No, permanent deferral.** | **Potassium binding resin** |
| Keflex | Defer 24 hrs. after course completed and feel well. | Antibiotic |
| Keflin | Defer 1 wk. | Antibiotic |
| Keftab | Defer 24 hrs. after course completed and feel well, if IM or IV defer one week. | Antibiotic |
| Kefzol | Defer 1 wk. | Antibiotic |
| Kemadrin | Yes. | Anti-Parkinsonism |
| Kenacort | Defer 72 hrs. if P.O. or IM; Yes, if topical or intra-articular. | Corticosteroid |
| Kerlone | Yes. | Antihypertensive, Beta blocker. |
| Ketochol | Yes. | Bile salt |
| Ketocholanic Acid | Yes. | Bile salt |
| Ketoconazole | Defer 1 wk. after course completed and feel well. | Antifungal |
| Ketoprofen | Yes, if arthritis inactive. Defer plateletpheresis donors 24 hrs. | Non-steroidal anti- inflammatory drug |
| Ketrolac | Yes. | Topical anti-inflammatory |
| Kinesed | Yes, if ulcer disease pain-free. | Anticholinergic/antispasmodic |
| Klonopin | Yes. | Anticonvulsant |
| K-Lor, K-Lyte, K-Lyte/Cl | Yes. | Electrolyte replacement |
| Klor-Con | Yes. | Electrolyte replacement |
| Klorvess | Yes. | Electrolyte replacement |
| **Koate** | **No, permanent deferral.** | **Antihemophilic Factor** |
| Kolantyl | Yes, if ulcer disease pain-free. | Antacid |
| Konakion | Evaluate underlying condition. | Vitamin K |
| Kondremul | Yes. | Laxative |
| Konsyl | Yes. | Laxative |
| *Konyne* | *No, permanent deferral if hemophilia B; otherwise, 12 months.* | *Blood product* |
| Kudrox | Yes, if ulcer disease pain-free. | Antacid |
| Kutrapressin | Yes, evaluate skin condition being treated. | Liver derivative |
| Ku-Zyme HP | Yes. | Enzyme replacement |
| Kwell | Defer 48 hrs. | Scabicide |
| Kytril | Drug OK, but not if associated with chemotherapy. | Antiemetic |
| L.A. Formula | Yes. | Laxative |
| Labetalol | Yes. | Antihypertensive |
| Lactaid | Yes. | Enzyme for lactose intolerance |
| Lactinex | Yes, if symptom free. | Antidiarrheal |
| Lactobacillus Cultures | Yes, if symptom free. | Antidiarrheal |
| *Lactulose* | *No, permanent deferral if liver disease; otherwise, yes.* | *Laxative* |
| Lamine | Yes, as antiemetic. Yes, if taken for allergies. Defer for 72 hours after symptoms are resolved if taken for cold/flu symptoms. | Antihistamine, antiemetic |
| Lamisil | Yes. | Anti-fungal |
| **Lamprene** | **No, permanent deferral.** | **Anti-leprosy** |
| **Lanoxin** | **No, permanent deferral.** | **Cardiac Glycoside** |
| **Lantoside C** | **No, permanent deferral.** | **Cardiac Glycoside** |
| *Lariam, Laricam* | *No, defer 1 year if in malarial area, defer for 3 years if used to treat acute malaria infection, otherwise, yes.* | *Anti-malarial* |
| Larodopa | Yes. | Anti-Parkinsonism |
| Larotid | Defer 24 hrs. after course completed and feel well; if IV or IM defer 1 wk. | Antibiotic |
| Lasix | Yes, if for hypertension and underlying condition not cause for deferral. | Antihypertensive, diuretic |
| Lederade Sequels | Yes, if taken for allergies. Defer for 72 hours after symptoms are resolved if taken for cold/flu symptoms. | Decongestant, antihistamine |
| Ledercillin | Defer 24 hrs. after course completed and feel well. | Antibiotic |
| Ledermycin | Defer 24 hrs. after course completed and feel well; if IV or IM defer 1 wk. Yes, if for acne. | Antibiotic |
| **Leflunomide** | **No** | **See Arava** |
| Lemiserp | Yes, watch for orthostatic pressure changes. | Antihypertensive |
| Lente Iletin, Lente Insulin | Yes, if dosage controlled and stable. | Hypoglycemic |
| Leritine | Yes, if not abuser. | Narcotic, analgesic |
| Lescol | Yes. | Cholesterol lowering agent. |
| Letter. | Yes. | Thyroid replacement |
| **Leucovorin** | **No, permanent deferral.** | **Corrects antifolate effect of methotrexate** |
| **Leukeran** | **No, permanent deferral.** | **Antineoplastic** |
| **Leuprolide** | **No, permanent deferral.** | **GRH agent used for prostate cancer patients** |
| *Levamisole* | *Defer 1 wk. if for worms; otherwise, permanent deferral.* | *Antihelminthic, immunomodulator, anti-rheumatoid* |
| Levatol | Yes | Adrenergic Receptor Antagonist |
| Levbid | Yes | Used for peptic ulcer |
| Levocarnitine | Yes. | Carnitine replacement |
| Levodopa | Yes. | Anti-Parkinsonism |
| Levo-Dromoran | Yes, if not abuser. | Narcotic, analgesic |
| Levoid | Yes. | Thyroid replacement |
| Levaquin (Levofloxacin) | Defer 24 hours after course completed and feel well. | Broadspectrum Antibiotic |
| Levopa | Yes. | Anti-Parkinsonism |
| **Levophed** | **No.** | **Restore B/P in hypotension** |
| Levorphanol | Yes, if not abuser. | Narcotic, analgesic |
| Levostat | Yes. | Lipid control agent |
| Levothroid | Yes. | Thyroid replacement |
| Levothyroxine | Yes. | Thyroid replacement |
| Levsin, Levsinex | Yes, if ulcer disease pain-free. | Anticholinergic/antispasmodic |
| Lexxel | Yes. | Antihypertensive |
| Librax | Yes, if ulcer disease pain-free. | Anticholinergic/antispasmodic |
| Libritabs | Yes. | Psychotropic |
| Librium | Yes. | Tranquilizer |
| Lidex | Yes. | Topical steroid |
| Lidone | Yes, if donor mentally and legally responsible. | Antipsychotic |
| Limbitrol | Yes. | Antidepressant, tranquilizer |
| Lincocin | Defer 24 hrs. after course completed and feel well; if IV or IM defer 1 wk. | Antibiotic |
| Lincomycin | Defer 24 hrs. after course completed and feel well; if IV or IM defer 1 wk. | Antibiotic |
| Lindane | Defer 48 hrs. | Scabicide |
| Lioresal | Yes. | Muscle relaxant |
| Liothyronine | Yes. | Thyroid replacement |
| Liotrix | Yes. | Thyroid replacement |
| Lipitor | Yes. | Antilipemic |
| Lipitrol | Yes. | Over-the-counter weight loss compound |
| *Lipo-Hepin* | *Defer pending medical evaluation with cessation of drug.* | *Anticoagulant* |
| Lipo-Lutin | Yes. | Hormone |
| Lipo-Nicin | Yes. | Vasodilator |
| *Liquaemin Sodium* | *Defer pending medical evaluation with cessation of drug.* | *Anticoagulant* |
| *Liquamar* | *Defer pending medical evaluation with cessation of drug.* | *Anticoagulant* |
| Liqui-Cee | Yes. | Vitamin C |
| Liquid Petrolatum | Yes. | Laxative |
| Liquiprim | Yes | Acetaminophen (liquid) |
| Liquiprin | Yes, if taken for allergies. Defer for 72 hours after symptoms are resolved if taken for cold/flu symptoms.  Defer 72 hrs for plateletpheresis or sole source platelets | Analgesic, antipyretic |
| Lisacort | Defer 72 hrs. if P.O. and IM; Yes, if topical and intra-articular. | Corticosteroid |
| Lisinopril | Yes, if for hypertension. | Antihypertensive |
| Lithane | Yes, if mentally and legally responsible. | Antimania |
| Lithionate | Yes, if mentally and legally responsible. | Antimania |
| Lithium | Yes, if mentally and legally responsible. | Antimania |
| Lithobid | Yes, if mentally and legally responsible. | Antimania |
| Lithonate | Yes, if mentally and legally responsible. | Antimania |
| Lisinopril | Yes, if for hypertension | ACE inhibitor |
| Lo/Ovral | Yes. | Oral contraceptive |
| Lodine | Yes, if arthritis inactive. Defer plateletpheresis donors24 hours. | Non-steroidal anti-inflammatory drug |
| Loestrin | Yes. | Oral contraceptive |
| Lomotil | Yes, if symptom free. | Anti-diarrheal |
| **Lomustine** | **No, permanent deferral.** | **Antineoplastic** |
| Loniten | Yes. | Antihypertensive |
| Loperamide | Yes, if symptom free. | Antidiarrheal |
| Lopid | Yes. | Antihyperlipidemic |
| Lopressor | Yes, if for hypertension. No, if for angina. | Antianginal, antihypertensive |
| Lopurin | Yes. | Uricosuric agent |
| Lorazepam | Yes. | Tranquilizer |
| Lorcet | Yes, if not an abuser | Narcotic analgesic |
| Lorelco | Yes. | Antihyperlipidemic |
| Loridine | Defer 24 hrs. after course completed and feel well; if IV or IM defer 1 wk. | Antibiotic |
| Lortab | Yes, if not abuser. | Narcotic analgesic. |
| Lotensin | Yes, if for hypertension. No, of for heart disease. | ACE Inhibitor. Antihypertensive |
| Lotrel | Yes , for hypertension. | ACE Inhibitor |
| Lotrimin | Yes. | Topical antifungal |
| Lotrisone | Defer 72 hrs after use completed if IV, IM or PO, otherwise, yes. | Corticosteroid Anti-inflammatory |
| Lovastatin | Yes. | Lipid control agent |
| Loxapine | Yes, if mentally and legally responsible. | Antipsychotic |
| Loxitane | Yes, if mentally and legally responsible. | Antipsychotic |
| Lozol | Yes, if for hypertension. | Antihypertensive, diuretic |
| LSD | Yes, if one or two incident experimentation. No, permanent deferral if chronic user. | Psychotogenic |
| L-Throxin | Yes. | Thyroid replacement |
| L-Tyrosine | Yes. | Antidepressant. Treatment for narcolepsy. |
| Ludiomil | Yes. | Antidepressant |
| Lufyllin | Yes, even if daily dose for maintenance. | Bronchodilator |
| Luminal | Yes. | Sedative |
| *Lupron* | *No, permanent deferral if for cancer. Otherwise, evaluate underlying condition.* | *Synthetic hormone agent (GRH) used for prostate cancer patients* |
| Luvox | Yes. | Used to treat obsessive compulsive disorder. |
| **Lypressin** | **No, permanent deferral.** | **Antidiuretic hormone** |
| **Lysodren** | **No, permanent deferral.** | **Antineoplastic** |
| Maalox | Yes, if ulcer disease pain-free. | Antacid |
| Macrodantin | Defer 24 hrs. after course completed and feel well. Yes, if for prophylactic use. | Antibiotic |
| Madelamine | Defer 24 hrs after course completed and feel well. | Antibacterial |
| Magan | Yes, if arthritis inactive | Antiarthritic |
| Magcyl | Yes. | Laxative |
| Magnaprin (Rugby) | Yes, if taken for allergies. Defer for 72 hours after symptoms are resolved if taken for cold/flu symptoms or for fever.  Defer 72 hrs for plateletpheresis or sole source platelets | ASA containing analgesic |
| Magnaprin Arthritis Strength (Rugby) | Yes, if taken for allergies. Defer for 72 hours after symptoms are resolved if taken for cold/flu symptoms or for fever.  Defer 72 hrs for plateletpheresis or sole source platelets | ASA containing analgesic |
| Magnesium Carbonate | Yes, if ulcer disease pain-free. | Antacid |
| Magnesium Citrate | Yes. | Laxative |
| Magnesium Hydroxide | Yes, if ulcer disease pain free. | Antacid |
| Magnesium Oxide | Yes, if ulcer disease pain free. | Antacid |
| Magnesium Phosphate | Yes, if ulcer disease pain free. | Antacid |
| Magnesium Salicylate | Yes, if arthritis inactive. | Anti-arthritic |
| Magnesium Sulfate | Yes. | Laxative |
| Magnesium Trisilicate | Yes, if ulcer disease pain-free. | Antacid |
| Malcotran | Yes, if ulcer disease pain-free. | Anticholinergic/antispasmodic |
| Maltsupex | Yes. | Laxative |
| Mandacon | Defer 24 hrs after course completed and feel well. | Antibacterial |
| Mandelets | Defer 24 hrs after course completed and feel well. | Antibacterial |
| *Mannex* | *No, until off drug and free of symptoms.* | *Anti-anginal* |
| *Mannitol Hexanitrate* | *No, until off medication and free of symptoms.* | *Anti-anginal* |
| Mansil | Defer 1 wk. after course completed and feel well. | Antihelminthic |
| Maolate | Yes. | Muscle relaxant |
| Maprotiline | Yes. | Antidepressant |
| Marax | Yes, even if daily dose for maintenance use. | Bronchodilator |
| Marezine | Yes, if taken for allergies. Defer for 72 hours after symptoms are resolved if taken for cold/flu symptoms. | Antihistamine |
| Margesic Compound No 65 (Vortech) | Yes, if taken for allergies. Defer for 72 hours after symptoms are resolved if taken for cold/flu symptoms or for fever.  Defer 72 hrs for plateletpheresis or sole source platelets | ASA containing analgesic |
| Marijuana | Yes, if not under the influence. | Street drug |
| **Marinol** | **Permanent deferral** | **Treats anorexia present in AIDS** |
| Marnal (Vorteck) | Yes, if taken for allergies. Defer for 72 hours after symptoms are resolved if taken for cold/flu symptoms or for fever.  Defer 72 hrs for plateletpheresis or sole source platelets | ASA containing analgesic |
| Marplan | Defer 72 hours. | Antidepressant, ganglionic blocking agent |
| Maso-Bemate | Yes. | Sedative/hypnotic |
| Matropinal | Yes, if ulcer disease pain-free. | Anticholinergic/antispasmodic |
| **Matulane** | **No, permanent deferral.** | **Antineoplastic** |
| Mavik | Yes, if for hypertension. No, if for heart disease. | Antihypertensive, ACE inhibitor |
| Maxibolin | Yes. | Hormone |
| Maximum Bayer | Yes, if taken for allergies. Defer for 72 hours after symptoms are resolved if taken for cold/flu symptoms or for fever.  Defer 72 hrs for plateletpheresis or sole source platelets | ASA containing analgesic |
| Maximum Strength Arthritis Anacin | Yes, if taken for allergies. Defer for 72 hours after symptoms are resolved if taken for cold/flu symptoms or for fever.  Defer 72 hrs for plateletpheresis or sole source platelets | ASA containing analgesic |
| *Maxitrate* | *No, until off medication and symptom free.* | *Anti-anginal* |
| Maxzide | Yes. | Antihypertensive |
| Mazindol | Yes. | Anorexiant |
| Measurin | Yes, if taken for allergies. Defer for 72 hours after symptoms are resolved if taken for cold/flu symptoms or for fever.  Defer 72 hrs for plateletpheresis or sole source platelets | Analgesic, antipyretic, anti-inflammatory, ASA. |
| Mebaral | Yes. | Anti-convulsant, sedative |
| Mebendazole | Defer 1 wk. after course completed and feel well. | Antihelminthic |
| Mebroin | Yes. | Anticonvulsant |
| Mecamylamine | Yes | Antihypertensive |
| **Mecaptopurine** | **No, permanent deferral.** | **Antineoplastic** |
| Meclan | Yes. | Topical antibiotic |
| Meclizine | Yes, if taken for allergies. Defer for 72 hours after symptoms are resolved if taken for cold/flu symptoms. | Antihistamine |
| Meclofenamate | Yes, if arthritis inactive. Defer plateletpheresis donors 24 hrs. | Non-steroidal anti-inflammatory |
| Meclomen | Yes, if arthritis inactive. Defer plateletpheresis donors 24 hrs. | Non-steroidal anti-inflammatory |
| Mediatric | Yes. | Hormone, vitamin |
| Medihaler-Epi | Yes, even if daily dose for maintenance. | Bronchodilator |
| Medihaler-Iso | Yes, even if daily dose for maintenance. | Bronchodilator |
| Medipren | Yes, if arthritis inactive. Defer plateletpheresis donors 24 hrs. | Non-steroidal anti-inflammatory agent |
| Medral | Yes, even if daily dose for maintenance. | Bronchodilator |
| Medrol | Defer 72 hrs, if P.O. or IM. Yes, if topical or intra-articular. | Corticosteroid |
| Medroxyprogesterone | No, if for cancer; otherwise, yes. | Hormone |
| Mefenamic Acid | Yes, if arthritis inactive.  Yes, if taken for allergies. Defer for 72 hours after symptoms are resolved if taken for cold/flu symptoms or for fever. | Non-steroidal anti-inflammatory, analgesic, anti-pyretic |
| Megace | No, if for cancer; otherwise, yes. | Antineoplastic/hormone |
| Megestrol | No, if for cancer; otherwise, yes | Antineoplastic/hormone |
| Mellaril | Yes, if mentally and legally responsible. | Antipsychotic, tranquilizer |
| **Melphalan** | **No, permanent deferral.** | **Antineoplastic** |
| *Menadione, Menadiol* | *Evaluate underlying condition.* | *Vitamin K* |
| Menagen | Yes. | Hormone |
| Menest | Yes. | Hormone |
| Menotropins | Yes. | Gonadotropin |
| Menrium | Yes. | Hormone/tranquilizer |
| Mentabal | Yes. | Anticonvulsant/sedative |
| Mepenzolate | Yes, if ulcer disease pain-free | Anticholinergic/antispasmodic |
| Mepergan Fortis | Yes, if not abuser. | Narcotic, analgesic |
| Meperidine | Yes, if not abuser. | Narcotic, analgesic |
| Mephobarbital | Yes. | Anticonvulsant, sedative |
| Mephoral | Yes. | Anticonvulsant, sedative |
| *Mephyton* | *Evaluate underlying condition.* | *Vitamin K* |
| Meprednisone | Yes if topical or intra-articular. | Corticosteroid |
| Mepriam | Yes, if not abuser. | Sedative/hypnotic |
| Mepro Aspirin (Eon) | Yes, if taken for allergies. Defer for 72 hours after symptoms are resolved if taken for cold/flu symptoms or for fever.  Defer 72 hrs for plateletpheresis or sole source platelets | ASA containing analgesic |
| Mepro Compound (Teva) | Yes, if taken for allergies. Defer for 72 hours after symptoms are resolved if taken for cold/flu symptoms or for fever.  Defer 72 hrs for plateletpheresis or sole source platelets | ASA containing analgesic |
| Meprobromate (Moore) | Yes, if taken for allergies. Defer for 72 hours after symptoms are resolved if taken for cold/flu symptoms or for fever.  Defer 72 hrs for plateletpheresis or sole source platelets | ASA containing analgesic |
| Meprobamate | Yes, if not abuser. | Sedative/hypnotic |
| Meprocarbomal | Yes, if taken for allergies. Defer for 72 hours after symptoms are resolved if taken for cold/flu symptoms or for fever.  Defer 72 hrs for plateletpheresis or sole source platelets | ASA containing analgesic |
| Meprocon | Yes, if not abuser. | Sedative/hypnotic |
| Meprospan | Yes, if not abuser. | Sedative/hypnotic |
| **Mercaptopurine** | **No, permanent deferral.** | **Antineoplastic** |
| Meridia | Yes. | Appetite suppressant |
| Mesalamine | Yes, if inflammatory bowel disease is under control. No, if for Crohn's Disease. | Anti-inflammatory enema |
| Mesantoin | Yes. | Anticonvulsant |
| Mesopin | Yes, if ulcer disease pain-free | Anticholinergic/antispasmodic |
| Mesoridazine | Yes, if mentally and legally responsible. | Phenothiazide tranquilizer |
| **Mestinon** | **No, permanent deferral.** | **Anticholinesterase** |
| Metahydrin | Yes. | Antihypertensive, diuretic |
| Metamucil | Yes. | Laxative |
| Metandren | No, if for cancer; otherwise, yes. | Hormone |
| Metaprel | Yes, even if daily dose for maintenance. | Bronchodilator |
| Metatensin | Yes, be alert for orthostatic pressure changes. | Antihypertensive |
| Metaxalone | Yes. | Muscle relaxant (may elevate liver function tests) |
| Methacycline | Defer 24 hours after course completed and feel well; if IV or IM defer 1 week. Yes if for acne. | Antibiotic |
| Methadone | Yes, if not abuser. | Narcotic, analgesic |
| Methamphetamine | Yes. | Anorexiant |
| Methandrostenolone | Yes. | Anabolic steroid |
| Methazolamide | Yes. | Antiglaucoma |
| Methdilazine | Yes, if taken for allergies. Defer for 72 hours after symptoms are resolved if taken for cold/flu symptoms. | Antihistamine |
| Methenamine | Defer 24 hours after course completed and feel well. | Antibacterial |
| Methergine | Defer 6 weeks after use. | Uterine stimulant |
| Methicillin | Defer 1 week, if IV or IM use. | Antibiotic |
| Methimazole | Yes. | Antithyroid |
| Methixene | Yes, if ulcer disease pain-free. | Anticholinergic/antispasmodic |
| Metho-500 | Yes. | Sedative/hypnotic |
| Methocarbamol | Yes. | Sedative/hypnotic |
| **Methosarb** | **No, permanent deferral.** | **Antineoplastic** |
| *Methotrexate* | *No, permanent deferral unless used for non-neoplastic disease. Evaluate underlying condition.* | *Antineoplastic* |
| Methoxsalen | Yes. | Repigmentation drug |
| Methscopolamine | Yes, if ulcer disease pain-free. | Anticholinergic/antispasmodic |
| Methyclothiazide | Yes. | Antihypertensive, diuretic |
| **Methyl CCNU** | **No, permanent deferral.** | **Antineoplastic** |
| Methylcellulose | Yes. | Laxative |
| Methylergonovine | Defer 6 weeks after use. | Uterine Stimulant |
| Methylodopa | Yes. | Antihypertensive |
| Methylphenidate | Yes. | Stimulant (narcolepsy) |
| Methylprednisolone | No, defer 72 hours, if p.o. or IM; yes, if topical of intra-articular. | Corticosteroid |
| *Methyltestosterone* | *No, if for cancer.* | *Anabolic steroid* |
| Methyprylon | Yes. | Sedative/hypnotic |
| Methysergide | Yes. | Ergot alkaloid |
| Meticortelone | Defer 72 hours if p.o. or IM; yes if topical or intra-articular. | Corticosteroid |
| Meticorten | Defer 72 hours if p.o. or IM: yes, if topical or intra-articular. | Corticosteroid |
| *Metoclopramide* | *Yes, but Evaluate underlying medical history.* | *Gastric stimulant, antiemetic often used for nausea secondary to chemotherapy.* |
| Metolazone | Yes. | Antihypertensive diuretic |
| Metoprolol (Toprol XL) | Yes, if for hypertension. No, if for angina. | Antihypertensive, beta blocking agent |
| Metreton | Defer 72 hours if p.o. or IM: yes if topical or intra-articular. | Corticosteroid |
| Metronidazole | Defer 1 week after course completed and feel well. Yes, if for vaginitis. | Antimicrobial |
| Mevacor | Yes. | Cholesterol lowering agent |
| *Mexiletine* | *No, until off medication and free of symptoms.* | *Antiarrhythmic* |
| *Mexitil* | *No, until off medication and free of symptoms.* | *Antiarrhythmic* |
| Miacalcin | Yes. | Nasal spray (for osteoporosis) |
| Micatin | Yes, if topical. No, defer 1 week after course completed and feel well. | Antifungal |
| Miconazole | Yes, if topical. No, defer 1 week after course completed and feel well. | Antifungal |
| Micrainin | Yes, if taken for allergies. Defer for 72 hours after symptoms are resolved if taken for cold/flu symptoms.  Defer 72 hrs for plateletpheresis or sole source platelets | Analgesic, hypnotic/sedative |
| Micro-K10 | Yes. | Potassium replacement |
| Micronase | Yes. | Oral hypoglycemic |
| Micronefrin | Yes, even if daily dose for maintenance. | Bronchodilator |
| Micronor | Yes. | Oral contraceptive |
| Midamor | Yes. | Antihypertensive, diuretic |
| Midazolam hydrochloride | Yes. | Sedative |
| Midol Caplets (Glenbrook) | Yes, if taken for allergies. Defer for 72 hours after symptoms are resolved if taken for cold/flu symptoms or for fever.  Defer 72 hrs for plateletpheresis or sole source platelets | ASA containing analgesic |
| Midol for Cramps Glenbrook) | Yes, if taken for allergies. Defer for 72 hours after symptoms are resolved if taken for cold/flu symptoms or for fever.  Defer 72 hrs for plateletpheresis or sole source platelets | ASA containing analgesic |
| Midrin | Yes, if taken for allergies. Defer for 72 hours after symptoms are resolved if taken for cold/flu symptoms or for fever.  Defer 72 hrs for plateletpheresis or sole source platelets | Analgesic, ASA (migraine headache) |
| Milk of Magnesia | Yes, if ulcer disease pain-free | Laxative, antacid |
| Milkinol | Yes. | Laxative |
| Milpath | Yes, if ulcer disease pain-free | Antispasmodic/anticholinergic hypnotic/sedative |
| Milprem | Yes. | Hormones, hypnotic/sedative |
| Miltown | Yes. | Hypnotic/sedative |
| *Miltrate* | *No, defer until off medication and symptom free. Evaluate underlying condition.* | *Antianginal* |
| Mineral Oil | Yes. | Laxative |
| Minipress | Yes. | Antihypertensive |
| Minizide | Yes, if for hypertension. No, if for heart disease. | Antihypertensive, diuretic |
| Minocin | Defer 24 hrs. after course Completed and feel well; if IV or IM defer 1 wk. Yes if for acne. | Antibiotic |
| Minocycline | Defer 24 hours after course completed and feel well. Yes, if for acne. | Antibiotic |
| Minoxidil | Yes. | Antihypertensive and topical to promote hair growth |
| Mintezol | Defer 1 week after course completed and feel well. | Antihelminthic |
| Miradon | Defer pending medical evaluation with cessation of drug. | Anticoagulant |
| Miraphen PSE | Yes, if taken for allergies. Defer for 72 hours after symptoms are resolved if taken for cold/flu symptoms. | Decongestant |
| Misoprostol | Yes, if 4 hours since last dose. | Anti-ulcer agent |
| **Mitomycin** | **No permanent deferral.** | **Antineoplastic** |
| **Mitotane** | **No permanent deferral.** | **Antineoplastic** |
| Mitrolan | Yes. | Laxative |
| M-M-R II | Defer 4 weeks (for rubella). | Vaccine: Measles, Mumps, and Rubella Virus Live |
| Moban | Yes, if mentally and legally responsible. | Antipsychotic |
| Mobidin | Yes, if arthritis is inactive. | Antiarthritic |
| Modane | Yes. | Laxative |
| Moderil | Yes, watch for orthostatic pressure changes. | Antihypertensive |
| Modicon | Yes. | Oral contraceptive |
| Moduretic | Yes. | Antihypertensive, diuretic |
| Molindone | Yes, if mentally and legally responsible. | Antipsychotic |
| Mol-Iron | Yes. | Hematinic |
| Momentum (Whitehall) | Yes, if taken for allergies. Defer for 72 hours after symptoms are resolved if taken for cold/flu symptoms or for fever.  Defer 72 hrs for plateletpheresis or sole source platelets | ASA containing analgesic |
| Momentum Muscular Backache | Yes, if taken for allergies. Defer for 72 hours after symptoms are resolved if taken for cold/flu symptoms or for fever.  Defer 72 hrs for plateletpheresis or sole source platelets | ASA containing analgesic |
| Monistat | Yes, if topical. Otherwise, no, defer 1 week after course completed and feel well. | Antifungal |
| Monopril | Yes, if for hypertension. No, if for heart disease | ACE Inhibitor. Antihypertensive |
| Morphine | Yes, if not abuser. | Narcotic, analgesic |
| Motrin | Yes, if arthritis inactive. Defer plateletpheresis donors 24 hours. | Non-steroidal anti-inflammatory |
| Moxalactam | Defer 24 hours after course completed and feel well; if IV or IM defer 1 week. | Antibiotic |
| Mucilose | Yes. | Laxative |
| Mucomyst | Defer until off medication and underlying condition resolved. | Mucolytic agent |
| Mudrane | Yes, even if daily dose for maintenance. | Bronchodilator |
| Multifuge | Defer 1 week after course completed and feel well. | Antihelminthic |
| Mulvidren | Yes. | Vitamins |
| Muse | Yes. | For erectile dysfunction |
| **Mutamycine** | **No, permanent deferral.** | **Antineoplastic** |
| My-Luck Effervescent Tablet (Perrigo) | Yes, if taken for allergies. Defer for 72 hours after symptoms are resolved if taken for cold/flu symptoms or for fever.  Defer 72 hrs for plateletpheresis or sole source platelets | ASA containing analgesic |
| *Myambutol* | *No, defer until off drug and disease free.* | *Antituberculous* |
| Mycelex | Yes. | Topical antifungal |
| Mychel | Defer 24 hours after course completed and feel well; if IV or IM defer 1 week. | Antibiotic |
| **Mycobutin** | **Permanent deferral.** | **Prevents Mycobacterium avium complex in HIV patients** |
| Mycostatin | Yes. | Topical antifungal |
| Mylanta | Yes, if ulcer disease pain-free. | Antacid |
| **Myleran** | **No, permanent deferral.** | **Antineoplastic** |
| Mylicon | Yes. | Antiflatulent |
| Myochrysine | Yes if arthritis is inactive. | Anti-rheumatic |
| Myotonachol | Yes. | Cholinomimetic |
| Mysoline | Yes. | Anticonvulsant |
| Mysteclin-F | Defer 1 week after course completed and feel well. | Antibiotic, antifungal |
| **Mytelase** | **No, permanent deferral.** | **Anticholinesterase** |
| Mytrex | Yes. | Topical antifungal |
| Nabumetone | Yes. No plateletpheresis deferral. (no anti-platelet effect) | Non-steroidal anti-inflammatory |
| Nadolol | No, if for angina (permanent deferral) or arrhythmia (defer while on drug). Otherwise, yes. | Antianginal, Antihypertensive, antiarrhythmic, antimigraine |
| Nafarelin | Yes. | Use in managing endometriosis |
| Nalbuphine | Yes, if not abuser. | Narcotic, analgesic |
| Naldecon | Yes, if taken for allergies. Defer for 72 hours after symptoms are resolved if taken for cold/flu symptoms. | Antihistamine, decongestant |
| Nalfon | Yes, if arthritis inactive. Defer plateletpheresis donors 24 hours. | Anti-inflammatory |
| Nalidixic Acid | Defer 24 hours after course completed and feel well | Antibacterial |
| Naltrexone | Yes, if not abuser. | Narcotic analgesic |
| Nandrolone | No, permanent deferral if renal disease Otherwise, yes. | Anabolic steroid. |
| Naprosyn | Yes, if arthritis inactive. Defer plateletpheresis donors 24 hours. | Non-steroidal anti-inflammatory |
| Naproxen | Yes, if arthritis inactive. Defer plateletpheresis donors 24 hours. | Non-steroidal anti-inflammatory |
| Naqua | Yes. | Antihypertensive, diuretic |
| Naquival | Yes, watch for orthostatic pressure changes. | Antihypertensive |
| Nardil | Defer 72 hours. | Ganglionic blocking agent, antidepressant |
| Nasacort | Yes. | Nasal spray for allergic rhinitis. |
| Nasalide | Yes. | Topical steroid |
| Natabec | Yes. | Vitamins |
| Natacyn | Yes. | Ophthalmic antifungal |
| Natalins | Yes. | Vitamins |
| Natamycin | Yes. | Ophthalmic antifungal |
| Naturetin | Yes. | Antihypertensive, diuretic |
| Navane | Yes, if mentally and legally responsible. | Antipsychotic, tranquilizer |
| NegGram | Defer 24 hours after course completed and feel well; if IV or IM defer 1 week. | Antibacterial |
| Nembutal | Yes, if not abuser. | Sedative |
| Neocholan | Yes. | Bile salt |
| Neogesic (Rev) (Vale) | Yes, if taken for allergies. Defer for 72 hours after symptoms are resolved if taken for cold/flu symptoms or for fever.  Defer 72 hrs for plateletpheresis or sole source platelets | ASA containing analgesic |
| Neoloid | Yes. | Laxative |
| **Neostigmine** | **No, permanent deferral.** | **Anticholinesterase (for myasthenia gravis)** |
| Neo-Synephrine | Yes, even if daily dose for maintenance. | Vasoconstrictor, bronchodilator |
| Neothylline | Yes, even if daily dose for maintenance. | Bronchodilator |
| Neotrizine | Defer 24 hours after course completed and feel well. | Antibiotic |
| Neptazane | Yes. | Antiglaucoma |
| Neurontin | Yes. | Anticonvulsant |
| Neutra-Phos | Yes. | Laxative |
| Neutropin | Yes. | Recombinant Growth Hormone |
| Niacin, Niacinamide, Nialex | Yes, watch for orthostatic pressure changes. | Vasodilator, vitamin, antihyperlipidemic |
| Niamid | Defer 72 hours after course completed. | Ganglionic blocking agent, antidepressant |
| Nicardipine | Yes if for hypertension. No if for heart disease. | Antianginal, anti-hypertensive |
| Nicobid, Nico-400, Nicolar | Yes, watch for orthostatic pressure changes. | Vasodilator, vitamin, antihyperlipidemic |
| Nicoderm | Yes. | Transdermal nicotine. Aid to smoking cessation. |
| Niconyl | Yes, if for prophylaxis. Otherwise, defer until off drug and well. | Antituberculous |
| Nicotinamide | Yes, watch for orthostatic pressure changes. | Vasodilator, vitamin, antihyperlipidemic |
| Nicotinic Acid | Yes, watch for orthostatic pressure changes. | Vasodilator, vitamin, antihyperlipidemic |
| Nicotinyl Alcohol | Yes. | Vasodilator (weak) |
| Nifedipine | Yes, if for esophageal achalasia, migraine or hypertension; otherwise, no. | Antianginal, calcium channel blocker |
| Niferex | Yes. | Hematinic |
| Night-Time Effervescent Tab (Zenith Goldline) | Yes, if taken for allergies. Defer for 72 hours after symptoms are resolved if taken for cold/flu symptoms or for fever.  Defer 72 hrs for plateletpheresis or sole source platelets | ASA containing analgesic |
| Nilstat | Yes. | Topical antifungal |
| Nimotop | Yes, for drug. No if has subarachnoid hemmorrage. | Calcium Channel Blocker |
| Niridazole | No, defer 1 week after course completed and feel well. | Antihelminthic |
| Nisentil | Yes, if not abuser. | Narcotic, analgesic |
| Nitrex | No, defer 24 hours after course completed and feel well. | Antibiotic |
| Nitro-Bid | No, defer until off medication and free of symptoms. | Antianginal |
| Nitrocap, Nitrodisc | No, defer until off medication and free of symptoms. | Antianginal |
| Nitrofor | Defer 24 hours after course completed and feel well. | Antibiotic |
| Nitrofurantoin | Defer 24 hours after course completed and feel well. | Antibiotic |
| Nitrofurazone | Yes. | Topical antibiotic |
| Nitroglycerin, Nitroglyn, Nitrol, Nitrong, Nitrospan, Nitrostat | No, defer until off medication and free of symptoms. | Antianginal |
| Nizatidine | Yes, if ulcer disease inactive. | Anti-ulcer agent |
| Nizoral | Yes, if for fungal infections of skin or nail beds. No, if for systemic (generalized) infection. | Antifungal |
| No Doz | Yes. | Stimulant |
| Noctec | Yes. | Hypnotic/sedative |
| Nodaline | Yes, if not abuser.  Yes, if taken for allergies. Defer for 72 hours after symptoms are resolved if taken for cold/flu symptoms. | Analgesic |
| Nolamine | Yes, if taken for allergies. Defer for 72 hours after symptoms are resolved if taken for cold/flu symptoms. | Antihistamine, decongestant |
| Nolex | Yes, if taken for allergies. Defer for 72 hours after symptoms are resolved if taken for cold/flu symptoms. | Decongestant, expectorant. |
| Noludar | Yes. | Hypnotic/sedative |
| *Nolvadex* | *Yes, if used to treat non-malignant disease. No, permanent deferral.* | *Antineoplastic* |
| Nor Q.D. | Yes. | Oral contraceptive |
| Norethindrone | Yes. | Oral contraceptive |
| Norflex | Yes, if taken for allergies. Defer for 72 hours after symptoms are resolved if taken for cold/flu symptoms. | Analgesic |
| Norfloxacin | Defer until 24 hours after course completed and feel well. | Antibiotic |
| Norgesic | Yes, if taken for allergies. Defer for 72 hours after symptoms are resolved if taken for cold/flu symptoms or for fever.  Defer 72 hrs for plateletpheresis or sole source platelets | Analgesic, antipyretic, ASA. |
| Norgesic Forte | Yes, if taken for allergies. Defer for 72 hours after symptoms are resolved if taken for cold/flu symptoms or for fever.  Defer 72 hrs for plateletpheresis or sole source platelets | ASA containing analgesic |
| Norinyl | Yes. | Oral Contraceptive |
| Norisodrine | Yes, even if daily dose for maintenance. | Bronchodilator |
| Norlestrin | Yes. | Oral contraceptive |
| Norlutate | Yes. | Oral contraceptive |
| Normodyne | Yes. | Antihypertensive |
| Noroxin | Defer until 24 hours after course completed and feel well. | Antibiotic |
| Noroxine | Yes. | Thyroid replacement |
| *Norpace* | *No, defer until off drug and free of symptoms.* | *Antiarrhythmic* |
| Norplant | Yes, do not apply BP cuff or tourniquet to arm used for implant device. | Contraceptive |
| Norpramin | Yes. | Tricyclic antidepressant |
| Nortriptyline | Yes. | Tricyclic antidepressant |
| Norvasc | Yes, if for hypertension. No, if for angina. | Calcium channel blocker, antihypertensive, anti-anginal. |
| Norwich | Yes, if taken for allergies. Defer for 72 hours after symptoms are resolved if taken for cold/flu symptoms or for fever.  Defer 72 hrs for plateletpheresis or sole source platelets | ASA containing analgesic |
| Norwich Extra-Strength | Yes, if taken for allergies. Defer for 72 hours after symptoms are resolved if taken for cold/flu symptoms or for fever.  Defer 72 hrs for plateletpheresis or sole source platelets | ASA containing analgesic |
| Novacaine | Yes, if taken for allergies. Defer for 72 hours after symptoms are resolved if taken for cold/flu symptoms. | Analgesic |
| Novafed | Yes, if taken for allergies. Defer for 72 hours after symptoms are resolved if taken for cold/flu symptoms. | Decongestant, antihistamine |
| Novahistine | Yes, if taken for allergies. Defer for 72 hours after symptoms are resolved if taken for cold/flu symptoms. | Antihistamine |
| Novatrin | Yes, if ulcer disease pain-free. | Anticholinergic/antispasmodic |
| Novolin Insulin | Yes, if dosage controlled and stable | Hypoglycemic |
| Novrad | Yes, if taken for allergies. Defer for 72 hours after symptoms are resolved if taken for cold/flu symptoms or for fever.  Defer 72 hrs for plateletpheresis or sole source platelets | ASA containing analgesic |
| NPH Iletin, NPH Insulin | Yes, if dosage controlled and stable. | Hypoglycemic |
| Nubain | Yes, if not abuser. | Narcotic, analgesic |
| Nuprin | Yes, if arthritis inactive. Defer plateletpheresis donors 24 hours. | Anti-inflammatory, analgesic |
| *Nydrazid* | *No, defer until course of medication completed and disease inactive.* | *Antituberculous* |
| Nylidrin | Yes, watch for orthostatic pressure changes. | Vasodilator |
| Nystaform | Yes. | Topical antifungal |
| Nystatin | Yes. | Topical antifungal |
| Obedrin-LA | Yes. | Amphetamine |
| Obetrol | Yes. | Anorexiant |
| Obotan | Yes. | Anorexiant |
| Ocuvite | Yes. | Zinc with vitamins & minerals |
| Ofloxacin | Defer 24 hrs after completion and feels well. | Antibiotic |
| Ogen | Yes. | Hormone |
| **Olsalazine** | **No.** | **Anti-inflammatory for ulcerative colitis** |
| Omeprazole | Yes, if ulcer disease pain-free. | Anti-ulcer agent |
| OmniHIB | Accept immediately if symptom-free. | Vaccine: Sterile conjugate of Haemophilus B and diphtheria protein |
| Omnipen | Defer 24 hours after course completed and feel well; if IV or IM defer 1 week. | Antibiotic |
| Optimine | Yes, if taken for allergies. Defer for 72 hours after symptoms are resolved if taken for cold/flu symptoms. | Antihistamine |
| Orap | Yes. | Antidepressant |
| Orasone | Defer 72 hours if P.O. or IM; yes, if topical or intra-articular. | Corticosteroid |
| Oratrol | Yes. | Antiglaucoma |
| Oretic | Yes. | Diuretic |
| Oreticyl | Yes. | Antihypertensive, diuretic |
| Oreton | No, if for cancer; otherwise yes. | Androgen |
| Organden | Yes, if taken for allergies. Defer for 72 hours after symptoms are resolved if taken for cold/flu symptoms. | Expectorant |
| Orinase | Yes. | Oral hypoglycemic |
| Ornade | Yes, if ulcer disease pain-free. | Anticholinergic, antispasmodic |
| Orphenadrine | Yes. | Muscle relaxant |
| Orphenadrine Citrate with Asprin | Yes, if taken for allergies. Defer for 72 hours after symptoms are resolved if taken for cold/flu symptoms or for fever.  Defer 72 hrs for plateletpheresis or sole source platelets | ASA containing analgesic |
| Orprine | Yes. | Sedative, anticonvulsant |
| Ortho-Novum | Yes. | Oral contraceptive |
| Orudis | Yes, if arthritis inactive. Defer plateletpheresis donors 24 hours. | Non-steroidal anti-inflammatory agent |
| Oruvail | Yes, if arthritis inactive. Defer plateletpheresis donors 24 hours. | Non-steroidal anti-inflammatory agent |
| Os-Cal Mone | Yes. | Vitamin, hormone |
| Ostone | No, defer 72 hours if P.O. or IM; yes, if topical or intra-articular. | Corticosteroid |
| O-V Statin | Yes. | Topical antifungal |
| Ovcon | Yes. | Oral contraceptive |
| Ovral | Yes. | Oral contraceptive |
| Ovrette | Yes. | Oral contraceptive |
| Ovulen | Yes. | Oral contraceptive |
| Oxabid | Yes; if ulcer disease pain-free. | Antacid |
| Oxacillin | No, defer 24 hours after course completed and feel well; if IV or IM defer 1 wk. | Antibiotic |
| Oxalid | Yes, if arthritis inactive. | Anti-inflammatory |
| Oxaminquine | No, defer 1 week after course completed and feel well. | Antihelminthic |
| Oxandrolone | Yes. | Anabolic steroid |
| Oxaprozin | Yes, if arthritis inactive. Defer plateletpheresis donors 24 hours. | Non steroidal anti-inflammatory |
| Oxazapam | Yes. | Tranquilizer, muscle relaxant |
| Oxazolid | Yes, if arthritis inactive. | Anti-inflammatory |
| Oxolinic Acid | Defer 24 hours after course completed and feel well. | Antibiotic |
| Oxsoralen | Yes. | Repigmentation drug |
| Oxtriphylline | Yes, even if daily dose for maintenance. | Bronchodilator |
| Oxybutynin | Yes. | Anticholinergic/antispasmodic |
| Oxymetazoline | Yes, if taken for allergies. Defer for 72 hours after symptoms are resolved if taken for cold/flu symptoms. | Decongestant |
| Oxymetholone | No, if renal disease; otherwise yes. | Anabolic Steroid |
| Oxyphenbutazone | Yes, if arthritis inactive. | Anti-inflammatory |
| Oxyphencyclimine | Yes, if ulcer disease pain-free. | Anticholinergic, antispasmodic |
| Oxytetraclor, Oxytetracycline | Defer 24 hours after course completed and feel well; if IV or IM defer 1 week. Yes, if for acne. | Antibiotic |
| P-200 | Yes. | Vasodilator |
| Pabalate | Yes, if taken for allergies. Defer for 72 hours after symptoms are resolved if taken for cold/flu symptoms. | Analgesic |
| P-A-C | Yes, if taken for allergies. Defer for 72 hours after symptoms are resolved if taken for cold/flu symptoms or for fever.  Defer 72 hrs for plateletpheresis or sole source platelets | Analgesic, ASA |
| Pagitane | Yes. | Anti-Parkinsonism |
| Pain-Aid (Zee) | Yes, if taken for allergies. Defer for 72 hours after symptoms are resolved if taken for cold/flu symptoms or for fever.  Defer 72 hrs for plateletpheresis or sole source platelets | ASA containing analgesic |
| Pain Reliever Tablets (Rugby) | Yes, if taken for allergies. Defer for 72 hours after symptoms are resolved if taken for cold/flu symptoms or for fever.  Defer 72 hrs for plateletpheresis or sole source platelets | ASA containing analgesic |
| Pamelor | Yes. | Tricyclic antidepressant |
| Pamine | Yes, if ulcer disease pain-free. | Anticholinergic, antispasmodic |
| Panadol | Yes, if taken for allergies. Defer for 72 hours after symptoms are resolved if taken for cold/flu symptoms. | Antipyretic, analgesic |
| Pancrease | Yes. | Enzyme replacement |
| Pancreatin | Yes. | Enzyme replacement |
| Pancrelipase | Yes. | Enzyme replacement |
| *Panheparin* | *Defer pending medical evaluation with cessation of drug.* | *Anticoagulant* |
| Panmycin | Defer 24 hours after course completed and feel well; if IV or IM defer 1 week; yes if for acne. | Antibiotic |
| Panodynes Analgesic (Keystone) | Yes, if taken for allergies. Defer for 72 hours after symptoms are resolved if taken for cold/flu symptoms or for fever.  Defer 72 hrs for plateletpheresis or sole source platelets | ASA containing analgesic |
| Panteric | Yes. | Enzyme replacement |
| *Panwarfarin* | *Defer pending medical evaluation with cessation of drug.* | *Anticoagulant* |
| Papaverine | Yes; unless used for arrhythmia. | Vasodilator |
| Paracort | Defer 72 hours if P.O. or IM; yes, if topical or intra-articular. | Corticosteroid |
| Paradione | Yes. | Anticonvulsant |
| Paraflex | Yes. | Muscle relaxant |
| Parafon | Yes. | Muscle relaxant |
| Parafuran | Defer 24 hours after course completed and feel well; if IV or IM defer 1 week. | Antibiotic |
| Paraglycine | Defer 72 hours. | Antihypertensive (MAO inhibitor) |
| Paral, Paraldehyde | Yes. | Anticonvulsant |
| Paramethasone | Defer 72 hours if p.o. or IM; yes, if topical or intra-articular. | Corticosteroid |
| Parasal | Yes, if taken for allergies. Defer for 72 hours after symptoms are resolved if taken for cold/flu symptoms or for fever. | Analgesic, antipyretic anti-inflammatory |
| Parda | Yes. | Anti-Parkinsonism |
| Paregoric | Yes, if symptom free for 3 days. | Antidiarrheal |
| Parepectolin | Yes, if symptom free. | Antidiarrheal |
| Parest | Yes. | Hypnotic/sedative |
| Pargel | Yes, if symptom free. | Antidiarrheal |
| Parlodel | Yes, if for Parkinson’s. No, if for Pituitary tumors. | Anti-Parkinsonism. Dopamine agonist |
| Parnate | Defer 72 hours. | Antidepressant (MAO inhibitor) |
| Paromomycin | Defer 1 week after course completed and feel well. | Amebicide |
| Parsidol | Yes. | Anti-Parkinsonism |
| *PAS* | *Defer until course of medication completed and disease inactive.* | *Antituberculous* |
| Pathibamate | Yes, if ulcer disease pain-free. | Anticholinergic/antispasmodic, hypnotic/sedative |
| Pathilon | Yes, if ulcer disease pain-free. | Anticholinergic/antispasmodic |
| Pathocil | Defer 24 hours after course completed and feel well. | Antibiotic |
| Pavabid, Pavacap, Paverine | Yes; no, if for arrhythmia. | Vasodilator |
| Pax-400 | Yes. | Hypnotic/sedative |
| Paxil | Yes. | Antidepressant |
| PBZ | Yes, if taken for allergies. Defer for 72 hours after symptoms are resolved if taken for cold/flu symptoms. | Antihistamine |
| Pc-Cap (Alra) | Yes, if taken for allergies. Defer for 72 hours after symptoms are resolved if taken for cold/flu symptoms or for fever.  Defer 72 hrs for plateletpheresis or sole source platelets | ASA containing analgesic |
| Pediamycin | Defer 24 hours after course completed and feel well; if IV or IM defer 1 week. Yes, if for acne. | Antibiotic |
| Pediazole | Defer 24 hrs. after course completed and feel well; if IV or IM defer 1 wk. Yes, if for acne. | Antibiotic |
| Pemoline | Yes. | Stimulant |
| Penapar VK | Defer 24 hours after course completed and feel well; if IV or IM defer 1 week. | Antibiotic |
| Penbritin | Defer 24 hours after course completed and feel well; if IV or IM defer 1 week. | Antibiotic |
| Penicillamine | Yes, if arthritis is inactive. No, if collagen vascular disease or other chronic disease. | Anti-inflammatory |
| Penicillin | Defer 24 hours after course completed and feel well; if IV or IM defer 1 week. If for STD, defer 12 months. Yes, for acne. | Antibiotic |
| Penntuss | Yes. | Anti-tussive |
| Pensyn | Defer 24 hours after course completed and feel well; if IV or IM defer 1 week. | Antibiotic |
| Pentacef | Defer 1 week. | Antibiotic |
| *Pentaerythritol Tetranitrate* | *No, defer until off medication and free of symptoms. Evaluate underlying condition.* |  |
| Pentazocine | Yes, if not abuser. | Narcotic, analgesic |
| Pentids | Defer 24 hours after course completed and feel well; if IV or IM defer 1 week. If for STD, defer 12 months. Yes, for acne. | Antibiotic |
| Pentobarbital | Yes. | Sedative/hypnotic |
| Pentoxifylline | Yes. | Anti-claudication |
| *Pentritol* | *No, defer until off medication and symptom free. Evaluate underlying condition.* | *Antianginal* |
| PenVeeK | Defer 24 hours after course completed and feel well; if IV or IM defer 1 week. If for STD, defer 12 months. Yes, for acne. | Antibiotic |
| Pepcid | Yes, if not active ulcer disease | Anti-ulcer medication |
| Pepidyne (Teva) | Yes, if taken for allergies. Defer for 72 hours after symptoms are resolved if taken for cold/flu symptoms or for fever.  Defer 72 hrs for plateletpheresis or sole source platelets | ASA containing analgesic |
| Pepto-Bismol | Yes, if symptom free for 3 days. | Antidiarrheal |
| Percocet | Yes, if not abuser. | Narcotic analgesic |
| Percodan | Yes, if not abuser. Defer 72 hrs for plateletpheresis or sole source platelets | Narcotic analgesic, ASA |
| Percodan-Demi | Yes, if taken for allergies. Defer for 72 hours after symptoms are resolved if taken for cold/flu symptoms or for fever.  Defer 72 hrs for plateletpheresis or sole source platelets | ASA containing analgesic |
| Percogesic | Yes, if taken for allergies. Defer for 72 hours after symptoms are resolved if taken for cold/flu symptoms. | Analgesic |
| Percorten | Defer 72 hours if P.O. or IV; yes, if topical or intra-articular. | Corticosteroid |
| Perdiem | Yes. | Laxative |
| Pergonal | Yes. | Fertility drug. |
| Periactin | Yes, if taken for allergies. Defer for 72 hours after symptoms are resolved if taken for cold/flu symptoms. | Antihistamine |
| Peri-Colace | Yes. | Laxative |
| *Peritrate* | *No, defer until off medication and symptom free.* | *Antianginal* |
| Permitil | Yes, if mentally and legally responsible. | Antipsychotic |
| Perphenazine | Yes, if mentally and legally responsible. | Antipsychotic |
| Persantine | No, permanent deferral if angina or heart disease. Yes if used as anti-platelet and underlying condition is not cause for deferral. No platelet collections. | Antianginal, antiplatelet |
| Persistin | Yes, if taken for allergies. Defer for 72 hours after symptoms are resolved if taken for cold/flu symptoms. | Analgesic, anti-inflammatory |
| Pertofrane | Yes. | Tricyclic antidepressant |
| Pertussis Immune Globulin | Yes, if feeling well | Anti-pertussis |
| Petrogalar | Yes. | Laxative |
| Pfizer-E | Defer 24 hours after course completed and feel well; if IV or IM defer 1 week. Yes if for acne. | Antibiotic |
| Pfizerpen VK | Defer 24 hours after course completed and feel well; if IV or IM defer 1 week. If for STD, defer 12 months. Yes, for acne. | Antibiotic |
| Pharmalgen | Yes. | Desensitization biological |
| Phazyme | Yes. | Antigas |
| Phelantin | Yes. | Anticonvulsant |
| Phenaphen | Yes, if taken for allergies. Defer for 72 hours after symptoms are resolved if taken for cold/flu symptoms or for fever.  Defer 72 hrs for plateletpheresis or sole source platelets | Analgesic, ASA |
| Phenazodine | Defer until infection cleared. Yes, if not has active infection. | Urinary analgesic |
| Phenazopyridine | Defer until infection cleared. Yes, if no active infection. | Urinary analgesic |
| Phendimetrazine | Yes. | Anorexiant |
| Phenelzine | Yes. | Antidepressant |
| Phenergan | Yes, as antiemetic. Yes, if taken for allergies. Defer for 72 hours after symptoms are resolved if taken for cold/flu symptoms or for fever. | Antihistamine, antiemetic |
| *Phenindione* | *Defer pending medical evaluation with cessation of drug.* | *Anticoagulant* |
| Phenmetrazine | Yes. | Anorexiant |
| Phenobarbital | Yes. | Anticonvulsant, tranquilizer |
| Phenolphthalein | Yes. | Laxative |
| Phenoxene | Yes. | Anti-Parkinsonism |
| *Phenoxybenzamine* | *No, permanent deferral if for pheochromocytoma. Evaluate underlying condition.* | *Antihypertensive, vasodilator (may cause low pressure or tachycardia on standing)* |
| *Phenprocoumon* | *Defer pending medical evaluation with cessation of drug.* | *Anticoagulant* |
| Phentermine | Yes. | Anorexiant |
| **Phentolamine** | **No, permanent deferral if for pheochromocytoma.** | **Antihypertensive** |
| Phentrol | Yes. | Anorexiant |
| Phenylbutazone | Yes, if arthritis inactive. | Non-steroidal anti-inflammatory |
| Phenylephrine | Yes, if taken for allergies. Defer for 72 hours after symptoms are resolved if taken for cold/flu symptoms. | Decongestant |
| Phenylpropanolamine | Yes, as anorexiant. Yes, if taken for allergies. Defer for 72 hours after symptoms are resolved if taken for cold/flu symptoms. | Anorexiant, decongestant |
| Phenytoin | Yes, as anticonvulsant. Yes, if taken for allergies. Defer for 72 hours after symptoms are resolved if taken for cold/flu symptoms. | Anticonvulsant, analgesic |
| Phosphates | Yes. | Mineral/laxative |
| Phospho-Soda | Yes. | Laxative |
| Phthalysulfa-thiazole | Defer 24 hours after course completed and feel well; if IV or IM defer 1 week. | Antibiotic |
| *Phytonadione* | *Evaluate underlying condition.* | *Vitamin K* |
| Pilocarpine | Yes. | Anti-glaucoma agent |
| Pinolol | Defer while on drug if for arrhythmia; yes if for hypertension. | Antiarrhythmic, antihypertensive |
| Piperacetazine | Yes, if mentally and legally responsible. | Antipsychotic |
| Piperazine | Defer 1 week after course completed and feel well. | Antihelminthic |
| Piroxicam | Yes, if arthritis inactive. Defer plateletpheresis donors for 3 days. | Non-steroidal anti-inflammatory |
| *Pitressin* | *No, permanent deferral if taken on regular basis and not as emergency.* | *Antidiuretic hormone* |
| Placidyl | Yes. | Hypnotic |
| Plantago Seed | Yes. | Laxative |
| Plaquenil | No, if for malaria and in malaria zone defer 1 year; if not in malaria zone or given for other indication then yes. | Anti-arthritic, anti-inflammatory. Antimalarial |
| Plavix (clopidrogel) | Acceptable for whole blood, red blood cells, FFP, and/or cryoprecipitate; for platelets defer for 5 days after last dose | Anti-platelet |
| Plegine | Yes. | Anorexiant |
| Plendil | Yes. | Calcium channel blocker. Antihypertensive. |
| Polaramine | Yes, if taken for allergies. Defer for 72 hours after symptoms are resolved if taken for cold/flu symptoms. | Antihistamine |
| Poloxamer 188 | Yes. | Laxative |
| Polycarbophil | Yes. | Laxative |
| Polycillin | Defer 24 hours after course completed and feel well; if IV of IM defer 1 week. | Antibiotic |
| Polymagma | Yes, if symptom free. | Antidiarrheal |
| Polymox | Defer 24 hours after course completed and feel well; if IV or IM defer 1 week. | Antibiotic |
| Polythiazide | Yes. | Antihypertensive, diuretic |
| Poly-Vi-Flor, Poly-Vi-Sol | Yes. | Vitamin |
| Pondimin | Yes. | Anorexiant |
| Ponstel | Yes, if arthritis inactive.  Yes, if taken for allergies. Defer for 72 hours after symptoms are resolved if taken for cold/flu symptoms. | Anti-inflammatory. Analgesic, antipyretic |
| Potaba | Yes. | For systemic anti-fibrosis |
| Potassium Bicarbonate, Citrate, Chloride, Gluconate | Yes. | Potassium replacement |
| Potassium Iodide | Yes for thyroid. Yes, if taken for allergies. Defer for 72 hours after symptoms are resolved if taken for cold/flu symptoms. | Expectorant, thyroid agent |
| Povan | Defer 1 week after course completed and feel well | Antihelminthic |
| Prantal | Yes, if ulcer disease pain-free. | Anticholinergic, antispasmodic |
| Pravachol | Yes. | Cholesterol lowering agent. |
| Prazepam | Yes. | Sedative, tranquilizer |
| Prazosin | No, if for heart disease. Yes, if for hypertension. | Antihypertensive |
| Precomp (Zee) | Yes, if taken for allergies. Defer for 72 hours after symptoms are resolved if taken for cold/flu symptoms or for fever.  Defer 72 hrs for plateletpheresis or sole source platelets | ASA containing analgesic |
| Precose X | Yes. | Anti-hyperglycemic |
| Prednis, Prednisal, Prednisolone, Prednisone, Predulose | Defer 72 hours if P.O. or IM; yes, if topical or intra-articular. | Corticosteroid |
| Pregnyl | Yes. | Gonadotropin |
| Preludin | Yes. | Stimulant |
| Premarin | Yes. | Hormone |
| Presalin (Roberts) | Yes, if taken for allergies. Defer for 72 hours after symptoms are resolved if taken for cold/flu symptoms or for fever.  Defer 72 hrs for plateletpheresis or sole source platelets | ASA containing analgesic |
| Presamine | Yes. | Tricyclic Antidepressant |
| Pre-Sate | Yes. | Anorexiant |
| Prevacid | Yes, if ulcer disease pain free | Anti-ulcer agent |
| Prilosec | Yes, if ulcer disease is pain free. | Anti-ulcer agent |
| Primaquine | No, if malarial zone, defer 1 year; if not in malarial zone, yes. | Antimalarial |
| Primatene | Yes, even if daily dose for maintenance. | Bronchodilator |
| Primidone | Yes. | Anticonvulsant |
| Principen | Defer 24 hours after course completed and feel well; if IV or IM defer 1 week. | Antibiotic |
| Prinivil | Yes, if for hypertension. | Antihypertensive |
| Prinzide | Yes, if for hypertension. | Antihypertensive |
| **Priscoline** | **No.** | **Vasodilator** |
| Probanthine | Yes, if ulcer disease pain-free. | Anticholinergic, antispasmodic |
| Probenecid | Yes. | Uricosuric agent |
| Probucol | Yes. | Antihyperlipidemic |
| *Procainamide* | *No, until off drug and symptom free.* | *Antiarrhythmic* |
| *Procan* | *No, until off drug and symptom free.* | *Antiarrhythmic* |
| **Procarbazine** | **No, permanent deferral.** | **Antineoplastic** |
| Procardia | No, if for angina. Yes if for esophageal achalasia, migraine or hypertension. | Vasodilator |
| Procholorperazine | Yes, if mentally and legally responsible. | Antipsychotic, antiemetic |
| Procyclidine | Yes. | Anticholinergic, anti-Parkinsonism |
| **Profilate** | **No, permanent deferral.** | **Blood factor product** |
| Progesic | Yes, if taken for allergies. Defer for 72 hours after symptoms are resolved if taken for cold/flu symptoms. | Analgesic |
| Progestasert, Progesterone | Yes. | Hormone |
| Proglycem | Yes, if blood glucose stable. | Hyperglycemic |
| Progynon | Yes. | Hormone |
| Proketazine | Yes, if mentally and legally responsible. | Antipsychotic |
| Prolixin | Yes, if mentally and legally responsible. | Antipsychotic |
| Proloid | Yes. | Thyroid replacement |
| Proloprim | Defer 24 hours after course completed and feel well; if IV or IM defer 1 week. | Antibiotic |
| Promachlor, Promapar | Yes, if mentally and legally responsible. | Antipsychotic |
| Promethazine | Yes, as anti-nauseant . Yes, if taken for allergies. Defer for 72 hours after symptoms are resolved if taken for cold/flu symptoms. | Anti-nauseant, antihistamine |
| Pronemia | Yes. | Hematinic |
| Pronestyl | No, until off drug and symptom free. | Antiarrhythmic |
| Propacil | Yes. | Antithyroid agent |
| Propadrine | Yes, if taken for allergies. Defer for 72 hours after symptoms are resolved if taken for cold/flu symptoms. | Decongestant |
| Propantheline | Yes, if ulcer disease pain-free. | Anticholinergic/antispasmodic |
| Propecia | *Defer for 1 month after stop taking the drug.* | Used for hair loss (same ingredient as Proscar: finasteride) |
| **Proplex** | **No, permanent deferral.** | **Factor IX complex** |
| Propox 65, Propoxyphene | Yes, if taken for allergies. Defer for 72 hours after symptoms are resolved if taken for cold/flu symptoms or for fever.  Defer 72 hrs for plateletpheresis or sole source platelets | Analgesic, ASA |
| Propoxyphene Compound (various) | Yes, if taken for allergies. Defer for 72 hours after symptoms are resolved if taken for cold/flu symptoms or for fever.  Defer 72 hrs for plateletpheresis or sole source platelets | ASA containing analgesic |
| Propranolol | No, if for arrhythmia (defer while on drug), angina (permanent deferral); Yes if for hypertension, migraine. | Antianginal, antiarrhythmic |
| Proprion Gel | Yes. | Topical antifungal |
| Propulsid | Yes. | Gastric stimulant, antiemetic |
| Propylhexedrine | Yes. | Beta 2 stimulator |
| Propylthiouracil | Yes. | Antithyroid agent |
| Proscar (finasteride) | Defer for 1 month after stop taking the drug. | Used for benign prostatic hypertrophy |
| Prostaphilin | Defer 24 hours after course completed and feel well; if IV or IM defer 1 week. | Antibiotic |
| **Prostigmin** | **No, permanent deferral.** | **Anticholinesterase (for myasthenia gravis)** |
| ProstinVR (alprostadil) | Yes | Treatment for erectile dysfunction |
| Protriptyline | Yes. | Tricyclic antidepressant |
| Protropin | Yes. | Growth hormone (recombinant) |
| Proventil | Yes, even if daily dose for maintenance. | Bronchodilator |
| Provera | Yes. | Hormone |
| Prozac | Yes. | Antidepressant |
| Prydon | Yes, if ulcer disease pain-free. | Anticholinergic/antispasmodic |
| Pseudoephedrine | Yes, if taken for allergies. Defer for 72 hours after symptoms are resolved if taken for cold/flu symptoms. | Decongestant |
| Pseudomal | Yes, if taken for allergies. Defer for 72 hours after symptoms are resolved if taken for cold/flu symptoms. | Antihistamine |
| Psyllium | Yes. | Laxative |
| PTU, Propylthiouracil | Yes. | For hyperthyroidism |
| **Purinethol** | **No. Permanent deferral.** | **Antineoplastic** |
| **Purodigin** | **No, permanent deferral.** | **Cardiac Glycoside** |
| Pyrantel Pamoate | Defer 1 week after course completed and feel well. | Antihelminthic |
| *Pyrazinamide* | *No, defer until disease inactive.* | *Antituberculous* |
| Pyrethrin | Defer 48 hours after course completed. | Pediculicide |
| Pyribenzamine | Yes, if taken for allergies. Defer for 72 hours after symptoms are resolved if taken for cold/flu symptoms. | Antihistamine |
| Pyridium | No, if infection not cleared. Yes, if no infection. | Urinary analgesic |
| **Pyridostigmine** | **No, permanent deferral if for myasthenia gravis** | **Anticholinesterase (for myasthenia gravis). Pyridostigmine is also used to counter chemical warfare agents; may donate if otherwise acceptable.** |
| Pyridoxine | Yes. | Vitamin |
| Pyrilamine | Yes, if taken for allergies. Defer for 72 hours after symptoms are resolved if taken for cold/flu symptoms. | Antihistamine |
| Pyrimethamine | No, defer 1 year if in malaria zone, otherwise yes. | Antimalarial |
| Pyristan | Yes, if taken for allergies. Defer for 72 hours after symptoms are resolved if taken for cold/flu symptoms. | Antihistamine |
| Pyrroxate | Yes, if taken for allergies. Defer for 72 hours after symptoms are resolved if taken for cold/flu symptoms. | Antihistamine, analgesic |
| Pyrvinium | Defer 1 week after course completed and feel well. | Antihelminthic |
| Quaalude | Yes, if not abuser. | Hypnotic/sedative |
| Quadrinal | Yes, even if daily dose for maintenance. | Bronchodilator |
| Questran | Yes. | Antihyperlipidemic |
| Quibron | Yes, even if daily dose for maintenance. | Bronchodilator |
| Quide | Yes, if mentally and legally responsible. | Antipsychotic |
| Quinacrine | No, defer 1 year if in malarial zone. No, defer 1 week after course completed if for giardia infection. Yes, if not in malaria zone or other indication. | Antimalarial, giardiacide |
| *Quinaglute* | *No, permanent deferral if for heart disease, defer while on drug if for arrhythmia.* | *Antiarrhythmic* |
| Quinapril | Yes, for stable hypertension | Ace Inhibitor |
| Quinethazone | Yes. | Antihypertensive, diuretic |
| *Quinidex* | *No, permanent deferral if for heart disease, defer while on drug if for arrhythmia.* | *Antiarrhythmic* |
| *Quinidine* | *No, permanent deferral if for heart disease, defer while on drug if for arrhythmia.* | *Antiarrhythmic* |
| Quinine | Yes, unless travel in a malarial zone. See specific country for deferral period. | Treatment for leg cramps. Antimalarial. |
| Rabeprazole (Aciphex) | Yes, if no evidence of bleeding. | Prevention of stomach ulcer bleeding |
| Rabies Immune Globulin | No, defer 12 months. | Biological |
| *Radioactive Iodine* | *No, if for diagnostic use; defer 72 hours or after results known whichever is first. No, defer 3 months if therapeutic use.* | *Antithyroid* |
| Rampiril | Yes, if for hypertension. No, if for heart disease. | ACE Inhibitor, antihypertensive |
| Ranitidine | Yes, if ulcer disease pain-free. | Antacid |
| Raudixin | Yes. | Antihypertensive |
| Rau-Sed | Yes, watch for orthostatic pressure changes. | Antihypertensive |
| Rauwiloid | Yes, watch for orthostatic pressure changes. | Antihypertensive |
| Rauwolfia Alkaloids | Yes, watch for orthostatic pressure changes. | Antihypertensive |
| Rauzide | Yes. | Antihypertensive |
| Redux | Yes. | Appetite suppressant |
| **Regitine** | **No, permanent deferral if for pheochromocytoma.** | **Antihypertensive** |
| Reglan | Yes, evaluate medical history. | Antiemetic, gastric stimulant |
| **Regonal** | **No, permanent deferral** | **Anticholinesterase (for myasthenia gravis)** |
| Regroton | Yes, watch for orthostatic pressure changes. | Antihypertensive |
| Rela | Yes. | Sedative/hypnotic |
| Relafen | Yes. No need for plateletpheresis deferral | Non-steroidal anti-inflammatory (no anti-platelet effect) |
| Remeron | Yes. | Antidepressant |
| Remsed | Yes, as anti-nausenat. Yes, if taken for allergies. Defer for 72 hours after symptoms are resolved if taken for cold/flu symptoms. | Antinauseant, antihistamine |
| Renese | Yes, if for hypertension | Antihypertensive, diuretic |
| Renoquid | Defer 24 hours after course completed and feel well. | Antibiotic |
| Repen-VK | Defer 24 hours after course completed and feel well; if IV or IM defer 1 week. | Antibiotic |
| Repoise | Yes. | Tranquilizer |
| Rescinnamine | Yes, watch for orthostatic pressure changes. | Antihypertensive |
| Reserpine, Reserpoid | Yes , watch for orthostatic pressure changes. | Antihypertensive |
| Restoril | Yes. | Tranquilizer |
| Retin-A | Yes, if topical. Defer 1 month from last dose if oral. | Vitamin A derivative |
| Retinol | Yes. | Vitamin A |
| **Retrovir** | **Permanent deferral.** | **Anti-viral agent** |
| Rhinex (Teva) | Yes, if taken for allergies. Defer for 72 hours after symptoms are resolved if taken for cold/flu symptoms or for fever.  Defer 72 hrs for plateletpheresis or sole source platelets | ASA containing analgesic |
| Rhinocaps (Ferndale) | Yes, if taken for allergies. Defer for 72 hours after symptoms are resolved if taken for cold/flu symptoms or for fever.  Defer 72 hrs for plateletpheresis or sole source platelets | ASA containing analgesic |
| Rhinocort (budesonide) | Yes, even if daily dose for maintenance. | Anti-inflammatory, steroid (anti-rhinitis) |
| Rho (D) Immune Globulin, Rh_o_GAM | Defer until 6 weeks after completion of pregnancy. | Anti-D immune globulin |
| RhoGAM | Accept after pregnancy, miscarriage or abortion and meets required wait of 6 weeks. | Vaccine: Rh Immune Globulin |
| Ribavirin | Defer 6 months. | Antiviral |
| Riboflavin | Yes. | Vitamin |
| **Rifabutin** | **No, permanent deferral.** | **Prevent Mycobacterium avium complex in HIV patients** |
| *Rifadin, Rifampin* | *No, defer until course of medication completed and disease inactive.* | *Antituberculosous* |
| *RIG* | *No, defer 12 months.* | *Biological* |
| *Rimactane* | *No, defer until course of medication completed and disease inactive.* | *Antituberculosous* |
| Rimso-50 (DMSO) | Defer for 1 week after drug completed. | Anti-inflammatory, anti-spasmodic |
| Rio-Dopa | Yes. | Anti-Parkinsonism |
| Riopan | Yes, if ulcer disease pain-free. | Antacid |
| Risendronate | Yes. | For osteoporosis |
| Risperdal | Yes. | Anti-psychotic |
| Ritalin | Yes. | Stimulant |
| Ritodrine | Defer 6 weeks after pregnancy terminated. | Sympathomimetic (inhibits labor) |
| Robalate | Yes, if ulcer disease pain-free. | Antacid |
| Robamox | Defer 24 hours after course completed and feel well; if IV or IM defer 1 week. | Antibiotic |
| Robantaline | Yes, if ulcer disease pain-free. | Anticholinergic/antispasmodic |
| Robaxin | Yes. | Muscle relaxant |
| Robaxisal | Yes. Defer 72 hrs for plateletpheresis or sole source platelets | Muscle relaxant |
| Robenecid | Yes. | Uricosuric agent |
| Robicillin VK | Defer 24 hours after course completed and feel well; if IV or IM defer 1 week. | Antibiotic |
| Robimycin | Defer 24 hours after course completed and feel well; if IV or IM defer 1 week. Yes, if for acne. | Antibiotic |
| Robinul | Yes, if ulcer disease pain-free. | Anticholinergic/antispasmodic |
| Robitet | Defer 24 hours after course completed and feel well; if IV or IM defer 1 week. Yes, if for acne. | Antibiotic |
| Robitussin | Yes, if taken for allergies. Defer for 72 hours after symptoms are resolved if taken for cold/flu symptoms. | Expectorant |
| Rocatrol | No, permanent deferral if renal disease. Otherwise, yes. | Vitamin D |
| Rocephin | Defer 1 week after course completed and feel well. | Antibiotic |
| Rofecoxib (Vioxx) | Yes. | Cox-2 inhibitor, anti-inflamatory; no platelet inhibitory effect. |
| **Roferon-A** | **No, permanent deferral.** | **Anti-leukemic currently** |
| Rogaine | Yes. | Topical for hair restoration |
| Rolaids | Yes, if ulcer disease pain-free. | Antacid |
| Rolidrin | Yes, watch for orthostatic pressure changes. | Vasodilator |
| Romethocarb | Yes. | Muscle relaxant |
| Rondec | Yes, if taken for allergies. Defer for 72 hours after symptoms are resolved if taken for cold/flu symptoms. | Decongestant, antihistamine |
| Rondomycin | Defer 24 hours after course completed and feel well; if IV or IM defer 1 week. Yes, if for acne. | Antibiotic |
| Roniacol | Yes. | Vasodilator (weak) |
| Ronigen | Yes. | Vitamin B derivative |
| Ropoxy | Yes, if taken for allergies. Defer for 72 hours after symptoms are resolved if taken for cold/flu symptoms. | Analgesic |
| Ropred | No, defer 72 hours if p.o. or IM; yes, if topical or intra-articular. | Corticosteroid |
| Rowasa | Yes, if inflammatory bowel disease is under control. No, if for Crohn's Disease. | Anti-inflammatory enema |
| Roxanol | Yes, if not abuser. | Narcotic, analgesic |
| Poxiprin (Roxane) | Yes, if taken for allergies. Defer for 72 hours if taken for cold/flu symptoms or for fever.  Defer 72 hrs for plateletpheresis or sole source platelets | ASA containing analgesic |
| RP-mycin | Defer 24 hrs. after course completed and feel well; if IV or IM defer 1 wk. Yes, if for acne. | Antibiotic |
| Rubramin PC | Yes. | Vitamin |
| Rufen | Yes, if arthritis inactive. Defer plateletpheresis donors 24 hours. | Non-steroidal anti-inflammatory |
| Rumacol (Republic) | Yes, if taken for allergies. Defer for 72 hours after symptoms are resolved if taken for cold/flu symptoms or for fever.  Defer 72 hrs for plateletpheresis or sole source platelets | ASA containing analgesic |
| Rynatann Rynatuss | Yes, if taken for allergies. Defer for 72 hours after symptoms are resolved if taken for cold/flu symptoms. | Antihistamine, decongestant |
| Salabuff (Ferndale | Yes, if taken for allergies. Defer for 72 hours after symptoms are resolved if taken for cold/flu symptoms or for fever.  Defer 72 hrs for plateletpheresis or sole source platelets | ASA containing analgesic |
| Salatin Capsules (Ferndale) | Yes, if taken for allergies. Defer for 72 hours after symptoms are resolved if taken for cold/flu symptoms or for fever.  Defer 72 hrs for plateletpheresis or sole source platelets | ASA containing analgesic |
| Saleto (Roberts) | Yes, if taken for allergies. Defer for 72 hours after symptoms are resolved if taken for cold/flu symptoms or for fever.  Defer 72 hrs for plateletpheresis or sole source platelets | ASA containing analgesic |
| Salicylsalicylic, Salsalate | Yes, if arthritis inactive. | Non-steroidal anti-inflammatory |
| Salocol (Roberts) | Yes, if taken for allergies. Defer for 72 hours after symptoms are resolved if taken for cold/flu symptoms or for fever.  Defer 72 hrs for plateletpheresis or sole source platelets | ASA containing analgesic |
| Saluron | Yes. | Antihypertensive, diuretic |
| Salutensin | Yes, watch for orthostatic pressure changes. | Antihypertensive, diuretic |
| Sandril | Yes, watch for orthostatic pressure changes. | Antihypertensive |
| Sanorex | Yes. | Anorexiant |
| Sansert | Yes. | Antimigraine |
| Sarodant | Defer 24 hours after course completed and feel well; if IV or IM defer 1 week. | Antibiotic |
| Saronil | Yes. | Sedative/hypnotic |
| **SAS-500** | **No.** | **Immunomodulator** |
| Savacort | No, defer 72 hours if p.o. or IM Yes, if topical or intra-articular. | Corticosteroid |
| Secobarbital, Seconal | Yes. | Sedative/hypnotic |
| Sectral | Yes, if for hypertension. No, if for arrhythmias. | Beta-Blocker, antihypertensive |
| Sedadrops | Yes. | Anticonvulsant, sedative |
| Sed-Tens SE | Yes, if ulcer disease pain-free. | Anticholinergic/antispasmodic |
| Selacryn | Yes. | Antihypertensive |
| Seldane | Yes, if taken for allergies. Defer for 72 hours after symptoms are resolved if taken for cold/flu symptoms. | Antihistamine |
| Selegiline Hcl | Yes. | Anti-Parkinsonism |
| Semilente Iletin, Semilente Insulin | Yes, if dosage controlled and stable. | Hypoglycemic |
| Semprex D | Yes, if taken for allergies. Defer for 72 hours after symptoms are resolved if taken for cold/flu symptoms. | Antihistamine |
| **Semustine** | **No, permanent deferral.** | **Antineoplastic** |
| Senna Pod, Senokot | Yes. | Laxative |
| Septra | Defer 24 hours after course completed and feel well; if IV or IM defer 1 week. Yes, for rosacea. | Antibiotic |
| Ser-Ap-Es | Yes, watch for orthostatic pressure changes. | Antihypertensive |
| Serax | Yes. | Anticonvulsant, antianxiety |
| Serensil | Yes. | Hypnotic/sedative |
| Serentil | Yes, if mentally and legally responsible. | Antipsychotic |
| Serephene | Yes. | Fertility agent |
| Serevent Inhaler | Yes. | Anti-asthmatic |
| Seroquel | Yes, If legally and mentally responsible | Anti-Psychotic |
| *Seromycin* | *No, defer until course of medication completed and disease inactive.* | *Antituberculous, antibiotic* |
| Serpasil, Serpasil-Apresoline, Serpasil-Esidrix | Yes, watch for orthostatic pressure changes. | Antihypertensive |
| Sertabs | Yes, watch for orthostatic pressure changes. | Antihypertensive |
| Servisone | Defer 72 hours if P.O. or IM; yes, if topical or intra-articular. | Corticosteroid |
| Serzone | Yes. | Anti depressant |
| Simeco | Yes, if ulcer disease pain-free. | Antacid |
| Simron | Yes. | Hematinic |
| Simvastatin | Yes. | Cholesterol-lowering agent |
| Sinarest | Yes, if taken for allergies. Defer for 72 hours after symptoms are resolved if taken for cold/flu symptoms. | Antihistamine |
| Sinemet | Yes. | Anti-Parkinsonism |
| Sine-Off Sinus (Smith-Kline) | Yes, if taken for allergies. Defer for 72 hours after symptoms are resolved if taken for cold/flu symptoms or for fever.  Defer 72 hrs for plateletpheresis or sole source platelets | ASA containing analgesic |
| Sinequan | Yes. | Antidepressant, antianxiety |
| Singlet | Yes, if taken for allergies. Defer for 72 hours after symptoms are resolved if taken for cold/flu symptoms. | Antihistamine, decongestant |
| Singulair | Yes. | Prophylaxis and treatment for chronic asthma |
| Sinubid | Yes, if taken for allergies. Defer for 72 hours after symptoms are resolved if taken for cold/flu symptoms. | Antihistamine, decongestant |
| Sinutab | Yes, if taken for allergies. Defer for 72 hours after symptoms are resolved if taken for cold/flu symptoms. | Antihistamine, decongestant |
| Sitosterols | Yes. | Cholesterol lowering agent |
| SK-65 | Yes, if taken for allergies. Defer for 72 hours after symptoms are resolved if taken for cold/flu symptoms or for fever.  Defer 72 hrs for plateletpheresis or sole source platelets | Analgesic, ASA |
| SK-Amitriptyline | Yes. | Tricyclic antidepressant |
| SK-Ampicillin | No, wait 24 hrs. after course completed and feel well, if IV or IM defer 1 wk. | Antibiotic |
| SK-Bamate | Yes, if not abuser. | Sedative/hypnotic |
| SK-Dexamethasone | Defer 72 hrs. if oral or IM, otherwise yes. | Steroid |
| Skelaxin | Yes. | Muscle relaxant (may elevate liver function tests) |
| SK-Lygen | Yes. | Tranquilizer |
| SK-Pramine | Yes. | Tricyclic antidepressant |
| SK-Reserpine | Yes , watch for orthostatic pressure changes. | Antihypertensive |
| SK-Soxazole | Defer 24 hours after course completed and feel well; if IV or IM defer 1 week. | Antibiotic |
| SK-Tetracycline | Defer 24 hours after course completed and feel well; if IV or IM defer 1 week. Yes, if for acne. | Antibiotic |
| Slim-Tabs | Yes. | Anorexiant |
| Slo-Bid | Yes, even if daily dose for maintenance. | Bronchodilator |
| Slo-Phyllin | Yes, even if daily dose for maintenance. | Bronchodilator |
| Sloprin (Econolab) | Yes, if taken for allergies. Defer for 72 hours after symptoms are resolved if taken for cold/flu symptoms or for fever.  Defer 72 hrs for plateletpheresis or sole source platelets | ASA containing analgesic |
| Slow K | Yes. | Potassium replacement |
| **Sodium Polystyrene** | **No, permanent deferral.** | **Potassium binding resin** |
| Sodium Salicylate | Yes, if arthritis inactive. | Anti-inflammatory |
| Sodol (Major) | Yes, if taken for allergies. Defer for 72 hours after symptoms are resolved if taken for cold/flu symptoms or for fever.  Defer 72 hrs for plateletpheresis or sole source platelets | ASA containing analgesic |
| Solatene | Yes, if skin is not orange. | Beta Carotene Sulfonate |
| Solganal | Yes, if arthritis inactive. | Anti-inflammatory |
| Solu-Cortef | Defer 72 hours if p.o. or IM; yes, if topical or intra-articular. | Cortisol |
| Soluject | Defer 72 hours if p.o. or IM; | Corticosteroid |
| Solu-Medrol | yes, if topical or intra-articular. |  |
| Soma | Yes. Defer 72 hrs for plateletpheresis or sole source platelets | Sedative/hypnotic |
| Soma w/Codeine | Yes, if taken for allergies. Defer for 72 hours after symptoms are resolved if taken for cold/flu symptoms or for fever.  Defer 72 hrs for plateletpheresis or sole source platelets | ASA containing analgesic |
| Somatrem | Yes. | Growth hormone (recombinant) |
| Somatropin | Yes. | Growth hormone (recombinant) |
| Somophyllin | Yes, even if daily dose for maintenance. | Bronchodilator |
| Sonilyn | Defer 24 hours after course | Sedative/hypnotic |
| Sopor | Yes, if not abuser. | Sedative/hypnotic |
| *Sorbitrate* | *Medical Director evaluation required* | *Antianginal* |
| Sorboquel | Yes. | Laxative |
| **Soriatane (Aciretin)** | **Permanent Deferral (teratogenic)** | **Anti-psoriasis** |
| Sparine | Yes. | Tranquilizer |
| Spastil | Yes, if ulcer disease pain-free. | Anticholinergic/antispasmodic |
| Spectinomycin | Defer 1 week after injection. | Antibiotic |
| Spectrobid | Defer 24 hours after course completed and feel well; if IV or IM defer 1 week. | Antibiotic |
| Spironolactone | Yes. | Diuretic |
| Sporonox | Yes if for superficial fungal infection. No, if for systemic (generalized) infection. | Anti-fungal |
| St Joseph Chewable | Yes, if taken for allergies. Defer for 72 hours after symptoms are resolved if taken for cold/flu symptoms or for fever.  Defer 72 hrs for plateletpheresis or sole source platelets | ASA containing analgesic |
| Stadol | Yes, if not abuser. | Narcotic, analgesic |
| Stanback Power | Yes, if taken for allergies. Defer for 72 hours after symptoms are resolved if taken for cold/flu symptoms or for fever.  Defer 72 hrs for plateletpheresis or sole source platelets | ASA containing analgesic |
| Staphcillin | Defer 1 week, if IV or IM use. | Antibiotic |
| Staticin | Yes. | Topical antibiotic |
| Statobex | Yes. | Anorexiant |
| Stearane | Defer 72 hours if p.o. or IM; yes, if topical or intra-articular. | Corticosteroid |
| Stelazine | Yes, if mentally and legally responsible. | Antipsychotic |
| Sterazolidin | Defer 72 hours if p.o. or IM; yes, if topical or intra-articular. | Corticosteroid |
| Stero-Darvon | Yes, if arthritis inactive. | Arthritis, anti-inflammatory |
| Stilphosterol | No, if for cancer; otherwise, yes. | Estrogen |
| Stim 250 | Yes. | Stimulant |
| Stimdex | Yes. | Stimulant |
| *Streptomycin* | *No, defer until disease inactive.* | *Antituberculous* |
| Sturartinic | Yes. | Hematinic |
| **Sub-Quin** | **No, permanent deferral for heart disease.** | **Antiarrhythmic** |
| Sucralfate | Yes, if ulcer disease pain-free. | Antiulcer |
| Sudafed | Yes, if taken for allergies. Defer for 72 hours after symptoms are resolved if taken for cold/flu symptoms. | Antihistamine |
| Sular | Yes. | Antihypertensive |
| Sulfa-chlorpyridazine, Sulfacytine, Sulfalar, Sulfameter, Sulfamethizole, Sulfamethoxazole | Defer 24 hours after course completed and feel well; if IV or IM defer 1 week. Yes for acne. | Antibiotic |
| **Sulfasalazine** | **No, permanent deferral.** | **Immunomodulator** |
| Sulfathalidine, Sulfisoxazole, Sulfose | Defer 24 hours after course completed and feel well; if IV or IM defer 1 week. | Antibiotic |
| **Sulfoxone** | **No, permanent deferral.** | **Antileprosy** |
| Sulindac | Yes, if arthritis inactive. Defer plateletpheresis donors 24 hours. | Non-steroidal, anti-inflammatory |
| Sulla | Defer 24 hours after course completed and feel well; if IV or IM defer 1 week. Yes for acne. | Antibiotic |
| Sumatriptan Succinate | Accept if migraine symptoms are resolved. Defer until next day after receiving injection. | Blocks production of Serotonin |
| Sumox | Defer 24 hours after course completed and feel well; if IV or IM defer 1 week. | Antibiotic |
| Sumycin | Defer 24 hours after course completed and feel well; if IV or IM defer 1 week. Yes, if for acne or rosacea. | Antibiotic |
| Supac (Mission) | Yes, if taken for allergies. Defer for 72 hours after symptoms are resolved if taken for cold/flu symptoms or for fever.  Defer 72 hrs for plateletpheresis or sole source platelets | ASA containing analgesic |
| Supen | Defer 24 hours after course completed and feel well; if IV or IM defer 1 week. | Antibiotic |
| Suprax | Defer 24 hours after course completed and feel well. | Antibiotic |
| Suprin (Invamed) | Yes, if taken for allergies. Defer for 72 hours after symptoms are resolved if taken for cold/flu symptoms or for fever.  Defer 72 hrs for plateletpheresis or sole source platelets | ASA containing analgesic |
| Suprofen | Yes, if arthritis inactive.  Yes, if taken for allergies. Defer for 72 hours after symptoms are resolved if taken for cold/flu symptoms or for fever.  Defer plateletpheresis donors 24 hours. | Non-steroidal anti-inflammatory. Analgesic |
| Suprol | Yes, if arthritis inactive. No, if for fever. Defer plateletpheresis donors 24 hours. | Non-steroidal anti-inflammatory. Analgesic |
| Surfak | Yes. | Laxative |
| Surfol | Yes. | Laxative |
| Surmontil | Yes. | Antidepressant |
| Sustaire | Yes, even if daily dose for maintenance. | Bronchodilator |
| Symmetrel | Yes. | Anti-Parkinson, Antiviral agent |
| Symptom 2, Symptom 3 | Yes, if taken for allergies. Defer for 72 hours after symptoms are resolved if taken for cold/flu symptoms. | Antihistamine |
| Synalar | Yes. | Topical steroid |
| Synalgos | Yes, if taken for allergies. Defer for 72 hours after symptoms are resolved if taken for cold/flu symptoms or for fever.  Defer 72 hrs for plateletpheresis or sole source platelets | Analgesic, ASA |
| Synarel | Yes. | Use in managing endometriosis |
| *Synkavite* | *Evaluate underlying condition.* | *Vitamin K* |
| Synthroid | Yes. | Thyroid replacement |
| Synvisc | Yes. Defer 1-week after injection. | Injection for arthritis. |
| Sytobex | Yes. | Vitamin B_12_ |
| Tabron | Yes. | Hematinic |
| Tacaryl | Yes, if taken for allergies. Defer for 72 hours after symptoms are resolved if taken for cold/flu symptoms. | Antihistamine |
| **TACE** | **No, permanent deferral.** | **Antineoplastic** |
| Tacrine | Yes, if legally and mentally responsible. May cause elevated ALT. | Anti-Dementia, Alzheimer's disease |
| Tagamet | Yes, if ulcer disease pain-free. | H2 receptor agonist |
| Talacen | Yes, if not abuser. | Analgesic, narcotic |
| Talwin | Yes, if not abuser.  Yes, if taken for allergies. Defer for 72 hours after symptoms are resolved if taken for cold/flu symptoms.  Defer 72 hrs for plateletpheresis or sole source platelets | Analgesic |
| Tamazepam | Yes. | Antianxiety agent |
| *Tambocor* | *No, defer until off the medication and stable.* | *Antiarrhythmic* |
| *Tamoxifen* | *No, permanent deferral unless used to treat non-malignant disease.* | *Antineoplastic* |
| Tamsulosin | Yes. | Used in treating benign prostatic hypertrophy |
| Tandearil | Yes, if arthritis inactive. | Anti-inflammatory |
| Tao | Defer 24 hours after course completed and feel well; if IV or IM defer 1 week. | Antibiotic |
| Tapazole | Yes. | Anti-thyroid |
| Taractan | Yes, evaluate underlying condition. | Potent tranquilizer |
| Tarka | Yes, for BP. | Calcium channel blocker |
| Tavist | Yes, if taken for allergies. Defer for 72 hours after symptoms are resolved if taken for cold/flu symptoms. | Antihistamine |
| Tazarotene | Defer 2 weeks | Topical Gel for acne, psoriasis |
| Tazicef | Defer 1 week. | Antibiotic |
| Tazidime | Defer 1 week. | Antibiotic |
| Technetium99 | Defer 2½ days, but should evaluate why test done and it's results. | Radioactive label |
| Tedral | Yes, even if daily dose for maintenance. | Bronchodilator |
| Teebacin | Yes. | Anti-inflammatory |
| **Tegison (etretinate)** | **No, permanent deferral.** | **Antipsoriasis agent (teratogenic)** |
| Tegopen | Defer 24 hours after course completed and feel well; if IV or IM defer 1 week. | Antibiotic |
| Tegretol | Yes. | Anticonvulsant, analgesic |
| Teldrin | Yes, if taken for allergies. Defer for 72 hours after symptoms are resolved if taken for cold/flu symptoms. | Antihistamine |
| Temaril | Yes, evaluate underlying condition.  Yes, if taken for allergies. Defer for 72 hours after symptoms are resolved if taken for cold/flu symptoms. | Antihistamine, Antipruitic |
| Temazepam | Yes. | Hypnotic/sedative |
| Temovate (clobetasol propionate) | Yes. | Topical steroid |
| Tempra | Yes. | Antipyretic |
| Tenex | Yes. | Antihypertensive |
| Tenol Plus | Yes, if taken for allergies. Defer for 72 hours after symptoms are resolved if taken for cold/flu symptoms or for fever.  Defer 72 hrs for plateletpheresis or sole source platelets | ASA containing analgesic |
| Tenoretic | Yes. | Anti-hypertensive (beta blocker) |
| Tenormin | Yes, if for hypertension. Yes for MVP and has no restrictions. No, if for angina. | Anti-hypertensive (beta blocker). Antianginal |
| Tenuate | Yes. | Anorexiant |
| Tepanil | Yes. | Anorexiant |
| Terazol (vaginal cream) | Yes. | Antifungal |
| Terbutaline | Yes, even if daily dose for maintenance. | Bronchodilator |
| Terfenadine | Yes, if taken for allergies. Defer for 72 hours after symptoms are resolved if taken for cold/flu symptoms. | Antihistamine |
| Terfonyl | Defer 24 hours after course completed and feel well; if IV or IM defer 1 week. | Antibiotic |
| Terpin Hydrate | Yes, if taken for allergies. Defer for 72 hours after symptoms are resolved if taken for cold/flu symptoms. | Expectorant |
| Terramycin, Terrastatin | Defer 24 hours after course completed and feel well; if IV or IM defer 1 week. Yes, if for acne. | Antibiotic |
| **Teslac** | **No, permanent deferral.** | **Antineoplastic agent** |
| Tessalon | Yes, if taken for allergies. Defer for 72 hours after symptoms are resolved if taken for cold/flu symptoms. | Antitussive |
| Testoject-E.P. | Yes. | Hormone |
| **Testolactone** | **No, permanent deferral.** | **Antineoplastic** |
| Testosterone | Yes. | Hormone |
| Testred | Yes. | Hormone |
| Tetanus Antitoxin (Equine) | Defer 3 months. | Biological |
| Tetanus Immune Globulin | No, defer 6 months. | Biological |
| Tetrachel | Defer 24 hours after course completed and feel well; if IV or IM defer 1 week. Yes, if for acne. | Antibiotic |
| Tetracycline, Tetracyn, Tetrastatin, Tetrex | Defer 24 hours after course completed and feel well; if IV or IM defer 1 week. Yes, if for acne. | Antibiotic |
| Theelin | Yes. | Hormone |
| Theobid, Theocap, Theoclear 80, Theo-Dor, Theolair, Theo-Organidin, Theophyl, Theospan | Yes, even if daily dose for maintenance. | Bronchodilator |
| Theracebrin | Yes. | Vitamins |
| Theragran | Yes. | Vitamins/hematinic |
| Theramycin | Defer 24 hrs. after course completed and feel well; if IV or IM defer 1 wk. Yes, if for acne. | Antibiotic |
| Therapy Bayer Asprin | Yes, if taken for allergies. Defer for 72 hours after symptoms are resolved if taken for cold/flu symptoms or for fever.  Defer 72 hrs for plateletpheresis or sole source platelets | ASA containing analgesic |
| Thiabendazole | Defer 1 week after course completed and feel well. | Antihelminthic |
| Thiamine | Yes. | Vitamin |
| Thiamozole | Yes. | Antithyroid |
| Thiethylperazine | Yes, if symptom free. | Antiemetic |
| **Thioguanine** | **No, permanent deferral.** | **Antineoplastic** |
| Thioridazine | Yes, if mentally and legally responsible. | Antipsychotic |
| Thiosulfil | Defer 24 hours after course completed and feel well; if IV or IM defer 1 week. | Antibiotic |
| Thiothixene | Yes, if mentally and legally responsible. | Antipsychotic |
| Thiphenamil | Yes, if ulcer disease pain-free. | Anticholinergic/antispasmodic |
| Thiuretic | Yes. | Antihypertensive, diuretic |
| Thorazine | Yes, if mentally and legally responsible. | Antipsychotic |
| Thyrar | Yes. | Thyroid replacement |
| Thyroglobulin, Thyroid Extract | Yes. | Thyroid replacement |
| Thyrolar | Yes. | Thyroid replacement |
| Tiazac (diltiazem HCl) | Yes, for hypertension | Calcium channel antagonist |
| Ticlid (ticlopidine HCl) | Yes for whole blood, FFP, RBC or cryopricipitate. Defer 2 wks for platelets. | Platelet aggregate inhibitor (treat as ASA, circle box) |
| Tigan | Yes, if symptom free. | Antinausea |
| Tilade inhaler | Yes. | Anti-asthmatic |
| Timentin | Defer until 1 week after course completed. | Antibiotic (IV) |
| Timolol | Yes, if for hypertension; no, if for arrhythmia or angina. | Antiarrhythmic, antianginal, antihypertensive |
| Tindal | Yes, if mentally and legally responsible. | Antipsychotic |
| Tirend | Yes. | Stimulant |
| Titralac | Yes, if ulcer disease pain-free. | Antacid |
| *Tocainide* | *No, defer until off medication and symptom free.* | *Antiarrhythmic* |
| Tocopherol | Yes. | Vitamin E |
| Tofranil | Yes. | Tranquilizer |
| Tolazamide | Yes. | Oral hypoglycemic |
| Tolazoline | Yes, watch for orthostatic pressure changes. | Vasodilator |
| Tolbutamide | Yes. | Oral hypoglycemic |
| Tolectin | Yes, if arthritis inactive. Defer plateletpheresis donors 72 hours. | Anti-inflammatory |
| Toleron | Yes. | Hematinic |
| Tolinase | Yes. | Oral hypoglycemic |
| Tolmetin | Yes, if arthritis inactive. Defer plateletpheresis donors 72 hours. | Anti-inflammatory |
| *Tonocard* | *No, defer until off medication and symptom free.* | *Antiarrhythmic* |
| Toprol XL (Metoprolol) | Yes, for blood pressure. | Beta blocker |
| Tora | Yes. | Anorexiant |
| Toradol | Yes, if arthritis inactive.  Yes, if taken for allergies. Defer for 72 hours after symptoms are resolved if taken for cold/flu symptoms.  Defer plateletpheresis donors 72 hours. | Non steroidal anti-inflammatory, analgesic. |
| Torecan | Yes. | Antiemetic |
| Torsemide | Yes. | Diuretic |
| Totacillin | Defer 24 hours after course completed and feel well; if IV or IM defer 1 week. | Antibiotic |
| Tralmag | Yes, if ulcer disease pain-free | Antacid |
| Tramadol | Yes, if taken for allergies. Defer for 72 hours after symptoms are resolved if taken for cold/flu symptoms. | Analgesic |
| Trandate | Yes. | Anti-hypertensive (beta blocker) |
| Tranmep | Yes. | Sedative/hypnotic |
| Tranxene | Yes. | Tranquilizer |
| Tranylcypromine | Yes. | Antidepressant |
| Trazodone | Yes. | Antidepressant |
| *Trecator-SC* | *No, defer until disease inactive.* | *Antituberculous* |
| Tremin | Yes. | Anti-Parkinson |
| Trental | Yes. | Anti-claudication |
| Trest | Yes, if ulcer disease pain-free. | Anticholinergic, antispasmodic |
| Trexan | Yes, if not abuser. | Narcotic antagonist |
| Triact | Yes. | Laxative |
| Triamcinolone | Defer 72 hours if p.o. or IM. Yes, if topical or intra-articular. | Corticosteroid |
| Triaminic, Triaminicin, Triaminicol | Yes, if taken for allergies. Defer for 72 hours after symptoms are resolved if taken for cold/flu symptoms.  Defer 72 hrs for plateletpheresis or sole source platelets | Antitussive, antihistamine |
| Triamterene | Yes. | Diuretic |
| Triavil | Yes, if mentally and legally responsible. | Antipsychotic, antidepressant |
| Tricholormethiazide | Yes. | Antihypertensive, diuretic |
| Triclofos, Triclos | Yes. | Hypnotic/sedative |
| Tridihexethyl | Yes, if ulcer disease pain-free. | Anticholinergic/antispasmodic |
| Tridione | Yes. | Anticonvulsant |
| Trifluoperazine | Yes, if mentally and legally responsible. | Antipsychotic |
| Triflupromazine | Yes, if mentally and legally responsible. | Antipsychotic, antiemetic |
| Trigesic | Yes, if taken for allergies. Defer for 72 hours after symptoms are resolved if taken for cold/flu symptoms or for fever.  Defer 72 hrs for plateletpheresis or sole source platelets | ASA containing analgesic |
| Trihemic 600 | Yes. | Hematinic |
| Trihexyphenidyl | Yes. | Anti-Parkinson |
| Trilafon | Yes, if mentally and legally responsible. | Antipsychotic |
| Trilisate | Yes, if arthritis inactive. | Anti-inflammatory |
| Trimeprazine | Yes, if taken for allergies. Defer for 72 hours after symptoms are resolved if taken for cold/flu symptoms. | Antihistamine |
| Trimethadione | Yes. | Anticonvulsant |
| Trimethaphan camsylate | Defer 72 hours. | Ganglionic blocking agent. (potent hypotensive) |
| Trimethobenzamide | Yes, if symptom free. | Antinauseant |
| Trimethoprim | Defer 24 hours after course completed and feel well. | Antibiotic |
| Trimipramine | Yes. | Antidepressant |
| Trimox | Defer 24 hours after course completed and feel well; if IV or IM defer 1 week. | Antibiotic |
| Trimpex | Defer 24 hours after course completed and feel well. | Antibiotic |
| Trimtab | Yes. | Anorexiant |
| Trinalin | Yes, if taken for allergies. Defer for 72 hours after symptoms are resolved if taken for cold/flu symptoms. | Antihistamine |
| Trind | Yes, if taken for allergies. Defer for 72 hours after symptoms are resolved if taken for cold/flu symptoms. | Decongestant, antihistamine |
| Trinprin (Trinity) | Yes, if taken for allergies. Defer for 72 hours after symptoms are resolved if taken for cold/flu symptoms or for fever.  Defer 72 hrs for plateletpheresis or sole source platelets | ASA containing analgesic |
| Trinsicon | Yes. | Vitamins/iron |
| Tri-Pain Caplets | Yes, if taken for allergies. Defer for 72 hours after symptoms are resolved if taken for cold/flu symptoms or for fever.  Defer 72 hrs for plateletpheresis or sole source platelets | ASA containing analgesic |
| Tripelennamine | Yes, if taken for allergies. Defer for 72 hours after symptoms are resolved if taken for cold/flu symptoms. | Antihistamine |
| Triprolidine | Yes, if taken for allergies. Defer for 72 hours after symptoms are resolved if taken for cold/flu symptoms. | Antihistamine |
| Trisalate | Yes, if arthritis inactive. | Anti-inflammatory |
| Trisogel | Yes, if ulcer disease pain-free. | Antacid |
| Trisomen | Yes, if ulcer disease pain-free. | Antacid |
| Trisoralen | Yes. | Pigmentation agent |
| Trisulfapyrimidines | Defer 24 hours after course completed and feel well. | Antibiotic |
| Triten | Yes, if taken for allergies. Defer for 72 hours after symptoms are resolved if taken for cold/flu symptoms. | Antihistamine |
| Tri-Vi-Flor | Yes. | Vitamins |
| Trobicin | Defer 24 hours after course completed and feel well; if IV or IM defer 1 week. | Antibiotic |
| Trocinate | Yes, if ulcer disease pain-free. | Anticholinergic/antispasmodic |
| Troleandomycin | Defer 24 hours after course completed and feel well; if IV or IM defer 1 week. | Antibiotic |
| Trusopt | Yes. | Eye drops for glaucoma |
| Tuinal | Yes, if not abuser. | Hypnotic |
| Tums | Yes, if ulcer disease pain-free. | Antacid |
| Tussagesic | Yes, if taken for allergies. Defer for 72 hours after symptoms are resolved if taken for cold/flu symptoms. | Antihistamine, decongestant, antitussive |
| Tussar, Tussend, Tussionex | Yes, if taken for allergies. Defer for 72 hours after symptoms are resolved if taken for cold/flu symptoms. | Antihistamine, decongestant |
| Tussi-Organidin | Yes, if taken for allergies. Defer for 72 hours after symptoms are resolved if taken for cold/flu symptoms. | Antitussive, antihistamine |
| Tuss-Ornade | Yes, as anticholinergic. Yes, if taken for allergies. Defer for 72 hours after symptoms are resolved if taken for cold/flu symptoms. | Antihistamine, anticholinergic |
| Twin-K-C1 | Yes. | Potassium replacement |
| Tylenol | Yes, if taken for allergies. Defer for 72 hours after symptoms are resolved if taken for cold/flu symptoms. | Antipyretic, Analgesic |
| Tylenol #3 | Yes, if taken for allergies. Defer for 72 hours after symptoms are resolved if taken for cold/flu symptoms. | Analgesic |
| Tylox | Yes, if taken for allergies. Defer for 72 hours after symptoms are resolved if taken for cold/flu symptoms. | Analgesic |
| Ulacort | Defer 72 hours if p.o. or IV; yes, if topical or intra-articular. | Corticosteroid |
| Ultracef | Defer 24 hours after course completed and feel well; if IV or IM defer 1 week. | Antibiotic |
| **Ultralente Iletin Ultralente Insulin** | **No, permanent deferral.** | **Hypoglycemic** |
| Ultram | Yes, if taken for allergies. Defer for 72 hours after symptoms are resolved if taken for cold/flu symptoms. | Analgesic |
| Unasyn | Defer for one week after stops medication. | Antibiotic |
| Unicap | Yes. | Vitamins |
| Unidur (theophylline) | Yes. | Anti-asthmatic |
| Unipen | Defer 24 hours after course completed and feel well; if IV or IM defer 1 week. | Antibiotic |
| Uniphyl | Yes, even if daily dose for maintenance. | Bronchodilator |
| Unitensen | Yes. | Antihypertensive |
| Univasc | Yes | Ace Inhibitor |
| Upotoin | Defer 24 hours after course completed and feel well; if IV or IM defer 1 week. | Antibiotic |
| Uracel | Yes, if arthritis inactive. | Anti-inflammatory |
| Urecholine | Yes. | Uricosuric |
| Urestrin | Yes. | Hormone |
| Urex | Defer 24 hours after course completed and feel well. | Antiseptic |
| Urised | Defer 24 hours after course completed and feel well. | Antiseptic |
| Urispas | Yes, if ulcer disease pain-free and/or urinary tract infection resolved. | Anticholinergic/antispasmodic |
| Urobiotic | Defer 24 hours after course completed and feel well. | Antibiotic |
| Urodine | Defer till infection is cleared; yes, if not has active infection.  Yes, if taken for allergies. Defer for 72 hours after symptoms are resolved if taken for cold/flu symptoms. | Analgesic |
| Urolax | Yes. | Uricosuric |
| Ursinus Inlay-Tabs (Sandoz) | Yes, if taken for allergies. Defer for 72 hours after symptoms are resolved if taken for cold/flu symptoms or for fever.  Defer 72 hrs for plateletpheresis or sole source platelets. | ASA containing analgesic |
| Ursodiol | Yes. | Gallstone dissolution agent |
| Utibid | Defer 24 hours after course completed and feel well. | Antiseptic |
| Uticillin VK | Defer 24 hours after course completed and feel well; if IV or IM defer 1 week. | Antibiotic |
| Utimox | Defer 24 hours after course completed and feel well; if IV or IM defer 1 week. | Antibiotic |
| Vagisec | Yes. | Topical antiseptic |
| Vagitrol | Yes. | Topical antiseptic |
| Valadol | Yes. | Antipyretic, analgesic |
| Valesin | Yes, if taken for allergies. Defer for 72 hours after symptoms are resolved if taken for cold/flu symptoms or for fever.  Defer 72 hrs for plateletpheresis or sole source platelets. | ASA containing analgesic |
| Valium | Yes. | Tranquilizer, anticonvulsant |
| Valmid | Yes. | Sedative/hypnotic |
| Valpin | Yes, if ulcer disease pain-free. | Anticholinergic/antispasmodic |
| Valproic Acid | Yes. | Anticonvulsant |
| Valrelease | Yes. | Muscle relaxant |
| Valsartan (Diovan) | Yes. | Angiotensin blocker (receptor) |
| Valtrex | Yes, if no active lesions. | Antiviral for Herpes virus |
| Vancenase (Azmacort) | See Azmacort |  |
| Vanceril Inhaler | Yes, even if daily dose for maintenance. | Anti-inflammatory steroid |
| Vanobid | Yes. | Topical antifungal |
| Vanquish | Yes. Defer 72 hrs for plateletpheresis or sole source platelets | Anti-inflammatory, antipyretic |
| Vansil | Defer 1 week after course completed and feel well. | Antihelminthic |
| Vantin | Defer for 24 hrs. after course completed and feel well. | Antibiotic |
| Vapo-Iso | Yes, even if daily dose for maintenance. | Bronchodilator |
| Vaponefrin | Yes, even if daily dose for maintenance. | Bronchodilator |
| Varicella-Zoster Immune Globulin | Defer 6 months. | Biological |
| Vasal | Yes; no, if for arrhythmia. | Vasodilator |
| Vascor | Yes | Calcium Channel Blocker |
| *Vascunitol* | *No, defer until off drug and symptom free.* | *Antianginal* |
| *Vasitol* | *No, until off drug and symptom free. Evaluate underlying condition.* | *Antianginal* |
| Vasodilan | Yes. | Beta adrenergic stimulant (watch for orthostatic change) |
| **Vasopressin** | **No, permanent deferral.** | **Antidiuretic hormone** |
| Vasoretic | Yes, if for hypertension. No, if for heart disease. | Antihypertensive, ACE inhibitor |
| Vasospan | Yes; no, if for arrhythmia. | Vasodilator |
| Vasotec | Yes, if for hypertension. No, if for heart disease. | Antihypertensive, ACE inhibitor |
| V-Cillin, V-Cillin K | Defer 24 hours after course completed and feel well; if IV or IM defer 1 week. | Antibiotic |
| Vectrin | Defer 24 hours after course completed and feel well; if IV or IM defer 1 week. Yes, if for acne. | Antibiotic |
| Veetids | Defer 24 hours after course completed and feel well; if IV or IM defer 1 week. | Antibiotic |
| Velosef | Defer 24 hours after course completed and feel well; if IV or IM defer 1 week. | Antibiotic |
| Ventolin, Ventyl | Yes, even if daily dose for maintenance. | Bronchodilator |
| **VePesid** | **Permanent deferral.** | **Chemotherapeutic agent** |
| Veracillin | Defer 24 hours after course completed and feel well; if IV or IM defer 1 week. | Antibiotic |
| Verapamil | Yes, if for hypertension. No, if for angina or arrhythmia. | Antianginal, antiarrhythmic, antihypertensive |
| Verelan | Yes, if for hypertension. No, if for angina or arrhythmia. | Antianginal, antiarrhythmic, antihypertensive |
| Verin | Yes, if taken for allergies. Defer for 72 hours after symptoms are resolved if taken for cold/flu symptoms or for fever.  Defer 72 hrs for plateletpheresis or sole source platelets. | ASA containing analgesic |
| Vermazine | Defer 1 week after course completed and feel well. | Antihelminthic |
| Vermox | Defer 1 week after course completed and feel well. | Antihelminthic |
| Versapen | Defer 24 hours after course completed and feel well; if IV or IM defer 1 week. | Antibiotic |
| Versed | Yes. | Sedative |
| Verstran | Yes. | Tranquilizer |
| Vertrol | Yes, as antiemetic. Yes, if taken for allergies. Defer for 72 hours after symptoms are resolved if taken for cold/flu symptoms. | Antihistamine, antiemetic |
| Vesicholine | Yes. | Cholinomimetic |
| Vesprine | Yes. | Tranquilizer, antiemetic |
| Viagra | Defer 12 hours. | For erectile dysfunction |
| Vibraform | Yes, if lesions not in antecubital region. | Anti-fungal |
| Vibramycin, Vibra-Tabs | Defer 24 hours after course completed and feel well; if IV or IM defer 1 week. Yes, if for acne. | Antibiotic |
| Vicodin | Yes, if taken for allergies. Defer for 72 hours after symptoms are resolved if taken for cold/flu symptoms. | Antitussive, analgesic |
| Vi-Daylin | Yes. | Vitamins |
| Vigran | Yes. | Vitamins |
| Vi-Magma | Yes. | Vitamins |
| Viokase | Yes. | Enzyme replacement |
| Vio-Serpine | Yes, watch for orthostatic pressure changes. | Antihypertensive |
| Vioxx (Rofecoxib) | See Rofecoxib |  |
| Virazole | Defer 6 months | Antiviral |
| Visken | Yes if for hypertension. No, if for Arrhythmias | Antiarrhythmic, antihypertensive |
| Vistaril | Yes. | Anti-anxiety |
| Vi-Syneral, Vitamins A, B, B_2_, B_6_, B_12_, C | Yes. | Vitamins |
| Vitamin D | No, if renal disease, otherwise, yes. | Vitamin |
| Vitamin K_1_, K_3_ | Evaluate underlying condition. | Vitamins |
| Vitamins E, C | Yes. | Vitamins |
| Viterra C, E | Yes. | Vitamins |
| Vitron C | Yes. | Vitamin |
| Vivactil | Yes. | Antidepressant |
| Vivarin | Yes. | Stimulant |
| Vizac | Yes. | Vitamin C,E,A preparation |
| Voltaren | Yes, if arthritis inactive. Defer 72 hours if for plateletpheresis. | Non-steroidal anti-inflammatory. |
| Vontrol | Yes. | Antiemetic |
| Voranil | Yes. | Anorexiant |
| *Warfarin* | *Defer pending medical evaluation with cessation of drug.* | *Anticoagulant* |
| Wellbutrin | Yes. | Anti-depressant |
| **Wellcovorin** | **No, permanent deferral.** | **Correct antifolate effect of methotrexate** |
| Wesprin Buffered (Wesley) | Yes, if taken for allergies. Defer for 72 hours after symptoms are resolved if taken for cold/flu symptoms or for fever.  Defer 72 hrs for plateletpheresis or sole source platelets. | ASA containing analgesic |
| Wigraine | Yes, if taken for allergies. Defer for 72 hours after symptoms are resolved if taken for cold/flu symptoms. | Analgesic |
| Win Gel | Yes, if ulcer disease pain-free. | Antacid |
| Winstrol | Yes, if not renal disease. | Anabolic steroid |
| Wycillin | Defer 24 hours after course completed and feel well; if IV or IM defer 1 week. | Antibiotic |
| Wygesic | Yes, if taken for allergies. Defer for 72 hours after symptoms are resolved if taken for cold/flu symptoms. | Analgesic, antipyretic |
| Wymox | Defer 24 hours after course completed and feel well; if IV or IM defer 1 week. | Antibiotic |
| Wytensin | Yes. | Antihypertensive |
| Xanax | Yes. | Tranquilizer |
| Yocon | Yes. | Antihypertensive, aphrodisiac |
| Yodoxin | Defer 1 week after course completed and feel well. | Amebicide |
| Yohimbine hydrochloride | See Yocon. |  |
| Yutopar | Defer 6 weeks after pregnancy terminated. | Sympathomimetic (inhibits labor) |
| Zactane | Yes, if taken for allergies. Defer for 72 hours after symptoms are resolved if taken for cold/flu symptoms. | Analgesic |
| Zactirin | Yes, if taken for allergies. Defer for 72 hours after symptoms are resolved if taken for cold/flu symptoms or for fever.  Defer 72 hrs for plateletpheresis or sole source platelets | Analgesic, ASA |
| Zantac | Yes, if ulcer disease pain-free. | Antacid |
| Zarontin | Yes. | Anticonvulsant |
| Zaroxolyn | Yes. | Diuretic, antihypertensive |
| Zebeta | Yes | Beta Blocker |
| Zemuron | Drug OK, but shuldn’t donate for 6 weeks after surgery. | Non-depolarizing muscle relaxant |
| Zephrex | Yes, if taken for allergies. Defer for 72 hours after symptoms are resolved if taken for cold/flu symptoms. | Decongestant, expectorant. |
| Zeste | Yes. | Hormone |
| Zestoretic | Yes, if for hypertension. | Antihypertensive |
| Zestril | Yes, if for hypertension. | Antihypertensive |
| Ziac | Yes | Antihypertensive |
| **Zidovudine** | **Permanent deferral.** | **Anti-HIV agent** |
| Zinacef | No, defer 24 hours after course completed, if IV or IM defer 1 week. | Antibiotic |
| Zithromax | No, defer 24 hours after course completed. Yes for acne. | Antibiotic |
| Zocor | Yes. | Cholesterol lowering agent. |
| Zoladex | Accept if taken for endometriosis. Defer if taken for cancer | Gonadotropin secretion inhibitor. |
| Zoloft | Yes. | Antidepressant |
| **Zonegran (Zonisamide)** | **No. Defer for 1 month after last dose. (Possible Fetal Tetragenicity)** | **Anti-seizure medication** |
| Zorane | Yes. | Oral contraceptive |
| Zorprin (Boots) | Yes, if taken for allergies. Defer for 72 hours after symptoms are resolved if taken for cold/flu symptoms or for fever.  Defer 72 hrs for plateletpheresis or sole source platelets | ASA containing analgesic |
| Zovirax | Yes. | Anti-herpes medication |
| Zyban | Yes. | Anti smoking aid |
| Zyloprim | Yes. | Uricosuric |
| Zymase | Yes | For pancreatic enzyme deficiency |
| Zyprexa | Yes. | Antipsychotic |
| Zyrtec | Yes, if taken for allergies. Defer for 72 hours after symptoms are resolved if taken for cold/flu symptoms. | Antihistamine |
