# Supplementary material for: Derivation of Xeno-Free and GMP-Grade Human Embryonic Stem Cells – Platforms for Future Clinical Applications
Source: PLoS One. 2012 Jun 20;7(6):e35325. doi: 10.1371/journal.pone.0035325 (PMC3380026; doi:10.1371/journal.pone.0035325)
Supplement: File S32 — Appendix 4, Malaria & vCJD Risk Countries. (DOC) [file pone.0035325.s046.doc]

**MALARIA/vCJD RISK COUNTRY LIST**

| Country | Malaria Risk | vCJD Risk | Note |
| --- | --- | --- | --- |
| Afghanistan | Yes* | No | Malaria: Risk in all areas. |
| Albania | No | *Yes* | **vCJD:**  (a) Defer DoD affiliated personnel (anyone with access to a DoD commissary in Europe) if they resided in the country for a cumulative period of 6 months or more from 1980-1996. For DoD affiliated personnel who resided in the country after 1 Jan 1997, (b) applies.  (b) Defer any donor that resided in the country for a cumulative period of 5 years or more from 1980 to present. |
| Algeria | Yes | No | **Malaria**: risk is limited to Ihrir (Illizi Department) |
| Andorra | No | *Yes* | **vCJD:**  (a) Defer DoD affiliated personnel (anyone with access to a DoD commissary in Europe) if they resided in the country for a cumulative period of 6 months or more from 1980-1996. For DoD affiliated personnel who resided in the country after 1 Jan 1997, (b) applies.  (b) Defer any donor that resided in the country for a cumulative period of 5 years or more from 1980 to present. |
| Angola | Yes | No | Malaria: Risk in all areas. |
| Anguilla | No | No |  |
| Antigua and Barbuda | No | No |  |
| Argentina | Yes | No | **Malaria:** Risk in rural areas near Bolivian border (Salta and Jujuy Provinces) and along border with Paraguay (Misiones and Corrientes Provinces). |
| Armenia | Yes | No | **Malaria:** Risk limited to western border areas: Masis, Ararat, and Artashat regions in Ararat District. |
| Australia | No | No |  |
| Austria | No | *Yes* | **vCJD:**  (a) Defer DoD affiliated personnel (anyone with access to a DoD commissary in Europe) if they resided in the country for a cumulative period of 6 months or more from 1980-1996. For DoD affiliated personnel who resided in the country after 1 Jan 1997, (b) applies.  (b) Defer any donor that resided in the country for a cumulative period of 5 years or more from 1980 to present. |
| Azerbaijan | Yes* | No | Malaria: Risk in all areas. |
| Azores (Portugal) | No | *Yes* | **vCJD:**  (a) Defer DoD affiliated personnel (anyone with access to a DoD commissary in Europe) if they resided in the country for a cumulative period of 6 months or more from 1980-1996. For DoD affiliated personnel who resided in the country after 1 Jan 1997, (b) applies.  (b) Defer any donor that resided in the country for a cumulative period of 5 years or more from 1980 to present. |
| Bahamas | No | No |  |
| Bahrain | No | No |  |
| Bangladesh | Yes | No | **Malaria:** All areas except no risk in city of Dhaka. |
| Barbados | No | No |  |
| Belarus | No | No |  |
| Belgium | No | *Yes* | **vCJD:**  (a) Defer DoD affiliated personnel (anyone with access to a DoD commissary in Europe) if they resided in the country for a cumulative period of 6 months or more from 1980-1996. For DoD affiliated personnel who resided in the country after 1 Jan 1997, (b) applies.  (b) Defer any donor that resided in the country for a cumulative period of 5 years or more from 1980 to present. |
| Belize (Br. Honduras) | Yes | No | **Malaria:** All except no risk in Belize City. |
| Benin (Dahomey) | Yes | No | Malaria: Risk in all areas. |
| Bermuda (U.K.) | No | No |  |
| Bhutan | Yes | No | **Malaria**: Rural areas in districts bordering India. |
| Bolivia | Yes | No | **Malaria:** Risk in the following departments: Beni, Cochabamba, Chuquisaca, La Paz, Pando, Santa Cruz, and Tarija. |
| Bosnia/Herzogovina | No | *Yes* | **vCJD:**  (a) Defer DoD affiliated personnel (anyone with access to a DoD commissary in Europe) if they resided in the country for a cumulative period of 6 months or more from 1980-1996. For DoD affiliated personnel who resided in the country after 1 Jan 1997, (b) applies.  (b) Defer any donor that resided in the country for a cumulative period of 5 years or more from 1980 to present. |
| Botswana | Yes | No | **Malaria:** Risk in northern part of country (north of 21ºS). |
| Br. Honduras (see Belize) |  |  | See Belize |
| Brazil | Yes | No | **Malaria:** Risk in States of Acre, Rondonia, Amapa, Amazonas, Roraima, Tocantins, Maranhao, Mato Grosso, and Para. These is also risk in urban areas, including large cities such as Porto Velho, Boa Vista, Macapa, Manaus, Santarem, and Maraba. The costal states form the “horn” south to the Uruguay border, including Iguassu Falls are not risk areas. |
| Brunei Darussalam | No | No |  |
| Bulgaria | No | Yes | **vCJD:**  (a) Defer DoD affiliated personnel (anyone with access to a DoD commissary in Europe) if they resided in the country for a cumulative period of 6 months or more from 1980-1996. For DoD affiliated personnel who resided in the country after 1 Jan 1997, (b) applies.  (b) Defer any donor that resided in the country for a cumulative period of 5 years or more from 1980 to present. |
| Burkina Faso (Upper Volta) | Yes | No | Malaria: Risk in all areas. |
| Burma (see Myanmar) |  |  | See Myanmar |
| Burundi | Yes | No | Malaria: Risk in all areas. |
| Cambodia | Yes | No | **Malaria:** All areas, except no risk in Phom Penh. There is risk at the temple complex at Angkor Wat. |
| Cameroon | Yes | No | Malaria: Risk in all areas. |
| Canada | No | No |  |
| Canary Islands (Spain) | No | *Yes* | **vCJD:**  (a) Defer DoD affiliated personnel (anyone with access to a DoD commissary in Europe) if they resided in the country for a cumulative period of 6 months or more from 1980-1996. For DoD affiliated personnel who resided in the country after 1 Jan 1997, (b) applies.  (b) Defer any donor that resided in the country for a cumulative period of 5 years or more from 1980 to present. |
| Cape Verde Islands | Yes | No | **Malaria:** Risk limited to Island of São Tiago |
| Cayman Islands (U.K.) | No | No |  |
| Central African Republic | Yes | No | Malaria: Risk in all areas. |
| Chad | Yes | No | Malaria: Risk in all areas. |
| Channel Islands (U.K.) | No | *Yes* | **vCJD**:  (a) Defer if donor has spent a cumulative time of three months or more from 1980 through 1996 anywhere in the U.K. *For DoD affiliated personnel and all other donors who only resided in the country after 1 Jan 1997, donors are not deferred.*  (b) Defer if donor received a transfusion in the U.K. since 1980. |
| Chile | No | No |  |
| China | Yes | No | **Malaria:** Rural areas only of the following provinces: Hainan, Yunnan, Fuijan, Guangdong, Guangxi, Guizhou, Sichuan, Xizang (in the Zangbo River Valley only), Anhui, Hubei, Hunan, Jiangsu, Jiangxi, Shadong, Shanghai, Zhejiang. In provinces with risk, transmission only occurs during warm weather:  North of latitude 33° N, transmission occurs July to November; from latitude 33° N to 25° N, transmission occurs May to December; south of latitude 25° N, transmission occurs year-round.  Note: Travelers to cities and popular tourist areas, including Yangtze River cruises, are not at risk. |
| China Hong Kong S.A.R. | Yes | No | **Malaria:** Risk in rural areas |
| China Macao S.A.R. | No | No |  |
| Christmas Island (Australia) | No | No |  |
| Colombia | Yes | No | **Malaria:** Risk in all rural areas except no risk in Bogota and vicinity. |
| Comoros | Yes | No | Malaria: Risk in all areas. |
| Congo | Yes | No | Malaria: Risk in all areas. |
| Cook Island (New Zealand) | No | No |  |
| Costa Rica | Yes | No | **Malaria:** Risk limited to provinces of Alujuela, Limon, Guanacaste, and Heredia. |
| Côte d'Ivoire (Ivory Coast) | Yes | No | Malaria: Risk in all areas. |
| Croatia | No | *Yes* | **vCJD:**  (a) Defer DoD affiliated personnel (anyone with access to a DoD commissary in Europe) if they resided in the country for a cumulative period of 6 months or more from 1980-1996. For DoD affiliated personnel who resided in the country after 1 Jan 1997, (b) applies.  (b) Defer any donor that resided in the country for a cumulative period of 5 years or more from 1980 to present. |
| Cuba | No | No |  |
| Cyprus | No | No |  |
| Czech Republic | No | *Yes* | **vCJD:**  (a) Defer DoD affiliated personnel (anyone with access to a DoD commissary in Europe) if they resided in the country for a cumulative period of 6 months or more from 1980-1996. For DoD affiliated personnel who resided in the country after 1 Jan 1997, (b) applies.  (b) Defer any donor that resided in the country for a cumulative period of 5 years or more from 1980 to present. |
| Dahomey (see Benin) |  |  |  |
| Denmark | No | *Yes* | **vCJD:**  (a) Defer DoD affiliated personnel (anyone with access to a DoD commissary in Europe) if they resided in the country for a cumulative period of 6 months or more from 1980-1996. For DoD affiliated personnel who resided in the country after 1 Jan 1997, (b) applies.  (b) Defer any donor that resided in the country for a cumulative period of 5 years or more from 1980 to present. |
| Diego Garcia | No | No |  |
| Djibouti | Yes | No | Malaria: Risk in all areas. |
| Dominica | No | No |  |
| Dominican Republic | Yes* | No | Malaria: Risk in all areas. |
| Democratic Republic of Congo |  |  | See Zaire |
| East Timor | Yes | No | Malaria: Risk in all areas. |
| Ecuador | Yes | No | **Malaria:** Risk in all areas except Guanyaquil, Quito, the central highland tourist areas, and the Galapogos Islands. |
| Egypt | Yes | No | **Malaria:** Very limited risk in El Faiyum area only. Travelers visiting main tourist areas, including Nile River cruises are not at risk. |
| El Salvador | Yes | No | **Malaria:** Rural areas of Santa Ana, Ahuachapan, and La Union only. |
| England (U.K.) | No | *Yes* | **vCJD**:  (a) Defer if donor has spent a cumulative time of three months or more from 1980 through 1996 anywhere in the U.K. *For DoD affiliated personnel and all other donors who only resided in the country after 1 Jan 1997, donors are not deferred.*  (b) Defer if donor received a transfusion in the U.K. since 1980. |
| Equatorial Guinea | Yes | No | Malaria: Risk in all areas. |
| Eritrea | Yes* | No | **Malaria:** All areas at risk except no risk in Asmara. |
| Estonia | No | No |  |
| Ethiopia | Yes* | No | **Malaria:** All areas except no risk in Addis Ababa. |
| Falkland Islands (U.K.) | No | *Yes* | **vCJD**:  (a) Defer if donor has spent a cumulative time of three months or more from 1980 through 1996 anywhere in the U.K. *For DoD affiliated personnel and all other donors who only resided in the country after 1 Jan 1997, donors are not deferred.*  (b) Defer if donor received a transfusion in the U.K. since 1980. |
| Faroe Islands (Denmark) | No | *Yes* | **vCJD:**  (a) Defer DoD affiliated personnel (anyone with access to a DoD commissary in Europe) if they resided in the country for a cumulative period of 6 months or more from 1980-1996. For DoD affiliated personnel who resided in the country after 1 Jan 1997, (b) applies.  (b) Defer any donor that resided in the country for a cumulative period of 5 years or more from 1980 to present. |
| Fiji | No | No |  |
| Finland | No | *Yes* | **vCJD:**  (a) Defer DoD affiliated personnel (anyone with access to a DoD commissary in Europe) if they resided in the country for a cumulative period of 6 months or more from 1980-1996. For DoD affiliated personnel who resided in the country after 1 Jan 1997, (b) applies.  (b) Defer any donor that resided in the country for a cumulative period of 5 years or more from 1980 to present. |
| France | No | *Yes* | **vCJD:**  (a) Defer DoD affiliated personnel (anyone with access to a DoD commissary in Europe) if they resided in the country for a cumulative period of 6 months or more from 1980-1996. For DoD affiliated personnel who resided in the country after 1 Jan 1997, (b) applies.  (b) Defer any donor that resided in the country for a cumulative period of 5 years or more from 1980 to present. |
| French Guiana | Yes | No | Malaria: Risk in all areas. |
| French Polynesia (Tahiti, Moorea Bora-Bora, Marquesas, Hiva Oa, Ua huka, Austral Islands, Tubvai, and Rurutu) | No | No |  |
| Gabon | Yes | No | Malaria: Risk in all areas. |
| Gambia | Yes | No | Malaria: Risk in all areas. |
| Georgia | Yes | No | **Malaria:** Risk in Southestern part of the country, in the Kakheti and Kveno Kartli regions. |
| Germany | No | *Yes* | **vCJD:**  (a) Defer DoD affiliated personnel (anyone with access to a DoD commissary in Europe) if they resided in the country for a cumulative period of 6 months or more from 1980-1996. For DoD affiliated personnel who resided in the country after 1 Jan 1997, (b) applies.  (b) Defer any donor that resided in the country for a cumulative period of 5 years or more from 1980 to present. |
| Ghana | Yes | No | Malaria: Risk in all areas. |
| Gibraltar (U.K.) | No | *Yes* | **vCJD**:  (a) Defer if donor has spent a cumulative time of three months or more from 1980 through 1996 anywhere in the U.K. For DoD affiliated personnel and all other donors who only resided in the country after 1 Jan 1997, donors are not deferred.  (b) Defer if donor received a transfusion in the U.K. since 1980. |
| Gilbert Islands (see Kiribati) |  |  | See Kiribati |
| Greece | No | *Yes* | **vCJD:**  (a) Defer DoD affiliated personnel (anyone with access to a DoD commissary in Europe) if they resided in the country for a cumulative period of 6 months or more from 1980-1996. For DoD affiliated personnel who resided in the country after 1 Jan 1997, (b) applies.  (b) Defer any donor that resided in the country for a cumulative period of 5 years or more from 1980 to present. |
| Greenland (Denmark) | No | *Yes*** | **vCJD:**  (a) Defer DoD affiliated personnel (anyone with access to a DoD commissary in Europe) if they resided in the country for a cumulative period of 6 months or more from 1980-1996. For DoD affiliated personnel who resided in the country after 1 Jan 1997, (b) applies.  (b) Defer any donor that resided in the country for a cumulative period of 5 years or more from 1980 to present. |
| Grenada | No | No |  |
| Guadeloupe (France) | No | No |  |
| Guam (U.S.) | No | No |  |
| Guatemala | Yes* | No | **Malaria:** Rural areas only. |
| Guinea | Yes | No | Malaria: Risk in all areas. |
| Guinea-Bissau | Yes | No | Malaria: Risk in all areas. |
| Guyana | Yes* | No | **Malaria:** Risk in all areas. |
| Haiti | Yes | No | Malaria: Risk in all areas. |
| Honduras | Yes | No | **Malaria:** Rural areas only, including Roatan and other Bay Islands. |
| Hungary | No | *Yes* | **vCJD:**  (a) Defer DoD affiliated personnel (anyone with access to a DoD commissary in Europe) if they resided in the country for a cumulative period of 6 months or more from 1980-1996. For DoD affiliated personnel who resided in the country after 1 Jan 1997, (b) applies.  (b) Defer any donor that resided in the country for a cumulative period of 5 years or more from 1980 to present. |
| Iceland | No | *Yes*** | **vCJD:** Defer DoD affiliated personnel (anyone with access to a DoD commissary in Europe) if they resided in the country for a cumulative period of 6 months or more from 1980-1996.  For DoD affiliated personnel and all other donors who only resided in the country after 1 Jan 1997, donors are not deferred. |
| India | Yes* | No | Malaria: Risk in all areas. |
| Indonesia | Yes | No | **Malaria:** Rural only, except high risk in all areas of Irian Jaya (western half of island of New Guinea). No risk in cities of Java and Sumatra and no risk for the main resort areas of Java, Sumatra, and Bali. Note: There is malaria risk at the temple complex of Borobudur. |
| Iran, Islamic Republic of | Yes | No | **Malaria:** Risk in rural areas only in the provinces of Sistan-Baluchestan, the tropical part of Kerman, Hormozgan, and parts of Bushehr, Fars, Ilam, Kohgiluyeh-Boyar, Lorestan, and Chahar Mahal-Bakhtiari, and the north of Khuzestan. |
| Iraq | Yes | No | **Malaria:** All of northern region; provinces of Duhok, Erbil, Ninawa, Sulaimaniya, Támim, Basrah |
| Ireland, Republic of | No | *Yes* | **vCJD:**  (a) Defer DoD affiliated personnel (anyone with access to a DoD commissary in Europe) if they resided in the country for a cumulative period of 6 months or more from 1980-1996. For DoD affiliated personnel who resided in the country after 1 Jan 1997, (b) applies.  (b) Defer any donor that resided in the country for a cumulative period of 5 years or more from 1980 to present. |
| Isle of Man (U.K.) | No | *Yes* | **vCJD**:  (a) Defer if donor has spent a cumulative time of three months or more from 1980 through 1996 anywhere in the U.K. For DoD affiliated personnel and all other donors who only resided in the country after 1 Jan 1997, donors are not deferred.  (b) Defer if donor received a transfusion in the U.K. since 1980. |
| Israel | No | No |  |
| Italy | No | *Yes* | **vCJD:**  (a) Defer DoD affiliated personnel (anyone with access to a DoD commissary in Europe) if they resided in the country for a cumulative period of 6 months or more from 1980-1996. For DoD affiliated personnel who resided in the country after 1 Jan 1997, (b) applies.  (b) Defer any donor that resided in the country for a cumulative period of 5 years or more from 1980 to present. |
| Jamaica | No | No |  |
| Japan | No | No |  |
| Johnston Atoll | No | No |  |
| Jordan | No | No |  |
| Kampuchea, Democratic  (see Cambodia) |  |  | See Cambodia |
| Kazakhstan | No | No |  |
| Kenya | Yes | No | **Malaria:** All areas (including game parks) except no risk in Nairobi. |
| Kiribati (Tarawa, Tabuaeran, Fanning, Kiritimati, Christmas, Banaba, and Ocean Island. | No | No |  |
| Korea, Democratic People's Republic of (North) | Yes* | No | **Malaria:** All areas  Note: Defer travelers for **24** **months**. |
| Korea, Republic of (South) | Yes* | No | **Malaria:** No risk in Seoul or areas south of Seoul (south of 37.7^o^ North)  Note: Defer travelers north of Seoul for **24** **months**. |
| Kosovo | No | *Yes* | **vCJD:**  (a) Defer DoD affiliated personnel (anyone with access to a DoD commissary in Europe) if they resided in the country for a cumulative period of 6 months or more from 1980-1996. For DoD affiliated personnel who resided in the country after 1 Jan 1997, (b) applies.  (b) Defer any donor that resided in the country for a cumulative period of 5 years or more from 1980 to present. |
| Kuwait | No | No |  |
| Kyrgyzstan | No | No |  |
| Lao People's Democratic Republic | Yes | No | **Malaria:** All areas, except no risk in city of Vientiane. |
| Latvia | No | No |  |
| Lebanon | No | No |  |
| Lesotho | No | No |  |
| Liberia | Yes | No | Malaria: Risk in all areas. |
| Libyan Arab Jamahiriya | No | No |  |
| Liechtenstein | No | *Yes* | **vCJD:**  (a) Defer DoD affiliated personnel (anyone with access to a DoD commissary in Europe) if they resided in the country for a cumulative period of 6 months or more from 1980-1996. For DoD affiliated personnel who resided in the country after 1 Jan 1997, (b) applies.  (b) Defer any donor that resided in the country for a cumulative period of 5 years or more from 1980 to present. |
| Lithuania | No | No |  |
| Luxembourg | No | *Yes* | **vCJD:**  (a) Defer DoD affiliated personnel (anyone with access to a DoD commissary in Europe) if they resided in the country for a cumulative period of 6 months or more from 1980-1996. For DoD affiliated personnel who resided in the country after 1 Jan 1997, (b) applies.  (b) Defer any donor that resided in the country for a cumulative period of 5 years or more from 1980 to present. |
| Macao (Portugal) | No | No |  |
| Macedonia, Former Yugoslav Republic of | No | *Yes* | **vCJD:**  (a) Defer DoD affiliated personnel (anyone with access to a DoD commissary in Europe) if they resided in the country for a cumulative period of 6 months or more from 1980-1996. For DoD affiliated personnel who resided in the country after 1 Jan 1997, (b) applies.  (b) Defer any donor that resided in the country for a cumulative period of 5 years or more from 1980 to present. |
| Madagascar | Yes | No | **Malaria:** Risk in all areas. |
| Madeira (Portugal) | No | *Yes* | **vCJD:**  (a) Defer DoD affiliated personnel (anyone with access to a DoD commissary in Europe) if they resided in the country for a cumulative period of 6 months or more from 1980-1996. For DoD affiliated personnel who resided in the country after 1 Jan 1997, (b) applies.  (b) Defer any donor that resided in the country for a cumulative period of 5 years or more from 1980 to present. |
| Malawi | Yes | No | Malaria: Risk in all areas. |
| Malaysia | Yes | No | **Malaria:** remote areas of peninsular Malaysia and Sarawak (NW Borneo). Urban and Costal areas are risk free. Sabah (NE Borneo) has risk throughout. |
| Maldives | No | No |  |
| Mali | Yes | No | Malaria: Risk in all areas. |
| Malta | No | No |  |
| Marshall Islands | No | No |  |
| Martinique (France) | No | No |  |
| Mauritania | Yes | No | **Malaria:** Risk in all areas, except no risk in northern region of Dakhlet-Nouadhibou, and Tiris-Zemour. |
| Mauritius | Yes | No | **Malaria:** Rural areas only, except no risk on Rodriguez Island. |
| Mayotte (French territorial collectivity) | Yes | No | Malaria: Risk in all areas. |
| Mexico | Yes | No | **Malaria:** No malaria risk exists along the United States-Mexico border. No malaria risk exists in the major resorts along the Pacific and Gulf Coasts. Risk in rural areas, including resorts in rural areas of the following states: Campeche, Chiapas, Guerrero, Michoahan, Nayarit, Oaxaca, Quintana Roo and Playa del Carmen (include bus trips immediately outside of Cacun and Cozumel), Sinaloa, and Tabasco. In addition, risk exists in the state of Jalisco (in its mountainous northern area only). Risk also exists in an area between 24N and 28N latitude and 106W and 110W longitude. This area is rarely visited by tourists. This area includes parts of the states of Sonora, Chihuahua, and Durango. |
| Micronesia (Yap Islands, pompei, Chuuk, and Kosrae) | No | No |  |
| Midway Island (U.S.) | No | No |  |
| Moldova | No | No |  |
| Monaco | No | *Yes* | **vCJD:**  (a) Defer DoD affiliated personnel (anyone with access to a DoD commissary in Europe) if they resided in the country for a cumulative period of 6 months or more from 1980-1996. For DoD affiliated personnel who resided in the country after 1 Jan 1997, (b) applies.  (b) Defer any donor that resided in the country for a cumulative period of 5 years or more from 1980 to present. |
| Mongolia | No | No |  |
| Montenegro | No | *Yes* | **vCJD:**  (a) Defer DoD affiliated personnel (anyone with access to a DoD commissary in Europe) if they resided in the country for a cumulative period of 6 months or more from 1980-1996. For DoD affiliated personnel who resided in the country after 1 Jan 1997, (b) applies.  (b) Defer any donor that resided in the country for a cumulative period of 5 years or more from 1980 to present. |
| Montserrat (U.K.) | No | No |  |
| Morocco | Yes | No | **Malaria:** limited risk in rural areas of Khouribga Province. The cities of Tangier, Rabat, Casablanca, Marrakech, and Fes do not has risk. |
| Mozambique | Yes | No | Malaria: Risk in all areas. |
| Myanmar (formerly Burma) | Yes | No | **Malaria:** Rural areas only. The cities of Yangon (formerly Rangoon) and Mandalay are not at risk. |
| Namibia | Yes | No | **Malaria:** Risk in the northern regions and in Omahake and Otjozondjupa and along the Kavango and Kunene rivers. |
| Nauru | No | No |  |
| Nepal | Yes* | No | **Malaria:** Risk in all areas except no risk in Katmandu. |
| Netherlands | No | *Yes* | **vCJD:**  (a) Defer DoD affiliated personnel (anyone with access to a DoD commissary in Europe) if they resided in the country for a cumulative period of 6 months or more from 1980-1996. For DoD affiliated personnel who resided in the country after 1 Jan 1997, (b) applies.  (b) Defer any donor that resided in the country for a cumulative period of 5 years or more from 1980 to present. |
| Netherlands Antilles | No | No |  |
| New Caledonia and Dependencies (France) | No | No |  |
| New Zealand | No | No |  |
| Nicaragua | Yes | No | **Malaria:** Rural areas only; however, risk exists in outskirts of Managua. |
| Niger | Yes | No | Malaria: Risk in all areas. |
| Nigeria | Yes | No | Malaria: Risk in all areas. |
| Niue (New Zealand) | No | No |  |
| Northern Ireland (U.K.) | No | *Yes* | **vCJD**:  (a) Defer if donor has spent a cumulative time of three months or more from 1980 through 1996 anywhere in the U.K. For DoD affiliated personnel and all other donors who only resided in the country after 1 Jan 1997, donors are not deferred.  (b) Defer if donor received a transfusion in the U.K. since 1980. |
| Northern Mariana Islands (Saipan, Tinian, and Rota Island) | No | No |  |
| Norway | No | Yes | **vCJD:**  (a) Defer DoD affiliated personnel (anyone with access to a DoD commissary in Europe) if they resided in the country for a cumulative period of 6 months or more from 1980-1996. For DoD affiliated personnel who resided in the country after 1 Jan 1997, (b) applies.  (b) Defer any donor that resided in the country for a cumulative period of 5 years or more from 1980 to present. |
| Oman | Yes | **Yes** | **Malaria: Limited risk in remote areas of Musandam Province**  **vCJD:**  **(a) Defer DoD affiliated personnel (anyone with access to a DoD commissary in Europe) if they resided in the country for a cumulative period of 6 months or more from 1980-1996. For DoD affiliated personnel who resided in the country after 1 Jan 1997, (b) applies.**  **(b) Defer any donor that resided in the country for a cumulative period of 5 years or more from 1980 to present.** |
| Pacific Islands, Trust Territory of the U.S.A. (Guam, American Samoa, Johnston Atoll, Wake Island, and Midway Islands) | No | No |  |
| Pakistan | Yes* | No | Malaria: Risk in all areas. |
| Palau | No | No |  |
| Panama | Yes | No | **Malaria**: Risk in rural areas of three provinces: Bocas del Toro, Darien, and San Blas. There is no risk in the Canal Zone or in Panama City and vicinity. |
| Papua New Guinea | Yes | No | Malaria: Risk in all areas. |
| Paraguay | Yes | No | **Malaria:** Risk in 3 Departments – Alto Parana, Caaguazu, and Canendiyu. |
| Peru | Yes | No | **Malaria:** Risk in all departments except: Arequipa, Moquegua, Puno, and Tacna. No risk in Lima and vicinity, Costal regions south of Lima, or highland tourist areas (Cuzco, Machu Piccu or Lake Titicaca). |
| Philippines | Yes | No | **Malaria:** Rural areas only except there is no risk in Provinces of Bohol, Catanduanes, Cebu, and metropolitan Manila. **Subic Bay is a risk area**. |
| Pitcairn (U.K.) | No | No |  |
| Poland | No | *Yes* | **vCJD:**  (a) Defer DoD affiliated personnel (anyone with access to a DoD commissary in Europe) if they resided in the country for a cumulative period of 6 months or more from 1980-1996. For DoD affiliated personnel who resided in the country after 1 Jan 1997, (b) applies.  (b) Defer any donor that resided in the country for a cumulative period of 5 years or more from 1980 to present. |
| Portugal | No | *Yes* | **vCJD:**  (a) Defer DoD affiliated personnel (anyone with access to a DoD commissary in Europe) if they resided in the country for a cumulative period of 6 months or more from 1980-1996. For DoD affiliated personnel who resided in the country after 1 Jan 1997, (b) applies.  (b) Defer any donor that resided in the country for a cumulative period of 5 years or more from 1980 to present. |
| Puerto Rico (U.S.) | No | No |  |
| Qatar | No | No |  |
| Republic of Ireland | No | *Yes* | **vCJD:**  (a) Defer DoD affiliated personnel (anyone with access to a DoD commissary in Europe) if they resided in the country for a cumulative period of 6 months or more from 1980-1996. For DoD affiliated personnel who resided in the country after 1 Jan 1997, (b) applies.  (b) Defer any donor that resided in the country for a cumulative period of 5 years or more from 1980 to present. |
| Republic of Moldavia | No | No |  |
| Reunion (France) | No | No |  |
| Romania | No | *Yes* | **vCJD:**  (a) Defer DoD affiliated personnel (anyone with access to a DoD commissary in Europe) if they resided in the country for a cumulative period of 6 months or more from 1980-1996. For DoD affiliated personnel who resided in the country after 1 Jan 1997, (b) applies.  (b) Defer any donor that resided in the country for a cumulative period of 5 years or more from 1980 to present. |
| Russian Federation | No | No |  |
| Rwanda | Yes | No | Malaria: Risk in all areas. |
| Saint Christopher (Saint Kitts) and Nevis (U.K.) | No | No |  |
| Saint Helena (U.K.) | No | No |  |
| Saint Lucia | No | No |  |
| Saint Pierre & Miquelon (France) | No | No |  |
| Saint Vincent and the Grenadines | No | No |  |
| Samoa (formerly Western Samoa) | No | No |  |
| Samoa, American (U.S.) | No | No |  |
| San Marino | No | *Yes* | **vCJD:**  (a) Defer DoD affiliated personnel (anyone with access to a DoD commissary in Europe) if they resided in the country for a cumulative period of 6 months or more from 1980-1996. For DoD affiliated personnel who resided in the country after 1 Jan 1997, (b) applies.  (b) Defer any donor that resided in the country for a cumulative period of 5 years or more from 1980 to present. |
| Sao Tome & Principe | Yes | No | Malaria: Risk in all areas. |
| Saudi Arabia | Yes | No | **Malaria:** All areas in western provinces except no risk in the high altitude areas of Asir Province (Yemen border) and the urban areas of Jeddah, Mecca, Medina, and Taif (Prince Sultan Airbase). |
| Scotland (U.K.) | No | *Yes* | **vCJD**:  (a) Defer if donor has spent a cumulative time of three months or more from 1980 through 1996 anywhere in the U.K. For DoD affiliated personnel and all other donors who only resided in the country after 1 Jan 1997, donors are not deferred.  (b) Defer if donor received a transfusion in the U.K. since 1980. |
| Senegal | Yes | No | Malaria: Risk in all areas. |
| Serbia | No | *Yes* | **vCJD:**  (a) Defer DoD affiliated personnel (anyone with access to a DoD commissary in Europe) if they resided in the country for a cumulative period of 6 months or more from 1980-1996. For DoD affiliated personnel who resided in the country after 1 Jan 1997, (b) applies.  (b) Defer any donor that resided in the country for a cumulative period of 5 years or more from 1980 to present. |
| Seychelles | No | No |  |
| Sierra Leone | Yes | No | Malaria: Risk in all areas. |
| Singapore | No | No |  |
| Slovak Republic | No | *Yes* | **vCJD:**  (a) Defer DoD affiliated personnel (anyone with access to a DoD commissary in Europe) if they resided in the country for a cumulative period of 6 months or more from 1980-1996. For DoD affiliated personnel who resided in the country after 1 Jan 1997, (b) applies.  (b) Defer any donor that resided in the country for a cumulative period of 5 years or more from 1980 to present. |
| Slovenia | No | *Yes* | **vCJD:**  (a) Defer DoD affiliated personnel (anyone with access to a DoD commissary in Europe) if they resided in the country for a cumulative period of 6 months or more from 1980-1996. For DoD affiliated personnel who resided in the country after 1 Jan 1997, (b) applies.  (b) Defer any donor that resided in the country for a cumulative period of 5 years or more from 1980 to present. |
| Solomon Islands | Yes | No | Malaria: Risk in all areas. |
| Somalia | Yes | No | Malaria: Risk in all areas. |
| South Africa | Yes | No | **Malaria:** Risk exists in the low altitude areas of the Mpumalanga Province (including Kruger National Park), Northern Privince, and northestern KwaZuluNatal as faar south as the Tugula River. |
| Spain | No | *Yes* | **vCJD:**  (a) Defer DoD affiliated personnel (anyone with access to a DoD commissary in Europe) if they resided in the country for a cumulative period of 6 months or more from 1980-1996. For DoD affiliated personnel who resided in the country after 1 Jan 1997, (b) applies.  (b) Defer any donor that resided in the country for a cumulative period of 5 years or more from 1980 to present. |
| Sri Lanka (Ceylon) | Yes | No | **Malaria:** Risk in all rural areas. No risk in the districts of Colombo, Kalutara, and Nuwara Eliya. |
| Sudan | Yes | No | Malaria: Risk in all areas. |
| Suriname | Yes | No | **Malaria:** Risk in rural areas only, except no risk in Paramaribo District and coastal areas north of 5º N. |
| Svalbard (Norway) | No | *Yes* | **vCJD:**  (a) Defer DoD affiliated personnel (anyone with access to a DoD commissary in Europe) if they resided in the country for a cumulative period of 6 months or more from 1980-1996. For DoD affiliated personnel who resided in the country after 1 Jan 1997, (b) applies.  (b) Defer any donor that resided in the country for a cumulative period of 5 years or more from 1980 to present. |
| Swaziland | Yes* | No | Malaria: Risk in all areas. |
| Sweden | No | *Yes* | **vCJD:**  (a) Defer DoD affiliated personnel (anyone with access to a DoD commissary in Europe) if they resided in the country for a cumulative period of 6 months or more from 1980-1996. For DoD affiliated personnel who resided in the country after 1 Jan 1997, (b) applies.  (b) Defer any donor that resided in the country for a cumulative period of 5 years or more from 1980 to present. |
| Switzerland | No | *Yes* | **vCJD:**  (a) Defer DoD affiliated personnel (anyone with access to a DoD commissary in Europe) if they resided in the country for a cumulative period of 6 months or more from 1980-1996. For DoD affiliated personnel who resided in the country after 1 Jan 1997, (b) applies.  (b) Defer any donor that resided in the country for a cumulative period of 5 years or more from 1980 to present. |
| Syrian Arab Republic | Yes | No | **Malaria:** Risk in rural areas only, except no risk in southern and western Districts of Deir-es-zor-and Sweida. |
| Tahiti | No | No |  |
| Taiwan | No | No |  |
| Tajikistan | Yes* | No | **Malaria:** Risk in all areas. |
| Tanzania, United Rep. | Yes | No | **Malaria:** Risk in all areas. |
| Thailand | Yes | No | **Malaria:** Limited risk in the areas that border Cambodia, Laos and Burma. No risk in cities and major tourist resorts (Bangkok, Chiang Mai, Chiang Rai, Pattaya, Phuket Island, and Ko Sumui). |
| Timor | Yes | No | Malaria: Risk in all areas. |
| Togo | Yes | No | Malaria: Risk in all areas. |
| Tokelau (New Zealand) | No | No |  |
| Tonga | No | No |  |
| Trinidad and Tobago | No | No |  |
| Tunisia | No | No |  |
| Turkey | Yes* | **Yes**** | *Malaria: Risk in all areas in eastern half of the country (including Incerlik and Adana.)  ****vCJD:**  **(a) Defer DoD affiliated personnel (anyone with access to a DoD commissary in Europe) if they resided in the country for cumulative period of 6 months or more from 1980-1996. For DoD affiliated personnel and all other donors who only resided in the country after 1 Jan 1997, donors are not deferred.** |
| Turkmenistan | Yes | No | **Malaria:** Risk in some villages in Mary, Lebap, and Balkan districts. |
| Tuvalu | No | No |  |
| Uganda | Yes | No | Malaria: Risk in all areas. |
| Ukraine | No | No |  |
| Union of Soviet Socialist Republics (former) |  |  | See individual countries |
| United Arab Emirates | Yes | No | **Malaria:** Very limited risk in the foothill areas and valleys in the mountainous regions of the northern Emirates bordering Oman’s Musandam Province. No risk in Abu Dhabi or in cities of Ajman, Dubai, Sharjah, and Umm al Qaiwan. |
| United Kingdom (U.K.) (England, Scotland, Wales, Northern Ireland, Channel Islands, Gibraltar, Falkland Islands and the Isle of Man) | No | *Yes* | **vCJD**:  (a) Defer if donor has spent a cumulative time of three months or more from 1980 through 1996 anywhere in the U.K. For DoD affiliated personnel and all other donors who only resided in the country after 1 Jan 1997, donors are not deferred.  (b) Defer if donor received a transfusion in the U.K. since 1980. |
| United States of America | No | No |  |
| Upper Volta (see Burkina Faso) |  |  | See Burkina Faso |
| Uruguay | No | No |  |
| Uzbekistan | No | No |  |
| Vanuatu (New Hebrides) | Yes | No | **Malaria:** Risk in all areas, except no risk on Fortuna Island. |
| Vatican City | No | *Yes* | **vCJD:**  (a) Defer DoD affiliated personnel (anyone with access to a DoD commissary in Europe) if they resided in the country for a cumulative period of 6 months or more from 1980-1996. For DoD affiliated personnel who resided in the country after 1 Jan 1997, (b) applies.  (b) Defer any donor that resided in the country for a cumulative period of 5 years or more from 1980 to present. |
| Venezuela | Yes | No | **Malaria:** Risk exists in rural areas of the following states: Apure, Amazonas, Barinas, Bolivar, Sucre, Tachira, and Delta Amacuro. |
| Vietnam | Yes | No | **Malaria:** Risk in rural areas only, except no risk in the Red River Delta and the coastal plain north of Nha Trang. |
| Virgin Islands, British | No | No |  |
| Virgin Islands, U.S. | No | No |  |
| Wake Island (U.S.) | No | No |  |
| Wales (U.K.) | No | *Yes* | **vCJD**:  (a) Defer if donor has spent a cumulative time of three months or more from 1980 through 1996 anywhere in the U.K. For DoD affiliated personnel and all other donors who only resided in the country after 1 Jan 1997, donors are not deferred.  (b) Defer if donor received a transfusion in the U.K. since 1980. |
| Wallis and Futua Islands | No | No |  |
| Yemen | Yes* | No | **Malaria:** Risk in all areas |
| Yugoslavia (Federal Republic includes Kosovo, Montenegro, and Serbia) | No | *Yes* | **vCJD:**  (a) Defer DoD affiliated personnel (anyone with access to a DoD commissary in Europe) if they resided in the country for a cumulative period of 6 months or more from 1980-1996. For DoD affiliated personnel who resided in the country after 1 Jan 1997, (b) applies.  (b) Defer any donor that resided in the country for a cumulative period of 5 years or more from 1980 to present. |
| Zaire | Yes | No | Malaria: Risk in all areas. |
| Zambia | Yes | No | Malaria: Risk in all areas. |
| Zimbabwe (Rhodesia) | Yes | No | **Malaria:** Risk in all areas, except no risk in cities of Harare and Bulawayo. |
